# Influence of Benthic Macrofauna as a Spatial Structuring Agent for Juvenile Haddock (*Melanogrammus aeglefinus*) on the Eastern Scotian Shelf, Atlantic Canada

**DOI:** 10.1371/journal.pone.0163374

**Published:** 2016-09-20

**Authors:** Beatriz Rincón, Ellen L. Kenchington

**Affiliations:** Department of Fisheries and Oceans, Bedford Institute of Oceanography, Dartmouth, Nova Scotia, Canada; University of Hyogo, JAPAN

## Abstract

We examined the habitat of juvenile haddock on the eastern Scotian Shelf (off Nova Scotia, Canada) in relation to grab-sampled benthic macrofaunal invertebrate species assemblages in order to determine whether there were significant differences in benthic macrofauna between areas of historically persistent high and low juvenile haddock abundance. Our analyses were conducted over two spatial scales in each of two years: among banks (Emerald, Western and Sable Island), approximately 60 km distant from each other, and between areas of high and low juvenile haddock abundance at distances of 10 to 30 km–all in an area that had not experienced groundfishing in the decade prior to sampling. We also examined fine-scale (10s of metres) within-site variability in the macrofauna and used surficial sediment characteristics, along with hydrographic variables, to identify environmental correlates. PERMANOVA identified statistically significant differences in biomass, density and composition of the benthos associated with juvenile haddock abundance; however it was difficult to determine whether the results had biological relevance. Post hoc tests showed that these differences occurred only on Sable Island Bank where both fish and benthos may have been independently responding to sediment type which was most different there (100% sand in the area of low haddock abundance vs. 22% gravel in the area of high haddock abundance). In total, 383 benthic taxa representing 13 phyla were identified. Annelida was the most specious phylum (36.29% of taxa, representing 33 families), followed by Arthropoda (with Crustaceans, mostly Amphipoda, accounting for 25.07% of the total number of taxa). The strongest pattern in the macrofauna was expressed at the largest scale, between banks, accounting for approximately 25% of the variation in the data. Emerald Bank, deeper, warmer and saltier than the Western and Sable Island Banks, had a distinctive fauna.

## Introduction

As in other vertebrates, the spatial structure of fish populations is shaped both by factors endogenous to the populations and by habitat heterogeneity [1]. In marine ecosystems, currents and water masses play major roles in determining fish distributions at large spatial scales (100s of kms), while smaller-scale structure is often attributed to endogenous behavioral responses [2–9]. Within populations, heterogeneities of seabed habitat, both biotic and abiotic, have generally been under-studied as a spatial-structuring agent for boreal fishes living on continental shelves. Exceptions include those species that clearly utilize particular habitats for spawning (e.g., herring *Clupea harengus*) or burrowing (e.g., sand eels *Ammodytes* spp.). Yet, it is well known that benthic species and habitats play a critical role in the population dynamics of some marine fish [10,11], especially the juveniles. Benthic invertebrates are known to provide food [10–13], while biotic and abiotic benthic habitat can provide refuge [10,14].

Establishing a relationship between fish density and the spatial structuring of benthic species and habitats requires joint collection of data over the range of spatial scales relevant to the fish populations. A number of studies have related fish distribution to the presence of structure-forming benthic species such as corals and sponges [15,16], or to particular physical habitats [17–19], but few studies have examined the spatial structuring of benthic species and habitats over the range of spatial scales relevant to fish stock units. Recently, Sell and Kröncke [20] found a correlation between benthic species distributions and demersal fish assemblages on the Dogger Bank (North Sea), which is one of the few temperate areas where the benthos and the fishery resources have been surveyed over a similar spatial extent. There, the two assemblages showed similar spatial structuring, likely arising through common responses to depth and sediment type. In some instances, individual fish species showed spatial correlation with invertebrate prey species.

If benthic invertebrate species distributions impose spatial structure within continental-shelf fish populations, then the relationship is most likely to be identified in those demersal fish assemblages which live most intimately with the sea floor [20], and especially in those species which have benthic life-history stages. In 2000, Canada’s Department of Fisheries and Oceans (DFO) commenced a multiyear, multidisciplinary project on the Scotian Shelf to investigate the role of seabed habitat for a demersal fish species and to test remotely-sensed seabed classification systems [19,21]. Fish on the Scotian Shelf are distributed independently along environmental gradients, rather than comprised of highly co-evolved, inter-dependent species [22]. Consequently, a single key species, haddock (*Melanogrammus aeglefinus*), rather than a fish assemblage was selected for study.

Haddock is a commercially harvested gadoid that is intimately associated with the seabed. It is found in the North Atlantic at depths from less than 50 m to about 350 m and temperatures ranging from 4–8°C [23]. On the Scotian Shelf, haddock spawn on gravel bottoms in April/May. The young have a 4-to-5 month pelagic phase, before moving to the seabed for the remainder of their juvenile stage [24,25]. That shift from a pelagic to a benthic existence occurs at approximately 8 cm length and is reflected in their diet [26]. Haddock populations typically exhibit highly temporally variable recruitment [22], which is commonly attributed to the effects of the environment and food supply during the pelagic phase [12], although the period of transition to the seabed and the following months as benthic juveniles have been cited as a determinant of year-class strength [3].

The haddock on the eastern Scotian Shelf are considered as a single management unit, with a range that formerly included the southern Gulf of St. Lawrence (encompassing Northwest Atlantic Fisheries Organization (NAFO) Divisions 4TVW), and as distinct from adjacent stocks on the western Scotian Shelf and north of the Laurentian Channel. In recent decades, the majority of eastern Scotian Shelf haddock have lived, and spawned, on the offshore banks from Emerald Bank in the west to Banquereau Bank in the east. In 1984, DFO closed a large area (within NAFO Division 4W) to trawlers in an attempt to reduce discarding of undersized haddock [27,28]. In 1987, a larger area on Western and Emerald Banks, reaching ≈12,776 km^2^ or 13% of the area occupied by the population [28], was closed year round to most groundfish fishing (though not to long-lining with large hooks for part of this time, nor to scallop dragging which was very light in this region and outside of the study areas). In September 1993, the fisheries for cod and haddock on the eastern Scotian Shelf were closed, to protect the depleted stocks, and remain so today. Those closures allowed a rare opportunity to sample benthic species and physical habitats on the fishing grounds of the eastern Scotian Shelf in a relatively undisturbed state.

We used that opportunity to examine the role of benthic macrofaunal communities as a spatial-structuring agent for juvenile haddock on the eastern Scotian Shelf. While other studies have focused on benthic habitat features [15–18] or on broad correlation of species distributions [20], we took a different approach. Areas (100 km^2^) of persistent high and low juvenile abundance were identified on each of three offshore banks within the area occupied by the stock unit, based on the probability of encountering juvenile haddock determined through the analyses of 32 years of data from the DFO summer groundfish surveys [21]. We then sampled the benthic macrofaunal invertebrate communities in those areas in each of two years to address the question: Do areas of preferred juvenile haddock habitat (areas of persistent high abundance) differ in benthic macrofaunal species composition from those that are not utilized as much (areas of persistent low abundance)?

Our intensive, temporally replicated grab-sampling also provided new data on the benthic macrofaunal communities of the eastern Scotian Shelf. In general, the benthic macrofauna of the offshore Scotian Shelf are poorly studied, and our study is also the first to compare those communities over large spatial scales. The grab-sampled macrofauna on Western and Banquereau Banks have been examined previously over small spatial scales in relation to fishing impacts [29–31], while others [32,33] minimally extended their own studies to the north and south respectively onto the Scotian Shelf. We additionally examined surficial sediment distribution, combined with dynamic bed-form characteristics (e.g., sandwaves) interpreted from sidescan sonar mosaics [34], along with hydrographic characteristics, to interpret the fine-scale (10s of metres) within-site and larger-scale (between sites within banks, between banks) macrofaunal patterns.

## Materials and Methods

### Study Area

**Fig 1:**
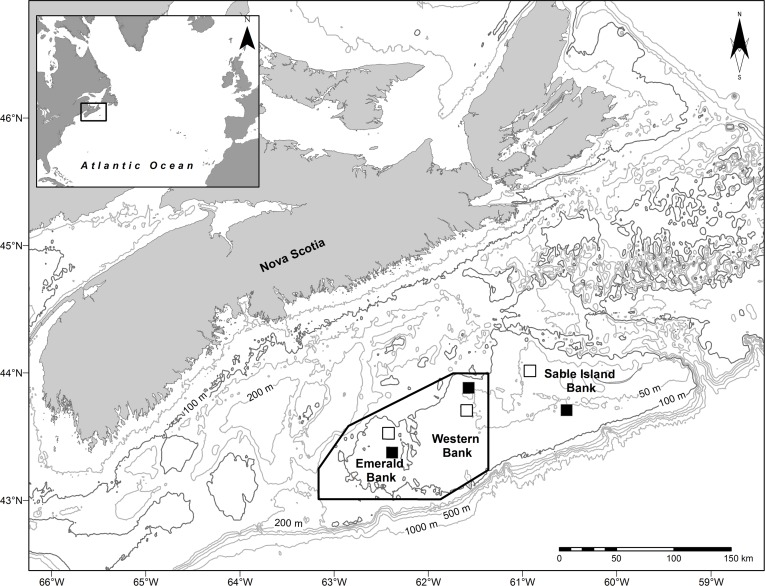
Map showing the locations of the three banks and six study areas on the Scotian Shelf. Black and white solid squares represent Low and High Haddock abundance areas respectively. Depth contours are shown for 50, 100 (darker shade), 150, 200, 300, 500 and 1000 m. Black polygon shows the area closed to groundfish fishing in 1987.

The Scotian Shelf is the portion of northwest Atlantic continental shelf lying off Nova Scotia, Canada, between the Laurentian and Northeast Channels. It is approximately 700 km long and between 125 and 230 km wide, and characterized by highly productive marine ecosystems and complex bottom topography [35]. The Laurentian Channel delimits the Scotian Shelf in the northeast, while to the southwest the Northeast Channel separates it from Georges Bank. The outer part of the Scotian Shelf is characterized by a number of large, shallow banks separated by transverse troughs; Sable Island Bank rising above the surface as its eponymous island [35]. The present study focused on three outer Scotian Shelf banks; Emerald, Western and Sable Island Banks (Fig 1).

Historically these banks were heavily exploited, having a fishing history dating back to the 17^th^ century. Groundfish, particularly Atlantic cod (*Gadus morhua*) and haddock, were the mainstay of the 20^th^ century fisheries [36]. Overfishing and environmental variability led to population collapses, which affected both fisheries and trophic structure. Most groundfish fisheries were closed in September 1993 as noted previously, but recovery has not followed expected trajectories [37].

### Sampling Design

Catches of juvenile haddock (including all younger than the age of sexual maturity) taken by summer research-vessel bottom-trawl surveys on Emerald, Western and Sable Island Banks from 1970 to 2001 had previously been mapped and areas of persistent high and low density identified [21]. While spatially variable within each bank, juvenile densities were similar on Emerald and Western Banks, and only slightly lower on Sable Island Bank [19]. Habitat utilization changes over time, however. In particular, when abundance is high, haddock expand over a larger area, apparently occupying less preferred habitats [27] and obscuring the relationship between optimal habitat types and fish distributions. We therefore focused on longer-term relationships, captured in the 32-year span of the survey data. In the event, a very strong 1999 year class of haddock was broadly spread across the Scotian Shelf during the period of our field program [21, 38]. Hence, spatial variations in the short-term responses of the benthos to the fish and vice versa may have been blurred.

Three pairs of 100 km^2^ study sites, one pair on each bank (Table 1, Fig 1), were selected as representative of areas with persistently the highest and lowest juvenile haddock densities, respectively within each bank [21]. We designated the members of each pair as “High” or “preferred” and as “Low” or “non-preferred” areas. Survey trawling for fish prior to benthic sampling in 2002 and in the last year of the study in 2005 found that the densities of juvenile haddock in the High sites on Western and Sable Island Banks were approximately an order of magnitude greater than those in the paired Low sites, consistent with the differential densities seen in the 1970–2001 survey data, but densities on the two Emerald Bank sites were equal [38]. On Emerald and Western Banks the paired sites were approximately 10 km distant from one another, while on Sable Island Bank they were approximately 30 km apart. Within each 100 km^2^ study site a 1 km x 5 km swath was randomly selected for detailed study.

**Table 1:** Summary of the sampling design and associated environmental data.

Bank	Haddock Abundance	Year	Number of Grab Samples	Average Depth (m)	Mean Bottom Salinity	Mean Bottom Temp. (°C)	Mean Bottom Current (m s^-1^)
Emerald	High	2003	9	77.42	34.07	7.70	0.0158
		2005	12	75.85	34.07	7.69	0.0158
	Low	2003	9	83.09	34.04	7.71	0.0170
		2005	8	80.02	34.04	7.72	0.0169
Western	High	2003	10	58.38	32.60	5.77	0.0163
		2005	11	59.15	32.58	5.79	0.0162
	Low	2003	10	52.96	33.34	5.35	0.0152
		2005	11	54.00	33.34	5.36	0.0152
Sable Island	High	2003	10	44.26	32.40	4.47	0.0193
		2005	12	44.39	32.41	4.46	0.0192
	Low	2003	10	56.15	32.46	5.79	0.0190
		2005	6	54.58	32.39	5.79	0.0190

### Benthos Sampling

**Fig 2:**
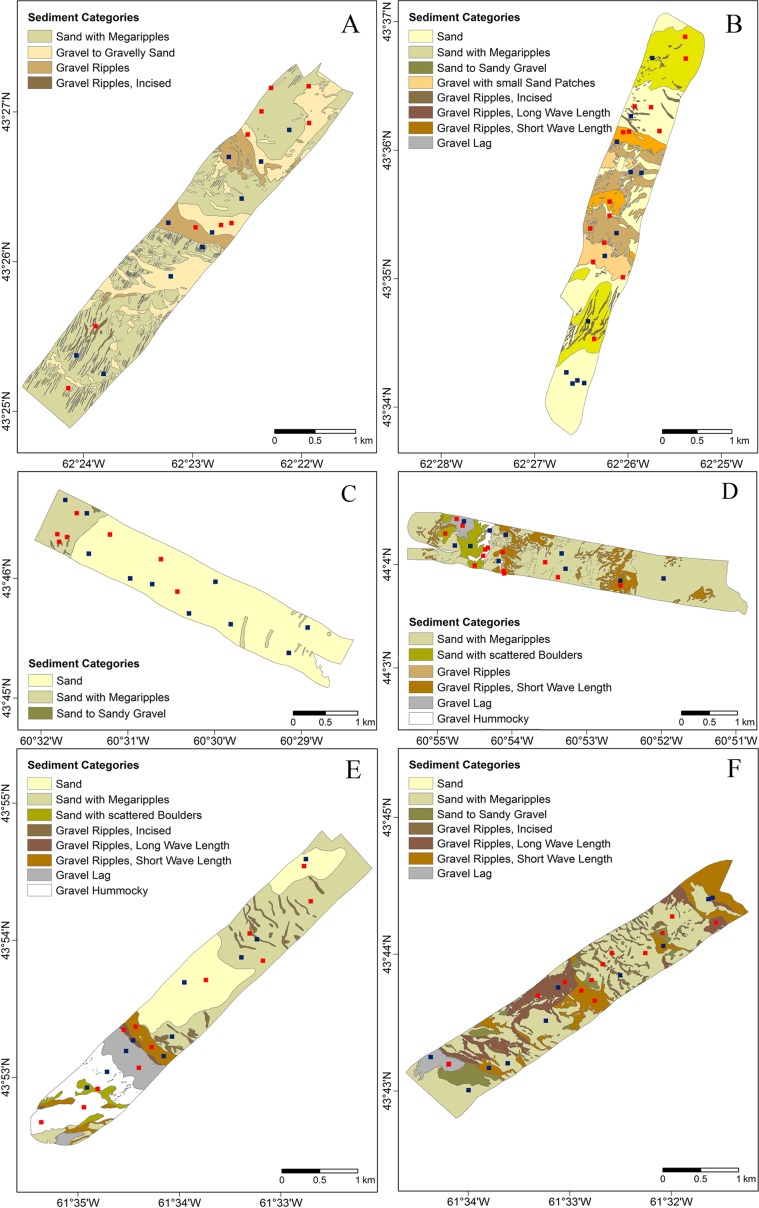
Distribution of surficial sediment types and video-grab sample locations within the surveyed swaths in each study site. Emerald Bank, Low Haddock Abundance site (A); Emerald Bank, High Haddock Abundance site (B); Sable Island Bank Low Haddock Abundance site (C); the Sable Island Bank High Haddock Abundance site (D); Western Bank, Low Haddock Abundance site (E); and Western Bank, High Haddock Abundance site (F). Surficial sediment types were interpreted from sidescan sonar [34]. Squares indicate video-grab sample locations (blue: 2003; red: 2005).

Between 6 and 12 grab samples were taken from each swath in each of 2003 and 2005 (Table 1, Fig 2). Benthic invertebrates were sampled with a video-grab, an electro-hydraulically actuated grab fitted with video cameras and halogen lights, which samples a 0.5 m^2^ area of seafloor [39,40]. The cameras were used to increase sampling efficiency by ensuring that the bottom was suitable for sampling and that the grab closed properly before being recovered. An ORE Trackpoint II ultra-short baseline acoustic tracking system was used to determine the position of the video-grab relative to the ship [41]. No permits were required to undertake this sampling and no endangered or protected species were collected in the samples.

The video-grab contents were washed over a 1 mm screen, with retained material being preserved in buffered formalin on board ship. After return to the laboratory, all organisms were identified to species level, where possible. Poor condition of specimens, lack of information about juvenile forms or gaps in taxonomic knowledge prevented species-level identifications for some specimens. Abundance and biomass (formalin wet weight, including both mantle cavity liquid and shells for molluscs) were determined for each taxon.

### Environmental Data

Depth and sediment type were determined for each grab sample, as were the estimates of mean bottom salinity, temperature and current (S1 Table, Fig 2). Depth was recorded from the ship’s sounder. Five sand and nine gravel sediment types had previously been interpreted from sidescan sonar mosaics and towed video observations on each swath. The categories were defined by sediment grain size and superimposed dynamic bedforms [34,42]. Sediment types were: Sand (S); Sand with Megaripples (SM); Sand to Sandy Gravel (SG); Sand with Scattered Boulders (SB); Sand Ribbons (SR); Gravel (G); Gravel to Gravelly Sand (GS); Gravel Ripples (GR); Gravel Ripples, Short Wave Length (GRS); Gravel Ripples, Long Wave Length (GRL); Gravel Ripples, Incised (GRI); Gravel Lag (GL); Gravel, Hummocky (GH); and Gravel with Small Sand Patches (GSP), although not all categories occurred in patches large enough to be visible on the maps presented here (Fig 2). Additionally, zones of boulders and sand ribbons, individual boulders, and the orientations of bedform crests were mapped [34]. Each grab sample was associated with one of these categories, based on its mapped position (S1 Table). The Sable Island Bank Low swath was the most homogeneous, with only categories S, SM, and SG present; the matching High site being mostly sand (78%), though with 4 gravel categories present (Fig 2). The Emerald Bank sites contained both sand and gravel categories (Fig 2), the Low site being 60% sand and the High site, 71%. The Western Bank High site was 60% sand, and the Low site there was 66% sand. Overall, Western Bank had the greatest diversity of sediment types (S1 Table, Fig 2).

Due to the absence of direct measurements, data for mean bottom temperature, salinity, and current speed were extracted from interpolated surfaces extracted from the Global Ocean Reanalysis and Simulations (GLORYS) model by Dr. Z. Wang, Oceans and Ecosystem Science Division, Bedford Institute of Oceanography. GLORYS is a numerical general circulation model with 1/4° resolution. The model used observational data collected from 1992 to 2011. Values for each sample were drawn from the interpolated surfaces for each variable (created with ordinary kriging) in ArcGIS v. 10.1 (Environmental Systems Research Institute, Redlands, CA).

### Statistical Analyses

Species accumulation curves were constructed with PRIMER version 6.1.6 [43] separately for each study site and year to ensure that the benthic assemblages were adequately sampled prior to analysis. The number of observations (Sobs) was permutated 999 times to produce standard deviations.

The Total Number of Taxa (S), Total Abundance of organisms (individuals m^-2^), Total Biomass of organisms (g m^-2^), Pielou’s Evenness Index (J’) and Shannon-Wiener’s Diversity Index (H’) were determined. Levene’s statistic was used to test the null hypothesis that the group variances were equal. Most variables had homogeneous variances (*P* ≥ 0.01) without transformation, however total biomass required ln (x+1)-transformation to achieve homogeneity of variances. Their variations were tested using full factorial univariate ANOVAs (performed with IBM SPSS Statistics, version 20.0.0, IBM Corporation, Somers, New York), with three fixed factors: (Juvenile) Haddock Abundance (2 levels: High, Low); Bank (3 levels: Emerald, Sable Island, Western); and Year (2 levels: 2003, 2005). Sums of squares were calculated taking the interaction terms into consideration. Tukey’s HSD was used to test for equality of group means in post hoc tests of significant factors and interactions. Statistical significance was evaluated after Bonferroni correction for multiple tests (α ≤ 0.001).

Bray-Curtis similarities were calculated on the ln (x+1)-transformed species abundance and biomass data and on the untransformed presence/absence of species (including colonial taxa). Prior to statistical analyses, taxa which contributed ≤ 1% of total abundance in each sample year were removed to reduce the effect of rarities on the analyses [44]. The effects of setting the removal criterion at ≤ 3% or ≤ 5% were examined but results did not differ from those which arose with a ≤ 1% cut off. Using the same design as for univariate ANOVA, permutation multivariate analyses of variances (PERMANOVAs) were conducted on each matrix with 999 permutations [45]. Permutation of residuals was performed under a reduced model [46] and permutated pairwise tests of significant factors were conducted. Statistical significance was evaluated after Bonferroni correction for multiple tests (α ≤ 0.001).

nMDS plots were used to visualize variations related to significant factors. Similarity percentages tests (SIMPER) were used to determine the macrofaunal taxa that contributed most to significant dissimilarities among factors. PERMANOVA, nMDS and SIMPER routines were implemented in PRIMER-E (Plymouth Routines in Multivariate Ecological Research; Primer-E Ltd., 3 Meadow View, Lutton, Ivybridge, UK).

The relationships between the environmental parameters (depth, sediment type (categorical variable), mean bottom temperature, salinity and current) and the abundance and biomass of the benthic communities were examined using a distance-based linear model (DISTLM) and distance-based redundancy analysis ordination (dbRDA) performed on the Bray-Curtis similarity matrices. The best selection procedure was run with 9999 permutations and with the adjusted R^2^ selection criterion implemented in PRIMER-E.

## Results

### Description of the Benthos

A total of 383 benthic taxa representing 13 phyla were identified (S2 Table). Of those, 52 species were only observed once and 30 were only observed twice. The samples collected in 2005 showed greater abundance and biomass and presented 52 more species than those collected in 2003. The Annelida was the most speciose phylum (36.3% of taxa, representing 33 families), followed by Arthropoda (with Crustaceans, mostly Amphipoda, accounting for 25.1% of the total number of taxa), Mollusca (19.1%—mostly Bivalvia and Gastropoda), Cnidaria (7.8%), Echinodermata (5.0%), Bryozoa (2.9%), Chordata (1.0%—mostly genera of Ascidians), and seven other phyla accounting for 2.9% of the total. Polychaetes and amphipods prevailed in abundance with 10 to 13 species accounting for 50% of the total, whereas bivalves and echinoderms prevailed in biomass, with 4 to 7 species accounting for 90% of the total. The three most frequently sampled species, based on presence/absence data, were the amphipod *Unciola irrorata*, and the polychaetes *Ampharete finmarchica* and *Clymenura borealis*.

The average Number of Taxa (S) found in each of the six study sites ranged from 44 to 69 (2003) and from 44 to 73 (2005). The highest numbers of taxa were found within Western Bank samples; two grab samples from the Low Haddock Abundance site sampled in 2003 yielded the highest number of taxa (126 and 109 species). Average Total Abundance ranged from 779 individuals m^-2^ (Emerald Bank, High Haddock Abundance) to 2,578 individuals m^-2^ (Western Bank, High Haddock Abundance) in 2003 and from 1,759 individuals m^-2^ (Sable Island Bank, Low Haddock Abundance) to 2,406 individuals m^-2^ (Western Bank, Low Haddock Abundance) in 2005. The top four most abundant taxa in both years were the bamboo worm *Clymenella zonalis*, the amphipod *Unciola irrorata*, and the polychaetes *Polygordius* sp. and *Chone* sp. Average Total Biomass ranged from 0.0176 kg m^-2^ (Emerald Bank, High Haddock Abundance) to 12.116 kg m^-2^ (Sable Island Bank, Low Haddock Abundance) in 2003 and from 0.212 kg m^-2^ (Emerald Bank, High Haddock Abundance) to 44.538 kg m^-2^ (Western Bank, High Haddock Abundance) in 2005. The highest biomass was found for echinoderms and bivalves, with the sea cucumber *Cucumaria frondosa*, the propeller clam *Cyrtodaria siliqua*, and the sand dollar *Echinarachnius parma* showing the highest biomass per species.

### Effects of Haddock Abundance, Bank and Year on Diversity Indices

Species-accumulation curves for each bank and area within bank approached the asymptote suggesting that sampling was adequate to compare species richness among stations (S1 Fig). Shannon’s Diversity (H’), Pielou’s Evenness Index (J’) and the Total Abundance of organisms (individuals m^-2^) showed no significant differences among factors (S2 Fig) or their interactions in the univariate ANOVAs. A significant model effect was found for the Total Number of Taxa (S) but none of the individual effect tests were significant. Total Biomass of organisms (g m^-2^) was significantly different among Banks, among levels of Haddock Abundance and in the interaction of those factors (S3 Table). Post hoc tests revealed significantly lower transformed macrofaunal biomass on Emerald Bank than on the other banks and in the areas where there was High Haddock Abundance versus Low (S3 Table). The significant interaction between these factors resulted from the High Haddock Abundance site on Sable Island Bank grouping with the Emerald Bank sites, and the Low Haddock Abundance site with the Western Bank sites as indicated by Tukey’s HSD post hoc test; the trends between High and Low Haddock Abundance within each Bank were all in the same direction.

### Community Analyses

#### Macrofaunal Species Abundance

PERMANOVA of the transformed abundance of the macrofauna showed an interaction between the factors Haddock Abundance and Bank, and one between Bank and Year; all other interactions terms were non-significant (Table 2). Each of the three factors was significant, with Bank explaining the largest proportion of the variance, followed by Year and Haddock Abundance (Table 2). Post hoc pairwise tests identified significant differences between every combination of main-effect factor levels. The Haddock Abundance x Bank interaction identified significant differences between High and Low Haddock Abundance within Sable Island Bank but not in the other banks. The Bank x Year interaction had significant differences between all combinations.

**Table 2:** PERMANOVA of the transformed abundance of macrofaunal taxa based on Bray-Curtis similarity.

Source	Degrees of Freedom	Sums of Squares (SS)	Mean Square (MS)	Pseudo-*F*	*P*_*(Perm)*_	Est. of Variance Component	Sq. Root of Variance Component
Haddock Abundance (HA)	1	7421.3	7421.3	4.571	0.001	100.15	10.01
Bank (BA)	2	50231	25116.0	15.469	0.001	606.97	24.64
Year (YR)	1	8271.1	8271.1	5.094	0.001	114.82	10.72
HAxBA	2	19322.0	9661.1	5.951	0.001	415.33	20.38
HAxYR	1	2892.2	2892.2	1.781	0.043	43.83	6.62
BAxYR	2	9053.7	4526.9	2.788	0.001	150.03	12.25
HAxBAxYR	2	3305.0	1652.5	1.018	0.401	2.99	1.73
Residual	108	1.75 x10^5^	1623.6			1623.60	40.29
Total	119	2.78 x10^5^					

**Fig 3:**
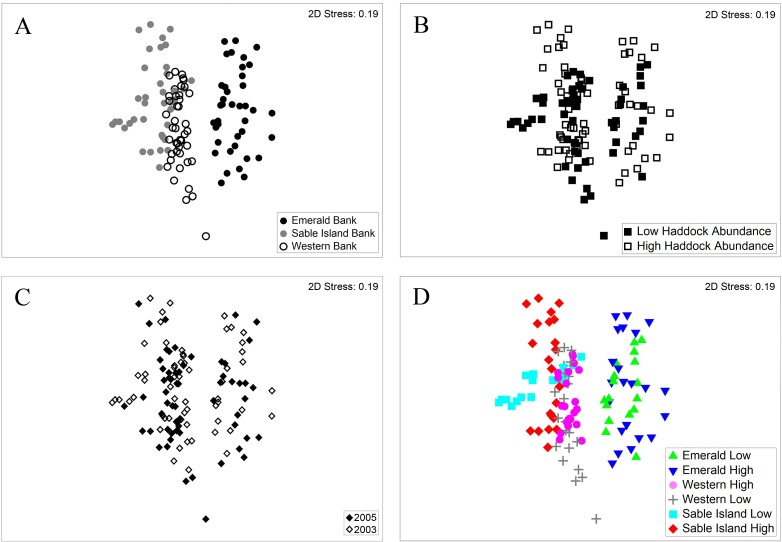
Non-metric multidimensional scaling (nMDS) of transformed abundance-based Bray Curtis similarities. (A) nMDS labelled by Bank; (B) nMDS labelled by Haddock Abundance; (C) nMDS labelled by Year; (D) nMDS labelled by Bank and Haddock Abundance levels.

nMDS plots confirmed the greater explanatory power of the Bank factor over Haddock Abundance (Fig 3). Labelling of the samples by Year showed no change to the observed pattern with large overlap between years within banks (Fig 3). SIMPER identified a high degree of variability among grab samples within each Bank, with average similarity ranging from 36.10% on Emerald Bank to 43.5% on Western Bank (Table 3). Ten taxa contributed to 50% of that variability on Emerald Bank, with 14 taxa accounting for that level on each of Sable Island and Western Banks. In the associated taxon lists (Table 3), only Emerald Bank had no molluscs or echinoderms present in that percentage, and variability was influenced by small polychaetes and crustaceans. The small amphipod *Ericthonius fasciatus* was not found in Sable Island Bank samples and accounted for 2% of the variation between Emerald and Sable Island Bank (Table 4). These two areas were the most dissimilar (75.84%; Table 4) and the dissimilarity was drawn from a large number of species, with 12 taxa contributing to just 20% of the total. Emerald and Western Banks had dissimilar community composition (71.39%) with 11 taxa contributing to 20% of the total. A maldanid polychaete *Clymenella zonalis* and the small amphipod *Ericthonius fasciatus* contributed to 4% of the total dissimilarity, with *E*. *fasicatus* present in the Western Bank samples but at lower abundance than in the Emerald Bank samples (Table 4). Western and Sable Island Banks were less dissimilar (65.10%) and the single taxon contributing most to that difference was again the maldanid polychaete *C*. *zonalis* (Table 4). SIMPER analysis of the two levels of Haddock Abundance showed that they were 68% dissimilar with 127 taxa contributing to 90% of that variation and all differences between those due to proportional abundance differences between the two groups and not absence of taxa. The maldanid polychaete *C*. *zonalis* explained the highest proportion of the variation of the individual taxa, although that was only 2%. This species was present in greater abundance in the samples from areas where Haddock Abundance was high.

**Table 3:** SIMPER analysis of transformed abundance of macrofauna within Emerald, Sable Island and Western Banks.

Bank (%Sim.)	Taxon	Av. Abund.	Av. Sim.	Sim./SD	Cont.%	Cum.%
Emerald	*Unciola irrorata*	2.75	2.81	1.71	7.78	7.78
(36.10%)	*Clymenura borealis*	2.39	2.62	1.00	7.26	15.04
	*Ericthonius fasciatus*	2.67	2.11	1.06	5.85	20.90
	*Chone* sp.	2.77	1.99	1.15	5.50	26.40
	*Aglaophamus circinata*	1.63	1.86	1.69	5.16	31.55
	*Glycera capitata*	1.88	1.74	1.50	4.82	36.38
	*Lumbrinerides acuta*	1.54	1.18	0.92	3.27	39.65
	*Ampharete finmarchica*	1.32	1.12	1.17	3.11	42.75
	*Exogone* sp.	1.72	0.98	0.63	2.71	45.46
	Syllidae	1.69	0.96	0.66	2.66	48.13
Sable Island	*Ampharete finmarchica*	2.86	2.14	1.74	5.71	5.71
(37.55%)	Nemertea	2.01	1.77	1.49	4.73	10.43
	*Lumbrinerides acuta*	2.13	1.66	0.78	4.43	14.86
	*Unciola irrorata*	2.75	1.60	1.05	4.27	19.13
	*Clymenura borealis*	1.94	1.37	1.25	3.65	22.79
	*Spiophanes bombyx*	2.07	1.32	1.16	3.52	26.30
	*Echinarachnius parma*	2.03	1.29	1.07	3.43	29.74
	*Ophelia limacina*	1.51	1.23	1.05	3.28	33.02
	Ascidiacea (solitary)	1.93	1.22	0.83	3.24	36.26
	*Scoloplos armiger*	1.63	1.08	0.81	2.87	39.14
	*Tharyx* sp.	1.70	1.08	0.98	2.87	42.01
	*Clymenella zonalis*	2.34	0.98	0.71	2.60	44.61
	*Nereis* sp.	1.70	0.96	1.15	2.55	47.17
	*Aglaophamus circinata*	1.72	0.89	0.82	2.37	49.54
Western	*Unciola irrorata*	3.98	2.79	2.07	6.41	6.41
(43.53%)	*Clymenella zonalis*	3.55	2.01	1.57	4.63	11.04
	*Edwardsia elegans*	2.59	1.86	1.70	4.26	15.30
	*Ampharete finmarchica*	2.49	1.74	1.59	4.00	19.30
	*Lumbrinerides acuta*	2.37	1.67	1.15	3.84	23.14
	Nemertea	2.38	1.50	1.95	3.44	26.57
	*Echinarachnius parma*	1.94	1.35	0.99	3.09	29.67
	*Clymenura borealis*	2.08	1.33	1.02	3.05	32.71
	Oligochaeta	2.33	1.26	1.15	2.89	35.60
	*Polygordius* sp.	2.60	1.20	0.92	2.77	38.37
	*Paraonis* sp.	2.05	1.18	1.60	2.72	41.09
	*Cyrtodaria siliqua*	1.90	1.18	1.40	2.71	43.80
	*Exogone* sp.	2.16	1.14	1.26	2.63	46.43
	*Tharyx* sp.	2.03	1.06	1.11	2.44	48.86

Table limited to the taxa contributing to 50% of the similarity among samples within banks.

Abbreviations: (% Sim.), total similarity among samples expressed as a percentage; Av.Abund., average abundance; Av. Sim., average contribution to the total similarity; Sim./SD, average contribution to the total similarity divided by standard deviation; Cont%, percentage contribution to similarity; Cum%, cumulative percentage contribution of contribution to similarity.

**Table 4:** SIMPER analysis of transformed abundance of macrofauna between Banks.

Bank (%Diss.)	Taxon	Av. Abund. (1)	Av. Abund. (2)	Av. Diss.	Cont.%	Cum.%
Emerald (1)	*Ericthonius fasciatus*	2.67	0.00	1.67	2.20	2.20
Sable Island (2)	*Chone* sp.	2.77	0.43	1.54	2.03	4.23
(75.84%)	*Clymenella zonalis*	1.61	2.34	1.43	1.88	6.11
	*Unciola irrorata*	2.75	2.75	1.39	1.83	7.94
	*Ampharete finmarchica*	1.32	2.86	1.22	1.61	9.55
	*Echinarachnius parma*	0.18	2.03	1.20	1.59	11.14
	*Lumbrinerides acuta*	1.54	2.13	1.19	1.57	12.71
	*Spiophanes bombyx*	0.34	2.07	1.15	1.52	14.23
	*Clymenura borealis*	2.39	1.94	1.15	1.52	15.75
	Ascidiacea (solitary)	1.01	1.93	1.12	1.48	17.23
	*Glycera capitata*	1.88	0.36	1.10	1.45	18.68
	*Exogone* sp.	1.72	0.17	1.06	1.40	20.08
Emerald (1)	*Clymenella zonalis*	1.61	3.55	1.58	2.21	2.21
Western (2)	*Ericthonius fasciatus*	2.67	0.39	1.44	2.01	4.22
(71.39%)	*Chone* sp.	2.77	1.64	1.33	1.86	6.08
	*Polygordius* sp.	1.18	2.60	1.31	1.84	7.92
	*Unciola irrorata*	2.75	3.98	1.25	1.75	9.67
	*Edwardsia elegans*	0.70	2.59	1.20	1.68	11.35
	*Echinarachnius parma*	0.18	1.94	1.17	1.63	12.99
	Oligochaeta	0.78	2.33	1.16	1.63	14.61
	*Exogone* sp.	1.72	2.16	1.10	1.54	16.15
	*Glycera capitata*	1.88	2.31	1.08	1.51	17.66
	*Cyrtodaria siliqua*	0.07	1.90	1.07	1.50	19.16
Sable Island (1)	*Clymenella zonalis*	2.34	3.55	1.44	2.22	2.22
Western (2)	*Unciola irrorata*	2.75	3.98	1.27	1.95	4.17
(65.10%)	*Polygordius* sp.	1.64	2.60	1.16	1.78	5.95
	*Edwardsia elegans*	1.77	2.59	1.09	1.67	7.62
	*Glycera capitata*	0.36	2.31	1.04	1.61	9.22
	Oligochaeta	1.17	2.33	1.00	1.54	10.77
	*Exogone* sp.	0.17	2.16	1.00	1.54	12.30
	*Protomedeia fasciata*	0.55	2.05	0.89	1.37	13.67
	*Lumbrinerides acuta*	2.13	2.37	0.87	1.34	15.01
	Syllidae	0.49	1.97	0.87	1.33	16.35
	*Spiophanes bombyx*	2.07	1.51	0.87	1.33	17.68
	*Chaetozone* sp. A	0.70	1.86	0.86	1.32	18.99
	*Arctica islandica*	1.44	1.16	0.85	1.30	20.30

Table limited to the taxa contributing to 20% of the dissimilarity between banks.

Abbreviations: %Diss., total dissimilarity between banks expressed as a percentage; Av.Abund., average abundance; Av. Diss., average contribution to the total dissimilarity; Cont%, percentage contribution to dissimilarity; Cum%, cumulative percentage contribution of contribution to dissimilarity.

The interaction between Haddock Abundance and Bank also explained a high proportion of the variability in the data (Table 5, Fig 3), being second only to the Bank factor (Fig 3). The nMDS plot (Fig 3) showed spatial separation of the Haddock Abundance levels on Western and Sable Island Banks, but not on Emerald Bank, although the levels of dissimilarity between levels on each Bank were all greater than 57% (Table 5). The pairwise permutations noted above identified significant differences only on Sable Island Bank. SIMPER identified relative proportions in the abundance of taxa as contributing to the differences between High and Low Haddock Abundance on each Bank as opposed to different species present (Table 5). The list of species contributing to 10% of the total dissimilarity between Haddock Abundance levels on each Bank is provided in Table 5. On Sable Island Bank, where the differences were most pronounced (average dissimilarity = 69.29%), the ocean quahog *Arctica islandica* and the sand dollar *Echinarchnius parma*, were much more abundant in the areas where Haddock Abundance was Low, while the polychaetes *Lumbrinerides acuta* and *Clymenella zonalis* were much more abundant in the areas of High Haddock Abundance (Table 5).

**Table 5:** SIMPER analysis of transformed abundance of macrofauna between the High and Low Haddock Abundance sites on each bank.

Bank (%Diss.)	Taxon	Av.Abund. (Low HA)	Av.Abund. (High HA)	Av.Diss.	Cont.%	Cum.%
Emerald	*Chone* sp.	2.97	2.61	1.71	2.63	2.63
(64.94%)	*Ericthonius fasciatus*	2.33	2.93	1.60	2.47	5.09
	*Unciola irrorata*	3.82	1.92	1.56	2.40	7.49
	Syllidae	2.49	1.07	1.52	2.35	9.84
Sable Island	*Arctica islandica*	3.05	0.28	1.50	2.16	2.16
(69.29%)	*Lumbrinerides acuta*	0.53	3.28	1.50	2.16	4.33
	*Echinarachnius parma*	3.54	0.94	1.46	2.11	6.44
	*Aglaophamus circinata*	3.17	0.67	1.41	2.04	8.47
	*Clymenella zonalis*	1.98	2.60	1.36	1.96	10.43
Western	*Clymenella zonalis*	3.10	4.01	1.25	2.15	2.15
(57.96%)	*Polygordius* sp.	2.74	2.45	1.17	2.02	4.17
	*Glycera capitata*	1.88	2.76	1.05	1.81	5.99
	*Unciola irrorata*	3.29	4.70	1.05	1.81	7.80
	*Tharyx* sp.	1.22	2.89	0.99	1.70	9.50

Table limited to the taxa contributing to 10% of the dissimilarity between the High and Low Haddock Abundance (HA) sites on each bank. Abbreviations: %Diss., total dissimilarity between banks expressed as a percentage; Av.Abund., average abundance; Av. Diss., average contribution to the total dissimilarity; Cont%, percentage contribution to dissimilarity; Cum%, cumulative percentage contribution of contribution to dissimilarity.

#### Macrofaunal Biomass and Presence/Absence

PERMANOVA of the transformed biomass of the macrofaunal taxa showed an interaction effect between Haddock Abundance and Bank; all other interactions were non-significant (Table 6). As for transformed abundance, all three factors were significant with Bank explaining the largest proportion of the variance, followed by Haddock Abundance with Year explaining the least proportion of the variance (Table 6). The PERMANOVA of the presence/absence data showed interaction effects between Haddock Abundance and Bank, and between Bank and Year; all other interactions were non-significant and all 3 factors were significant (Table 6). As for the other analyses, Bank had the largest variance component. The nMDS configurations for these analyses (not shown) were very similar to that of those generated from abundance data both with respect to Bank separating the samples from one another and with the two levels of Haddock Abundance showing overlapping distributions.

**Table 6:** PERMANOVA of the transformed biomass and untransformed presence/absence of macrofaunal taxa based on Bray-Curtis similarity.

Variable	Source	Degrees of Freedom		Sums of Squares (SS)		Mean Square (MS)		Pseudo-*F*		*P*_*(Perm)*_		Est. of Variance Component		Sq. Root of Variance Component	
Biomass	Haddock Abundance (HA)	1		7625.5		7625.5		4.631		0.001		103.28		10.16	
	Bank (BA)	2		49606.0		24803.0		15.064		0.001		598.29		24.46	
	Year (YR)	1		5862.1		5862.1		3.560		0.001		72.82		8.53	
	HAxBA	2		21583.0		10791		6.554		0.001		472.55		21.74	
	HAxYR	1		2939.9		2939.9		1.786		0.040		44.68		6.68	
	BAxYR	2		7182.7		3591.4		2.181		0.003		100.50		10.03	
	HAxBAxYR	2		3013.3		1506.7		0.915		0.574		-14.46		-3.80	
	Residual	108		1.78 x10^5^		1646.5						1646.50		40.58	
	Total	119		2.79 x10^5^											
Presence/ Absence	Haddock Abundance (HA)	1		4727.0		4727.0		3.893		0.001		60.68		7.79	
	Bank (BA)	2		22520.0		18.5		18.992		0.001		550.48		23.46	
	Year (YR)	1		7182.1		5.9		6.135		0.001		103.09		10.15	
	HAxBA	2		5871.0		4.8		5.027		0.001		240.64		15.51	
	HAxYR	1		2379.1		1.9		1.987		0.025		40.24		6.34	
	BAxYR	2		4170.7		3.4		3.548		0.001		152.78		12.36	
	HAxBAxYR	2		2467.0		1233.5		1.016		0.401		2.00		1.41	
	Residual	108		1.31x10^5^		1214.2						1214.20		34.85	
	Total	119		2.15x10^5^											

#### Macrofauna Contributing to Differences in Areas of High and Low Haddock Abundance

Haddock Abundance showed significant differences in PERMANOVA between High and Low levels in all 3 variables: transformed abundance and biomass, and presence/absence of taxa (Tables 2 and 6). A SIMPER analysis listing those taxa accounting for 20% of the variation between the two levels, for each variable, is provided in Table 7. Areas with High Haddock Abundance were between 58% and 68% different in benthic community composition from areas with Low Haddock Abundance and many species contributed to the differentiation (Table 7). Areas with High Haddock Abundance were characterized by larger numbers of the maldanid polychaete *C*. *zonalis*, the elegant burrowing anemone *Edwardsia elegans*, the polychaete *Lumbrinerides acuta*, the bristle worm *Glycera capitata*, and the amphipod *Ericthonius fasciatus*. Biomass of the elegant burrowing anemone *E*. *elegans*, the bristle worm *G*. *capitata*, the polychaete *Ophelia limacina*, and Cerianthidae was higher in areas with High Haddock Abundance. While the bristle worm *Scoloplos armiger*, the polychaete *O*. *limacina*, the oval spoonclam *Periploma leanum*, Gastropoda, the polychaete *Exogone* sp., the bristle worm *Notomastus latericeus*, Mytiloidea, the bristle worm *Orbinia swani*, the bubble snail *Cylichna alba*, and the moonsnail *Euspira* sp. occurred more frequently in areas with Low Haddock Abundance.

**Table 7:** SIMPER analysis of transformed abundance, transformed biomass and presence/absence of macrofauna between High and Low Haddock Abundance sites across Banks.

		Low HA	High HA			
Variable (%Diss.)	Taxon	Aver.	Aver.	Av.Diss.	Cont.%	Cum.%
Abundance	*Clymenella zonalis*	2.41	2.64	1.42	2.09	2.09
(68.14%)	*Unciola irrorata*	3.32	3.08	1.26	1.85	3.94
	*Polygordius* sp.	1.86	1.81	1.15	1.68	5.62
	*Chone* sp.	1.62	1.62	1.14	1.67	7.29
	*Echinarachnius parma*	1.99	0.90	1.02	1.50	8.79
	*Edwardsia elegans*	1.67	1.76	1.01	1.48	10.27
	*Lumbrinerides acuta*	1.71	2.29	0.99	1.45	11.72
	*Exogone* sp.	1.59	1.22	0.99	1.45	13.17
	*Glycera capitata*	1.47	1.62	0.97	1.42	14.59
	Syllidae	1.69	1.18	0.96	1.41	16.00
	*Clymenura borealis*	2.31	1.99	0.96	1.41	17.41
	Oligochaeta	1.54	1.39	0.95	1.40	18.81
	*Ericthonius fasciatus*	0.72	1.25	0.91	1.34	20.15
Biomass	*Echinarachnius parma*	5.86	2.56	1.74	2.55	2.55
(68.08%)	*Cyrtodaria siliqua*	4.88	2.92	1.60	2.34	4.89
	*Arctica islandica*	4.49	2.88	1.46	2.14	7.04
	*Clymenura borealis*	6.16	4.86	1.21	1.78	8.82
	*Clymenella zonalis*	4.06	3.91	1.19	1.74	10.56
	*Nephtys caeca*	3.41	2.91	1.17	1.73	12.29
	*Edwardsia elegans*	3.69	3.90	1.12	1.64	13.92
	*Glycera capitata*	2.85	3.42	1.03	1.51	15.43
	*Aglaophamus circinata*	4.11	3.19	1.03	1.51	16.94
	*Ophelia limacina*	2.29	2.89	1.02	1.49	18.43
	Cerianthidae	1.75	3.10	1.01	1.48	19.91
Occurrence	*Chone* sp.	0.47	0.33	0.54	0.92	0.92
(58.56%)	*Cyrtodaria siliqua*	0.40	0.29	0.53	0.90	1.83
	*Scoloplos armiger*	0.28	0.38	0.53	0.90	2.73
	*Polycirrus* sp.	0.47	0.35	0.53	0.90	3.63
	*Ophelia limacina*	0.35	0.44	0.52	0.89	4.52
	*Chaetozone* sp. A	0.37	0.35	0.52	0.88	5.40
	*Euchone papillosa*	0.42	0.37	0.52	0.88	6.28
	*Aricidea catherinae*	0.39	0.37	0.52	0.88	7.16
	Gastropoda	0.24	0.35	0.51	0.88	8.04
	*Hippomedon serratus*	0.30	0.31	0.51	0.88	8.92
	*Periploma leanum*	0.29	0.32	0.51	0.88	9.79
	*Cistenides granulata*	0.37	0.35	0.51	0.88	10.67
	*Nephtys caeca*	0.34	0.30	0.51	0.87	11.54
	*Exogone* sp.	0.39	0.41	0.51	0.87	12.42
	*Arctica islandica*	0.42	0.38	0.51	0.87	13.29
	*Notomastus latericeus*	0.37	0.42	0.51	0.87	14.16
	Mytiloidea	0.28	0.36	0.51	0.87	15.03
	Syllidae	0.47	0.39	0.51	0.87	15.90
	Oligochaeta	0.43	0.39	0.51	0.87	16.76
	*Orbinia swani*	0.38	0.47	0.51	0.86	17.63
	*Cylichna alba*	0.28	0.32	0.50	0.86	18.48
	*Euspira* sp.	0.26	0.30	0.50	0.85	19.34
	*Spio filicornis*	0.53	0.39	0.50	0.85	20.19

Table limited to the taxa contributing to 20% of the dissimilarity between levels of Haddock Abundance for each variable. Abbreviations: %Diss., total dissimilarity between Haddock Abundance levels expressed as a percentage; HA, Haddock Abundance; Aver., average; Av. Diss., average contribution to the total dissimilarity; Cont%, percentage contribution to dissimilarity; Cum%, cumulative percentage contribution of contribution to dissimilarity.

### Environmental Influence on Macrofauna

DISTLM models constructed the best combination of environmental variables that accounted for the variation seen in the data. Sediment Type was the factor explaining the highest variability in all three analyses, with respect to abundance, biomass and presence/absence of macrofauna. The rest of the variables increased the value of Adjusted R^2^ up to 43.8–45.1% of the explained variation (Table 8).

**Table 8:** Distance-based linear model (DistLM) of Bray-Curtis similarities between samples in abundance, biomass and presence/absence of macrofauna against environmental variables.

Variable	Environmental Variable	Adj. *R*^2^	Pseudo-*F*	*P*	Prop.	Cum.
Abundance	Sediment Type	0.194	3.378	0.0001	0.276	0.276
	Mean Bottom Temperature	0.271	12.117	0.0001	0.074	0.351
	Mean Bottom Current	0.303	5.940	0.0001	0.035	0.386
	Mean Bottom Salinity	0.341	6.942	0.0001	0.038	0.425
	Depth	0.350	2.453	0.0008	0.013	0.439
Biomass	Sediment Type	0.200	3.465	0.0001	0.281	0.281
	Depth	0.258	9.261	0.0001	0.058	0.340
	Mean Bottom Current	0.303	7.805	0.0001	0.046	0.386
	Mean Bottom Salinity	0.347	8.085	0.0001	0.044	0.430
	Mean Bottom Temperature	0.352	1.713	0.0240	0.094	0.440
Presence/	Sediment Type	0.200	3.461	0.0001	0.281	0.281
Absence	Depth	0.284	13.465	0.0001	0.081	0.363
	Mean Bottom Current	0.323	7.108	0.0001	0.040	0.403
	Mean Bottom Salinity	0.361	7.071	0.0001	0.038	0.442
	Mean Bottom Temperature	0.365	1.768	0.0310	0.009	0.451

Abbreviations: Adj. R^2^, adjusted R^2^; Prop., proportion of variance explained by each variable; Cum., cumulative proportion of variance explained by multiple variables.

A total of 43.9% of the total variability in macrofaunal abundance was explained by all the variables analyzed; with Sediment Type explaining 27.6% and subsequent variables 16.2%. Sediment Type also explained the highest variability in the biomass and presence/absence data (28.1%), followed by Depth, and Mean Bottom Current, Salinity and Temperature. The variables combined explained 44 and 45.1% of total variation respectively.

**Fig 4:**
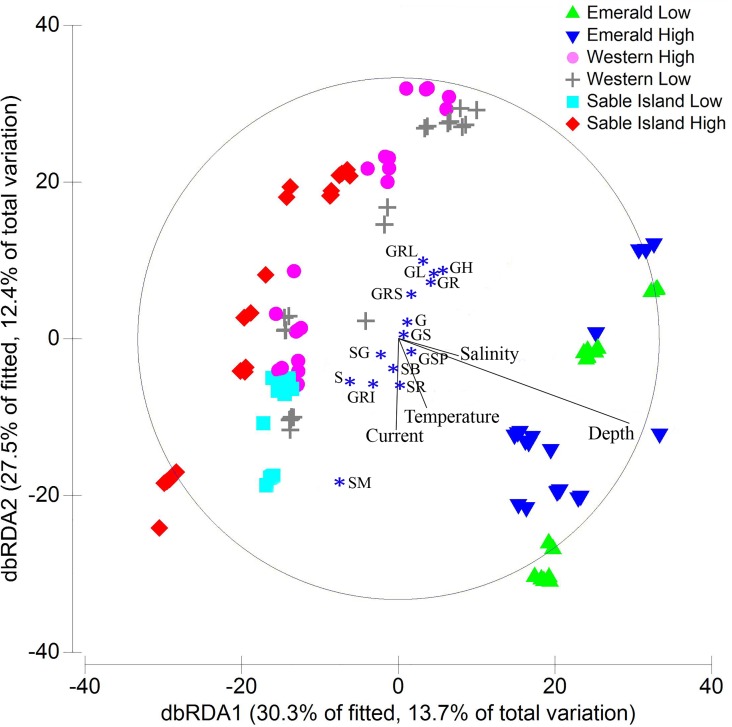
Distance-based RDA ordination of macrofauna based on transformed abundance data as predicted by a DISTLM model. The environmental variables that best explain the variation in infaunal abundance on the three banks are shown. Vectors indicate direction of the effect of quantitative variables in the ordination plot. The variable Sediment Type is illustrated using the end point of the vector (*) for each of the 14 categories.

On the abundance dbRDA plot (Fig 4) the first two axes explained 59.2% of the fitted variation and 25.9% of the total variation. The pattern of the macrofaunal samples on the plot suggested two gradients of variation. The first gradient was driven by the variable Depth, with deepest samples on the lower right quadrant (Emerald Bank) and shallowest on the upper left and explaining 13.6% of the total variation. There was a clear separation between Emerald Bank samples and the rest, while Sable and Western Bank samples were not strongly differentiated. dbRDA ordination of biomass and presence/absence samples showed similar patterns, with samples plotted according to different sediment types and depth. The second gradient was driven by the variable Sediment Type, distinguishing samples of Sand-group bottom types in the lower left quadrant and gravel-types in the upper right and explaining 12.3% of the total variation. Samples of Sable Bank Low Haddock Abundance were displayed forming a tight group in the lower left quadrant, indicating a high proportion of sandy sediments.

## Discussion

### Link between Benthos and Haddock Spatial Structure

Haddock are an ecologically and commercially important fish of the continental shelves of the North Atlantic, with strong links to the benthos [47–50]. Physical parameters such as temperature and depth play a key role in the large-scale spatial structuring of haddock distribution [10], while small-scale spatial structure of haddock populations is linked to life processes such as growth and survival determined in part by predator avoidance, prey availability and protection by camouflage and cover [21].

Our study embedded knowledge of the long-term (32 years), persistent distribution of juvenile haddock abundance in its experimental design, in the form of a factor with two levels, High and Low, characterized by long-term catch rates differing by an order of magnitude [38]. We were interested in determining whether the benthic species composition of the macrofauna could be a factor in determining preferred juvenile habitat for this species. As a factor explaining patterns in benthic macrofaunal communities it could be interpreted in three ways. Significant effects could be due to habitat selection by the juvenile haddock actively choosing an area based on benthic species composition; to habitat modification produced by the fish selectively feeding on the benthos over a long period of time in a given area; and/or to fish and benthos responding separately to the same environmental drivers. Juvenile Haddock Abundance was a statistically significant factor in our multivariate analyses of macrofaunal community composition as assessed using data on abundance, biomass and species occurrence. Therefore one or more of these dynamics between fish and benthos could be operating to induce this effect.

Descriptively the benthic communities at High and Low sites, within Banks, were between 58% and 70% dissimilar. However, the biological relevance of those differences was not conspicuous and samples did not show clear separation in the MDS ordinations. Statistically significant pairwise tests between areas of High and Low juvenile Haddock Abundance were only identified on Sable Island Bank, creating a significant interaction effect that explained a large portion of the variability in the data. The statistical difference in the benthic communities based on abundance, biomass and species occurrence between the sites on Sable Island Bank was clearly explained by differences in the sediment types (abundance: Fig 2 and Fig 4). The Low Haddock Abundance samples were characterized entirely by fine sandy sediment bottoms with large and continuous areas of low bathymetric relief (Fig 2), whereas the High Haddock Abundance samples showed more diverse, rugged and spatially heterogeneous bottoms with both coarse gravel and sand sediments [21]. As a result, the Sable Island Bank benthic communities in the area with Low Haddock Abundance were characterized by infaunal species associated with mud or sand bottoms.

As observed in previous studies, physical habitat can have a strong impact on juvenile haddock spatial distribution due to its essential role in predator avoidance which is the primary source of mortality for demersal fish [21,51]. We note that species such as the sand dollar *Echinarachnius parma*, the bivalve *Arctica islandica*, and the clam *Cyrtodaria siliqua* are found in fine sands [52–54] and their association with the Low Haddock Abundance site on Sable Island Bank may reflect an avoidance of fine sands by the juvenile fish where they would have little camouflage. Abiotic and biotic habitat parameters including sediment composition and presence of emergent fauna or topography, mediate avoidance of predators [51,55–57]. Thus, structurally complex benthic habitats such as those containing coarse sediments, small-scale topographical variability, patched sediment distribution or emergent epibenthic fauna are stated to enhance predator avoidance and therefore increase juvenile fish survivorship [16,51,55,58–60]. These sorts of habitats were more widespread within High Haddock Abundance areas (Fig 2). Haddock are known to be associated with gravel bottoms [10,11,18,23,57,58], including in the study areas [60] and could take advantage of the higher availability of these types of bottoms within preferred areas to camouflage by mimicking the coloration and texture of the uneven seabeds to block visual recognition by predators. Similar behaviors have previously been documented on Georges Bank by Lough et al. [58]. Also, the higher number of crevices among coarser sediments might serve as refuge providing spaces inaccessible to larger-bodied predators [18,51] which has been observed in another demersal fish species, Atlantic cod *Gadus morhua*, by Gotceitas and Brown [61]. They found that in the presence of predators, juvenile cod changed their substrate preference from sand or gravel-pebble to cobble. Finally, these hard substrates support diverse communities of sessile taxa such as Porifera, Hydrozoa or Bivalvia [62,63] that can provide microhabitat biogenic structure and resources [60] that are also used as shelter for juvenile fish [16,64]. Therefore, the extreme difference in sediment type and in the associated communities between preferred and non-preferred sites on Sable Island Bank may indicate that sediment type, rather than the benthic species composition that occupy it, is the key determinant of juvenile haddock abundance in that area, with both fish and benthos responding to it in different ways. Further, as the same trend was not seen on the other banks which also differed in the proportions of sand and gravel between areas of high and low haddock abundance, this relationship is likely non-linear, with some minimal patch size involved in rendering the area non-preferred by the fish. However we were unable to test that hypothesis with these data, not having data on fish abundance at the same fine spatial scale as the benthos. Integrating such data on haddock abundance in future, obtained through video observations, could help determine the relative importance of the relationship between juvenile haddock size and abundance and macrobenthic communities.

It was expected that our results would show a positive association between macrofaunal density and juvenile haddock abundance, as benthic prey density, a subset of the macrofauna, influences the opportunistic diet of haddock [12,13,20,48–50]. The main prey of haddock are small and slow moving benthic infauna [50, 65–67] typically including crustaceans, polychaetes, molluscs, and echinoderms [12, 23,26,50,67,68] ranking in different order according to haddock age [49], prey availability, location and year [48]. On the Scotian Shelf, several studies have analyzed the diet of haddock in the study area [12,13,65,66] although only Kohler and Fitzgerald [66] and Mahon and Neilson [12] focused on juveniles. Both of those studies ranked crustaceans, mainly amphipods and decapods, as the major prey group, followed by echinoderms. In our study we found that at the species level the link between juvenile haddock abundance and benthic prey distribution was not consistent. Some known prey [12,66], such as the amphipod *Unciola irrorata* or the shrimp *Crangon septempsinosa* among others, were more abundant within High Haddock Abundance areas as expected, while others such as the amphipod *Leptocheirus pinguins* or the echinoid *E*. *parma* were more abundant within Low Haddock Abundance areas. Thus, it was not possible to conclude that juvenile haddock were actively selecting the preferred areas according to benthic species composition.

### New Insights into Benthic Macrofaunal Communities on the Eastern Scotian Shelf

Our study is the first to describe the benthic macrofaunal communities over broad as well as smaller spatial scales on the eastern Scotian Shelf. The regional benthic fauna off eastern Canada have previously been described using various sampling gears [32,33,69,70–73] although none of those studies were focused on the eastern Scotian Shelf. Comparable studies to ours were Davis and Gilhen [74], Davis [75], Gilkinson et al. [31], Henry et al. [30], and Kenchington et al. [30], but these were limited to small-spatial scales, latterly collected to examine the impacts of experimental bottom trawling on benthic communities.

Emerald, Western and Sable Island Banks are located in the outer part of the Scotian Shelf and this study has shown them to contain diverse benthic communities. The overall level of community dissimilarity in relative abundance of macrofaunal species between banks was high (Emerald Bank vs Sable Island Bank, 75.8%; Emerald Bank vs. Western Bank 71.4%; Western Bank vs. Sable Island Bank, 65.1%) and was based on differences in a large number of species, and paralleled in biomass and species occurrence. This large scale-variation was expressed as a clear differentiation between Emerald Bank macrofaunal communities and those in Sable Island and Western Banks in the nMDS ordinations, driven primarily by differences in depth, and to a lesser degree by bottom salinity and temperature, as indicated in the dbRDA ordinations. The macrofaunal communities on Sable Island and Western Banks were also distinctive from each other with respect to abundance, biomass and occurrence; however they presented themselves as a continuum rather than as a discontinuity in two dimensional space, as visualized by nMDS, and driven by Sediment Type (Fig 4, Table 8). Overall, Sediment Type was the dominant variable explaining the largest percentage of variation in the macrofaunal community data. Although statistically significant differences were found between the two years of study, there was no temporal change in this dominant among-bank pattern.

Average depth decreases from the southwest (Emerald Bank) to northeast (Sable Island Bank), with the samples from Emerald Bank being at about 80 m, or twice as deep as those from Western Bank at about 43 m and from Sable Island Bank at about 38 m. Bottom temperature and salinity decrease in the same direction, with Emerald Bank being not only deeper, but warmer and saltier. The number of taxa observed and total biomass were both significantly lower on Emerald Bank due in part to the relative paucity of echinoderms and molluscs which were prevalent on Western and Sable Island Banks.

Macrofaunal communities from Western and Sable Island Banks, although significantly different, were more similar to one another than either was to Emerald Bank, which is in concordance with the findings of Courtney et al. [42] who stated that from a geological perspective, Western Bank was a continuum of Sable Island Bank, rather than a separate bank. This distinction of the Emerald Bank macrofauna was not previously known. Seabed topography and substrate type are known to be key structuring factors of benthic assemblages controlling the presence or absence of several sediment-dependent species [62,70,76–80] and they explained the differentiation among the Western and Sable Island Bank samples, as seen in the dbRDA ordination. Emerald Bank seabed showed a lesser variety in sediment types and benthic habitats in comparison to Western Bank seabed that hosted a larger variety of sediments and habitats, which is associated with higher number of organisms and high diversity [36,70,81] especially sessile epifauna [62]. Hence, a more species-rich and abundant community of diverse phyla was found there, which is in concordance with previous studies [29].

This strong statistical signal in the marcobenthos, separating Emerald Bank from the other banks does not correspond with the contemporary abundance of haddock which were generally similar across banks but lower on Sable Island Bank [38].

## Conclusions

We were not able to detect compelling evidence that the temporally-persistent distribution of juvenile haddock density was spatially correlated with differences in benthic macrofaunal communities on the eastern Scotian Shelf. Only on Sable Island Bank were distinct macrofaunal assemblages associated with areas of preferred and non-preferred juvenile haddock habitat. There, both juvenile fish and benthos may be independently responding to the same environmental driver, namely sediment type–the area of non-preferred habitat being 100% sand, while approximately 22% of the habitat in the preferred area was gravel. On the other banks, the sediment types were much more similar in the preferred and non-preferred areas, which lacked statistically significant difference in associated macrofaunal communities. We hypothesize that selection of preferred habitats in the studied banks if present, occurs over fine spatial scales of less than 1 km and may be related to the availability of complex boundaries between gravel and sand areas and their greater topographic relief [82] that allows juvenile haddock to balance predator avoidance on gravel habitats with increased prey abundance/biomass. A future comparative study including stomach contents of juvenile haddock in the area would give insight into the role of benthic prey species as structuring drivers of haddock spatial distribution and abundance [83].

## Supporting Information

S1 Fig Species accumulation curves for each study area and year. The number of observations (Sobs) was used as the estimator and the bars represent standard deviations derived from 999 permutations of the data. A) Curves constructed by Bank (E = Emerald; S = Sable Island; W = Western) and Year (3 = 2003; 5 = 2005); B) Curves constructed with 2005 data only, by Bank and Haddock Abundance Level (High, Low); C) Curves constructed with 2003 data only, by Bank and Haddock Abundance Level (High, Low).
(TIFF)

S2 Fig Mean and standard deviation of Total Number of Taxa (S), Pielou's Evenness Index (J'), Shannon-Wienner Diversity Index (H'), and Total Abundance by Bank, Juvenile Haddock Abundance, and Year. (TIF)

S1 Table Location and associated environmental data for each video-grab sample. (DOCX)

S2 Table Abundance and biomass of macrofaunal taxa by video-grab sample. (DOCX)

S3 Table Summary of ANOVA of transformed Total Biomass (g m^-2^) testing for effect of Year, Bank and Haddock Abundance. (DOCX)
