# Supplementary figures and images for: Influence of Benthic Macrofauna as a Spatial Structuring Agent for Juvenile Haddock (Melanogrammus aeglefinus) on the Eastern Scotian Shelf, Atlantic Canada
Source: PLoS One. 2016 Sep 20;11(9):e0163374. doi: 10.1371/journal.pone.0163374 (PMC5029893; doi:10.1371/journal.pone.0163374)

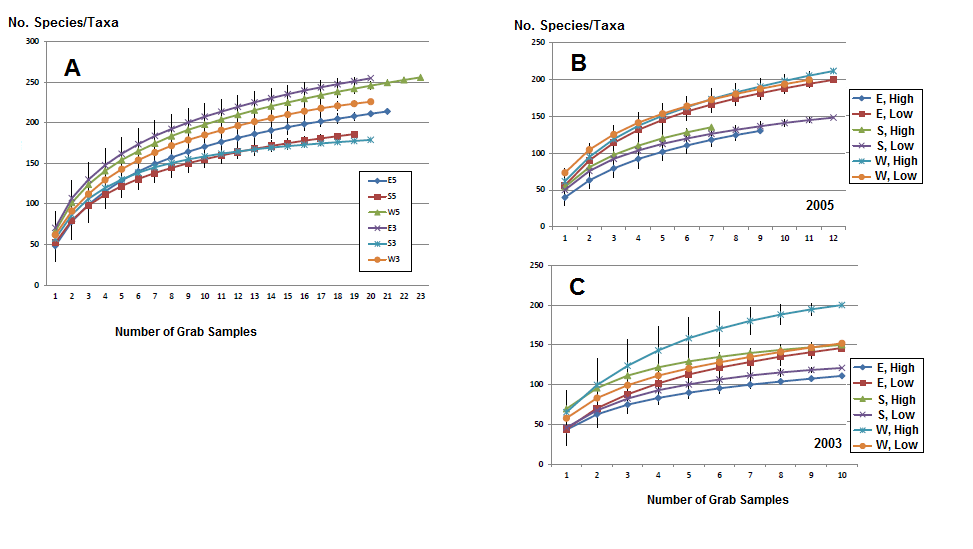

Supplement: S1 Fig — The number of observations (Sobs) was used as the estimator and the bars represent standard deviations derived from 999 permutations of the data. A) Curves constructed by Bank (E = Emerald; S = Sable Island; W = Western) and Year (3 = 2003; 5 = 2005); B) Curves constructed with 2005 data only, by Bank and Haddock Abundance Level (High, Low); C) Curves constructed with 2003 data only, by Bank and Haddock Abundance Level (High, Low). (TIFF) [file pone.0163374.s001.tiff]

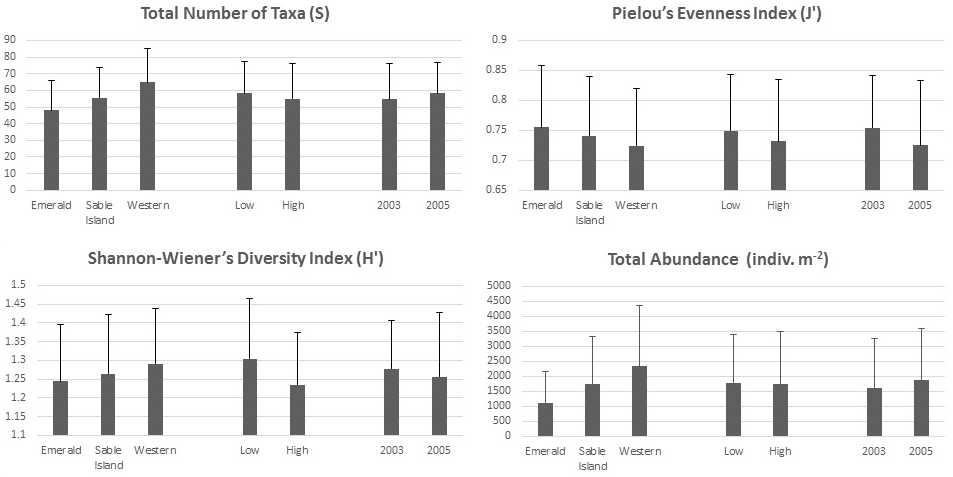

Supplement: S2 Fig — (TIF) [file pone.0163374.s002.tif]
