# Supplementary material for: Influence of Benthic Macrofauna as a Spatial Structuring Agent for Juvenile Haddock (Melanogrammus aeglefinus) on the Eastern Scotian Shelf, Atlantic Canada
Source: PLoS One. 2016 Sep 20;11(9):e0163374. doi: 10.1371/journal.pone.0163374 (PMC5029893; doi:10.1371/journal.pone.0163374)
Supplement: S1 Table — (DOCX) [file pone.0163374.s003.docx]

**S1 Table. Location and associated environmental data for each video-grab sample.**

| **Bank** | **Haddock Abund-ance Level** | **Year** | **Video-grab Code** | **Latitude (Decimal Degrees)** | **Longitude (Decimal Degrees)** | **Depth (m)** | **Sediment Type** | **Mean Bottom Salinity** | **Mean Bottom Temp. (°C)** | **Mean Bottom Current (m s^-1^)** |
| --- | --- | --- | --- | --- | --- | --- | --- | --- | --- | --- |
| Emerald | High | 2003 | VG-02-03 | 43.611730 | -62.428530 | 71.9 | SR | 34.08 | 7.62 | 0.0156 |
|  |  |  | VG-03-03 | 43.604219 | -62.432409 | 73.9 | GRI | 34.08 | 7.73 | 0.0157 |
|  |  |  | VG-04-03 | 43.577640 | -62.440339 | 79.0 | SR | 34.05 | 7.69 | 0.0160 |
|  |  |  | VG-05-03 | 43.571030 | -62.444260 | 87.0 | S | 34.05 | 7.69 | 0.0165 |
|  |  |  | VG-06-03 | 43.586109 | -62.437260 | 78.3 | GSP | 34.08 | 7.73 | 0.0158 |
|  |  |  | VG-07-03 | 43.589060 | -62.435119 | 81.0 | GR | 34.08 | 7.73 | 0.0158 |
|  |  |  | VG-08-03 | 43.600909 | -62.434950 | 74.8 | G | 34.08 | 7.73 | 0.0157 |
|  |  |  | VG-09-03 | 43.596980 | -62.432530 | 75.0 | GR | 34.08 | 7.73 | 0.0157 |
|  |  |  | VG-10-03 | 43.596850 | -62.430590 | 75.9 | S | 34.06 | 7.65 | 0.0157 |
|  |  | 2005 | EH_EVG03 | 43.585295 | -62.439075 | 78.4 | S | 34.08 | 7.73 | 0.0158 |
|  |  |  | EH_EVG04 | 43.575477 | -62.439033 | 80.0 | SR | 34.05 | 7.69 | 0.0160 |
|  |  |  | EH_VG01 | 43.614628 | -62.422449 | 73.0 | SR | 34.08 | 7.62 | 0.0156 |
|  |  |  | EH_VG02 | 43.611843 | -62.422590 | 72.3 | SR | 34.08 | 7.62 | 0.0156 |
|  |  |  | EH_VG03 | 43.605352 | -62.428519 | 73.7 | S | 34.06 | 7.65 | 0.0157 |
|  |  |  | EH_VG04 | 43.602338 | -62.427076 | 75.0 | S | 34.06 | 7.65 | 0.0157 |
|  |  |  | EH_VG05 | 43.605556 | -62.431472 | 73.0 | GRI | 34.06 | 7.65 | 0.0157 |
|  |  |  | EH_VG06 | 43.602040 | -62.432907 | 75.0 | G | 34.08 | 7.73 | 0.0157 |
|  |  |  | EH_VG07 | 43.591521 | -62.436230 | 75.7 | S | 34.08 | 7.73 | 0.0158 |
|  |  |  | EH_VG08 | 43.589832 | -62.439622 | 77.9 | GR | 34.08 | 7.73 | 0.0158 |
|  |  |  | EH_VG09 | 43.588025 | -62.437187 | 77.8 | GR | 34.08 | 7.73 | 0.0158 |
|  |  |  | EH_VG10 | 43.583472 | -62.433850 | 78.4 | S | 34.05 | 7.69 | 0.0160 |
|  | Low | 2003 | VG-11-03 | 43.420960 | -62.396780 | 84.5 | GRI | 34.04 | 7.75 | 0.0171 |
|  |  |  | VG-12-03 | 43.423009 | -62.400829 | 85.2 | SM | 34.03 | 7.70 | 0.0171 |
|  |  |  | VG-13-03 | 43.431680 | -62.386240 | 84.4 | GS | 34.04 | 7.70 | 0.0170 |
|  |  |  | VG-14-03 | 43.434869 | -62.381349 | 83.9 | GRI | 34.04 | 7.70 | 0.0170 |
|  |  |  | VG-15-03 | 43.437359 | -62.386620 | 83.0 | GR | 34.04 | 7.70 | 0.0170 |
|  |  |  | VG-16-03 | 43.436489 | -62.379840 | 83.4 | GS | 34.04 | 7.70 | 0.0170 |
|  |  |  | VG-17-03 | 43.440179 | -62.375259 | 82.1 | SM | 34.04 | 7.70 | 0.0170 |
|  |  |  | VG-18-03 | 43.444749 | -62.377209 | 81.1 | GR | 34.04 | 7.70 | 0.0170 |
|  |  |  | VG-19-03 | 43.444260 | -62.372259 | 80.2 | GS | 34.04 | 7.70 | 0.0170 |
|  |  | 2005 | EC_VG01 | 43.425922 | -62.397812 | 83.2 | SM | 34.03 | 7.70 | 0.0171 |
|  |  |  | EC_VG02 | 43.419401 | -62.402177 | 84.5 | SM | 34.04 | 7.75 | 0.0171 |
|  |  |  | EC_VG03 | 43.436903 | -62.382378 | 83.4 | GR | 34.04 | 7.70 | 0.0170 |
|  |  |  | EC_VG05 | 43.437311 | -62.376930 | 80.4 | GS | 34.04 | 7.70 | 0.0170 |
|  |  |  | EC_VG06 | 43.447172 | -62.374332 | 80.4 | GS | 34.04 | 7.70 | 0.0168 |
|  |  |  | EC_VG07 | 43.449770 | -62.372160 | 79.5 | SM | 34.04 | 7.70 | 0.0168 |
|  |  |  | EC_VG08 | 43.452362 | -62.370677 | 78.9 | GS | 34.04 | 7.70 | 0.0168 |
|  |  |  | EC_VG09 | 43.448428 | -62.364919 | 76.9 | GS | 34.07 | 7.80 | 0.0168 |
| Western | High | 2003 | VG-31-03 | 43.739469 | -61.525480 | 55.4 | GRS | 32.69 | 5.57 | 0.0159 |
|  |  |  | VG-32-03 | 43.739469 | -61.526699 | 55.3 | SM | 32.69 | 5.57 | 0.0159 |
|  |  |  | VG-33-03 | 43.733950 | -61.534160 | 58.6 | GRS | 32.55 | 5.86 | 0.0160 |
|  |  |  | VG-34-03 | 43.730510 | -61.541249 | 57.4 | GRI | 32.55 | 5.86 | 0.0161 |
|  |  |  | VG-35-03 | 43.728969 | -61.551659 | 59.4 | GRL | 32.55 | 5.86 | 0.0163 |
|  |  |  | VG-36-03 | 43.724719 | -61.553590 | 59.4 | SM | 32.55 | 5.86 | 0.0163 |
|  |  |  | VG-37-03 | 43.719679 | -61.560090 | 59.4 | SM | 32.61 | 5.78 | 0.0165 |
|  |  |  | VG-38-03 | 43.719099 | -61.563110 | 60.1 | GRS | 32.61 | 5.78 | 0.0165 |
|  |  |  | VG-38E-03 | 43.716479 | -61.566139 | 59.8 | SM | 32.61 | 5.78 | 0.0165 |
|  |  |  | VG-39-03 | 43.720570 | -61.572299 | 59.0 | GL | 32.61 | 5.78 | 0.0167 |
|  |  | 2005 | WH_EVG01 | 43.736293 | -61.525218 | 55.4 | GRL | 32.69 | 5.57 | 0.0159 |
|  |  |  | WH_EVG02 | 43.732855 | -61.542259 | 58.1 | GRI | 32.55 | 5.86 | 0.0161 |
|  |  |  | WH_VG01 | 43.719822 | -61.569220 | 60.1 | GL | 32.61 | 5.78 | 0.0165 |
|  |  |  | WH_VG02 | 43.727893 | -61.554442 | 58.8 | S | 32.55 | 5.86 | 0.0163 |
|  |  |  | WH_VG03 | 43.729452 | -61.550012 | 60.1 | GRL | 32.55 | 5.86 | 0.0163 |
|  |  |  | WH_VG04 | 43.728475 | -61.547293 | 60.1 | GRS | 32.55 | 5.86 | 0.0163 |
|  |  |  | WH_VG05 | 43.727164 | -61.545147 | 60.1 | GRS | 32.55 | 5.86 | 0.0161 |
|  |  |  | WH_VG06 | 43.731628 | -61.543706 | 59.4 | GRI | 32.55 | 5.86 | 0.0161 |
|  |  |  | WH_VG07 | 43.732958 | -61.536647 | 59.4 | SM | 32.55 | 5.86 | 0.0161 |
|  |  |  | WH_VG08 | 43.735258 | -61.533814 | 58.7 | SG | 32.69 | 5.57 | 0.0159 |
|  |  |  | WH_VG09 | 43.737341 | -61.532133 | 56.7 | SM | 32.69 | 5.57 | 0.0159 |
|  | Low | 2003 | VG-40-03 | 43.881950 | -61.581299 | 56.4 | SB | 33.32 | 5.35 | 0.0156 |
|  |  |  | VG-41-03 | 43.883690 | -61.578020 | 54.8 | GH | 33.32 | 5.35 | 0.0151 |
|  |  |  | VG-42-03 | 43.886409 | -61.574809 | 55.0 | GL | 33.32 | 5.35 | 0.0151 |
|  |  |  | VG-43-03 | 43.887569 | -61.573590 | 54.9 | GRL | 33.32 | 5.35 | 0.0151 |
|  |  |  | VG-44-03 | 43.885680 | -61.568629 | 54.2 | GRS | 33.32 | 5.35 | 0.0155 |
|  |  |  | VG-45-03 | 43.887960 | -61.567189 | 52.1 | SM | 33.32 | 5.35 | 0.0155 |
|  |  |  | VG-46-03 | 43.894430 | -61.565040 | 51.1 | S | 33.32 | 5.35 | 0.0155 |
|  |  |  | VG-47-03 | 43.897579 | -61.555590 | 50.5 | SM | 33.38 | 5.35 | 0.0150 |
|  |  |  | VG-48-03 | 43.899769 | -61.553040 | 51.0 | SM | 33.38 | 5.35 | 0.0150 |
|  |  |  | VG-49-03 | 43.909190 | -61.544680 | 49.6 | S | 33.38 | 5.35 | 0.0149 |
|  |  | 2005 | WC_EVG01 | 43.894627 | -61.561532 | 51.39 | S | 33.32 | 5.35 | 0.0155 |
|  |  |  | WC_EVG02 | 43.886640 | -61.570577 | 54.1 | GRS | 33.32 | 5.35 | 0.0155 |
|  |  |  | WC_VG01 | 43.877724 | -61.588932 | 58.7 | GH | 33.36 | 5.42 | 0.0152 |
|  |  |  | WC_VG02 | 43.879466 | -61.581887 | 56.7 | GH | 33.32 | 5.35 | 0.0156 |
|  |  |  | WC_VG03 | 43.881852 | -61.579590 | 55.39 | GH | 33.32 | 5.35 | 0.0156 |
|  |  |  | WC_VG04 | 43.884036 | -61.572937 | 56.0 | GL | 33.32 | 5.35 | 0.0151 |
|  |  |  | WC_VG05 | 43.888733 | -61.575167 | 56.0 | GRL | 33.32 | 5.35 | 0.0151 |
|  |  |  | WC_VG07 | 43.896853 | -61.552109 | 52.0 | SM | 33.38 | 5.35 | 0.0150 |
|  |  |  | WC_VG08 | 43.900337 | -61.554115 | 52.0 | SM | 33.38 | 5.35 | 0.0150 |
|  |  |  | WC_VG09 | 43.904102 | -61.544575 | 51.29 | SM | 33.38 | 5.35 | 0.0150 |
|  |  |  | WC_VG10 | 43.908301 | -61.545048 | 51.29 | S | 33.38 | 5.35 | 0.0150 |
| Sable Island | High | 2003 | VG-21-03 | 44.694700 | -60.911769 | 45.1 | SM | 32.42 | 4.46 | 0.0190 |
|  |  |  | VG-22-03 | 44.733499 | -60.909590 | 46.7 | GL | 32.42 | 4.46 | 0.0193 |
|  |  |  | VG-23-03 | 44.693199 | -60.908310 | 47.0 | SB | 32.42 | 4.46 | 0.0191 |
|  |  |  | VG-24-03 | 44.717700 | -60.903889 | 49.0 | GH | 32.42 | 4.46 | 0.0193 |
|  |  |  | VG-25-03 | 44.710199 | -60.900350 | 44.2 | GR | 32.42 | 4.46 | 0.0193 |
|  |  |  | VG-26-03 | 44.668099 | -60.902099 | 48.0 | GH | 32.42 | 4.46 | 0.0191 |
|  |  |  | VG-27-03 | 44.677600 | -60.887960 | 42.5 | SM | 32.38 | 4.45 | 0.0192 |
|  |  |  | VG-28-03 | 44.652800 | -60.887169 | 43.3 | SM | 32.38 | 4.45 | 0.0192 |
|  |  |  | VG-29-03 | 44.631399 | -60.874980 | 40.0 | GRS | 32.38 | 4.45 | 0.0198 |
|  |  |  | VG-30-03 | 44.632999 | -60.865340 | 36.8 | SM | 32.36 | 4.57 | 0.0200 |
|  |  | 2005 | SH_EVG01 | 44.064987 | -60.900803 | 45.9 | GR | 32.42 | 4.46 | 0.0191 |
|  |  |  | SH_EVG02 | 44.067931 | -60.905490 | 45.6 | GH | 32.42 | 4.46 | 0.0191 |
|  |  |  | SH_VG01 | 44.073973 | -60.911295 | 46.9 | GL | 32.42 | 4.46 | 0.0192 |
|  |  |  | SH_VG02 | 44.071672 | -60.913854 | 46.6 | SB | 32.42 | 4.46 | 0.0192 |
|  |  |  | SH_VG03 | 44.072470 | -60.909787 | 46.3 | GL | 32.42 | 4.46 | 0.0193 |
|  |  |  | SH_VG04 | 44.068930 | -60.904888 | 44.9 | GH | 32.42 | 4.46 | 0.0191 |
|  |  |  | SH_VG05 | 44.068432 | -60.901037 | 45.2 | GRS | 32.42 | 4.46 | 0.0191 |
|  |  |  | SH_VG06 | 44.066645 | -60.891685 | 41.2 | SM | 32.38 | 4.45 | 0.0192 |
|  |  |  | SH_VG07 | 44.064126 | -60.888727 | 42.2 | SM | 32.38 | 4.45 | 0.0192 |
|  |  |  | SH_VG08 | 44.062553 | -60.874884 | 37.4 | GRS | 32.38 | 4.45 | 0.0198 |
|  |  |  | SH_VG09 | 44.066352 | -60.907620 | 44.9 | SB | 32.42 | 4.46 | 0.0191 |
|  |  |  | SH_VG10 | 44.065421 | -60.901155 | 45.6 | GR | 32.42 | 4.46 | 0.0191 |
|  | Low | 2003 | VG-50-03 | 43.777389 | -60.527470 | 52.0 | SM | 32.36 | 5.83 | 0.0189 |
|  |  |  | VG-51-03 | 43.775460 | -60.523380 | 53.2 | SM | 32.40 | 5.68 | 0.0191 |
|  |  |  | VG-52-03 | 43.769810 | -60.523240 | 56.5 | S | 32.47 | 5.77 | 0.0190 |
|  |  |  | VG-53-03 | 43.766199 | -60.515450 | 59.4 | S | 32.47 | 5.77 | 0.0190 |
|  |  |  | VG-54-03 | 43.765320 | -60.511240 | 59.5 | S | 32.47 | 5.77 | 0.0190 |
|  |  |  | VG-55-03 | 43.761069 | -60.504350 | 58.3 | S | 32.47 | 5.77 | 0.0190 |
|  |  |  | VG-56-03 | 43.759399 | -60.496400 | 55.9 | S | 32.50 | 5.83 | 0.0191 |
|  |  |  | VG-57-03 | 43.755150 | -60.485390 | 54.5 | S | 32.50 | 5.83 | 0.0189 |
|  |  |  | VG-58-03 | 43.765380 | -60.499090 | 58.9 | S | 32.50 | 5.83 | 0.0191 |
|  |  |  | VG-59-03 | 43.758609 | -60.481610 | 53.3 | S | 32.50 | 5.83 | 0.0189 |
|  |  | 2005 | SC_VG02 | 43.768540 | -60.509467 | 59.7 | S | 32.47 | 5.77 | 0.0190 |
|  |  |  | SC_VG03 | 43.772193 | -60.519022 | 56.7 | S | 32.40 | 5.68 | 0.0191 |
|  |  |  | SC_VG04 | 43.775302 | -60.525370 | 52.6 | SM | 32.36 | 5.83 | 0.0189 |
|  |  |  | SC_VG05 | 43.772767 | -60.529446 | 52.6 | SM | 32.36 | 5.83 | 0.0189 |
|  |  |  | SC_VG06 | 43.771484 | -60.528658 | 52.6 | SG | 32.36 | 5.83 | 0.0189 |
|  |  |  | SC_VG07 | 43.772097 | -60.527096 | 53.3 | SM | 32.36 | 5.83 | 0.0189 |

Sediment Type Abbreviations: S, sand; SM, Sand with Megaripples; SG, Sand to Sandy Gravel; SB, Sand with Scattered Boulders; SR, Sand Ribbons; G, Gravel; GS, Gravel to Gravelly Sand; GR, Gravel Ripples; GRS, Gravel Ripples, Short Wave Length; GRL, Gravel Ripples, Long Wave Length; GRI, Gravel Ripples, Incised; GL, Gravel Lag; GH, Gravel, Hummocky; GSP, Gravel with Small Sand Patches.
