# Supplementary material for: Influence of Benthic Macrofauna as a Spatial Structuring Agent for Juvenile Haddock (Melanogrammus aeglefinus) on the Eastern Scotian Shelf, Atlantic Canada
Source: PLoS One. 2016 Sep 20;11(9):e0163374. doi: 10.1371/journal.pone.0163374 (PMC5029893; doi:10.1371/journal.pone.0163374)
Supplement: S2 Table — (DOCX) [file pone.0163374.s004.docx]

**S2 Table. Abundance and biomass of macrofaunal taxa by video-grab sample.**

| **Video-grab Code** | **Taxon** | **Abundance**  **(indiv m^-2^)** | **Biomass**  **(g m^-2^)** |
| --- | --- | --- | --- |
| ec_vg01 | *Aglaophamus circinata* | 20 | 0.921 |
|  | *Ampharete finmarchica* | 6 | 0.0206 |
|  | *Aricidea* sp. B | 2 | 0.0004 |
|  | Ascidiacea (solitary) | 16 | 0.4132 |
|  | *Axionice maculata* | 22 | 0.1536 |
|  | *Chaetozone* sp. | 4 | 0.0016 |
|  | *Chone* sp. | 122 | 0.1406 |
|  | *Clymenura borealis* | 124 | 23.576 |
|  | *Colus* sp. | 14 | 0.0362 |
|  | *Dyopedos* sp. | 4 | 0.002 |
|  | *Edwardsia sulcata* | 4 | 0.047 |
|  | *Ericthonius rubricornis* | 58 | 0.049 |
|  | *Glycera capitata* | 6 | 0.4848 |
|  | *Golfingia* sp. | 4 | 0.0276 |
|  | *Goniada maculata* | 4 | 0.1272 |
|  | *Lumbrinerides acuta* | 12 | 0.2754 |
|  | *Macoma calcarea* | 10 | 0.0054 |
|  | Nemertea | 6 | 0.5484 |
|  | *Nereis* sp. | 2 | 0.18 |
|  | *Nicomache lumbricalis* | 4 | 0.6414 |
|  | *Notomastus latericeus* | 18 | 1.1924 |
|  | *Ophelina acuminata* | 4 | 0.081 |
|  | *Ophiura* sp. juvenile | 2 | 0.0008 |
|  | *Owenia fusiformis* | 32 | 0.043 |
|  | *Parvicardium pinnulatum* | 14 | 0.5228 |
|  | *Polycirrus* sp. | 12 | 0.0208 |
|  | *Scalibregma inflatum* | 4 | 0.7464 |
|  | *Scolelepis squamata* | 4 | 0.0302 |
|  | *Scoloplos armiger* | 4 | 0.0758 |
|  | *Spiochaetopterus typicus* | 4 | 0.0874 |
|  | *Strongylocentrotus* sp. | 2 | 0.017 |
|  | Syllidae | 28 | 0.0018 |
|  | *Tharyx* sp. | 4 | 0.011 |
|  | *Travisia* sp. | 2 | 0.3844 |
|  | *Unciola irrorata* | 276 | 1.387 |
| ec_vg02 | *Aglaophamus circinata* | 8 | 0.129 |
|  | *Ampelisca macrocephala* | 2 | 0.032 |
|  | *Arctica islandica* | 12 | 71.8486 |
|  | *Argissa hamatipes* | 4 | 0.004 |
|  | Ascidiacea (solitary) | 14 | 0.416 |
|  | *Astarte* sp. | 2 | 0.0064 |
|  | *Axionice maculata* | 20 | 0.0614 |
|  | *Boreocingula* sp. | 4 | 0.0094 |
|  | *Chiridotea tuftsii* | 8 | 0.0324 |
|  | *Chone* sp. | 6 | 0.0014 |
|  | *Cirolana polita* | 2 | 0.027 |
|  | *Clymenura borealis* | 42 | 8.7426 |
|  | *Colus* sp. | 2 | 0.0062 |
|  | *Cylichna alba* | 6 | 0.0156 |
|  | *Dyopedos* sp. | 26 | 0.0222 |
|  | *Ericthonius fasciatus* | 2 | 0.0002 |
|  | *Glycera capitata* | 2 | 0.0014 |
|  | *Goniada maculata* | 8 | 0.1418 |
|  | *Hippomedon serratus* | 4 | 0.1312 |
|  | *Leptocheirus pinguis* | 2 | 0.005 |
|  | *Levensinea gracilis* | 2 | 0.001 |
|  | *Lumbrinerides acuta* | 4 | 0.1134 |
|  | Nemertea | 4 | 0.0064 |
|  | *Nephtys discors* | 2 | 1.4298 |
|  | *Nereis* sp. | 2 | 0.1112 |
|  | *Notomastus latericeus* | 6 | 0.5332 |
|  | *Ophiura sarsi* | 78 | 0.0436 |
|  | *Orbinia swani* | 2 | 0.1096 |
|  | *Owenia fusiformis* | 12 | 0.0212 |
|  | *Parvicardium pinnulatum* | 4 | 0.0548 |
|  | *Periploma leanum* | 4 | 0.6426 |
|  | *Psammonyx* sp. | 14 | 0.3174 |
|  | *Scalibregma inflatum* | 2 | 0.1394 |
|  | *Scolelepis squamata* | 8 | 0.0276 |
|  | *Solariella obscura* | 2 | 0.0824 |
|  | *Spiochaetopterus typicus* | 2 | 0.0892 |
|  | *Travisia* sp. | 12 | 0.1264 |
|  | *Unciola irrorata* | 24 | 0.1406 |
| ec_vg03 | *Aglaophamus circinata* | 20 | 0.12558 |
|  | *Ampharete finmarchica* | 4 | 0.00178 |
|  | *Anonyx sarsi* | 2 | 0.00124 |
|  | *Arctica islandica* | 2 | 0.1012 |
|  | *Aricidea* sp. B | 10 | 0.0001 |
|  | Ascidiacea (solitary) | 6 | 0.07766 |
|  | *Axionice maculata* | 14 | 0.00616 |
|  | *Chaetozone* sp. | 20 | 0.00222 |
|  | *Chiridotea tuftsii* | 2 | 0.00148 |
|  | *Chone* sp. | 138 | 0.00554 |
|  | *Cirolana polita* | 10 | 1.00388 |
|  | *Clymenura borealis* | 44 | 1.24764 |
|  | *Colus* sp. | 10 | 0.00086 |
|  | *Cylichna alba* | 6 | 0.0106 |
|  | *Diastylis quadrispinosa* | 4 | 0.0104 |
|  | *Ericthonius fasciatus* | 338 | 0.093 |
|  | *Ericthonius rubricornis* | 150 | 0.2127 |
|  | *Euclymene zonalis* | 20 | 0.0049 |
|  | Gastropoda | 4 | 0.0124 |
|  | *Glycera capitata* | 12 | 0.02298 |
|  | *Lumbrinerides acuta* | 12 | 0.01106 |
|  | *Macoma calcarea* | 8 | 0.00042 |
|  | *Melita dentata* | 36 | 0.135 |
|  | *Ophiopholis aculeata* | 2 | 0.0298 |
|  | *Ophiura robusta* | 8 | 0.00762 |
|  | *Ophiura* sp. juvenile | 8 | 0.1864 |
|  | *Parvicardium pinnulatum* | 16 | 0.03466 |
|  | *Periploma leanum* | 4 | 0.29642 |
|  | *Phoxocephalus holbolli* | 6 | 0.007 |
|  | *Placopecten magellanicus* | 2 | 2.3168 |
|  | Polygordiidae | 12 | 0.00006 |
|  | *Protodorvillea kefersteini* | 6 | 0.00024 |
|  | *Scalibregma inflatum* | 6 | 0.01316 |
|  | *Scolelepis squamata* | 2 | 0.0019 |
|  | *Spiochaetopterus typicus* | 2 | 0.00482 |
|  | *Spiophanes bombyx* | 8 | 0.00474 |
|  | *Strongylocentrotus* sp. | 2 | 0.04 |
|  | Syllidae | 40 | 0.00052 |
|  | *Syrrhoe crenulata* | 20 | 0.0818 |
|  | *Tiron spiniferus* | 14 | 0.0212 |
|  | *Unciola irrorata* | 1544 | 3.25574 |
| ec_vg05 | *Aglaophamus circinata* | 6 | 1.0364 |
|  | *Ampharete finmarchica* | 20 | 0.0434 |
|  | *Anonyx sarsi* | 10 | 0.8198 |
|  | *Aricidea* sp. | 2 | 0.0028 |
|  | *Aricidea* sp. A | 14 | 0.0154 |
|  | *Asabellides* sp. | 2 | 0.0066 |
|  | *Astarte* sp. | 24 | 0.0156 |
|  | *Axionice maculata* | 12 | 0.037 |
|  | *Chaetozone setosa* | 4 | 0.0242 |
|  | *Chaetozone* sp. | 14 | 0.1032 |
|  | *Chlamys islandica* | 2 | 0.0184 |
|  | *Chone* sp. | 816 | 1.6258 |
|  | *Clymenura borealis* | 44 | 14.8222 |
|  | *Cyclocardia* sp. A | 2 | 0.1792 |
|  | *Cylichna alba* | 2 | 0.045 |
|  | *Diastylis quadrispinosa* | 6 | 0.042 |
|  | *Drilonereis magna* | 6 | 0.1286 |
|  | *Ericthonius rubricornis* | 384 | 0.3748 |
|  | *Euclymene zonalis* | 132 | 1.2526 |
|  | *Glycera capitata* | 68 | 1.3948 |
|  | *Harpinia propinqua* | 2 | 0.0042 |
|  | *Laonice cirrata* | 36 | 0.0376 |
|  | *Lumbrinerides acuta* | 66 | 0.8916 |
|  | *Melita dentata* | 4 | 0.0048 |
|  | Mytiloidea | 2 | 0.001 |
|  | *Nereis* sp. | 2 | 0.004 |
|  | *Nothria conchylega* | 4 | 0.1592 |
|  | *Notomastus latericeus* | 4 | 0.053 |
|  | *Ophelina acuminata* | 2 | 0.033 |
|  | *Paraonis* sp. | 2 | 0.001 |
|  | *Parvicardium pinnulatum* | 12 | 0.0396 |
|  | *Polycirrus* sp. | 2 | 0.0054 |
|  | *Polydora caulleryi* | 8 | 0.008 |
|  | Polygordiidae | 78 | 0.0196 |
|  | *Praxillella praetermissa* | 30 | 0.288 |
|  | *Protodorvillea kefersteini* | 4 | 0.001 |
|  | *Scalibregma inflatum* | 4 | 0.1336 |
|  | *Scolelepis squamata* | 4 | 0.007 |
|  | *Solariella obscura* | 2 | 0.1392 |
|  | Syllidae | 18 | 0.0036 |
|  | *Syrrhoe crenulata* | 6 | 0.0256 |
|  | *Tharyx* sp. | 8 | 0.0152 |
|  | *Tiron spiniferus* | 4 | 0.0086 |
|  | *Unciola irrorata* | 1384 | 5.2894 |
| ec_vg06 | *Aglaophamus circinata* | 12 | 1.223 |
|  | *Ampharete finmarchica* | 8 | 0.1176 |
|  | *Anonyx sarsi* | 2 | 0.0042 |
|  | *Arctica islandica* | 8 | 0.0042 |
|  | *Argissa hamatipes* | 2 | 0.0022 |
|  | *Aricidea catherinae* | 52 | 0.0878 |
|  | *Aricidea* sp. | 40 | 0.047 |
|  | Ascidiacea (solitary) | 4 | 0.1818 |
|  | *Axionice maculata* | 10 | 0.0906 |
|  | *Chaetozone* sp. | 44 | 0.2652 |
|  | *Chone* sp. | 178 | 0.5704 |
|  | *Cirolana polita* | 8 | 0.2348 |
|  | *Clymenura borealis* | 66 | 12.3308 |
|  | *Cyclocardia borealis* | 4 | 0.0002 |
|  | *Cyrtodaria siliqua* | 6 | 0.0064 |
|  | *Dyopedos* sp. | 2 | 0.0016 |
|  | *Ericthonius fasciatus* | 118 | 0.133 |
|  | *Ericthonius rubricornis* | 56 | 0.0862 |
|  | *Euclymene zonalis* | 68 | 0.5886 |
|  | *Exogone* sp. | 122 | 0.0198 |
|  | Gastropoda | 4 | 0.0942 |
|  | *Glycera capitata* | 96 | 0.8056 |
|  | *Harmothoe extenuata* | 2 | 0.0034 |
|  | *Hippomedon serratus* | 4 | 0.0614 |
|  | *Lumbrinerides acuta* | 88 | 1.5296 |
|  | Lysianassidae | 2 | 0.0012 |
|  | *Melphidippa spinosa* | 2 | 0.001 |
|  | Mytiloidea | 2 | 0.0006 |
|  | Nemertea | 6 | 0.012 |
|  | Nephtyidae sp. juvenile | 20 | 0.0536 |
|  | *Nereis* sp. | 2 | 0.1328 |
|  | *Nothria conchylega* | 2 | 0.0066 |
|  | *Notomastus latericeus* | 12 | 0.3536 |
|  | Oligochaeta | 22 | 0.131 |
|  | *Ophiura* sp. juvenile | 4 | 0.0008 |
|  | *Orbinia swani* | 2 | 0.3116 |
|  | *Paraonis* sp. | 28 | 0.0634 |
|  | *Periploma leanum* | 44 | 0.0152 |
|  | *Polycirrus* sp. | 2 | 0.0022 |
|  | *Polydora caulleryi* | 10 | 0.0066 |
|  | Polygordiidae | 208 | 0.1446 |
|  | *Protodorvillea kefersteini* | 2 | 0.0002 |
|  | Questidae | 2 | 0.0002 |
|  | *Solariella obscura* | 2 | 0.1006 |
|  | *Spio filicornis* | 12 | 0.0256 |
|  | Syllidae | 116 | 0.012 |
|  | *Syrrhoe crenulata* | 6 | 0.012 |
|  | *Tharyx* sp. | 4 | 0.0012 |
|  | *Tiron spiniferus* | 2 | 0.0016 |
|  | *Unciola irrorata* | 690 | 2.7774 |
| ec_vg07 | *Aglaophamus circinata* | 24 | 0.8584 |
|  | *Ampelisca macrocephala* | 2 | 0.0368 |
|  | *Ampharete finmarchica* | 6 | 0.0072 |
|  | *Aricidea catherinae* | 6 | 0.0028 |
|  | *Aricidea* sp. B | 8 | 0.0048 |
|  | *Capitella capitata* | 4 | 0.0092 |
|  | *Chaetozone* sp. | 10 | 0.0476 |
|  | *Chiridotea tuftsii* | 2 | 0.0058 |
|  | *Chone* sp. | 34 | 0.0138 |
|  | *Cirolana polita* | 4 | 0.1602 |
|  | *Cistenides granulata* | 6 | 0.0074 |
|  | *Clymenura borealis* | 48 | 10.2928 |
|  | *Crangon septemspinosa* | 2 | 0.1322 |
|  | *Dyopedos* sp. | 8 | 0.0044 |
|  | *Echinarachnius parma* | 2 | 0.0202 |
|  | *Edotea triloba* | 2 | 0.0132 |
|  | *Ericthonius rubricornis* | 18 | 0.0142 |
|  | *Euspira* sp. | 2 | 0.119 |
|  | Gastropoda | 10 | 0.038 |
|  | *Glycera capitata* | 22 | 0.146 |
|  | *Hippomedon serratus* | 10 | 0.298 |
|  | *Lumbrinerides acuta* | 34 | 0.3006 |
|  | *Macoma calcarea* | 10 | 5.8136 |
|  | *Monoculodes* sp. | 2 | 0.0054 |
|  | *Nephtys bucera* | 2 | 0.2256 |
|  | *Ophelia limacina* | 6 | 1.349 |
|  | *Orchomenella minuta* | 2 | 0.0106 |
|  | *Paraonis* sp. | 4 | 0.0022 |
|  | *Polycirrus* sp. | 2 | 0.0022 |
|  | Polygordiidae | 12 | 0.0018 |
|  | *Psammonyx* sp. | 2 | 0.0766 |
|  | *Scalibregma inflatum* | 6 | 0.1648 |
|  | *Scolelepis squamata* | 8 | 0.0392 |
|  | Spionidae | 12 | 0.0044 |
|  | Syllidae | 74 | 0.0076 |
|  | *Syrrhoe crenulata* | 8 | 0.0268 |
|  | *Tharyx* sp. | 2 | 0.005 |
|  | *Unciola irrorata* | 48 | 0.2336 |
| ec_vg08 | *Aglaophamus circinata* | 12 | 0.0966 |
|  | *Ampharete finmarchica* | 10 | 0.1888 |
|  | *Anonyx sarsi* | 4 | 0.1422 |
|  | *Arcteobia anticostiensis* | 4 | 0.026 |
|  | *Aricidea catherinae* | 6 | 0.0022 |
|  | *Aricidea* sp. | 20 | 0.0064 |
|  | *Aricidea wassi* | 4 | 0.0138 |
|  | Ascidiacea (solitary) | 4 | 0.1774 |
|  | *Chaetozone setosa* | 2 | 0.0052 |
|  | *Chaetozone* sp. | 16 | 0.0418 |
|  | *Chiridotea tuftsii* | 4 | 0.0046 |
|  | *Chone* sp. | 10 | 0.0122 |
|  | *Cirolana polita* | 28 | 0.9626 |
|  | *Clymenura borealis* | 184 | 35.2254 |
|  | *Cylichna alba* | 4 | 0.0002 |
|  | *Dyopedos* sp. | 14 | 0.003 |
|  | *Edotea montosa* | 2 | 0.0034 |
|  | *Edotea triloba* | 2 | 0.002 |
|  | *Ericthonius fasciatus* | 20 | 0.0176 |
|  | *Ericthonius rubricornis* | 20 | 0.0146 |
|  | *Euclymene zonalis* | 2 | 0.0192 |
|  | *Exogone* sp. | 92 | 0.0178 |
|  | Gastropoda | 42 | 0.0276 |
|  | *Glycera capitata* | 16 | 0.044 |
|  | *Hippomedon serratus* | 28 | 0.144 |
|  | *Levensinea gracilis* | 6 | 0.0038 |
|  | *Lumbrinerides acuta* | 30 | 0.3864 |
|  | Lysianassidae | 64 | 0.5644 |
|  | *Macoma calcarea* | 4 | 2.775 |
|  | Nemertea | 2 | 0.0094 |
|  | Nephtyidae sp. juvenile | 24 | 0.0298 |
|  | *Notomastus latericeus* | 2 | 0.0046 |
|  | Oligochaeta | 4 | 0.0086 |
|  | *Ophiura* sp. juvenile | 16 | 0.005 |
|  | *Orbinia swani* | 2 | 0.039 |
|  | *Owenia fusiformis* | 2 | 0.0006 |
|  | *Paraonis* sp. | 6 | 0.0054 |
|  | *Periploma leanum* | 20 | 2.995 |
|  | *Polycirrus* sp. | 8 | 0.0196 |
|  | Polygordiidae | 6 | 0.0014 |
|  | *Praxillella praetermissa* | 4 | 0.0158 |
|  | *Protodorvillea kefersteini* | 4 | 0.0004 |
|  | *Psammonyx* sp. | 52 | 0.4374 |
|  | *Scalibregma inflatum* | 4 | 0.1412 |
|  | *Scolelepis squamata* | 6 | 0.1362 |
|  | *Solariella obscura* | 6 | 0.056 |
|  | *Spiophanes bombyx* | 10 | 0.0988 |
|  | Syllidae | 26 | 0.0028 |
|  | *Tharyx* sp. | 28 | 0.4468 |
|  | *Travisia* sp. | 8 | 0.4696 |
|  | *Unciola irrorata* | 32 | 0.0668 |
| ec_vg09 | *Acirsa* sp. | 4 | 0.0042 |
|  | Actiniaria (non burrowing) | 112 | 0.0596 |
|  | *Aglaophamus circinata* | 6 | 0.0718 |
|  | *Ampharete finmarchica* | 8 | 0.0266 |
|  | *Amphitrite affinis* | 2 | 0.0692 |
|  | *Anomia* sp. | 4 | 0.09 |
|  | *Aricidea* sp. A | 6 | 0.004 |
|  | *Asabellides* sp. | 36 | 0.0512 |
|  | Ascidiacea (solitary) | 6 | 0.1134 |
|  | *Astarte* sp. | 24 | 6.6806 |
|  | *Axionice maculata* | 2 | 0.0146 |
|  | *Axius serratus* | 2 | 0.1292 |
|  | *Bathyarca pectunculoides* | 6 | 0.0992 |
|  | *Caprella* sp. | 20 | 0.0742 |
|  | *Chone* sp. | 116 | 0.229 |
|  | *Cistenides granulata* | 18 | 0.0518 |
|  | *Drilonereis magna* | 2 | 0.1636 |
|  | *Edwardsia elegans* | 2 | 0.0014 |
|  | *Eunice pennata* | 10 | 0.0216 |
|  | *Glycera capitata* | 4 | 0.107 |
|  | *Golfingia* sp. | 32 | 0.03 |
|  | *Goniada maculata* | 6 | 0.1658 |
|  | *Harmothoe extenuata* | 2 | 0.0072 |
|  | *Henricia* sp. | 2 | 0.1062 |
|  | *Leptocheirus pinguis* | 2 | 0.0264 |
|  | *Lithodes maja* | 2 | 0.087 |
|  | *Lumbrineris fragilis* | 2 | 0.1208 |
|  | *Mediomastus ambiseta* | 4 | 0.0143 |
|  | *Melina* sp. | 2 | 0.0074 |
|  | *Melita dentata* | 2 | 0.0012 |
|  | *Modiolus modiolus* | 4 | 0.4716 |
|  | Mytiloidea | 12 | 0.0188 |
|  | Nemertea | 4 | 0.1282 |
|  | *Nereis* sp. | 4 | 0.0422 |
|  | *Nothria conchylega* | 6 | 0.0466 |
|  | *Ophelina acuminata* | 8 | 0.2516 |
|  | *Ophiopholis aculeata* | 2 | 0.1922 |
|  | *Ophiura robusta* | 6 | 0.0034 |
|  | *Parvicardium pinnulatum* | 4 | 0.0388 |
|  | *Phascolion strombus* | 2 | 0.016 |
|  | *Photis* sp. | 142 | 0.055 |
|  | *Phyllodoce groenlandica* | 2 | 0.1204 |
|  | Platyhelminthes | 2 | 0.0042 |
|  | *Polycirrus* sp. | 12 | 0.0468 |
|  | Polygordiidae | 2 | 0.0008 |
|  | *Prionospio cirrifera* | 8 | 0.021 |
|  | *Puncturella noachina* | 58 | 0.2458 |
|  | *Scalibregma inflatum* | 2 | 0.0096 |
|  | *Strongylocentrotus* sp. | 2 | 0.022 |
|  | Syllidae | 32 | 0.0018 |
|  | *Terebellides stroemi* | 4 | 0.012 |
|  | *Terebratulina septentrionalis* | 4 | 0.6138 |
|  | *Tharyx* sp. | 20 | 0.3192 |
|  | *Tonicella rubra* | 2 | 0.018 |
|  | *Unciola irrorata* | 18 | 0.0936 |
| eh_evg03 | *Aglaophamus circinata* | 60 | 1.86022 |
|  | *Ampharete finmarchica* | 4 | 0.0041 |
|  | *Anomia* sp. | 2 | 0.0128 |
|  | *Arcteobia anticostiensis* | 2 | 0.0454 |
|  | *Arctica islandica* | 12 | 0.0318 |
|  | *Argissa hamatipes* | 2 | 0.0004 |
|  | *Aricidea catherinae* | 2 | 0.0022 |
|  | *Aricidea* sp. A | 2 | 0.0018 |
|  | Ascidiacea (solitary) | 146 | 1.08388 |
|  | *Astarte* sp. | 56 | 0.53324 |
|  | *Axionice maculata* | 4 | 0.0396 |
|  | *Caprella* sp. | 2 | 0.00378 |
|  | *Chaetozone* sp. | 2 | 0.0004 |
|  | *Chone* sp. | 414 | 0.38306 |
|  | *Cistenides granulata* | 48 | 0.13054 |
|  | *Clymenura borealis* | 28 | 0.4227 |
|  | *Crystallophrisson nitidulum* | 4 | 0.09592 |
|  | *Cyrtodaria siliqua* | 2 | 3.45 |
|  | *Diastylis quadrispinosa* | 2 | 0.0104 |
|  | *Drilonereis magna* | 2 | 0.0002 |
|  | *Edwardsia elegans* | 8 | 0.04662 |
|  | *Epizoanthus* sp. | 216 | 0.1558 |
|  | *Ericthonius fasciatus* | 118 | 0.00826 |
|  | *Euchone papillosa* | 2 | 0.001 |
|  | *Euclymene zonalis* | 2 | 0.00304 |
|  | *Eudorella* sp. | 2 | 0.0004 |
|  | *Eulalia bilineata* | 4 | 0.01234 |
|  | *Exogone* sp. | 6 | 0.0004 |
|  | Flabelligeridae | 2 | 0.004 |
|  | *Galathowenia oculata* | 8 | 0.00146 |
|  | Gastropoda | 66 | 0.04508 |
|  | *Gattyana cirrhosa* | 2 | 0.0688 |
|  | *Goniada maculata* | 14 | 0.2675 |
|  | *Goniadella gracilis* | 6 | 0.00596 |
|  | *Harmothoe imbricata* | 6 | 0.0585 |
|  | *Hiatella arctica* | 2 | 0.0154 |
|  | *Hippomedon serratus* | 2 | 0.0004 |
|  | *Jasmineira* sp. | 24 | 0.0232 |
|  | *Laonice cirrata* | 2 | 0.004 |
|  | *Leaena ebranchiata* | 2 | 0.0026 |
|  | *Leptocheirus pinguis* | 2 | 0.0016 |
|  | *Lumbrinerides acuta* | 2 | 0.0013 |
|  | Lysianassidae | 6 | 0.00566 |
|  | *Macoma calcarea* | 2 | 0.0866 |
|  | *Mediomastus ambiseta* | 12 | 0.05092 |
|  | *Melina* sp. | 8 | 0.116 |
|  | *Moelleria costulata* | 2 | 0.0054 |
|  | Mytiloidea | 2 | 0.0004 |
|  | *Nassarius* sp. | 2 | 0.00876 |
|  | Nemertea | 16 | 0.53152 |
|  | *Nephtys caeca* | 6 | 0.0126 |
|  | *Nereis* sp. | 6 | 0.04382 |
|  | *Nicomache lumbricalis* | 2 | 0.1016 |
|  | *Nothria conchylega* | 80 | 0.4885 |
|  | *Notomastus latericeus* | 6 | 0.7144 |
|  | *Ophelia limacina* | 2 | 0.07738 |
|  | *Ophiacantha bidentata* | 2 | 0.02856 |
|  | *Ophiomitra robusta* | 22 | 0.00164 |
|  | *Ophiopholis aculeata* | 2 | 0.3164 |
|  | *Ophiura* sp. juvenile | 16 | 0.0044 |
|  | *Orbinia swani* | 10 | 0.10194 |
|  | *Owenia fusiformis* | 84 | 0.05388 |
|  | *Parvicardium pinnulatum* | 80 | 0.32588 |
|  | *Petaloproctus tenuis* | 28 | 0.056 |
|  | *Petalosarsia declivis* | 2 | 0.00008 |
|  | *Phyllodoce groenlandica* | 4 | 0.08978 |
|  | *Placopecten magellanicus* | 2 | 0.0014 |
|  | *Polycirrus* sp. | 2 | 0.0064 |
|  | *Polydora concharum* | 2 | 0.0062 |
|  | *Prionospio cirrifera* | 2 | 0.00016 |
|  | *Prionospio steenstrupi* | 2 | 0.0034 |
|  | *Psolus* sp. | 2 | 0.0062 |
|  | *Puncturella noachina* | 14 | 0.12806 |
|  | *Scalibregma inflatum* | 10 | 0.06428 |
|  | *Scoloplos armiger* | 16 | 0.53948 |
|  | Sipuncula | 2 | 0.001 |
|  | *Spio filicornis* | 2 | 0.0546 |
|  | Spionidae | 4 | 0.00024 |
|  | *Stenosemus albus* | 30 | 0.45892 |
|  | *Strongylocentrotus* sp. | 12 | 0.10684 |
|  | *Tharyx* sp. | 54 | 0.58358 |
|  | *Thyasira* sp. | 2 | 0.0058 |
|  | *Tonicella rubra* | 6 | 0.03226 |
|  | *Trichotropis borealis* | 2 | 0.0864 |
|  | *Unciola irrorata* | 14 | 0.0059 |
| eh_evg04 | *Aglaophamus circinata* | 6 | 0.0464 |
|  | *Alvania* sp. | 2 | 0.0016 |
|  | *Ampharete finmarchica* | 6 | 0.018 |
|  | *Arcteobia anticostiensis* | 2 | 0.0004 |
|  | *Arctica islandica* | 4 | 0.1948 |
|  | *Argissa hamatipes* | 2 | 0.0016 |
|  | *Aricidea catherinae* | 2 | 0.0002 |
|  | *Aricidea* sp. | 2 | 0.0008 |
|  | *Axionice maculata* | 2 | 0.0194 |
|  | *Boreocingula* sp. | 2 | 0.0028 |
|  | *Capitella capitata* | 2 | 0.0004 |
|  | *Chaetozone* sp. | 12 | 0.0246 |
|  | *Chone* sp. | 78 | 0.1166 |
|  | *Clymenura borealis* | 44 | 15.696 |
|  | *Cylichna alba* | 2 | 0.0786 |
|  | *Edwardsia elegans* | 6 | 0.065 |
|  | *Ericthonius fasciatus* | 36 | 0.0274 |
|  | *Euclymene zonalis* | 28 | 0.2474 |
|  | *Eudorella* sp. | 2 | 0.0002 |
|  | *Exogone* sp. | 16 | 0.0012 |
|  | Gastropoda | 2 | 0.0008 |
|  | *Glycera capitata* | 4 | 0.0482 |
|  | *Hippomedon serratus* | 2 | 0.033 |
|  | *Jasmineira* sp. | 2 | 0.0038 |
|  | *Levensinea gracilis* | 12 | 0.0044 |
|  | *Nereis* sp. | 2 | 0.0106 |
|  | *Nothria conchylega* | 6 | 0.1714 |
|  | *Notomastus latericeus* | 30 | 0.5628 |
|  | *Ophiura sarsi* | 12 | 0.0176 |
|  | *Orbinia swani* | 4 | 0.188 |
|  | *Owenia fusiformis* | 10 | 0.0106 |
|  | *Paraonis* sp. | 10 | 0.0038 |
|  | *Parvicardium pinnulatum* | 8 | 1.3718 |
|  | *Polycirrus* sp. | 2 | 0.0396 |
|  | Polygordiidae | 2 | 0.0002 |
|  | *Proclea graffi* | 24 | 0.0268 |
|  | *Scalibregma inflatum* | 4 | 0.0824 |
|  | *Scolelepis squamata* | 4 | 0.0314 |
|  | *Solariella obscura* | 2 | 0.0078 |
|  | *Spiochaetopterus typicus* | 2 | 0.0822 |
|  | *Stenopleustes inermis* | 2 | 0.0002 |
|  | Syllidae | 2 | 0.0002 |
|  | *Tharyx* sp. | 28 | 0.0484 |
|  | *Tiron spiniferus* | 2 | 0.0018 |
|  | *Travisia* sp. | 4 | 0.0202 |
|  | *Unciola irrorata* | 12 | 0.0406 |
| eh_vg01 | *Acirsa borealis* | 4 | 0.0524 |
|  | Actiniaria (non burrowing) | 56 | 0.0602 |
|  | *Aglaophamus circinata* | 6 | 0.2226 |
|  | *Ampharete finmarchica* | 2 | 0.0052 |
|  | *Amphitrite affinis* | 2 | 0.0202 |
|  | *Anonyx sarsi* | 2 | 0.0356 |
|  | *Argissa hamatipes* | 2 | 0.0012 |
|  | *Aricidea catherinae* | 4 | 0.0038 |
|  | *Aricidea* sp. | 16 | 0.011 |
|  | *Asabellides* sp. | 4 | 0.012 |
|  | *Astarte* sp. | 16 | 0.0416 |
|  | *Bathyarca pectunculoides* | 6 | 0.0506 |
|  | Buccinidae | 34 | 0.4644 |
|  | *Bylgides* sp. | 2 | 0.0162 |
|  | *Caprella* sp. | 10 | 0.0918 |
|  | Cerianthidae | 10 | 3.2698 |
|  | *Chaetozone* sp. | 12 | 0.0158 |
|  | *Chiridotea tuftsii* | 4 | 0.0136 |
|  | *Chone* sp. | 866 | 8.4322 |
|  | *Cistenides granulata* | 10 | 0.0064 |
|  | *Clymenura borealis* | 98 | 10.6488 |
|  | *Cylichna alba* | 12 | 0.1262 |
|  | *Diastylis quadrispinosa* | 4 | 0.0128 |
|  | *Drilonereis magna* | 4 | 0.3694 |
|  | *Dyopedos* sp. | 26 | 0.0154 |
|  | *Ensis directus* | 2 | 0.0322 |
|  | *Ericthonius fasciatus* | 1190 | 0.7222 |
|  | *Eudorella* sp. | 14 | 0.009 |
|  | *Eunice pennata* | 4 | 0.1254 |
|  | *Euspira* sp. | 2 | 0.0052 |
|  | *Euzonus flabelliferus* | 4 | 0.0726 |
|  | Gastropoda | 2 | 0.014 |
|  | *Gattyana cirrhosa* | 2 | 0.0066 |
|  | *Glycera capitata* | 24 | 1.7842 |
|  | *Golfingia* sp. | 30 | 0.1784 |
|  | *Goniada maculata* | 6 | 0.203 |
|  | *Harmothoe extenuata* | 4 | 0.8496 |
|  | *Harpinia propinqua* | 10 | 0.0056 |
|  | *Heteranomia squamula* | 2 | 0.015 |
|  | *Hippomedon serratus* | 2 | 0.005 |
|  | *Janira alta* | 38 | 0.1 |
|  | *Laonice cirrata* | 4 | 0.117 |
|  | *Lithodes maja* | 2 | 0.0328 |
|  | *Lumbrinerides acuta* | 2 | 0.0114 |
|  | *Lumbrineris fragilis* | 4 | 2.7004 |
|  | *Mediomastus ambiseta* | 16 | 0.387 |
|  | *Melina* sp. | 22 | 0.2796 |
|  | Mytiloidea | 2 | 0.2614 |
|  | *Nereis* sp. | 16 | 0.1016 |
|  | *Nicomache lumbricalis* | 4 | 1.872 |
|  | *Nothria conchylega* | 184 | 1.8316 |
|  | *Ophelina acuminata* | 4 | 0.0698 |
|  | *Ophiura robusta* | 12 | 0.0876 |
|  | *Ophiura sarsi* | 12 | 0.128 |
|  | *Orbinia swani* | 4 | 0.1022 |
|  | Ostracoda | 2 | 0.0032 |
|  | *Parvicardium pinnulatum* | 42 | 0.3958 |
|  | *Photis* sp. | 2 | 0.0024 |
|  | *Phyllodoce groenlandica* | 8 | 0.0708 |
|  | *Placopecten magellanicus* | 4 | 0.2208 |
|  | *Polycirrus* sp. | 2 | 0.0004 |
|  | Polygordiidae | 4 | 0.0012 |
|  | *Praxillella praetermissa* | 6 | 0.1742 |
|  | *Prionospio cirrifera* | 10 | 0.0128 |
|  | *Psammonyx* sp. | 10 | 0.07 |
|  | *Puncturella noachina* | 34 | 0.1404 |
|  | *Scalibregma inflatum* | 14 | 0.3774 |
|  | *Scolelepis squamata* | 4 | 0.0374 |
|  | *Streblosoma spiralis* | 2 | 0.0452 |
|  | *Strongylocentrotus* sp. | 4 | 0.0386 |
|  | Syllidae | 20 | 0.004 |
|  | *Tharyx* sp. | 32 | 0.4884 |
|  | *Tiron spiniferus* | 2 | 0.0084 |
|  | *Tonicella rubra* | 28 | 0.405 |
|  | *Unciola irrorata* | 4 | 0.0124 |
|  | *Westwoodilla brevicalar* | 2 | 0.003 |
| eh_vg02 | *Aglaophamus circinata* | 14 | 0.0878 |
|  | *Ampelisca macrocephala* | 2 | 0.024 |
|  | *Ampharete finmarchica* | 10 | 0.0212 |
|  | *Arctica islandica* | 2 | 0.1204 |
|  | *Aricidea wassi* | 12 | 0.0562 |
|  | *Chiridotea tuftsii* | 2 | 0.0236 |
|  | *Chone* sp. | 4 | 0.0056 |
|  | *Clymenura borealis* | 82 | 9.8726 |
|  | *Corymorpha pendula* | 2 | 0.3178 |
|  | *Dyopedos* sp. | 16 | 0.013 |
|  | *Edwardsia elegans* | 14 | 0.9004 |
|  | *Euzonus flabelliferus* | 2 | 0.0042 |
|  | Gastropoda | 2 | 0.0048 |
|  | *Hippomedon serratus* | 6 | 0.099 |
|  | *Nephtys caeca* | 12 | 0.0574 |
|  | *Notomastus latericeus* | 2 | 0.0012 |
|  | *Ophiura robusta* | 66 | 0.2266 |
|  | *Parvicardium pinnulatum* | 6 | 0.636 |
|  | *Phyllodoce groenlandica* | 4 | 0.4308 |
|  | *Psammonyx* sp. | 14 | 0.2792 |
|  | *Spiophanes bombyx* | 4 | 0.0216 |
|  | *Tharyx* sp. | 6 | 0.0094 |
|  | *Travisia* sp. | 16 | 0.0828 |
|  | *Unciola irrorata* | 4 | 0.0092 |
| eh_vg03 | *Aglaophamus circinata* | 2 | 0.018 |
|  | *Aricidea wassi* | 2 | 0.002 |
|  | Ascidiacea (solitary) | 2 | 0.0912 |
|  | *Chaetozone* sp. | 6 | 0.0054 |
|  | *Clymenura borealis* | 24 | 4.5242 |
|  | *Dyopedos* sp. | 2 | 0.0016 |
|  | *Edwardsia elegans* | 2 | 0.0828 |
|  | *Ericthonius fasciatus* | 8 | 0.0068 |
|  | *Exogone* sp. | 2 | 0.0002 |
|  | Gastropoda | 4 | 0.0216 |
|  | *Glycera capitata* | 6 | 0.0786 |
|  | *Lumbrinerides acuta* | 2 | 0.0794 |
|  | *Notomastus latericeus* | 2 | 0.0028 |
|  | *Ophelia limacina* | 2 | 0.786 |
|  | *Ophiura sarsi* | 24 | 0.0208 |
|  | *Orbinia swani* | 2 | 0.01 |
|  | *Paraonis* sp. | 2 | 0.0012 |
|  | *Periploma leanum* | 4 | 1.192 |
|  | *Psammonyx* sp. | 8 | 0.0858 |
|  | *Scolelepis squamata* | 2 | 0.888 |
|  | *Tharyx* sp. | 2 | 0.003 |
|  | *Unciola irrorata* | 14 | 0.06 |
| eh_vg04 | *Aglaophamus circinata* | 14 | 0.03694 |
|  | *Ampharete finmarchica* | 48 | 0.0072 |
|  | *Arctica islandica* | 4 | 0.0152 |
|  | *Aricidea catherinae* | 8 | 0.0026 |
|  | *Aricidea* sp. A | 4 | 0.00038 |
|  | *Astarte* sp. | 2 | 0.00072 |
|  | Asteroidea | 2 | 4.20352 |
|  | Buccinidae | 4 | 0.0018 |
|  | *Chaetozone* sp. | 52 | 0.00792 |
|  | *Chone* sp. | 46 | 0.00654 |
|  | *Clymenura borealis* | 110 | 2.15434 |
|  | *Cylichna alba* | 2 | 0.00674 |
|  | *Dyopedos* sp. | 14 | 0.00122 |
|  | *Echinarachnius parma* | 4 | 0.0313 |
|  | *Edwardsia elegans* | 2 | 0.01192 |
|  | *Ericthonius fasciatus* | 50 | 0.00488 |
|  | *Euclymene zonalis* | 22 | 0.01796 |
|  | *Galathowenia oculata* | 2 | 0.00032 |
|  | Gastropoda | 6 | 0.00084 |
|  | *Gattyana cirrhosa* | 2 | 0.00504 |
|  | *Glycera capitata* | 6 | 0.00262 |
|  | *Goniadella gracilis* | 4 | 0.01738 |
|  | *Hippomedon serratus* | 2 | 0.0025 |
|  | *Jaera dibranchiata* | 4 | 0.0004 |
|  | *Levensinea gracilis* | 8 | 0.00034 |
|  | *Lumbrinerides acuta* | 2 | 0.0004 |
|  | *Lumbrineris fragilis* | 10 | 0.01472 |
|  | *Mediomastus ambiseta* | 32 | 0.03542 |
|  | *Melina* sp. | 4 | 0.005 |
|  | *Nephtys discors* | 2 | 0.2595 |
|  | *Nereis* sp. | 2 | 0.00068 |
|  | *Nothria conchylega* | 12 | 0.01814 |
|  | *Ophelia limacina* | 2 | 0.0332 |
|  | *Ophiura robusta* | 44 | 0.00514 |
|  | *Orbinia swani* | 4 | 0.01094 |
|  | *Owenia fusiformis* | 6 | 0.00022 |
|  | *Paraonis* sp. | 6 | 0.00016 |
|  | *Parvicardium pinnulatum* | 2 | 0.07928 |
|  | *Scalibregma inflatum* | 4 | 0.00954 |
|  | *Scolelepis squamata* | 4 | 0.00292 |
|  | *Spiophanes bombyx* | 4 | 0.0158 |
|  | Syllidae | 10 | 0.00012 |
|  | *Tharyx* sp. | 22 | 0.00176 |
|  | *Travisia* sp. | 4 | 0.0047 |
|  | *Unciola irrorata* | 18 | 0.00386 |
| eh_vg05 | *Aglaophamus circinata* | 14 | 0.4606 |
|  | *Ampharete finmarchica* | 12 | 0.0484 |
|  | *Aricidea* sp. | 2 | 0.002 |
|  | *Aricidea wassi* | 6 | 0.0092 |
|  | Ascidiacea (solitary) | 64 | 1.2858 |
|  | *Chaetozone setosa* | 2 | 0.0036 |
|  | *Chaetozone* sp. | 12 | 0.0188 |
|  | *Chone* sp. | 4 | 0.0014 |
|  | *Clinocardium ciliatum* | 4 | 0.3644 |
|  | *Clymenura borealis* | 62 | 5.4628 |
|  | *Cylichna alba* | 6 | 0.1108 |
|  | *Cylichna occulata* | 2 | 0.0656 |
|  | *Dyopedos* sp. | 32 | 0.0176 |
|  | *Edwardsia elegans* | 4 | 0.1262 |
|  | *Ericthonius fasciatus* | 76 | 0.0858 |
|  | *Exogone* sp. | 2 | 0.0002 |
|  | Gastropoda | 4 | 0.0158 |
|  | *Glycera capitata* | 14 | 0.1668 |
|  | *Glycera dibranchiata* | 4 | 0.0868 |
|  | *Harmothoe extenuata* | 2 | 0.0056 |
|  | *Harmothoe imbricata* | 2 | 0.004 |
|  | *Hippomedon serratus* | 8 | 0.1176 |
|  | *Levensinea gracilis* | 2 | 0.0032 |
|  | *Lumbrinerides acuta* | 22 | 0.7096 |
|  | Nemertea | 4 | 1.5714 |
|  | *Nephtys caeca* | 4 | 2.478 |
|  | *Nephtys discors* | 2 | 2.3176 |
|  | Oligochaeta | 2 | 0.0006 |
|  | *Ophelia limacina* | 4 | 0.1936 |
|  | *Ophiura robusta* | 62 | 0.0496 |
|  | *Ophiura sarsi* | 68 | 0.0478 |
|  | *Orbinia swani* | 6 | 0.1244 |
|  | *Parvicardium pinnulatum* | 4 | 0.2028 |
|  | *Photis* sp. | 4 | 0.0028 |
|  | *Polydora caulleryi* | 2 | 0.0346 |
|  | *Polydora concharum* | 2 | 0.0274 |
|  | Polygordiidae | 4 | 0.0004 |
|  | *Prionospio cirrifera* | 2 | 0.001 |
|  | *Psammonyx* sp. | 18 | 0.531 |
|  | Questidae | 2 | 0.0002 |
|  | *Strongylocentrotus* sp. | 6 | 0.1848 |
|  | Syllidae | 8 | 0.0016 |
|  | *Tharyx* sp. | 10 | 0.0254 |
|  | *Travisia* sp. | 4 | 0.024 |
|  | *Unciola irrorata* | 18 | 0.0324 |
| eh_vg06 | Actiniaria (non burrowing) | 58 | 0.1066 |
|  | *Aeginina longicornis* | 30 | 0.159 |
|  | *Aglaophamus circinata* | 34 | 0.9894 |
|  | *Ampelisca aequicornis* | 10 | 0.0246 |
|  | *Ampelisca agassizi* | 2 | 0.0068 |
|  | *Ampharete finmarchica* | 20 | 0.2044 |
|  | Amphiuridae | 4 | 0.0146 |
|  | *Anomia* sp. | 24 | 0.1738 |
|  | *Anonyx sarsi* | 4 | 0.0354 |
|  | *Apistobranchus typicus* | 8 | 0.0126 |
|  | *Asabellides* sp. | 12 | 0.021 |
|  | Ascidiacea (solitary) | 10 | 0.0166 |
|  | *Astarte* sp. | 20 | 0.0358 |
|  | *Axionice maculata* | 4 | 0.0248 |
|  | *Axius serratus* | 4 | 0.691 |
|  | *Bathyarca pectunculoides* | 12 | 0.2608 |
|  | Cerianthidae | 2 | 0.139 |
|  | *Chaetozone* sp. | 78 | 0.2574 |
|  | *Chlamys islandica* | 24 | 0.713 |
|  | *Chone* sp. | 164 | 0.7462 |
|  | *Cistenides granulata* | 78 | 0.3722 |
|  | *Cucumaria frondosa* | 10 | 8.3622 |
|  | *Dendrobeania* sp. | 2 | 3.0598 |
|  | *Diastylis quadrispinosa* | 12 | 0.016 |
|  | *Eualus pusiolus* | 4 | 0.1988 |
|  | *Euchone papillosa* | 2 | 0.0006 |
|  | *Euclymene zonalis* | 16 | 0.4188 |
|  | *Eudorella* sp. | 6 | 0.006 |
|  | *Eunice pennata* | 16 | 1.0988 |
|  | *Eusirus cuspidatus* | 8 | 0.1128 |
|  | *Glycera capitata* | 18 | 0.5236 |
|  | *Golfingia* sp. | 24 | 0.0124 |
|  | *Goniada maculata* | 18 | 1.404 |
|  | *Harmothoe extenuata* | 6 | 0.0468 |
|  | *Harpinia plumosa* | 12 | 0.0106 |
|  | *Heteranomia squamula* | 18 | 0.093 |
|  | *Hippomedon serratus* | 2 | 0.004 |
|  | *Janira alta* | 26 | 0.091 |
|  | *Laonice cirrata* | 2 | 0.0716 |
|  | *Leptocheirus pinguis* | 18 | 0.0706 |
|  | *Leptostylis ampullacea* | 4 | 0.0032 |
|  | *Lumbrinerides acuta* | 78 | 1.1904 |
|  | *Melina* sp. | 20 | 0.132 |
|  | *Melita dentata* | 12 | 0.1028 |
|  | *Modiolus modiolus* | 2 | 0.1288 |
|  | *Moelleria costulata* | 46 | 0.0882 |
|  | Mytiloidea | 60 | 0.2902 |
|  | Nemertea | 6 | 0.1444 |
|  | *Nephtys discors* | 2 | 0.5212 |
|  | *Nereis* sp. | 6 | 0.1296 |
|  | *Ninoe nigripes* | 6 | 0.2506 |
|  | *Nothria conchylega* | 24 | 0.7488 |
|  | *Notomastus latericeus* | 16 | 0.7064 |
|  | *Ophiopholis aculeata* | 4 | 0.3592 |
|  | *Ophiura robusta* | 14 | 0.0974 |
|  | *Ophiura sarsi* | 2 | 0.1472 |
|  | *Ophiura* sp. juvenile | 46 | 0.1176 |
|  | *Orbinia swani* | 2 | 0.2052 |
|  | Ostracoda | 16 | 0.0244 |
|  | *Pagurus* sp. | 2 | 0.03 |
|  | *Paraonis* sp. | 22 | 0.027 |
|  | *Pardalisca cuspidata* | 2 | 0.0408 |
|  | *Parvicardium pinnulatum* | 24 | 0.0508 |
|  | *Phascolion strombus* | 14 | 0.1136 |
|  | *Pholoe minuta* | 4 | 0.0094 |
|  | *Phyllodoce groenlandica* | 2 | 0.0292 |
|  | *Phyllodoce maculata* | 2 | 0.0506 |
|  | *Platyhelminthes* | 2 | 0.0046 |
|  | *Pleurogonium spinosissimus* | 4 | 0.0018 |
|  | *Polycirrus* sp. | 12 | 0.0196 |
|  | *Polydora caulleryi* | 2 | 0.0058 |
|  | *Polydora concharum* | 4 | 0.0026 |
|  | Polygordiidae | 210 | 0.0778 |
|  | *Prionospio cirrifera* | 8 | 0.018 |
|  | *Psolus* sp. | 4 | 0.0312 |
|  | *Puncturella noachina* | 62 | 0.7582 |
|  | *Scalibregma inflatum* | 2 | 0.0448 |
|  | *Scolelepis squamata* | 4 | 0.093 |
|  | *Scoloplos armiger* | 4 | 0.027 |
|  | Serpulidae | 828 | 0.142 |
|  | *Strongylocentrotus* sp. | 34 | 1.304 |
|  | *Sycon* sp. | 6 | 0.008 |
|  | Syllidae | 136 | 0.0316 |
|  | *Terebratulina septentrionalis* | 162 | 8.6352 |
|  | *Tharyx* sp. | 96 | 2.2348 |
|  | *Thyasira* sp. | 46 | 0.292 |
|  | *Tonicella rubra* | 84 | 1.1572 |
|  | *Tricellaria gracilis* | 2 | 0.0124 |
|  | Turridae | 86 | 0.5956 |
| eh_vg07 | *Aglaophamus circinata* | 26 | 0.938 |
|  | *Ampelisca macrocephala* | 8 | 0.0656 |
|  | *Ampharete finmarchica* | 12 | 0.019 |
|  | *Arctica islandica* | 4 | 0.001 |
|  | *Aricidea catherinae* | 12 | 0.0056 |
|  | *Aricidea* sp. | 6 | 0.001 |
|  | Ascidiacea (solitary) | 2 | 0.0052 |
|  | *Axionice maculata* | 2 | 0.0032 |
|  | *Caprella* sp. | 2 | 0.002 |
|  | *Chaetozone* sp. A | 16 | 0.0448 |
|  | *Chiridotea tuftsii* | 2 | 0.009 |
|  | *Chone* sp. | 18 | 0.0212 |
|  | *Cirolana polita* | 2 | 0.0178 |
|  | *Clymenura borealis* | 48 | 13.4322 |
|  | *Cyclocardia novangliae* | 2 | 0.001 |
|  | *Cyclocardia* sp. A | 2 | 0.0008 |
|  | *Cylichna alba* | 2 | 0.0358 |
|  | *Dyopedos* sp. | 8 | 0.0068 |
|  | *Edotea montosa* | 2 | 0.0166 |
|  | *Ericthonius fasciatus* | 26 | 0.0222 |
|  | *Euclymene zonalis* | 6 | 0.0094 |
|  | *Exogone* sp. | 88 | 0.0122 |
|  | Gastropoda | 12 | 0.015 |
|  | *Glycera capitata* | 18 | 0.1616 |
|  | *Hippomedon serratus* | 10 | 0.1784 |
|  | *Jasmineira* sp. | 2 | 0.001 |
|  | *Lumbrinerides acuta* | 36 | 0.3388 |
|  | Lysianassidae | 2 | 0.0016 |
|  | Nemertea | 2 | 0.0008 |
|  | *Notomastus latericeus* | 2 | 0.0152 |
|  | *Ophelia limacina* | 2 | 0.528 |
|  | *Ophiura* sp. juvenile | 2 | 0.0002 |
|  | *Paraonis* sp. | 8 | 0.0034 |
|  | *Periploma leanum* | 36 | 6.8518 |
|  | *Photis* sp. | 2 | 0.0018 |
|  | *Polycirrus* sp. | 2 | 0.008 |
|  | Polygordiidae | 8 | 0.0006 |
|  | *Prionospio steenstrupi* | 2 | 0.0028 |
|  | Questidae | 2 | 0.0004 |
|  | *Scalibregma inflatum* | 4 | 0.235 |
|  | *Scolelepis squamata* | 10 | 0.0608 |
|  | *Spio filicornis* | 6 | 0.0102 |
|  | Syllidae | 32 | 0.0042 |
|  | *Syrrhoe crenulata* | 6 | 0.0354 |
|  | *Tharyx* sp. | 2 | 0.0112 |
|  | *Tiron spiniferus* | 2 | 0.0012 |
|  | *Unciola irrorata* | 54 | 0.2754 |
| eh_vg08 | *Aglaophamus circinata* | 28 | 0.0944 |
|  | *Ampharete finmarchica* | 4 | 0.039 |
|  | Amphiuridae | 2 | 0.004 |
|  | *Arcteobia anticostiensis* | 2 | 0.0156 |
|  | *Arctica islandica* | 2 | 0.009 |
|  | *Aricidea* sp. | 18 | 0.0032 |
|  | *Aricidea wassi* | 4 | 0.0044 |
|  | Ascidiacea (solitary) | 90 | 0.6392 |
|  | *Astarte* sp. | 4 | 0.0024 |
|  | Asteroidea | 2 | 0.0094 |
|  | *Boreocingula* sp. | 2 | 0.0012 |
|  | Cerianthidae | 4 | 3.4578 |
|  | *Chaetozone* sp. | 18 | 0.0236 |
|  | *Chlamys islandica* | 6 | 0.2538 |
|  | *Chone* sp. | 4 | 0.0018 |
|  | *Cucumaria frondosa* | 2 | 0.0102 |
|  | *Cyclocardia novangliae* | 2 | 0.785 |
|  | *Cylichna alba* | 6 | 0.0822 |
|  | *Edwardsia elegans* | 2 | 0.1806 |
|  | *Euspira* sp. | 12 | 0.0498 |
|  | *Exogone* sp. | 100 | 0.0114 |
|  | Gastropoda | 32 | 0.046 |
|  | *Glycera capitata* | 8 | 0.0176 |
|  | *Golfingia* sp. | 14 | 0.0122 |
|  | *Goniadella gracilis* | 6 | 0.0164 |
|  | *Harmothoe extenuata* | 2 | 0.001 |
|  | *Hiatella arctica* | 2 | 0.0138 |
|  | *Levensinea gracilis* | 4 | 0.0006 |
|  | *Lumbrinerides acuta* | 26 | 0.2794 |
|  | *Margarites striatus* | 2 | 0.0052 |
|  | Nemertea | 2 | 0.0014 |
|  | *Ophiura robusta* | 6 | 0.001 |
|  | *Ophiura* sp. juvenile | 2 | 0.005 |
|  | *Orbinia swani* | 2 | 0.0244 |
|  | *Owenia fusiformis* | 2 | 0.0012 |
|  | *Paraonis* sp. | 4 | 0.0018 |
|  | *Parvicardium pinnulatum* | 66 | 0.2192 |
|  | *Phascolion strombus* | 2 | 0.054 |
|  | Polygordiidae | 4 | 0.0022 |
|  | *Psolus* sp. | 2 | 0.0042 |
|  | *Puncturella noachina* | 4 | 0.0178 |
|  | *Scalibregma inflatum* | 4 | 0.099 |
|  | *Scolelepis* sp. A | 6 | 0.0832 |
|  | *Solariella obscura* | 2 | 0.0028 |
|  | *Spiophanes bombyx* | 8 | 0.0534 |
|  | *Stenosemus albus* | 2 | 0.0014 |
|  | *Strongylocentrotus* sp. | 2 | 0.0112 |
|  | *Tharyx* sp. | 16 | 0.0146 |
|  | *Travisia* sp. | 8 | 0.3588 |
|  | Trochidae | 2 | 0.0004 |
| eh_vg09 | *Acanthonotozoma serratum* | 2 | 0.0008 |
|  | Actiniaria (non burrowing) | 12 | 0.00216 |
|  | *Aglaophamus circinata* | 10 | 0.02238 |
|  | *Ampharete finmarchica* | 20 | 0.00466 |
|  | Amphiuridae | 48 | 0.00608 |
|  | *Anomia* sp. | 2 | 0.00076 |
|  | *Anonyx sarsi* | 4 | 0.00368 |
|  | *Arabella iricolor* | 8 | 0.26446 |
|  | *Aricidea* sp. | 6 | 0.00066 |
|  | *Asabellides* sp. | 10 | 0.00218 |
|  | Ascidiacea (solitary) | 2 | 0.00062 |
|  | *Astarte* sp. | 50 | 0.495 |
|  | *Axionice maculata* | 4 | 0.00064 |
|  | *Axius serratus* | 2 | 0.00168 |
|  | *Bathyarca pectunculoides* | 8 | 0.02022 |
|  | *Bylgides* sp. | 6 | 0.00404 |
|  | *Caprella* sp. | 2 | 0.0004 |
|  | Cerianthidae | 2 | 0.07762 |
|  | *Chaetozone* sp. | 32 | 0.0042 |
|  | *Chlamys islandica* | 18 | 0.13794 |
|  | *Chone* sp. | 812 | 0.39284 |
|  | *Cirolana polita* | 2 | 0.01146 |
|  | *Cistenides granulata* | 52 | 0.04186 |
|  | *Colus stimpsoni* | 64 | 0.01614 |
|  | *Crystallophrisson nitidulum* | 2 | 0.0009 |
|  | *Cylichna alba* | 10 | 0.00152 |
|  | *Dendrobeania* sp. | 2 | 0.00378 |
|  | *Diastylis quadrispinosa* | 4 | 0.0014 |
|  | *Drilonereis magna* | 2 | 0.01042 |
|  | *Ericthonius fasciatus* | 814 | 0.06118 |
|  | *Euchone papillosa* | 6 | 0.0042 |
|  | *Euclymene zonalis* | 8 | 0.00608 |
|  | *Eudorella* sp. | 16 | 0.00084 |
|  | *Eunice pennata* | 12 | 0.09654 |
|  | *Glycera capitata* | 36 | 0.26258 |
|  | *Golfingia* sp. | 42 | 0.0027 |
|  | *Goniada maculata* | 18 | 0.06652 |
|  | *Harmothoe extenuata* | 2 | 0.01436 |
|  | *Harpinia plumosa* | 24 | 0.00228 |
|  | *Hiatella arctica* | 2 | 0.00274 |
|  | *Janira alta* | 32 | 0.01066 |
|  | *Laonice cirrata* | 2 | 0.0069 |
|  | *Leptostylis ampullacea* | 2 | 0.00024 |
|  | *Lumbrinerides acuta* | 4 | 0.00034 |
|  | *Mactromeris polynyma* | 10 | 2.226 |
|  | *Melina* sp. | 18 | 0.01822 |
|  | *Modiolus modiolus* | 2 | 0.02102 |
|  | *Moelleria costulata* | 22 | 0.0017 |
|  | Mytiloidea | 2 | 0.00036 |
|  | *Myxicola infundibulum* | 4 | 0.1113 |
|  | Nemertea | 16 | 0.01778 |
|  | *Nephtys discors* | 2 | 0.00536 |
|  | *Nereis* sp. | 16 | 0.01492 |
|  | *Nicomache lumbricalis* | 2 | 0.01482 |
|  | *Nothria conchylega* | 108 | 0.11544 |
|  | *Notomastus latericeus* | 46 | 0.09656 |
|  | *Ophelina acuminata* | 8 | 0.0075 |
|  | *Ophiopholis aculeata* | 10 | 0.02188 |
|  | *Ophiura robusta* | 8 | 0.0048 |
|  | *Ophiura* sp. juvenile | 18 | 0.00486 |
|  | *Orbinia swani* | 12 | 0.00282 |
|  | Ostracoda | 4 | 0.00086 |
|  | *Owenia fusiformis* | 14 | 0.00228 |
|  | *Pagurus* sp. | 2 | 0.00302 |
|  | *Parvicardium pinnulatum* | 64 | 0.10508 |
|  | *Phascolion strombus* | 14 | 0.02068 |
|  | *Phyllodoce groenlandica* | 6 | 0.025 |
|  | *Pleurogonium spinosissimus* | 6 | 0.00014 |
|  | *Polycirrus* sp. | 6 | 0.00496 |
|  | *Polydora concharum* | 2 | 0.00072 |
|  | *Prionospio steenstrupi* | 12 | 0.00078 |
|  | *Puncturella noachina* | 44 | 0.0542 |
|  | Sabellidae | 6 | 0.00362 |
|  | *Scalibregma inflatum* | 18 | 0.03288 |
|  | *Scolelepis squamata* | 4 | 0.00116 |
|  | *Scoloplos armiger* | 8 | 0.0113 |
|  | *Stenosemus albus* | 2 | 0.0079 |
|  | *Strongylocentrotus* sp. | 22 | 0.06386 |
|  | Syllidae | 16 | 0.00006 |
|  | *Syrrhoe crenulata* | 4 | 0.00106 |
|  | *Terebellides stroemi* | 12 | 0.01418 |
|  | *Tharyx* sp. | 50 | 0.20816 |
|  | *Thysanoessa* sp. | 2 | 0.00254 |
|  | *Tiron spiniferus* | 6 | 0.00118 |
|  | *Tonicella rubra* | 8 | 0.00186 |
|  | *Unciola irrorata* | 44 | 0.01126 |
| eh_vg10 | Actiniaria (non burrowing) | 4 | 0.00494 |
|  | *Aglaophamus circinata* | 32 | 0.12544 |
|  | *Ampelisca aequicornis* | 2 | 0.00014 |
|  | *Ampharete finmarchica* | 12 | 0.00634 |
|  | *Amphipholis squamata* | 4 | 0.00022 |
|  | *Apistobranchus typicus* | 2 | 0.00012 |
|  | *Arctica islandica* | 8 | 0.01006 |
|  | *Aricidea* sp. | 96 | 0.00982 |
|  | Ascidiacea (solitary) | 16 | 7.7082 |
|  | *Astarte* sp. | 28 | 0.0485 |
|  | *Bathyarca pectunculoides* | 2 | 0.0012 |
|  | Buccinidae | 12 | 0.01608 |
|  | Cerianthidae | 4 | 0.06266 |
|  | *Chone* sp. | 296 | 0.06638 |
|  | *Cistenides granulata* | 32 | 0.0928 |
|  | *Clinocardium ciliatum* | 16 | 0.0788 |
|  | *Crystallophrisson nitidulum* | 4 | 0.1068 |
|  | *Cylichna alba* | 2 | 0.00568 |
|  | *Cyrtodaria siliqua* | 2 | 7.357 |
|  | *Drilonereis magna* | 4 | 0.0114 |
|  | *Edwardsia elegans* | 4 | 0.0043 |
|  | *Edwardsia sulcata* | 2 | 0.00254 |
|  | *Ericthonius fasciatus* | 178 | 0.01974 |
|  | *Euchone papillosa* | 4 | 0.0024 |
|  | *Euclymene zonalis* | 4 | 0.00116 |
|  | *Eudorella* sp. | 6 | 0.00022 |
|  | *Eunice pennata* | 4 | 0.0488 |
|  | *Euspira* sp. | 2 | 0.00036 |
|  | *Galathowenia oculata* | 2 | 0.0066 |
|  | Gastropoda | 10 | 0.0446 |
|  | *Gattyana cirrhosa* | 2 | 0.00376 |
|  | *Golfingia* sp. | 4 | 0.00044 |
|  | *Goniada maculata* | 8 | 0.02014 |
|  | *Harmothoe imbricata* | 6 | 0.00764 |
|  | *Harpinia plumosa* | 2 | 0.00006 |
|  | *Janira alta* | 2 | 0.0004 |
|  | *Jasmineira* sp. | 2 | 0.0014 |
|  | *Laonice cirrata* | 2 | 0.0418 |
|  | *Mediomastus ambiseta* | 2 | 0.0016 |
|  | *Melina* sp. | 14 | 0.0351 |
|  | Mytiloidea | 6 | 0.01166 |
|  | *Neosabellides* sp. | 4 | 0.0031 |
|  | *Nereis* sp. | 6 | 0.00812 |
|  | *Nothria conchylega* | 64 | 0.4686 |
|  | *Notomastus latericeus* | 10 | 0.02188 |
|  | Oligochaeta | 16 | 0.0194 |
|  | *Ophelina acuminata* | 2 | 0.00288 |
|  | *Ophiura robusta* | 10 | 0.00026 |
|  | *Owenia fusiformis* | 10 | 0.0158 |
|  | *Parvicardium pinnulatum* | 38 | 0.03862 |
|  | *Phascolion strombus* | 8 | 0.085 |
|  | *Polycirrus* sp. | 2 | 0.0018 |
|  | *Polydora concharum* | 6 | 0.0158 |
|  | *Puncturella noachina* | 6 | 0.0938 |
|  | *Scolelepis squamata* | 4 | 0.00454 |
|  | *Scoloplos armiger* | 10 | 0.00348 |
|  | *Solemya* sp. | 2 | 0.2162 |
|  | *Stenosemus albus* | 6 | 0.085 |
|  | *Strongylocentrotus* sp. | 8 | 0.00918 |
|  | *Thalassema* sp. | 2 | 0.00038 |
|  | *Tharyx* sp. | 20 | 0.04156 |
|  | *Thracia* sp. | 2 | 0.0004 |
|  | *Thyasira* sp. | 10 | 0.0672 |
|  | *Tonicella rubra* | 6 | 0.00818 |
|  | *Unciola irrorata* | 10 | 0.00962 |
| sc_vg02 | Actiniaria (non burrowing) | 2 | 0.0006 |
|  | *Aglaophamus circinata* | 62 | 3.4546 |
|  | *Ampelisca macrocephala* | 4 | 0.1308 |
|  | *Ampharete finmarchica* | 50 | 0.0898 |
|  | *Arctica islandica* | 80 | 46.0936 |
|  | *Aricidea catherinae* | 224 | 0.1382 |
|  | Ascidiacea (solitary) | 402 | 21.7508 |
|  | *Autolytus* sp. | 2 | 0.0008 |
|  | Campanulariidae | 2 | 0.9224 |
|  | *Cancer borealis* | 2 | 0.2842 |
|  | *Caprella* sp. | 40 | 0.0842 |
|  | Cerianthidae | 8 | 6.3652 |
|  | *Chiridotea tuftsii* | 2 | 0.0004 |
|  | *Chone* sp. | 2 | 0.0002 |
|  | *Cistenides granulata* | 52 | 0.3828 |
|  | *Clymenura borealis* | 6 | 2.2358 |
|  | *Corophium crassicorne* | 22 | 0.0092 |
|  | *Cylichna alba* | 4 | 0.0438 |
|  | *Cyrtodaria siliqua* | 4 | 0.06 |
|  | *Diastylis sculpta* | 2 | 0.0058 |
|  | *Echinarachnius parma* | 74 | 16.23 |
|  | *Edotea montosa* | 14 | 0.0718 |
|  | *Edwardsia elegans* | 1170 | 30.4462 |
|  | *Eteone longa* | 12 | 0.143 |
|  | *Euchone incolor* | 4 | 0.0002 |
|  | *Euclymene zonalis* | 14 | 5.4406 |
|  | *Eucratea loricata* | 2 | 0.002 |
|  | *Gattyana cirrhosa* | 6 | 0.0046 |
|  | *Goniada maculata* | 40 | 1.472 |
|  | *Hydrallmania falcata* | 2 | 0.3076 |
|  | *Leptocheirus pinguis* | 32 | 0.4902 |
|  | *Leptognathia* sp. | 2 | 0.002 |
|  | Nemertea | 16 | 9.4674 |
|  | Nephtyidae sp. juvenile | 46 | 0.1446 |
|  | *Nephtys caeca* | 6 | 9.896 |
|  | *Nereis* sp. | 4 | 0.0668 |
|  | Nuculidae | 206 | 1.5652 |
|  | *Ophelina breviata* | 2 | 0.0002 |
|  | *Ophiura sarsi* | 2 | 3.1196 |
|  | *Ophiura* sp. juvenile | 8 | 0.1024 |
|  | *Orchomenella minuta* | 2 | 0.0048 |
|  | Ostracoda | 4 | 0.001 |
|  | *Owenia fusiformis* | 266 | 0.7032 |
|  | *Pagurus* sp. | 2 | 0.0792 |
|  | *Paraedwardsia arenaria* | 2 | 0.196 |
|  | *Parvicardium pinnulatum* | 4 | 0.5816 |
|  | *Pelonaia corrugata* | 34 | 64.569 |
|  | *Pentamera calcigera* | 6 | 12.246 |
|  | *Pherusa affinis* | 16 | 8.555 |
|  | *Pholoe minuta* | 34 | 0.2158 |
|  | *Phoronis* sp. | 810 | 18.7386 |
|  | *Photis* sp. | 2 | 0.0016 |
|  | *Phyllodoce maculata* | 2 | 0.0002 |
|  | *Phyllodoce mucosa* | 4 | 0.0118 |
|  | Platyhelminthes | 4 | 0.0372 |
|  | *Podoceropsis* sp. | 2 | 0.001 |
|  | *Polycirrus* sp. | 2 | 0.0002 |
|  | *Polydora concharum* | 4 | 0.0204 |
|  | *Polydora socialis* | 62 | 0.1568 |
|  | *Pontogeneia inermis* | 4 | 0.006 |
|  | *Protomedeia fasciata* | 16 | 0.0074 |
|  | *Rhizocaulus verticillatus* | 2 | 0.008 |
|  | *Scoloplos armiger* | 50 | 1.0702 |
|  | *Serripes groenlandicus* | 28 | 0.1148 |
|  | *Sertularella* sp. | 2 | 0.0024 |
|  | *Sertularia mirabilis* | 2 | 0.0298 |
|  | *Sertularia* sp. | 2 | 0.0002 |
|  | *Spio filicornis* | 44 | 0.0492 |
|  | *Spiophanes bombyx* | 104 | 0.5366 |
|  | *Sthenelais limicola* | 10 | 0.039 |
|  | *Tharyx* sp. | 14 | 0.0258 |
|  | *Thracia* sp. | 2 | 0.2972 |
|  | *Thyasira* sp. | 6 | 0.024 |
|  | *Unciola irrorata* | 36 | 0.206 |
| sc_vg03 | *Acanthohaustorius spinosus* | 56 | 0.0368 |
|  | *Aeginina longicornis* | 4 | 0.003 |
|  | *Aglaophamus circinata* | 28 | 1.2266 |
|  | *Ampelisca macrocephala* | 2 | 0.0012 |
|  | *Ampharete finmarchica* | 28 | 0.1704 |
|  | *Anonyx sarsi* | 2 | 0.0026 |
|  | *Arctica islandica* | 4 | 0.079 |
|  | *Aricidea catherinae* | 80 | 0.0392 |
|  | *Aricidea* sp. B | 2 | 0.0008 |
|  | *Aricidea wassi* | 72 | 0.0906 |
|  | Ascidiacea (solitary) | 26 | 2.6246 |
|  | *Autolytus* sp. | 8 | 0.0038 |
|  | *Balanus* sp. | 2 | 0.0112 |
|  | Campanulariidae | 2 | 0.1946 |
|  | *Cancer borealis* | 2 | 0.304 |
|  | *Capitella capitata* | 8 | 0.0084 |
|  | Capitellidae | 2 | 0.0394 |
|  | *Caprella* sp. | 6 | 0.0766 |
|  | *Chiridotea tuftsii* | 22 | 0.0798 |
|  | *Chone* sp. | 8 | 0.0052 |
|  | *Cirolana polita* | 4 | 0.0538 |
|  | *Cistenides granulata* | 22 | 0.2006 |
|  | *Clymenella torquata* | 2 | 0.8734 |
|  | *Clymenura borealis* | 36 | 7.059 |
|  | *Colus* sp. | 2 | 0.0056 |
|  | *Corophium crassicorne* | 12 | 0.0096 |
|  | *Cylichna alba* | 6 | 0.0284 |
|  | *Dyopedos* sp. | 2 | 0.0032 |
|  | *Echinarachnius parma* | 70 | 4.4308 |
|  | *Edotea montosa* | 34 | 0.0752 |
|  | *Edwardsia elegans* | 408 | 25.464 |
|  | *Euchone papillosa* | 42 | 0.1454 |
|  | *Euclymene zonalis* | 10 | 0.4132 |
|  | *Eudorellopsis deformis* | 2 | 0.0018 |
|  | *Goniada maculata* | 22 | 0.373 |
|  | *Harmothoe extenuata* | 4 | 0.0118 |
|  | *Leptocheirus pinguis* | 42 | 0.8556 |
|  | *Leptognathia* sp. | 16 | 0.0038 |
|  | *Lumbrineris fragilis* | 2 | 0.0058 |
|  | *Macoma calcarea* | 4 | 0.0012 |
|  | *Mediomastus ambiseta* | 6 | 0.0044 |
|  | Nemertea | 6 | 7.0802 |
|  | Nephtyidae sp. juvenile | 20 | 0.0256 |
|  | *Nephtys caeca* | 28 | 13.1762 |
|  | *Nereis* sp. | 16 | 0.1608 |
|  | Nudibranchia Group 4B | 2 | 0.0002 |
|  | Oligochaeta | 20 | 0.0064 |
|  | *Ophelia limacina* | 12 | 0.1276 |
|  | *Orbinia swani* | 2 | 0.0684 |
|  | Ostracoda | 2 | 0.0004 |
|  | *Owenia fusiformis* | 8 | 0.0202 |
|  | *Pagurus* sp. | 2 | 0.0024 |
|  | *Paraonis* sp. | 56 | 0.054 |
|  | *Parvicardium pinnulatum* | 8 | 1.1312 |
|  | *Pherusa affinis* | 8 | 3.0772 |
|  | *Pholoe minuta* | 14 | 0.0394 |
|  | *Phoronis* sp. | 18 | 0.3066 |
|  | *Photis* sp. | 30 | 0.0156 |
|  | *Phyllodoce mucosa* | 30 | 0.1168 |
|  | Phyllodocidae sp. juvenile | 2 | 0.0072 |
|  | *Polydora concharum* | 42 | 0.3074 |
|  | *Polydora socialis* | 38 | 0.0794 |
|  | *Prionospio cirrifera* | 4 | 0.0002 |
|  | *Scoloplos armiger* | 70 | 0.8948 |
|  | *Serripes groenlandicus* | 30 | 0.0716 |
|  | *Sertularia* sp. | 2 | 2.0016 |
|  | *Spio filicornis* | 20 | 0.0414 |
|  | *Sthenelais limicola* | 8 | 0.01 |
|  | Syllidae | 12 | 0.0014 |
|  | *Tharyx* sp. | 26 | 0.1114 |
|  | *Unciola irrorata* | 128 | 0.5204 |
| sc_vg04 | Actiniaria (burrowing) | 10 | 1.1604 |
|  | *Aglaophamus circinata* | 30 | 1.4454 |
|  | *Amauropsis islandica* | 2 | 1.5932 |
|  | *Ampharete finmarchica* | 16 | 0.0108 |
|  | *Arctica islandica* | 4 | 0.1598 |
|  | *Aricidea catherinae* | 10 | 0.0134 |
|  | *Aricidea wassi* | 6 | 0.0114 |
|  | *Asabellides* sp. | 2 | 0.0022 |
|  | *Capitella capitata* | 12 | 0.0802 |
|  | *Chaetozone* sp. | 6 | 0.0118 |
|  | *Cirolana polita* | 6 | 0.5982 |
|  | *Cistenides granulata* | 28 | 0.4368 |
|  | *Clymenura borealis* | 50 | 2.9106 |
|  | *Corophium crassicorne* | 2 | 0.0006 |
|  | *Crangon septemspinosa* | 2 | 0.0078 |
|  | *Echinarachnius parma* | 62 | 1.5036 |
|  | *Edwardsia elegans* | 4 | 0.056 |
|  | *Euchone papillosa* | 46 | 0.1604 |
|  | *Euclymene zonalis* | 32 | 0.137 |
|  | *Hippomedon serratus* | 4 | 0.0818 |
|  | *Levensinea gracilis* | 6 | 0.0016 |
|  | *Lumbrinerides acuta* | 16 | 0.1132 |
|  | *Lumbrineris fragilis* | 4 | 1.1654 |
|  | *Modiolus modiolus* | 2 | 0.0002 |
|  | *Monoculodes* sp. | 2 | 0.0032 |
|  | Nemertea | 8 | 0.9624 |
|  | Nephtyidae sp. juvenile | 2 | 0.0156 |
|  | *Nephtys bucera* | 4 | 0.0034 |
|  | Nephtys caeca | 2 | 0.4898 |
|  | *Nereis* sp. | 6 | 0.1938 |
|  | *Notomastus latericeus* | 16 | 0.1396 |
|  | *Ophelia limacina* | 6 | 2.376 |
|  | *Orbinia swani* | 2 | 0.0516 |
|  | *Pagurus* sp. | 2 | 0.0028 |
|  | *Paraonis* sp. | 4 | 0.0016 |
|  | *Parvicardium pinnulatum* | 2 | 0.0004 |
|  | *Periploma leanum* | 16 | 2.8354 |
|  | *Pholoe tecta* | 2 | 0.0004 |
|  | *Phoxocephalus holbolli* | 4 | 0.0138 |
|  | *Polycirrus* sp. | 2 | 0.0082 |
|  | *Scolelepis squamata* | 4 | 0.0092 |
|  | *Scoloplos armiger* | 8 | 0.6684 |
|  | *Solariella obscura* | 2 | 0.053 |
|  | *Spiophanes bombyx* | 24 | 0.0738 |
|  | *Tharyx* sp. | 16 | 0.0206 |
|  | *Unciola irrorata* | 74 | 0.2896 |
| sc_vg05 | *Aglaophamus circinata* | 24 | 0.9742 |
|  | *Ampelisca macrocephala* | 2 | 0.1656 |
|  | *Ampharete finmarchica* | 48 | 0.0608 |
|  | *Anonyx sarsi* | 2 | 0.0038 |
|  | *Aricidea catherinae* | 6 | 0.0122 |
|  | *Aricidea* sp. A | 4 | 0.0052 |
|  | *Astarte* sp. | 2 | 0.0456 |
|  | *Chone* sp. | 28 | 0.144 |
|  | *Cistenides granulata* | 22 | 0.3782 |
|  | *Clymenura borealis* | 118 | 9.0252 |
|  | *Crangon septemspinosa* | 8 | 0.277 |
|  | *Cylichna alba* | 2 | 0.0604 |
|  | *Cyrtodaria siliqua* | 4 | 464.0316 |
|  | *Echinarachnius parma* | 104 | 422.969 |
|  | *Edwardsia sulcata* | 18 | 4.7406 |
|  | *Hippomedon serratus* | 10 | 0.0528 |
|  | *Lumbrinerides acuta* | 4 | 0.0674 |
|  | *Lumbrineris fragilis* | 8 | 1.6044 |
|  | *Margarites striatus* | 2 | 0.21 |
|  | *Monoculodes* sp. | 6 | 0.0196 |
|  | Nemertea | 12 | 2.4108 |
|  | *Nephtys caeca* | 16 | 14.357 |
|  | *Nereis* sp. | 4 | 0.218 |
|  | *Notomastus latericeus* | 30 | 0.3456 |
|  | *Ophelia limacina* | 4 | 0.0192 |
|  | *Orbinia swani* | 4 | 0.2168 |
|  | *Pagurus* sp. | 2 | 0.0046 |
|  | *Paraonis* sp. | 6 | 0.0014 |
|  | *Parvicardium pinnulatum* | 6 | 0.2138 |
|  | *Periploma leanum* | 32 | 4.0844 |
|  | *Phoxocephalus holbolli* | 28 | 0.0366 |
|  | Platyhelminthes | 4 | 0.0504 |
|  | Polygordiidae | 4 | 0.0002 |
|  | *Sertularia* sp. | 2 | 0.001 |
|  | *Spiophanes bombyx* | 12 | 0.0942 |
|  | *Sthenelais limicola* | 4 | 0.0116 |
|  | Syllidae | 4 | 0.0002 |
|  | *Thuiaria* sp. | 2 | 0.0002 |
|  | *Unciola irrorata* | 118 | 0.4668 |
| sc_vg06 | *Acanthohaustorius spinosus* | 2 | 0.0618 |
|  | Actiniaria (burrowing) | 2 | 0.5296 |
|  | *Aglaophamus circinata* | 62 | 4.1118 |
|  | *Ampharete finmarchica* | 30 | 0.4732 |
|  | *Anonyx sarsi* | 2 | 0.0054 |
|  | *Arcteobia anticostiensis* | 2 | 0.0068 |
|  | *Arctica islandica* | 12 | 0.221 |
|  | *Aricidea catherinae* | 32 | 0.0302 |
|  | *Aricidea wassi* | 26 | 0.036 |
|  | Ascidiacea (solitary) | 8 | 0.7762 |
|  | *Capitella capitata* | 14 | 0.024 |
|  | Capitellidae | 18 | 0.0094 |
|  | Cerianthidae | 2 | 7.4164 |
|  | *Chaetozone* sp. | 46 | 0.04 |
|  | *Chone* sp. | 8 | 0.0008 |
|  | *Cirolana polita* | 62 | 0.2328 |
|  | *Cistenides granulata* | 2 | 0.403 |
|  | *Clymenura borealis* | 154 | 10.792 |
|  | *Crangon septemspinosa* | 2 | 0.0136 |
|  | *Diaphana minuta* | 4 | 0.1404 |
|  | *Diastylis sculpta* | 6 | 0.003 |
|  | *Echinarachnius parma* | 116 | 6.6958 |
|  | *Edotea montosa* | 4 | 0.0052 |
|  | *Eteone longa* | 2 | 0.0028 |
|  | *Euchone papillosa* | 254 | 1.0716 |
|  | *Euclymene zonalis* | 190 | 0.632 |
|  | *Euspira* sp. | 4 | 0.5332 |
|  | *Glycera dibranchiata* | 2 | 1.7066 |
|  | *Goniadella gracilis* | 2 | 0.0034 |
|  | *Harmothoe extenuata* | 6 | 0.0112 |
|  | *Hippomedon serratus* | 4 | 0.0184 |
|  | *Leptocheirus pinguis* | 2 | 0.1368 |
|  | *Levensinea gracilis* | 18 | 0.0146 |
|  | *Lumbrinerides acuta* | 2 | 0.033 |
|  | *Lumbrineris fragilis* | 4 | 0.2004 |
|  | *Monoculodes* sp. | 2 | 0.0052 |
|  | *Nassarius* sp. | 2 | 0.42 |
|  | Nemertea | 12 | 9.5978 |
|  | *Nephtys caeca* | 14 | 0.6108 |
|  | *Nereis* sp. | 4 | 0.1794 |
|  | *Notomastus latericeus* | 58 | 0.2756 |
|  | *Ophelia limacina* | 8 | 0.0728 |
|  | *Orbinia swani* | 278 | 0.5388 |
|  | *Pagurus* sp. | 2 | 0.0052 |
|  | *Paraonis* sp. | 14 | 0.0046 |
|  | *Parvicardium pinnulatum* | 4 | 0.0018 |
|  | *Periploma leanum* | 24 | 3.0574 |
|  | *Pherusa affinis* | 2 | 0.0018 |
|  | *Pholoe tecta* | 4 | 0.0024 |
|  | *Phoxocephalus holbolli* | 14 | 0.0134 |
|  | *Phyllodoce mucosa* | 2 | 0.0004 |
|  | *Pleusymtes glaber* | 2 | 0.0004 |
|  | *Podoceropsis* sp. | 2 | 0.0006 |
|  | Polygordiidae | 6 | 0.001 |
|  | *Protomedeia fasciata* | 2 | 0.0002 |
|  | *Scolelepis squamata* | 4 | 0.0878 |
|  | *Scoloplos armiger* | 22 | 0.1684 |
|  | *Serripes groenlandicus* | 2 | 0.0002 |
|  | *Protomedeia fasciata* | 2 | 0.0002 |
|  | *Scolelepis squamata* | 2 | 0.0028 |
|  | *Scoloplos armiger* | 80 | 0.579 |
|  | *Serripes groenlandicus* | 4 | 0.0002 |
|  | *Tharyx* sp. | 72 | 0.1282 |
|  | *Unciola irrorata* | 276 | 0.907 |
| sc_vg07 | Actiniaria (burrowing) | 4 | 0.35 |
|  | Actiniaria (non burrowing) | 2 | 0.0118 |
|  | *Aglaophamus circinata* | 56 | 3.5068 |
|  | *Ampelisca macrocephala* | 2 | 0.0122 |
|  | *Ampharete finmarchica* | 96 | 0.15 |
|  | *Arctica islandica* | 6 | 0.308 |
|  | *Aricidea catherinae* | 14 | 0.0142 |
|  | *Aricidea wassi* | 4 | 0.0048 |
|  | Ascidiacea (solitary) | 60 | 5.7582 |
|  | *Cancer borealis* | 2 | 0.004 |
|  | *Capitella capitata* | 6 | 0.0088 |
|  | Capitellidae | 8 | 0.0076 |
|  | *Chaetozone setosa* | 10 | 0.028 |
|  | *Chone* sp. | 16 | 0.0376 |
|  | *Cirolana polita* | 18 | 0.8304 |
|  | *Cistenides granulata* | 54 | 0.5954 |
|  | *Clymenura borealis* | 54 | 5.279 |
|  | *Crangon septemspinosa* | 20 | 0.109 |
|  | *Cyrtodaria siliqua* | 2 | 0.0192 |
|  | *Diastylis sculpta* | 4 | 0.0022 |
|  | *Echinarachnius parma* | 150 | 6.3534 |
|  | *Edotea montosa* | 12 | 0.0374 |
|  | *Euchone papillosa* | 120 | 0.3136 |
|  | *Euclymene zonalis* | 90 | 0.9476 |
|  | *Euspira* sp. | 2 | 0.0034 |
|  | *Harmothoe extenuata* | 2 | 0.0038 |
|  | *Hippomedon serratus* | 2 | 0.0012 |
|  | *Levensinea gracilis* | 2 | 0.0004 |
|  | *Lumbrinerides acuta* | 6 | 0.077 |
|  | *Lumbrineris fragilis* | 2 | 0.2662 |
|  | *Monoculodes* sp. | 18 | 0.1016 |
|  | Nemertea | 4 | 0.502 |
|  | Nephtyidae sp. juvenile | 2 | 0.0018 |
|  | *Nephtys bucera* | 10 | 1.271 |
|  | *Nephtys caeca* | 26 | 3.4248 |
|  | *Nereis* sp. | 2 | 0.0022 |
|  | *Notomastus latericeus* | 32 | 0.1196 |
|  | *Ophelia limacina* | 4 | 0.0154 |
|  | *Ophelina acuminata* | 2 | 0.0536 |
|  | *Orbinia swani* | 18 | 1.383 |
|  | *Pagurus* sp. | 2 | 0.0026 |
|  | *Paraonis* sp. | 4 | 0.0008 |
|  | *Parougia eliasoni* | 4 | 0.0012 |
|  | *Parvicardium pinnulatum* | 6 | 0.0022 |
|  | *Periploma leanum* | 4 | 0.0126 |
|  | *Pholoe tecta* | 2 | 0.0002 |
|  | *Phoxocephalus holbolli* | 4 | 0.016 |
|  | Platyhelminthes | 12 | 0.1172 |
|  | *Pleusymtes glaber* | 4 | 0.0016 |
|  | *Polycirrus* sp. | 4 | 0.0006 |
|  | Polygordiidae | 4 | 0.0018 |
|  | *Protomedeia fasciata* | 2 | 0.0008 |
|  | *Scoloplos armiger* | 8 | 0.0786 |
|  | *Solariella obscura* | 14 | 2.1196 |
|  | *Spio filicornis* | 4 | 0.0042 |
|  | *Spiophanes bombyx* | 36 | 0.101 |
|  | *Sthenelais limicola* | 2 | 0.0156 |
|  | Syllidae | 8 | 0.0002 |
|  | *Tharyx* sp. | 6 | 0.0016 |
|  | *Unciola irrorata* | 176 | 0.5678 |
| sh_evg01 | *Achelia* sp. | 2 | 0.0002 |
|  | *Aeginina longicornis* | 2 | 0.0008 |
|  | *Aglaophamus circinata* | 12 | 1.0994 |
|  | *Ampharete finmarchica* | 644 | 1.4524 |
|  | *Aricidea catherinae* | 2 | 0.0002 |
|  | *Aricidea* sp. | 4 | 0.003 |
|  | *Aricidea wassi* | 2 | 0.0016 |
|  | Campanulariidae | 2 | 0.0002 |
|  | *Cancer borealis* | 4 | 0.0344 |
|  | Cerianthidae | 2 | 0.3544 |
|  | *Chaetozone* sp. | 12 | 0.0176 |
|  | *Cistenides granulata* | 2 | 0.0024 |
|  | *Clymenura borealis* | 20 | 1.132 |
|  | *Crangon septemspinosa* | 22 | 0.1626 |
|  | *Edwardsia elegans* | 6 | 0.09 |
|  | *Eteone longa* | 10 | 0.038 |
|  | *Euchone papillosa* | 6 | 0.003 |
|  | *Euclymene zonalis* | 2476 | 35.3734 |
|  | *Eulalia viridis* | 2 | 0.004 |
|  | *Exogone* sp. | 2 | 0.0002 |
|  | Gastropoda | 4 | 0.0002 |
|  | *Glycera capitata* | 4 | 0.8234 |
|  | *Glycera dibranchiata* | 6 | 0.3018 |
|  | *Goniadella gracilis* | 448 | 0.5276 |
|  | *Harmothoe extenuata* | 50 | 0.0632 |
|  | *Hiatella arctica* | 2 | 0.0028 |
|  | *Leptognathia* sp. | 2 | 0.0002 |
|  | *Levensinea gracilis* | 4 | 0.0014 |
|  | *Lumbrinerides acuta* | 184 | 1.3476 |
|  | *Lumbrineris fragilis* | 84 | 7.3586 |
|  | *Macoma calcarea* | 2 | 0.0002 |
|  | *Monoculodes* sp. | 16 | 0.0254 |
|  | Mytiloidea | 2 | 0.0022 |
|  | Nemertea | 64 | 0.5478 |
|  | *Nephtys caeca* | 14 | 1.4562 |
|  | *Nereis* sp. | 120 | 1.5074 |
|  | Nudibranchia Group 3 | 4 | 0.0058 |
|  | Oligochaeta | 54 | 0.0564 |
|  | *Ophelia limacina* | 2 | 2.1058 |
|  | *Ophiopholis aculeata* | 2 | 0.0002 |
|  | *Ophiura* sp. juvenile | 8 | 0.001 |
|  | *Orbinia swani* | 2 | 0.0292 |
|  | *Paraonis* sp. | 88 | 0.0348 |
|  | *Parvicardium pinnulatum* | 18 | 0.0026 |
|  | *Pherusa* sp. juvenile | 16 | 0.0112 |
|  | *Pholoe tecta* | 20 | 0.0158 |
|  | *Phyllodoce groenlandica* | 4 | 0.0996 |
|  | *Phyllodoce maculata* | 10 | 0.001 |
|  | *Phyllodoce mucosa* | 52 | 0.0742 |
|  | *Placopecten magellanicus* | 4 | 13.6386 |
|  | Platyhelminthes | 16 | 0.1032 |
|  | *Pleusymtes glaber* | 30 | 0.01 |
|  | *Polydora socialis* | 4 | 0.0006 |
|  | Polygordiidae | 142 | 0.0412 |
|  | *Pontogeneia inermis* | 2 | 0.0028 |
|  | *Protomedeia fasciata* | 6 | 0.0022 |
|  | *Psolus* sp. | 2 | 0.0012 |
|  | *Scalibregma inflatum* | 2 | 0.0052 |
|  | *Scolelepis* sp. A | 18 | 0.0208 |
|  | *Scolelepis squamata* | 2 | 0.146 |
|  | *Spio filicornis* | 6 | 0.0076 |
|  | *Spiophanes bombyx* | 12 | 0.0306 |
|  | Syllidae | 4 | 0.0002 |
|  | *Tharyx* sp. | 4 | 0.0068 |
|  | *Unciola irrorata* | 390 | 0.4954 |
| sh_evg02 | *Aglaophamus circinata* | 6 | 0.5696 |
|  | *Arcteobia anticostiensis* | 2 | 0.0032 |
|  | *Aricidea catherinae* | 4 | 0.0014 |
|  | *Astarte* sp. | 4 | 0.4244 |
|  | *Chaetozone* sp. | 10 | 0.009 |
|  | *Cistenides granulata* | 4 | 0.002 |
|  | *Crangon septemspinosa* | 28 | 0.2114 |
|  | *Cucumaria frondosa* | 6 | 0.0128 |
|  | *Edwardsia elegans* | 30 | 2.0052 |
|  | *Eteone longa* | 2 | 0.0002 |
|  | *Euchone papillosa* | 2 | 0.0002 |
|  | *Euspira* sp. | 2 | 0.0042 |
|  | Gastropoda | 2 | 0.0002 |
|  | *Glycera capitata* | 4 | 0.5338 |
|  | *Goniadella gracilis* | 170 | 0.3296 |
|  | *Harmothoe extenuata* | 10 | 0.0258 |
|  | *Leptognathia* sp. | 8 | 0.0018 |
|  | *Liocyma fluctuosa* | 4 | 0.0056 |
|  | *Lumbrineris fragilis* | 4 | 3.5752 |
|  | *Mactromeris polynyma* | 6 | 0.0048 |
|  | *Monoculodes* sp. | 2 | 0.0048 |
|  | Mytiloidea | 30 | 0.2514 |
|  | *Nephtys bucera* | 10 | 0.2732 |
|  | *Nephtys caeca* | 8 | 0.1752 |
|  | *Nereis* sp. | 52 | 0.8042 |
|  | Oligochaeta | 20 | 0.0346 |
|  | *Ophiura* sp. juvenile | 2 | 0.0002 |
|  | *Pagurus* sp. | 4 | 0.0276 |
|  | *Pandora* sp. | 4 | 0.0906 |
|  | *Paraonis* sp. | 36 | 0.0212 |
|  | *Parvicardium pinnulatum* | 20 | 0.0078 |
|  | *Pherusa* sp. juvenile | 12 | 0.005 |
|  | *Pholoe tecta* | 8 | 0.0048 |
|  | *Photis* sp. | 12 | 0.0032 |
|  | *Phyllodoce maculata* | 2 | 0.0088 |
|  | *Phyllodoce mucosa* | 28 | 0.0702 |
|  | Platyhelminthes | 16 | 0.0616 |
|  | *Pleusymtes glaber* | 2 | 0.0066 |
|  | *Polycirrus* sp. | 2 | 0.0044 |
|  | Polygordiidae | 90 | 0.0308 |
|  | *Psolus* sp. | 2 | 0.001 |
|  | *Scolelepis* sp. A | 24 | 0.0498 |
|  | *Spio filicornis* | 2 | 0.007 |
|  | *Spiophanes bombyx* | 10 | 0.0022 |
|  | *Sthenelais limicola* | 2 | 0.002 |
|  | *Tharyx* sp. | 30 | 0.0482 |
|  | *Thracia* sp. | 10 | 0.0028 |
|  | *Unciola irrorata* | 216 | 0.6152 |
| sh_vg01 | *Achelia* sp. | 8 | 0.0178 |
|  | Actiniaria (non burrowing) | 2 | 0.4818 |
|  | *Aeginina longicornis* | 12 | 0.0326 |
|  | *Aglaophamus circinata* | 6 | 1.3572 |
|  | *Ampharete finmarchica* | 332 | 0.563 |
|  | *Aricidea catherinae* | 6 | 0.0046 |
|  | *Astarte* sp. | 6 | 3.3102 |
|  | *Cancer borealis* | 18 | 0.0332 |
|  | *Caprella* sp. | 202 | 0.238 |
|  | Cerianthidae | 8 | 6.3226 |
|  | *Chaetozone* sp. A | 44 | 0.0694 |
|  | *Chone* sp. | 2 | 0.0016 |
|  | *Cistenides granulata* | 4 | 0.0028 |
|  | *Clymenura borealis* | 4 | 0.5164 |
|  | *Crangon septemspinosa* | 46 | 0.1192 |
|  | *Dendrobeania* sp. | 2 | 0.4382 |
|  | *Edwardsia elegans* | 8 | 0.1904 |
|  | *Eteone longa* | 2 | 0.0026 |
|  | *Euchone papillosa* | 2 | 0.0006 |
|  | *Glycera dibranchiata* | 10 | 0.3692 |
|  | *Goniadella gracilis* | 146 | 0.206 |
|  | *Harmothoe extenuata* | 30 | 0.9224 |
|  | *Lumbrinerides acuta* | 52 | 0.2478 |
|  | *Lumbrineris fragilis* | 56 | 2.55 |
|  | *Modiolus modiolus* | 92 | 0.011 |
|  | *Monoculodes* sp. | 4 | 0.0136 |
|  | Mytiloidea | 108 | 3.8644 |
|  | Nemertea | 8 | 0.0332 |
|  | Nephtyidae sp. juvenile | 26 | 0.4408 |
|  | *Nephtys bucera* | 8 | 4.73 |
|  | *Nereis* sp. | 130 | 1.707 |
|  | Nudibranchia Group 3 | 2 | 0.001 |
|  | Oligochaeta | 50 | 0.055 |
|  | *Ophiopholis aculeata* | 6 | 0.2474 |
|  | *Ophiura* sp. juvenile | 8 | 0.002 |
|  | *Pagurus* sp. | 4 | 0.037 |
|  | *Pandora* sp. | 4 | 0.6552 |
|  | *Paraonis* sp. | 58 | 0.0226 |
|  | *Pherusa affinis* | 8 | 0.6842 |
|  | *Pherusa* sp. juvenile | 16 | 0.023 |
|  | *Pholoe tecta* | 8 | 0.0146 |
|  | *Photis* sp. | 8 | 0.0018 |
|  | *Phyllodoce maculata* | 14 | 0.0412 |
|  | *Phyllodoce mucosa* | 12 | 0.008 |
|  | Platyhelminthes | 30 | 0.06 |
|  | *Pleusymtes glaber* | 54 | 0.0318 |
|  | *Polycirrus* sp. | 4 | 0.05 |
|  | *Polydora socialis* | 2 | 0.0002 |
|  | Polygordiidae | 120 | 0.04 |
|  | *Protodorvillea kefersteini* | 2 | 0.0002 |
|  | *Protomedeia fasciata* | 4 | 0.0002 |
|  | *Scalibregma inflatum* | 2 | 0.0024 |
|  | *Scolelepis* sp. A | 4 | 0.008 |
|  | *Spio filicornis* | 4 | 0.0142 |
|  | *Spiophanes bombyx* | 6 | 0.0006 |
|  | Stenothoidae | 2 | 0.0002 |
|  | Syllidae | 2 | 0.0002 |
|  | *Tharyx* sp. | 42 | 0.1496 |
|  | *Thracia* sp. | 12 | 0.0326 |
|  | *Unciola irrorata* | 952 | 2.6296 |
| sh_vg02 | *Ampharete finmarchica* | 26 | 0.2334 |
|  | *Arctica islandica* | 4 | 0.0212 |
|  | *Aricidea* sp. A | 6 | 0.0098 |
|  | *Aricidea wassi* | 14 | 0.0128 |
|  | Ascidiacea (solitary) | 16 | 0.0598 |
|  | Campanulariidae | 2 | 0.0066 |
|  | *Caprella* sp. | 8 | 0.0078 |
|  | *Cauloramphus* sp. | 2 | 0.0234 |
|  | *Chaetozone* sp. | 44 | 0.0862 |
|  | *Cirolana polita* | 2 | 0.0074 |
|  | *Cistenides granulata* | 6 | 0.007 |
|  | *Clymenura borealis* | 10 | 0.0402 |
|  | *Crangon septemspinosa* | 12 | 0.0482 |
|  | *Cyrtodaria siliqua* | 2 | 0.0094 |
|  | Echinodermata | 22 | 0.0022 |
|  | *Ensis directus* | 4 | 1.5942 |
|  | *Euchone papillosa* | 10 | 0.0038 |
|  | *Euclymene zonalis* | 16 | 0.084 |
|  | Gastropoda | 4 | 0.1062 |
|  | *Glycera dibranchiata* | 2 | 0.0426 |
|  | *Goniada maculata* | 4 | 0.0294 |
|  | *Harmothoe extenuata* | 4 | 0.007 |
|  | *Hippomedon serratus* | 6 | 0.0076 |
|  | *Hydrallmania falcata* | 2 | 0.0014 |
|  | *Levensinea gracilis* | 26 | 0.0112 |
|  | *Lumbrinerides acuta* | 78 | 0.8864 |
|  | *Lumbrineris fragilis* | 6 | 0.1132 |
|  | Nemertea | 18 | 1.383 |
|  | *Nephtys bucera* | 24 | 0.6916 |
|  | *Nereis* sp. | 8 | 0.0604 |
|  | *Notomastus latericeus* | 6 | 0.03 |
|  | *Ophelia limacina* | 20 | 1.5598 |
|  | *Orbinia swani* | 4 | 0.1212 |
|  | *Pandora* sp. | 4 | 0.0044 |
|  | *Parvicardium pinnulatum* | 6 | 0.0006 |
|  | *Pherusa plumosa* | 4 | 0.0022 |
|  | *Pholoe minuta* | 8 | 0.002 |
|  | *Photis* sp. | 2 | 0.0022 |
|  | *Phyllodoce mucosa* | 18 | 0.0192 |
|  | Platyhelminthes | 24 | 0.1624 |
|  | Pleustidae juvenile | 4 | 0.0016 |
|  | *Pleusymtes glaber* | 2 | 0.001 |
|  | Polygordiidae | 8 | 0.003 |
|  | *Scalibregma inflatum* | 2 | 0.0072 |
|  | *Scolelepis squamata* | 6 | 0.0154 |
|  | *Scoloplos armiger* | 32 | 0.3696 |
|  | *Sertularia* sp. | 2 | 0.0032 |
|  | *Spiophanes bombyx* | 14 | 0.019 |
|  | *Sthenelais limicola* | 10 | 0.5848 |
|  | Syllidae | 6 | 0.0006 |
|  | *Unciola irrorata* | 36 | 0.0682 |
| sh_vg03 | *Aeginina longicornis* | 2 | 0.0042 |
|  | *Aglaophamus circinata* | 22 | 2.3286 |
|  | *Ampharete finmarchica* | 390 | 1.2276 |
|  | *Arcteobia anticostiensis* | 2 | 0.0044 |
|  | *Arctica islandica* | 2 | 0.0002 |
|  | *Aricidea catherinae* | 8 | 0.005 |
|  | Asteroidea | 2 | 0.036 |
|  | *Cancer borealis* | 2 | 0.0036 |
|  | *Caprella* sp. | 6 | 0.009 |
|  | *Cauloramphus* sp. | 2 | 0.011 |
|  | Cerianthidae | 2 | 0.0676 |
|  | *Chaetozone* sp. | 12 | 0.014 |
|  | *Chiridotea tuftsii* | 2 | 0.0026 |
|  | *Cistenides granulata* | 6 | 0.0026 |
|  | *Clymenura borealis* | 6 | 0.619 |
|  | *Crangon septemspinosa* | 14 | 0.0312 |
|  | Cumacea sp. A | 2 | 0.0054 |
|  | *Cyrtodaria siliqua* | 12 | 0.014 |
|  | *Echinarachnius parma* | 2 | 0.0378 |
|  | *Edwardsia elegans* | 26 | 0.5522 |
|  | *Eteone longa* | 2 | 0.0054 |
|  | *Euchone papillosa* | 8 | 0.0042 |
|  | *Euclymene zonalis* | 484 | 3.4426 |
|  | Gastropoda | 6 | 0.0218 |
|  | *Glycera capitata* | 4 | 0.3308 |
|  | *Goniadella gracilis* | 86 | 0.1074 |
|  | *Harmothoe extenuata* | 6 | 0.01 |
|  | *Lumbrinerides acuta* | 82 | 0.8242 |
|  | *Lumbrineris fragilis* | 16 | 5.2484 |
|  | *Monoculodes* sp. | 2 | 0.0032 |
|  | Mytiloidea | 20 | 0.0332 |
|  | *Nassarius* sp. | 6 | 1.2274 |
|  | Nemertea | 28 | 0.1952 |
|  | Nephtyidae sp. juvenile | 8 | 0.0558 |
|  | *Nephtys bucera* | 4 | 0.9548 |
|  | *Nephtys caeca* | 4 | 6.0188 |
|  | *Nereis* sp. | 124 | 1.2008 |
|  | Nudibranchia Group 3 | 2 | 0.0002 |
|  | Oligochaeta | 6 | 0.0082 |
|  | *Ophelia limacina* | 4 | 0.0188 |
|  | *Ophiopholis aculeata* | 4 | 0.001 |
|  | *Ophiura robusta* | 2 | 0.01 |
|  | *Ophiura* sp. juvenile | 2 | 0.0004 |
|  | *Paraonis* sp. | 12 | 0.005 |
|  | *Parougia eliasoni* | 2 | 0.0002 |
|  | *Parvicardium pinnulatum* | 14 | 0.0056 |
|  | *Periploma leanum* | 2 | 0.0022 |
|  | *Pholoe tecta* | 4 | 0.003 |
|  | *Phyllodoce maculata* | 2 | 0.0012 |
|  | *Phyllodoce mucosa* | 24 | 0.0372 |
|  | Platyhelminthes | 34 | 0.162 |
|  | *Pleusymtes glaber* | 4 | 0.0046 |
|  | Polycirrus sp. | 2 | 0.0176 |
|  | Polygordiidae | 64 | 0.0168 |
|  | *Scolelepis* sp. A | 12 | 0.0098 |
|  | *Scoloplos armiger* | 2 | 0.0136 |
|  | *Spio filicornis* | 2 | 0.003 |
|  | *Spiophanes bombyx* | 12 | 0.0208 |
|  | *Sthenelais limicola* | 2 | 0.0026 |
|  | *Tharyx* sp. | 76 | 0.203 |
|  | *Unciola irrorata* | 960 | 2.327 |
| sh_vg04 | *Achelia* sp. | 4 | 0.013 |
|  | Actiniaria (non burrowing) | 2 | 0.009 |
|  | *Aeginina longicornis* | 8 | 0.0776 |
|  | *Aglaophamus circinata* | 6 | 1.7614 |
|  | *Ampharete finmarchica* | 92 | 0.1998 |
|  | *Anonyx sarsi* | 4 | 0.0402 |
|  | *Aricidea catherinae* | 44 | 0.0366 |
|  | Asteroidea | 8 | 8.5686 |
|  | *Autolytus* sp. | 4 | 0.00224 |
|  | Bryozoa Ascophora | 2 | 0.232 |
|  | *Caprella* sp. | 98 | 0.1878 |
|  | Cerianthidae | 8 | 3.0448 |
|  | *Chaetozone* sp. A | 22 | 0.026 |
|  | *Cistenides granulata* | 4 | 0.0012 |
|  | *Clymenura borealis* | 2 | 0.0002 |
|  | *Crangon septemspinosa* | 16 | 0.0998 |
|  | *Cucumaria frondosa* | 2 | 0.0016 |
|  | Didemnidae | 2 | 0.067 |
|  | *Echinarachnius parma* | 2 | 0.0002 |
|  | *Edwardsia elegans* | 8 | 0.2228 |
|  | *Ericthonius rubricornis* | 20 | 0.0362 |
|  | *Eteone longa* | 2 | 0.0038 |
|  | *Euclymene zonalis* | 486 | 6.0908 |
|  | Gastropoda | 2 | 0.0002 |
|  | *Glycera capitata* | 30 | 0.3986 |
|  | *Goniadella gracilis* | 4 | 0.0914 |
|  | *Grammaria* sp. | 2 | 0.041 |
|  | *Harmothoe extenuata* | 12 | 0.0498 |
|  | *Harmothoe imbricata* | 8 | 0.1388 |
|  | *Ischyrocerus* sp. | 16 | 0.009 |
|  | *Levensinea gracilis* | 2 | 0.0002 |
|  | *Lumbrinerides acuta* | 34 | 0.1664 |
|  | *Lumbrineris fragilis* | 32 | 0.4998 |
|  | *Margarites striatus* | 2 | 0.0614 |
|  | *Melita dentata* | 16 | 0.1988 |
|  | *Metopella* sp. | 2 | 0.0014 |
|  | *Modiolus modiolus* | 250 | 0.042 |
|  | *Nassarius* sp. | 2 | 0.2582 |
|  | Nemertea | 36 | 0.0922 |
|  | Nephtyidae sp. juvenile | 4 | 0.0514 |
|  | *Nephtys bucera* | 4 | 0.0208 |
|  | *Nephtys caeca* | 2 | 1.4066 |
|  | *Nereis* sp. | 70 | 0.7242 |
|  | Nudibranchia Group 3 | 4 | 0.0022 |
|  | *Nymphon grossipes* | 2 | 0.0074 |
|  | Oligochaeta | 36 | 0.0432 |
|  | *Ophiopholis aculeata* | 10 | 0.0516 |
|  | *Ophiura robusta* | 6 | 0.0548 |
|  | *Ophiura* sp. juvenile | 14 | 0.0136 |
|  | *Pagurus* sp. | 8 | 0.0672 |
|  | *Paraonis* sp. | 46 | 0.0188 |
|  | *Parvicardium pinnulatum* | 12 | 0.002 |
|  | *Pholoe tecta* | 4 | 0.002 |
|  | *Phoxocephalus holbolli* | 18 | 0.0054 |
|  | *Phyllodoce maculata* | 10 | 0.0198 |
|  | *Placopecten magellanicus* | 2 | 0.0002 |
|  | Platyhelminthes | 12 | 0.0998 |
|  | *Pleusymtes glaber* | 4 | 0.0022 |
|  | *Polycirrus* sp. | 6 | 0.0418 |
|  | Polygordiidae | 32 | 0.006 |
|  | *Protomedeia fasciata* | 2 | 0.0012 |
|  | *Psolus* sp. | 6 | 0.009 |
|  | *Scoloplos armiger* | 2 | 0.0068 |
|  | *Spio filicornis* | 2 | 0.0016 |
|  | *Tharyx* sp. | 32 | 0.0608 |
|  | *Unciola irrorata* | 618 | 1.9374 |
| sh_vg05 | *Aglaophamus circinata* | 14 | 1.3304 |
|  | *Ampharete finmarchica* | 50 | 0.1428 |
|  | *Arctica islandica* | 2 | 0.0006 |
|  | *Aricidea wassi* | 4 | 0.0028 |
|  | Ascidiacea (solitary) | 2 | 0.0012 |
|  | *Chaetozone setosa* | 28 | 0.0396 |
|  | *Cirolana polita* | 2 | 0.072 |
|  | *Clymenura borealis* | 14 | 2.9502 |
|  | *Crangon septemspinosa* | 30 | 0.1042 |
|  | Cumacea sp. A | 4 | 0.0048 |
|  | *Echinarachnius parma* | 2 | 0.0004 |
|  | *Edotea montosa* | 2 | 0.0132 |
|  | *Edwardsia elegans* | 2 | 0.0236 |
|  | *Ensis directus* | 2 | 0.0956 |
|  | *Eteone longa* | 2 | 0.0278 |
|  | *Euclymene zonalis* | 84 | 0.2474 |
|  | *Euspira* sp. | 2 | 0.0002 |
|  | Gastropoda | 4 | 0.0002 |
|  | *Glycera dibranchiata* | 4 | 0.1026 |
|  | *Goniadella gracilis* | 2 | 0.0002 |
|  | *Harmothoe extenuata* | 2 | 0.0012 |
|  | *Hippomedon serratus* | 2 | 0.0118 |
|  | *Leptognathia* sp. | 2 | 0.0004 |
|  | *Levensinea gracilis* | 6 | 0.0016 |
|  | *Lumbrinerides acuta* | 122 | 0.9828 |
|  | *Lumbrineris fragilis* | 2 | 0.0424 |
|  | *Monoculodes* sp. | 20 | 0.066 |
|  | Mytiloidea | 2 | 0.234 |
|  | Nemertea | 14 | 0.2938 |
|  | *Nephtys bucera* | 52 | 1.6284 |
|  | *Nereis* sp. | 8 | 0.1582 |
|  | *Notomastus latericeus* | 2 | 0.0162 |
|  | Nudibranchia Group 4b | 2 | 0.0002 |
|  | Oligochaeta | 12 | 0.014 |
|  | *Ophelia limacina* | 22 | 0.4116 |
|  | *Orbinia swani* | 8 | 0.2788 |
|  | *Orchomenella minuta* | 2 | 0.001 |
|  | *Parvicardium pinnulatum* | 4 | 0.0006 |
|  | Platyhelminthes | 24 | 0.1392 |
|  | *Polycirrus* sp. | 2 | 0.0004 |
|  | *Scolelepis squamata* | 16 | 0.023 |
|  | *Scoloplos armiger* | 6 | 0.056 |
|  | Sipuncula | 2 | 0.0014 |
|  | *Spiophanes bombyx* | 14 | 0.0672 |
|  | *Sthenelais limicola* | 14 | 0.0238 |
|  | *Unciola irrorata* | 24 | 0.062 |
| sh_vg06 | Actiniaria (burrowing) | 2 | 0.0014 |
|  | *Aglaophamus circinata* | 6 | 0.0472 |
|  | *Ampharete finmarchica* | 36 | 0.1354 |
|  | *Aricidea catherinae* | 4 | 0.0042 |
|  | *Aricidea* sp. A | 12 | 0.0084 |
|  | *Aricidea wassi* | 10 | 0.0012 |
|  | Ascidiacea (solitary) | 2 | 0.0012 |
|  | Bivalvia | 2 | 0.0004 |
|  | *Cistenides granulata* | 2 | 0.0012 |
|  | *Clymenura borealis* | 6 | 0.0028 |
|  | *Crangon septemspinosa* | 30 | 0.134 |
|  | Cumacea sp. A | 6 | 0.0044 |
|  | *Diastylis sculpta* | 18 | 0.02 |
|  | *Echinarachnius parma* | 2 | 0.1852 |
|  | *Ensis directus* | 4 | 0.0502 |
|  | Gastropoda | 2 | 0.0006 |
|  | *Glycera dibranchiata* | 6 | 0.6518 |
|  | *Harpinia serrata* | 2 | 0.003 |
|  | *Lumbrinerides acuta* | 58 | 0.2804 |
|  | *Lumbrineris fragilis* | 2 | 1.5082 |
|  | *Monoculodes* sp. | 4 | 0.0086 |
|  | *Nassarius* sp. | 4 | 0.4666 |
|  | Nemertea | 12 | 2.6022 |
|  | Nephtyidae sp. juvenile | 2 | 0.014 |
|  | *Nephtys bucera* | 22 | 0.6678 |
|  | *Notomastus latericeus* | 2 | 0.0018 |
|  | *Ophelia limacina* | 8 | 0.124 |
|  | *Orbinia swani* | 2 | 0.0076 |
|  | *Parvicardium pinnulatum* | 2 | 0.001 |
|  | *Phyllodoce maculata* | 2 | 0.0014 |
|  | Platyhelminthes | 24 | 0.1006 |
|  | *Scoloplos armiger* | 32 | 0.319 |
|  | *Spio filicornis* | 2 | 0.0024 |
|  | *Sthenelais limicola* | 22 | 0.0966 |
|  | *Tharyx* sp. | 12 | 0.0156 |
| sh_vg07 | *Alvania* sp. | 4 | 0.01562 |
|  | *Ampharete finmarchica* | 14 | 0.00754 |
|  | *Aricidea catherinae* | 2 | 0.00038 |
|  | *Chaetozone setosa* | 10 | 0.00056 |
|  | *Cirolana polita* | 2 | 0.01142 |
|  | *Clymenura borealis* | 10 | 0.01064 |
|  | *Crangon septemspinosa* | 18 | 0.00764 |
|  | *Cylichna alba* | 2 | 0.00974 |
|  | Echinodermata | 4 | 0.0018 |
|  | *Ensis directus* | 14 | 0.00194 |
|  | *Hippomedon serratus* | 2 | 0.0004 |
|  | *Levensinea gracilis* | 6 | 0.00018 |
|  | *Lumbrinerides acuta* | 88 | 0.08688 |
|  | *Lumbrineris fragilis* | 2 | 0.00628 |
|  | *Monoculodes* sp. | 2 | 0.0007 |
|  | Mytiloidea | 2 | 0.0042 |
|  | Nemertea | 16 | 0.0455 |
|  | *Nephtys bucera* | 26 | 0.44768 |
|  | *Nereis* sp. | 6 | 0.0022 |
|  | *Ophelia limacina* | 18 | 0.03938 |
|  | *Orbinia swani* | 4 | 0.00552 |
|  | *Orchomenella minuta* | 2 | 0.0069 |
|  | *Paraonis* sp. | 2 | 0.00004 |
|  | *Parvicardium pinnulatum* | 6 | 0.00016 |
|  | Platyhelminthes | 10 | 0.00338 |
|  | *Scoloplos armiger* | 8 | 0.00582 |
|  | *Spiophanes bombyx* | 2 | 0.0002 |
|  | *Sthenelais limicola* | 2 | 0.00098 |
|  | Syllidae | 2 | 0.0002 |
|  | *Unciola irrorata* | 6 | 0.00156 |
| sh_vg08 | *Ampharete finmarchica* | 10 | 0.0374 |
|  | *Amphiporeia* sp. | 18 | 0.156 |
|  | Ascidiacea (solitary) | 8 | 0.0578 |
|  | *Autolytus* sp. | 42 | 0.021 |
|  | *Cirolana polita* | 6 | 0.2116 |
|  | *Clymenura borealis* | 2 | 0.0124 |
|  | *Crangon septemspinosa* | 38 | 0.1142 |
|  | Cumacea sp. A | 2 | 0.002 |
|  | *Echinarachnius parma* | 4 | 0.182 |
|  | *Ensis directus* | 6 | 0.0094 |
|  | *Euspira* sp. | 4 | 0.002 |
|  | *Goniadella gracilis* | 12 | 0.0142 |
|  | *Hippomedon serratus* | 4 | 0.0448 |
|  | *Lumbrinerides acuta* | 64 | 0.3502 |
|  | *Lumbrineris fragilis* | 2 | 0.1302 |
|  | *Mactromeris polynyma* | 16 | 0.007 |
|  | *Monoculodes* sp. | 10 | 0.0298 |
|  | Nemertea | 4 | 0.7444 |
|  | *Nephtys bucera* | 42 | 3.9274 |
|  | *Ophelia limacina* | 62 | 11.2886 |
|  | *Periploma leanum* | 4 | 0.104 |
|  | Platyhelminthes | 12 | 0.1214 |
|  | Polygordiidae | 6 | 0.0004 |
|  | *Scolelepis* sp. A | 10 | 0.0068 |
|  | *Spiophanes bombyx* | 10 | 0.0526 |
| sh_vg09 | *Ampharete finmarchica* | 28 | 0.0632 |
|  | *Arctica islandica* | 4 | 0.0002 |
|  | *Aricidea wassi* | 12 | 0.0026 |
|  | Ascidiacea (solitary) | 26 | 0.0744 |
|  | *Bathyporeia quoddyensis* | 2 | 0.0028 |
|  | *Chaetozone* sp. A | 30 | 0.0184 |
|  | *Chiridotea tuftsii* | 2 | 0.0026 |
|  | *Cistenides granulata* | 2 | 0.0002 |
|  | *Clymenura borealis* | 4 | 0.02 |
|  | *Crangon septemspinosa* | 12 | 0.242 |
|  | Cumacea sp. A | 10 | 0.0104 |
|  | *Cyrtodaria siliqua* | 2 | 0.0016 |
|  | *Diastylis sculpta* | 2 | 0.0016 |
|  | *Echinarachnius parma* | 2 | 0.0008 |
|  | *Edwardsia elegans* | 2 | 0.0112 |
|  | *Eteone longa* | 2 | 0.0958 |
|  | *Euchone papillosa* | 2 | 0.0002 |
|  | *Euclymene zonalis* | 22 | 0.1106 |
|  | *Exogone* sp. | 4 | 0.0006 |
|  | *Glycera dibranchiata* | 2 | 0.0562 |
|  | *Goniadella gracilis* | 2 | 0.001 |
|  | *Harmothoe extenuata* | 4 | 0.0034 |
|  | *Harmothoe imbricata* | 12 | 0.025 |
|  | *Hippomedon serratus* | 10 | 0.0582 |
|  | *Leptognathia* sp. | 6 | 0.0002 |
|  | *Levensinea gracilis* | 28 | 0.0056 |
|  | *Lumbrinerides acuta* | 56 | 0.365 |
|  | *Lumbrineris fragilis* | 6 | 4.0174 |
|  | *Modiolus modiolus* | 2 | 0.0008 |
|  | *Monoculodes* sp. | 12 | 0.0216 |
|  | Mytiloidea | 2 | 0.0006 |
|  | *Nassarius* sp. | 4 | 0.1688 |
|  | Nemertea | 20 | 0.1104 |
|  | *Nephtys bucera* | 30 | 1.4458 |
|  | *Nephtys caeca* | 4 | 0.4318 |
|  | *Nereis* sp. | 4 | 0.0898 |
|  | *Notomastus latericeus* | 2 | 0.0006 |
|  | *Ophelia limacina* | 20 | 1.7768 |
|  | *Ophiura* sp. juvenile | 2 | 0.0002 |
|  | *Orbinia swani* | 4 | 0.13 |
|  | *Orchomenella minuta* | 4 | 0.004 |
|  | *Paraonis* sp. | 16 | 0.0024 |
|  | *Parvicardium pinnulatum* | 4 | 0.0008 |
|  | *Pholoe tecta* | 2 | 0.0002 |
|  | *Photis* sp. | 2 | 0.0002 |
|  | *Phyllodoce mucosa* | 10 | 0.0058 |
|  | Platyhelminthes | 26 | 0.0772 |
|  | Polygordiidae | 12 | 0.0024 |
|  | *Protomedeia fasciata* | 2 | 0.0002 |
|  | *Scolelepis* sp. A | 12 | 0.0102 |
|  | *Scoloplos armiger* | 14 | 0.153 |
|  | *Spiophanes bombyx* | 18 | 0.0144 |
|  | *Tharyx* sp. | 30 | 0.0134 |
|  | *Unciola irrorata* | 10 | 0.0076 |
| sh_vg10 | *Achelia* sp. | 4 | 0.0016 |
|  | *Admete* sp. | 2 | 0.2058 |
|  | *Aeginina longicornis* | 18 | 0.06 |
|  | *Aglaophamus circinata* | 2 | 0.1276 |
|  | *Ampharete finmarchica* | 170 | 0.768 |
|  | *Anonyx sarsi* | 2 | 0.02 |
|  | Asteroidea | 12 | 1.1934 |
|  | *Balanus* sp. | 4 | 0.0114 |
|  | Bryozoa Ascophora | 2 | 0.0284 |
|  | *Cancer borealis* | 46 | 1.0378 |
|  | *Capitella capitata* | 2 | 0.003 |
|  | *Caprella* sp. | 92 | 0.1438 |
|  | *Cauloramphus* sp. | 2 | 0.0314 |
|  | Cerianthidae | 6 | 1.6564 |
|  | *Chaetozone* sp. A | 12 | 0.0134 |
|  | *Clymenura borealis* | 8 | 1.3168 |
|  | *Crangon septemspinosa* | 26 | 0.0956 |
|  | *Cucumaria frondosa* | 36 | 0.1182 |
|  | *Dendrobeania* sp. | 2 | 0.0066 |
|  | *Edwardsia* sp. | 2 | 0.28 |
|  | *Eteone longa* | 6 | 0.008 |
|  | *Eualus pusiolus* | 8 | 0.0466 |
|  | *Euclymene zonalis* | 1186 | 13.5472 |
|  | *Eucratea loricata* | 2 | 0.0242 |
|  | *Eudendrium* sp. | 2 | 0.001 |
|  | *Eulalia viridis* | 2 | 0.0044 |
|  | Gastropoda | 2 | 0.0002 |
|  | *Glycera capitata* | 12 | 1.3244 |
|  | *Goniadella gracilis* | 166 | 0.2012 |
|  | *Harmothoe extenuata* | 64 | 0.1032 |
|  | *Harmothoe imbricata* | 6 | 0.238 |
|  | *Hiatella arctica* | 2 | 0.0002 |
|  | *Hippomedon serratus* | 2 | 0.0114 |
|  | *Hyas coarctatus* | 2 | 0.0152 |
|  | *Hydrozoa Athecata* | 2 | 0.0388 |
|  | *Lumbrinerides acuta* | 76 | 0.4538 |
|  | *Lumbrineris fragilis* | 82 | 3.31 |
|  | *Melita dentata* | 14 | 0.1726 |
|  | *Modiolus modiolus* | 36 | 0.0004 |
|  | *Monoculodes* sp. | 6 | 0.0122 |
|  | Mytiloidea | 60 | 0.0666 |
|  | Nemertea | 46 | 0.1134 |
|  | *Nephtys caeca* | 6 | 12.6278 |
|  | *Nereis* sp. | 42 | 0.5462 |
|  | Nudibranchia Group 3 | 4 | 0.0022 |
|  | *Nymphon grossipes* | 2 | 0.0012 |
|  | Oedicerotidae | 2 | 0.0044 |
|  | Oligochaeta | 30 | 0.0298 |
|  | *Ophiopholis aculeata* | 6 | 0.0034 |
|  | *Ophiura* sp. juvenile | 34 | 0.011 |
|  | Ostracoda | 2 | 0.0002 |
|  | *Paraonis* sp. | 8 | 0.0264 |
|  | *Parvicardium pinnulatum* | 12 | 0.0036 |
|  | *Pherusa* sp. juvenile | 32 | 0.0202 |
|  | *Pholoe tecta* | 28 | 0.0216 |
|  | *Phyllodoce maculata* | 16 | 0.0098 |
|  | *Phyllodoce mucosa* | 8 | 0.0136 |
|  | Platyhelminthes | 14 | 0.069 |
|  | *Pleusymtes glaber* | 36 | 0.017 |
|  | *Podoceropsis* sp. | 52 | 0.0052 |
|  | *Polycirrus* sp. | 10 | 0.0624 |
|  | Polygordiidae | 14 | 0.0046 |
|  | Polynoidae sp. juvenile | 2 | 0.0002 |
|  | *Pontogeneia inermis* | 22 | 0.0304 |
|  | *Protomedeia fasciata* | 8 | 0.0038 |
|  | *Psolus* sp. | 12 | 0.0116 |
|  | *Rhizocaulus verticillatus* | 2 | 0.0294 |
|  | *Scalibregma inflatum* | 2 | 0.0086 |
|  | *Scolelepis squamata* | 4 | 0.016 |
|  | *Sertularia* sp. | 2 | 0.0018 |
|  | *Spio filicornis* | 4 | 0.0064 |
|  | Stenothoidae | 8 | 0.0002 |
|  | *Strongylocentrotus* sp. | 12 | 0.0162 |
|  | Syllidae | 2 | 0.0002 |
|  | *Thuiaria* sp. | 2 | 0.0088 |
|  | *Unciola irrorata* | 468 | 1.5066 |
| vg02 | *Aglaophamus circinata* | 2 | 0.47 |
|  | *Ampharete finmarchica* | 2 | 0.0046 |
|  | *Aricidea catherinae* | 4 | 0.0094 |
|  | Aricidea sp. B | 4 | 0.0088 |
|  | Ascidiacea (solitary) | 2 | 0.0084 |
|  | *Chaetozone* sp. A | 14 | 0.0632 |
|  | *Chiridotea tuftsii* | 4 | 0.0072 |
|  | *Clymenura borealis* | 18 | 4.026 |
|  | *Edwardsia elegans* | 6 | 0.1726 |
|  | *Ericthonius fasciatus* | 4 | 0.0116 |
|  | *Euclymene zonalis* | 22 | 0.1552 |
|  | *Exogone* sp. | 24 | 0.0102 |
|  | Gastropoda | 2 | 0.0038 |
|  | *Glycera capitata* | 12 | 0.092 |
|  | *Hippomedon serratus* | 4 | 0.026 |
|  | *Laphania boecki* | 2 | 0.0472 |
|  | *Lumbrinerides acuta* | 6 | 0.1216 |
|  | *Nothria conchylega* | 2 | 0.0632 |
|  | Oligochaeta | 4 | 0.0002 |
|  | *Ophiura* sp. juvenile | 2 | 0.0018 |
|  | *Orbinia swani* | 2 | 0.1188 |
|  | *Paraonis* sp. | 10 | 0.012 |
|  | *Parvicardium pinnulatum* | 2 | 0.0822 |
|  | *Periploma leanum* | 2 | 0.4796 |
|  | *Polycirrus* sp. | 4 | 0.007 |
|  | Polygordiidae | 4 | 0.0024 |
|  | *Psammonyx* sp. | 2 | 0.0366 |
|  | *Solariella obscura* | 2 | 0.0042 |
|  | *Spio filicornis* | 4 | 0.0034 |
|  | *Stenopleustes inermis* | 2 | 0.0004 |
|  | Syllidae | 12 | 0.0014 |
|  | *Unciola irrorata* | 14 | 0.0302 |
| vg03 | Actiniaria (burrowing) | 2 | 0.1784 |
|  | *Aglaophamus circinata* | 6 | 0.1178 |
|  | *Arctica islandica* | 2 | 0.017 |
|  | *Aricidea wassi* | 4 | 0.0174 |
|  | *Chaetozone* sp. A | 2 | 0.0032 |
|  | *Clymenura borealis* | 26 | 3.835 |
|  | *Corymorpha pendula* | 2 | 0.8322 |
|  | *Diaphana minuta* | 2 | 0.0054 |
|  | *Echinarachnius parma* | 8 | 1.097 |
|  | *Ericthonius fasciatus* | 2 | 0.0026 |
|  | *Euchone papillosa* | 2 | 0.005 |
|  | *Euzonus flabelliferus* | 2 | 0.0312 |
|  | *Glycera capitata* | 12 | 0.1096 |
|  | *Hippomedon serratus* | 2 | 0.0556 |
|  | *Lumbrinerides acuta* | 8 | 0.253 |
|  | *Mediomastus ambiseta* | 2 | 0.001 |
|  | Nemertea | 4 | 0.324 |
|  | *Notomastus latericeus* | 2 | 0.159 |
|  | *Ophelia limacina* | 2 | 4.5638 |
|  | *Ophiura sarsi* | 8 | 0.036 |
|  | *Paraonis* sp. | 2 | 0.004 |
|  | *Parvicardium pinnulatum* | 4 | 0.0256 |
|  | *Periploma leanum* | 4 | 4.392 |
|  | *Polycirrus* sp. | 2 | 0.0016 |
|  | *Scolelepis* sp. A | 2 | 0.0066 |
|  | *Solariella obscura* | 4 | 0.0078 |
|  | *Travisia* sp. | 4 | 0.2854 |
|  | *Unciola irrorata* | 4 | 0.0228 |
| vg04 | *Aglaophamus circinata* | 10 | 0.0846 |
|  | *Ampharete finmarchica* | 2 | 0.0024 |
|  | *Arcteobia anticostiensis* | 4 | 0.0072 |
|  | *Arctica islandica* | 10 | 0.176 |
|  | *Asabellides* sp. | 2 | 0.0004 |
|  | Ascidiacea (solitary) | 4 | 0.2392 |
|  | *Axionice maculata* | 4 | 0.0058 |
|  | *Chaetozone* sp. A | 8 | 0.027 |
|  | *Chiridotea tuftsii* | 6 | 0.0108 |
|  | *Clymenura borealis* | 30 | 8.134 |
|  | *Corymorpha pendula* | 2 | 1.0404 |
|  | *Edwardsia elegans* | 2 | 0.0018 |
|  | *Ericthonius fasciatus* | 8 | 0.0292 |
|  | *Glycera capitata* | 4 | 0.0424 |
|  | *Goniada maculata* | 4 | 0.0538 |
|  | *Hippomedon serratus* | 2 | 0.0078 |
|  | *Leptognathia* sp. | 2 | 0.0002 |
|  | Nemertea | 6 | 0.4292 |
|  | *Nothria conchylega* | 2 | 0.0032 |
|  | *Notomastus latericeus* | 12 | 0.8492 |
|  | *Ophiura* sp. juvenile | 2 | 0.001 |
|  | *Orbinia swani* | 2 | 0.1676 |
|  | *Owenia fusiformis* | 2 | 0.0032 |
|  | *Paraonis* sp. | 2 | 0.0006 |
|  | *Periploma leanum* | 2 | 0.8054 |
|  | *Prionospio cirrifera* | 2 | 0.003 |
|  | Sabellidae | 2 | 0.0006 |
|  | *Scolelepis* sp. A | 2 | 0.0296 |
|  | *Solariella obscura* | 6 | 0.0132 |
|  | *Spiochaetopterus typicus* | 16 | 0.3284 |
|  | *Spiophanes bombyx* | 6 | 0.0186 |
|  | *Travisia* sp. | 4 | 0.1148 |
|  | *Unciola irrorata* | 6 | 0.0406 |
| vg05 | *Aglaophamus circinata* | 6 | 0.1492 |
|  | *Ampelisca macrocephala* | 2 | 0.0102 |
|  | *Ampharete finmarchica* | 6 | 0.0536 |
|  | *Aricidea* sp. A | 2 | 0.0034 |
|  | *Aricidea wassi* | 2 | 0.0002 |
|  | *Axionice maculata* | 8 | 0.04 |
|  | *Chaetozone* sp. A | 2 | 0.0012 |
|  | *Cirolana polita* | 6 | 0.1468 |
|  | *Clymenura borealis* | 66 | 14.652 |
|  | *Diastylis sculpta* | 2 | 0.003 |
|  | *Echinarachnius parma* | 2 | 0.5676 |
|  | *Edwardsia elegans* | 4 | 0.1836 |
|  | *Exogone* sp. | 2 | 0.0002 |
|  | *Galathowenia oculata* | 4 | 0.0158 |
|  | *Glycera capitata* | 2 | 0.0154 |
|  | *Hippomedon serratus* | 2 | 0.0378 |
|  | *Lumbrinerides acuta* | 10 | 0.123 |
|  | Nemertea | 2 | 0.0056 |
|  | *Notomastus latericeus* | 2 | 0.0168 |
|  | *Ophiura sarsi* | 2 | 0.0146 |
|  | *Orbinia swani* | 2 | 0.031 |
|  | *Owenia fusiformis* | 2 | 0.0008 |
|  | *Paraonis* sp. | 4 | 0.0018 |
|  | *Parvicardium pinnulatum* | 4 | 0.4268 |
|  | *Periploma leanum* | 4 | 4.6336 |
|  | *Polycirrus* sp. | 8 | 0.0214 |
|  | *Polydora caulleryi* | 4 | 0.0396 |
|  | *Prionospio cirrifera* | 2 | 0.0054 |
|  | *Psammonyx* sp. | 4 | 0.088 |
|  | *Scolelepis* sp. A | 4 | 0.031 |
|  | *Spiochaetopterus typicus* | 10 | 0.462 |
|  | *Spiophanes bombyx* | 8 | 0.177 |
|  | *Strongylocentrotus* sp. | 2 | 0.0002 |
|  | *Travisia* sp. | 6 | 0.1938 |
|  | *Unciola irrorata* | 6 | 0.0232 |
| vg06 | *Aglaophamus circinata* | 12 | 0.7 |
|  | *Ampelisca vadorum* | 2 | 0.0122 |
|  | *Ampharete finmarchica* | 4 | 0.0046 |
|  | Amphiuridae | 2 | 0.0012 |
|  | *Arcteobia anticostiensis* | 2 | 0.0006 |
|  | *Arctica islandica* | 12 | 0.0082 |
|  | *Asabellides* sp. | 2 | 0.0058 |
|  | *Astarte* sp. | 2 | 0.0038 |
|  | *Chone* sp. | 110 | 1.4898 |
|  | *Diastylis quadrispinosa* | 2 | 0.0102 |
|  | *Edwardsia elegans* | 2 | 0.0628 |
|  | *Ericthonius fasciatus* | 170 | 0.2242 |
|  | *Euclymene zonalis* | 2 | 0.0018 |
|  | *Eudorella* sp. | 2 | 0.0018 |
|  | *Exogone* sp. | 6 | 0.0002 |
|  | *Galathowenia oculata* | 6 | 0.0196 |
|  | *Goniada maculata* | 6 | 0.086 |
|  | *Harmothoe imbricata* | 2 | 0.02 |
|  | *Lumbrineris fragilis* | 2 | 0.0648 |
|  | *Mediomastus ambiseta* | 2 | 0.0146 |
|  | *Melina* sp. | 12 | 0.1252 |
|  | Nemertea | 2 | 0.021 |
|  | *Nereis* sp. | 4 | 0.0098 |
|  | *Nothria conchylega* | 6 | 0.021 |
|  | *Notomastus latericeus* | 10 | 0.1736 |
|  | *Ophiura* sp. juvenile | 2 | 0.0002 |
|  | *Owenia fusiformis* | 12 | 0.0136 |
|  | *Paraonis* sp. | 2 | 0.0012 |
|  | *Parvicardium pinnulatum* | 16 | 0.1228 |
|  | *Phascolion strombus* | 8 | 0.0906 |
|  | *Polycirrus* sp. | 6 | 0.1674 |
|  | *Proclea graffi* | 2 | 0.0004 |
|  | *Puncturella noachina* | 4 | 0.0422 |
|  | Sabellidae | 98 | 0.047 |
|  | *Samytha sexicirrata* | 2 | 0.0066 |
|  | *Scolelepis* sp. A | 2 | 0.017 |
|  | *Scoloplos armiger* | 6 | 0.0568 |
|  | *Spio filicornis* | 8 | 0.006 |
|  | *Spiochaetopterus typicus* | 4 | 0.0414 |
|  | *Stenosemus albus* | 4 | 0.1002 |
|  | *Strongylocentrotus* sp. | 6 | 0.0094 |
|  | *Tachyrhynchus reticulatus* | 4 | 0.032 |
|  | *Terebellides stroemi* | 2 | 0.0072 |
|  | *Tharyx* sp. | 36 | 0.2438 |
|  | *Thyasira* sp. | 8 | 0.0586 |
| vg07 | Actiniaria (burrowing) | 2 | 0.0022 |
|  | *Ampharete finmarchica* | 30 | 0.0372 |
|  | *Amphilochus manudens* | 2 | 0.0006 |
|  | Amphiuridae | 40 | 0.0758 |
|  | *Anomia* sp. | 4 | 0.007 |
|  | *Arctica islandica* | 4 | 0.1862 |
|  | *Aricidea catherinae* | 14 | 0.0156 |
|  | *Aricidea* sp. B | 4 | 0.0072 |
|  | *Asabellides* sp. | 18 | 0.029 |
|  | Ascidiacea (solitary) | 26 | 0.088 |
|  | *Astarte* sp. | 28 | 33.2432 |
|  | *Axionice maculata* | 10 | 0.0952 |
|  | *Bathyarca pectunculoides* | 4 | 0.0568 |
|  | Capitellidae | 16 | 0.184 |
|  | Cerianthidae | 8 | 2.5638 |
|  | *Chone* sp. | 404 | 1.8694 |
|  | *Cucumaria frondosa* | 6 | 0.02 |
|  | *Cyclocardia novangliae* | 8 | 1.8708 |
|  | *Cyclocardia* sp. A | 2 | 0.0016 |
|  | *Cylichna alba* | 6 | 0.0176 |
|  | *Drilonereis magna* | 10 | 1.627 |
|  | *Dyopedos* sp. | 4 | 0.001 |
|  | *Ericthonius fasciatus* | 878 | 1.0944 |
|  | *Eteone longa* | 10 | 0.0878 |
|  | *Euclymene zonalis* | 304 | 1.3874 |
|  | *Eudendrium* sp. | 2 | 0.004 |
|  | *Eulalia bilineata* | 2 | 0.0048 |
|  | *Eunice pennata* | 24 | 2.0336 |
|  | *Eunoe nodosa* | 2 | 0.426 |
|  | *Exogone* sp. | 180 | 0.04 |
|  | *Galathowenia oculata* | 2 | 0.003 |
|  | Gastropoda | 52 | 0.05 |
|  | *Glycera capitata* | 36 | 0.7852 |
|  | *Gonactinia prolifera* | 4 | 0.0128 |
|  | *Harmothoe extenuata* | 2 | 0.0024 |
|  | *Harmothoe imbricata* | 2 | 0.0492 |
|  | *Harpinia plumosa* | 4 | 0.001 |
|  | *Janira alta* | 16 | 0.0502 |
|  | *Laonice cirrata* | 2 | 0.3056 |
|  | *Laphania boecki* | 2 | 0.0398 |
|  | *Leptostylis ampullacea* | 6 | 0.0044 |
|  | *Lumbrineris fragilis* | 8 | 4.147 |
|  | *Mactromeris polynyma* | 6 | 0.0048 |
|  | *Mediomastus ambiseta* | 2 | 0.0204 |
|  | *Melina* sp. | 6 | 0.0152 |
|  | *Melita dentata* | 14 | 0.0732 |
|  | *Modiolus modiolus* | 2 | 0.0022 |
|  | *Moelleria costulata* | 60 | 0.0536 |
|  | Mytiloidea | 4 | 0.0094 |
|  | *Myxicola infundibulum* | 4 | 1.3846 |
|  | Nemertea | 8 | 0.2518 |
|  | *Nothria conchylega* | 184 | 1.3098 |
|  | *Notomastus latericeus* | 38 | 2.1722 |
|  | Oligochaeta | 4 | 0.0146 |
|  | *Ophiopholis aculeata* | 2 | 0.005 |
|  | *Ophiura robusta* | 14 | 0.2766 |
|  | *Ophiura sarsi* | 12 | 0.2886 |
|  | Ostracoda | 2 | 0.0036 |
|  | *Paragytis margarete* | 4 | 0.0004 |
|  | *Paraonis* sp. | 8 | 0.0066 |
|  | *Parougia eliasoni* | 2 | 0.0004 |
|  | *Parvicardium pinnulatum* | 48 | 1.8086 |
|  | *Phascolion strombus* | 12 | 0.1994 |
|  | *Pherusa plumosa* | 2 | 0.0044 |
|  | *Pholoe tecta* | 6 | 0.0034 |
|  | *Phyllodoce groenlandica* | 4 | 0.0016 |
|  | *Placopecten magellanicus* | 2 | 0.1888 |
|  | *Pleurogonium spinosissimus* | 12 | 0.0052 |
|  | *Polycirrus* sp. | 26 | 0.4754 |
|  | *Polydora caulleryi* | 8 | 0.0162 |
|  | Polygordiidae | 2 | 0.0008 |
|  | *Prionospio cirrifera* | 54 | 0.0944 |
|  | *Proclea graffi* | 40 | 0.0562 |
|  | *Protodorvillea gaspeensis* | 4 | 0.0012 |
|  | *Pseudopotamilla* sp. | 6 | 0.0294 |
|  | *Psolus* sp. | 6 | 0.0128 |
|  | *Puncturella noachina* | 46 | 0.0694 |
|  | Questidae | 2 | 0.0004 |
|  | Sabellidae | 246 | 0.1792 |
|  | *Scalibregma inflatum* | 22 | 0.7534 |
|  | *Scolelepis* sp. A | 2 | 0.023 |
|  | Sipuncula | 38 | 0.0268 |
|  | *Sphaerodoropsis minuta* | 4 | 0.0022 |
|  | *Spio filicornis* | 2 | 0.0006 |
|  | *Spiochaetopterus typicus* | 2 | 0.1866 |
|  | *Stenopleustes inermis* | 12 | 0.0064 |
|  | *Stenosemus albus* | 10 | 0.1194 |
|  | *Strongylocentrotus* sp. | 92 | 0.584 |
|  | Syllidae | 2 | 0.0002 |
|  | *Tachyrhynchus reticulatus* | 4 | 0.0038 |
|  | *Terebellides stroemi* | 10 | 0.0856 |
|  | *Tharyx* sp. | 56 | 0.3864 |
|  | *Tiron spiniferus* | 14 | 0.0356 |
|  | *Unciola irrorata* | 70 | 0.2066 |
|  | *Westwoodilla brevicalar* | 2 | 0.0074 |
| vg08 | *Aglaophamus circinata* | 4 | 0.1452 |
|  | *Arctica islandica* | 4 | 0.0082 |
|  | *Aricidea catherinae* | 4 | 0.0028 |
|  | *Capitella capitata* | 2 | 0.0004 |
|  | *Chone* sp. | 6 | 1.2914 |
|  | *Edwardsia elegans* | 6 | 0.2494 |
|  | *Ericthonius fasciatus* | 86 | 0.1262 |
|  | *Euclymene zonalis* | 2 | 0.0026 |
|  | *Exogone* sp. | 12 | 0.003 |
|  | *Galathowenia oculata* | 4 | 0.0154 |
|  | *Glycera capitata* | 4 | 0.1838 |
|  | *Goniada maculata* | 6 | 0.0326 |
|  | *Lumbrineris fragilis* | 2 | 0.3952 |
|  | *Moelleria costulata* | 2 | 0.0042 |
|  | *Nereis* sp. | 2 | 0.0028 |
|  | *Nothria conchylega* | 2 | 0.0026 |
|  | *Notomastus latericeus* | 6 | 1.104 |
|  | *Ophiura* sp. juvenile | 2 | 0.0012 |
|  | *Orbinia swani* | 2 | 0.1178 |
|  | *Paraonis* sp. | 2 | 0.0022 |
|  | *Photis* sp. | 2 | 0.0018 |
|  | Sabellidae | 6 | 0.0072 |
|  | *Spio filicornis* | 8 | 0.0138 |
|  | *Strongylocentrotus* sp. | 4 | 0.0244 |
|  | *Tharyx* sp. | 16 | 0.9402 |
|  | *Thyasira* sp. | 4 | 0.192 |
|  | *Travisia* sp. | 2 | 0.006 |
|  | *Unciola irrorata* | 16 | 0.0606 |
| vg09 | Actiniaria (burrowing) | 2 | 0.0022 |
|  | *Aglaophamus circinata* | 4 | 0.038 |
|  | *Ampharete finmarchica* | 26 | 0.0542 |
|  | *Aricidea catherinae* | 8 | 0.0048 |
|  | *Aricidea* sp. B | 2 | 0.0004 |
|  | *Asabellides* sp. | 2 | 0.0036 |
|  | *Astarte* sp. | 2 | 0.0008 |
|  | *Axionice maculata* | 10 | 0.1392 |
|  | *Bathyarca pectunculoides* | 2 | 0.0196 |
|  | Cerianthidae | 2 | 0.5262 |
|  | *Chaetozone* sp. A | 2 | 0.001 |
|  | *Chone* sp. | 396 | 7.773 |
|  | *Cistenides granulata* | 4 | 0.1766 |
|  | *Cylichna alba* | 6 | 0.0262 |
|  | *Drilonereis magna* | 2 | 0.5286 |
|  | *Edwardsia elegans* | 2 | 0.002 |
|  | *Ericthonius fasciatus* | 480 | 0.5662 |
|  | *Euchone incolor* | 6 | 0.002 |
|  | *Euclymene zonalis* | 200 | 1.3628 |
|  | *Eunice pennata* | 4 | 0.416 |
|  | *Exogone* sp. | 52 | 0.0128 |
|  | *Galathowenia oculata* | 6 | 0.019 |
|  | Gastropoda | 10 | 0.0128 |
|  | *Glycera capitata* | 18 | 0.6506 |
|  | *Harmothoe imbricata* | 2 | 0.0088 |
|  | *Janira alta* | 6 | 0.0112 |
|  | *Leptostylis ampullacea* | 2 | 0.0038 |
|  | *Mediomastus ambiseta* | 8 | 0.0018 |
|  | *Melina* sp. | 6 | 0.051 |
|  | *Melita dentata* | 2 | 0.0954 |
|  | *Moelleria costulata* | 6 | 0.0038 |
|  | Mytiloidea | 2 | 0.0054 |
|  | *Myxicola infundibulum* | 2 | 0.096 |
|  | Nemertea | 4 | 0.014 |
|  | Nephtyidae sp. juvenile | 2 | 0.0002 |
|  | *Nothria conchylega* | 22 | 0.2344 |
|  | *Notomastus latericeus* | 10 | 0.0826 |
|  | Oligochaeta | 6 | 0.005 |
|  | *Ophiura* sp. juvenile | 12 | 0.0166 |
|  | *Orbinia swani* | 2 | 0.0002 |
|  | *Orchomenella minuta* | 4 | 0.0192 |
|  | Ostracoda | 2 | 0.002 |
|  | Pandalidae | 2 | 0.1972 |
|  | *Paraonis* sp. | 6 | 0.0018 |
|  | *Parvicardium pinnulatum* | 6 | 0.0332 |
|  | *Phascolion strombus* | 12 | 0.0946 |
|  | *Pleurogonium spinosissimus* | 8 | 0.0008 |
|  | *Polycirrus* sp. | 6 | 0.0768 |
|  | *Prionospio cirrifera* | 18 | 0.0306 |
|  | *Proclea graffi* | 16 | 0.0276 |
|  | *Protodorvillea gaspeensis* | 2 | 0.0006 |
|  | *Psolus* sp. | 2 | 0.006 |
|  | Questidae | 4 | 0.0032 |
|  | Sabellidae | 52 | 0.02 |
|  | *Scalibregma inflatum* | 18 | 0.36 |
|  | *Scolelepis* sp. A | 8 | 0.5296 |
|  | *Solariella obscura* | 2 | 0.008 |
|  | *Sphaerodoropsis minuta* | 2 | 0.0038 |
|  | *Spio filicornis* | 8 | 0.0018 |
|  | *Stenosemus albus* | 2 | 0.0002 |
|  | *Strongylocentrotus* sp. | 44 | 0.0486 |
|  | Syllidae | 4 | 0.0002 |
|  | *Terebellides stroemi* | 4 | 0.0228 |
|  | *Tharyx* sp. | 30 | 0.2152 |
|  | *Tiron spiniferus* | 6 | 0.0118 |
|  | *Unciola irrorata* | 98 | 0.4318 |
| vg10 | *Aglaophamus circinata* | 8 | 0.259 |
|  | *Ampharete finmarchica* | 4 | 0.0016 |
|  | *Aricidea catherinae* | 8 | 0.0068 |
|  | *Aricidea* sp. B | 34 | 0.023 |
|  | Ascidiacea (solitary) | 2 | 0.009 |
|  | *Axionice maculata* | 4 | 0.0096 |
|  | *Bathyarca pectunculoides* | 4 | 0.2548 |
|  | Bivalvia | 2 | 0.0004 |
|  | *Chaetozone* sp. A | 12 | 0.031 |
|  | *Chone* sp. | 16 | 0.0132 |
|  | *Clymenura borealis* | 18 | 3.781 |
|  | *Edwardsia elegans* | 4 | 0.0224 |
|  | *Ericthonius fasciatus* | 50 | 0.0412 |
|  | *Euclymene zonalis* | 144 | 0.8338 |
|  | *Euspira* sp. | 4 | 0.0268 |
|  | *Exogone* sp. | 106 | 0.0264 |
|  | *Galathowenia oculata* | 2 | 0.0006 |
|  | Gastropoda | 6 | 0.0352 |
|  | *Glycera capitata* | 46 | 0.5594 |
|  | *Levensinea gracilis* | 4 | 0.0008 |
|  | *Lumbrinerides acuta* | 2 | 0.0522 |
|  | *Melita dentata* | 2 | 0.001 |
|  | Nemertea | 2 | 0.0126 |
|  | *Nephtys caeca* | 2 | 1.3624 |
|  | *Nereis* sp. | 2 | 0.0014 |
|  | *Nothria conchylega* | 2 | 0.0004 |
|  | *Notomastus latericeus* | 6 | 0.3322 |
|  | Oligochaeta | 48 | 0.0104 |
|  | *Ophiura* sp. juvenile | 6 | 0.0008 |
|  | *Paraonis* sp. | 12 | 0.028 |
|  | *Periploma leanum* | 2 | 1.9142 |
|  | *Petalosarsia declivis* | 2 | 0.0008 |
|  | *Photis* sp. | 2 | 0.0008 |
|  | *Polycirrus* sp. | 12 | 0.0484 |
|  | Polygordiidae | 28 | 0.0092 |
|  | *Proclea graffi* | 4 | 0.0028 |
|  | *Protodorvillea kefersteini* | 30 | 0.0104 |
|  | Sabellidae | 12 | 0.0038 |
|  | *Scalibregma inflatum* | 2 | 0.3366 |
|  | *Spio filicornis* | 4 | 0.002 |
|  | *Spiochaetopterus typicus* | 2 | 0.5736 |
|  | Syllidae | 104 | 0.0098 |
|  | *Tharyx* sp. | 12 | 0.0256 |
|  | *Unciola irrorata* | 92 | 0.9654 |
| vg11 | Actiniaria (burrowing) | 2 | 0.0016 |
|  | *Aglaophamus circinata* | 12 | 0.1728 |
|  | *Arctica islandica* | 16 | 7.8616 |
|  | Ascidiacea (solitary) | 10 | 0.1272 |
|  | *Axionice maculata* | 6 | 0.0586 |
|  | *Chiridotea tuftsii* | 6 | 0.0362 |
|  | *Chone* sp. | 6 | 0.004 |
|  | *Clymenura borealis* | 50 | 13.2176 |
|  | *Cyclocardia novangliae* | 2 | 0.0074 |
|  | *Drilonereis magna* | 2 | 0.0404 |
|  | *Echinarachnius parma* | 2 | 0.1176 |
|  | *Ericthonius fasciatus* | 4 | 0.004 |
|  | *Euspira* sp. | 2 | 0.0034 |
|  | *Exogone* sp. | 8 | 0.001 |
|  | Gastropoda | 4 | 0.022 |
|  | *Glycera capitata* | 6 | 0.0792 |
|  | *Goniada maculata* | 6 | 0.1388 |
|  | *Hippomedon serratus* | 4 | 0.114 |
|  | *Liocyma fluctuosa* | 2 | 0.9026 |
|  | *Lumbrinerides acuta* | 4 | 0.0466 |
|  | *Melina* sp. | 2 | 0.005 |
|  | *Nereis* sp. | 2 | 0.0334 |
|  | *Nothria conchylega* | 2 | 0.017 |
|  | *Notomastus latericeus* | 8 | 0.3366 |
|  | Oligochaeta | 6 | 0.0066 |
|  | *Ophiura sarsi* | 2 | 0.0068 |
|  | *Owenia fusiformis* | 6 | 0.0256 |
|  | *Paraonis* sp. | 4 | 0.0014 |
|  | *Parvicardium pinnulatum* | 6 | 0.0758 |
|  | *Placopecten magellanicus* | 2 | 0.0122 |
|  | Sabellidae | 4 | 0.0004 |
|  | *Scalibregma inflatum* | 6 | 0.3056 |
|  | *Solariella obscura* | 2 | 0.017 |
|  | *Spio filicornis* | 2 | 0.0004 |
|  | *Spiochaetopterus typicus* | 2 | 0.0222 |
|  | *Spiophanes bombyx* | 2 | 0.0006 |
|  | *Thracia* sp. | 2 | 0.9714 |
|  | *Travisia* sp. | 2 | 0.0338 |
|  | *Unciola irrorata* | 46 | 0.5972 |
| vg12 | *Aglaophamus circinata* | 6 | 0.1802 |
|  | *Alvania* sp. | 6 | 0.0058 |
|  | *Ampharete finmarchica* | 4 | 0.0144 |
|  | *Arctica islandica* | 28 | 165.7574 |
|  | *Aricidea* sp. B | 4 | 0.0134 |
|  | *Aricidea wassi* | 2 | 0.003 |
|  | *Asabellides* sp. | 2 | 0.0022 |
|  | Ascidiacea (solitary) | 10 | 0.0802 |
|  | *Axionice maculata* | 10 | 0.0288 |
|  | *Chaetozone* sp. A | 6 | 0.0124 |
|  | *Chone* sp. | 4 | 0.0002 |
|  | *Clymenura borealis* | 18 | 6.6742 |
|  | *Diaphana minuta* | 2 | 0.0054 |
|  | *Ericthonius fasciatus* | 32 | 0.0576 |
|  | *Euspira* sp. | 2 | 0.004 |
|  | *Galathowenia oculata* | 12 | 0.0262 |
|  | Gastropoda | 2 | 0.018 |
|  | *Glycera capitata* | 4 | 0.0652 |
|  | *Goniada maculata* | 12 | 0.3504 |
|  | *Hippomedon serratus* | 10 | 0.3306 |
|  | *Lumbrinerides acuta* | 6 | 0.0628 |
|  | Nemertea | 4 | 0.0046 |
|  | Nephtyidae sp. juvenile | 4 | 0.0028 |
|  | *Nereis* sp. | 2 | 0.0518 |
|  | *Notomastus latericeus* | 8 | 1.214 |
|  | *Ophelina acuminata* | 2 | 0.002 |
|  | *Ophelina breviata* | 2 | 0.0018 |
|  | *Ophiura* sp. juvenile | 2 | 0.0002 |
|  | *Owenia fusiformis* | 16 | 0.0294 |
|  | *Periploma leanum* | 2 | 0.002 |
|  | *Polydora socialis* | 2 | 0.0016 |
|  | *Proclea graffi* | 2 | 0.0004 |
|  | Sabellidae | 8 | 0.0034 |
|  | *Scalibregma inflatum* | 2 | 0.2318 |
|  | *Scolelepis* sp. A | 2 | 0.0226 |
|  | *Solariella obscura* | 6 | 0.0856 |
|  | *Spio filicornis* | 4 | 0.0084 |
|  | *Spiochaetopterus typicus* | 14 | 0.459 |
|  | *Spiophanes bombyx* | 2 | 0.0056 |
|  | *Travisia* sp. | 10 | 0.1536 |
|  | *Unciola irrorata* | 22 | 0.2948 |
| vg13 | *Aglaophamus circinata* | 4 | 0.064 |
|  | *Alvania* sp. | 4 | 0.003 |
|  | *Ampharete finmarchica* | 12 | 0.0412 |
|  | *Aricidea catherinae* | 12 | 0.0066 |
|  | *Aricidea* sp. B | 84 | 0.0622 |
|  | Ascidiacea (solitary) | 4 | 0.0498 |
|  | *Axionice maculata* | 8 | 0.0058 |
|  | Bivalvia | 22 | 0.0932 |
|  | *Chaetozone* sp. | 4 | 0.0044 |
|  | *Chaetozone* sp. A | 16 | 0.0672 |
|  | *Chone* sp. | 80 | 0.0576 |
|  | *Cirolana polita* | 8 | 0.8948 |
|  | *Clymenura borealis* | 80 | 14.6742 |
|  | *Drilonereis magna* | 4 | 0.1026 |
|  | *Ericthonius fasciatus* | 26 | 0.0202 |
|  | *Euclymene zonalis* | 44 | 0.3798 |
|  | *Euspira* sp. | 2 | 0.0204 |
|  | *Exogone* sp. | 394 | 0.085 |
|  | *Glycera capitata* | 52 | 0.3074 |
|  | *Lumbrinerides acuta* | 100 | 1.3766 |
|  | Nephtyidae sp. juvenile | 8 | 0.002 |
|  | *Notomastus latericeus* | 2 | 0.3302 |
|  | Oligochaeta | 56 | 0.0568 |
|  | *Orbinia swani* | 2 | 0.174 |
|  | *Paraonis* sp. | 14 | 0.0092 |
|  | *Parvicardium pinnulatum* | 2 | 0.001 |
|  | *Phascolion strombus* | 4 | 0.0602 |
|  | *Pholoe tecta* | 4 | 0.072 |
|  | *Polycirrus* sp. | 14 | 0.1026 |
|  | *Polydora caulleryi* | 22 | 0.0474 |
|  | Polygordiidae | 40 | 0.0322 |
|  | *Prionospio cirrifera* | 4 | 0.0024 |
|  | *Proclea graffi* | 2 | 0.0014 |
|  | *Protodorvillea kefersteini* | 30 | 0.0092 |
|  | Questidae | 38 | 0.0052 |
|  | Sabellidae | 4 | 0.002 |
|  | *Scalibregma inflatum* | 6 | 0.2452 |
|  | Sipuncula | 2 | 0.001 |
|  | *Solariella obscura* | 2 | 0.0406 |
|  | *Spio filicornis* | 54 | 0.085 |
|  | *Spiochaetopterus typicus* | 2 | 0.4258 |
|  | *Spiophanes bombyx* | 2 | 0.0016 |
|  | Syllidae | 238 | 0.0262 |
|  | *Tharyx* sp. | 12 | 0.0504 |
|  | *Thracia* sp. | 10 | 0.4342 |
|  | *Tiron spiniferus* | 2 | 0.002 |
|  | *Unciola irrorata* | 158 | 1.8626 |
| vg14 | *Aglaophamus circinata* | 10 | 0.172 |
|  | *Alvania* sp. | 4 | 0.018 |
|  | *Arctica islandica* | 24 | 0.1098 |
|  | *Asabellides* sp. | 2 | 0.001 |
|  | Ascidiacea (solitary) | 2 | 0.0064 |
|  | *Axionice maculata* | 2 | 0.0752 |
|  | *Chaetozone* sp. A | 6 | 0.015 |
|  | *Chiridotea tuftsii* | 10 | 0.0348 |
|  | *Cirolana polita* | 10 | 0.2 |
|  | *Clymenura borealis* | 92 | 22.381 |
|  | *Dyopedos* sp. | 2 | 0.0012 |
|  | *Ericthonius fasciatus* | 10 | 0.0184 |
|  | *Exogone* sp. | 2 | 0.0004 |
|  | *Galathowenia oculata* | 2 | 0.0052 |
|  | Gastropoda | 4 | 0.018 |
|  | *Glycera capitata* | 2 | 0.0274 |
|  | *Goniada maculata* | 6 | 0.1488 |
|  | *Hippomedon serratus* | 6 | 0.15 |
|  | *Levensinea gracilis* | 2 | 0.0036 |
|  | *Lumbrinerides acuta* | 14 | 0.235 |
|  | Nemertea | 2 | 0.016 |
|  | *Notomastus latericeus* | 8 | 0.8328 |
|  | *Ophelina acuminata* | 2 | 0.0024 |
|  | *Ophelina breviata* | 2 | 0.0012 |
|  | *Ophiura sarsi* | 2 | 0.025 |
|  | *Ophiura* sp. juvenile | 4 | 0.0082 |
|  | *Owenia fusiformis* | 10 | 0.0172 |
|  | *Parvicardium pinnulatum* | 4 | 0.168 |
|  | *Placopecten magellanicus* | 2 | 0.0008 |
|  | *Proclea graffi* | 2 | 0.0016 |
|  | *Pseudounicola obliquua* | 2 | 0.0034 |
|  | Sabellidae | 2 | 0.0004 |
|  | *Scolelepis* sp. A | 2 | 0.0302 |
|  | *Scoloplos armiger* | 6 | 0.0796 |
|  | *Solariella obscura* | 2 | 0.017 |
|  | *Spio filicornis* | 2 | 0.0016 |
|  | *Spiochaetopterus typicus* | 6 | 0.5486 |
|  | Spionidae | 6 | 0.116 |
|  | *Travisia* sp. | 2 | 0.0108 |
|  | *Unciola irrorata* | 42 | 0.4726 |
| vg15 | Actiniaria (burrowing) | 6 | 0.5758 |
|  | *Alvania* sp. | 2 | 0.0018 |
|  | *Ampharete finmarchica* | 6 | 0.0216 |
|  | *Aricidea catherinae* | 6 | 0.006 |
|  | *Aricidea* sp. B | 28 | 0.0786 |
|  | *Astarte* sp. | 4 | 4.5092 |
|  | *Axionice maculata* | 20 | 0.1516 |
|  | Bivalvia | 2 | 0.0014 |
|  | Cerianthidae | 2 | 207.92 |
|  | *Chaetozone* sp. A | 4 | 0.0108 |
|  | *Chone* sp. | 78 | 0.0584 |
|  | *Clymenura borealis* | 26 | 7.0716 |
|  | *Drilonereis magna* | 6 | 0.2876 |
|  | *Edwardsia elegans* | 28 | 0.0134 |
|  | *Ericthonius fasciatus* | 122 | 0.1726 |
|  | *Eteone longa* | 4 | 0.0338 |
|  | *Euchone incolor* | 2 | 0.0002 |
|  | *Euclymene zonalis* | 230 | 2.623 |
|  | *Eunice pennata* | 4 | 0.9512 |
|  | *Exogone* sp. | 170 | 0.0372 |
|  | Gastropoda | 4 | 0.018 |
|  | *Glycera capitata* | 68 | 1.1042 |
|  | *Harmothoe imbricata* | 2 | 0.0234 |
|  | *Lumbrinerides acuta* | 16 | 0.375 |
|  | *Mediomastus ambiseta* | 2 | 0.0096 |
|  | *Myxicola infundibulum* | 2 | 2.24 |
|  | Nemertea | 6 | 0.0226 |
|  | *Neosabellides* sp. | 2 | 0.0004 |
|  | *Nereis* sp. | 4 | 0.247 |
|  | *Nothria conchylega* | 6 | 0.02 |
|  | Oligochaeta | 30 | 0.012 |
|  | *Ophelina breviata* | 2 | 0.0048 |
|  | *Ophiura* sp. juvenile | 2 | 0.0004 |
|  | *Parvicardium pinnulatum* | 10 | 0.6124 |
|  | *Phascolion strombus* | 2 | 0.0044 |
|  | *Photis* sp. | 4 | 0.011 |
|  | *Polycirrus* sp. | 8 | 0.13 |
|  | *Polydora caulleryi* | 30 | 0.1118 |
|  | Polygordiidae | 28 | 0.0466 |
|  | *Prionospio cirrifera* | 4 | 0.0048 |
|  | *Proclea graffi* | 14 | 0.0292 |
|  | *Protodorvillea kefersteini* | 6 | 0.0062 |
|  | *Pseudopotamilla* sp. | 2 | 0.0182 |
|  | *Psolus* sp. | 2 | 0.0002 |
|  | Questidae | 54 | 0.0142 |
|  | Sabellidae | 34 | 0.0184 |
|  | *Scalibregma inflatum* | 2 | 0.0006 |
|  | Sipuncula | 4 | 0.0012 |
|  | *Spio filicornis* | 14 | 0.0252 |
|  | *Spiochaetopterus typicus* | 4 | 2.4802 |
|  | Syllidae | 56 | 0.0046 |
|  | *Terebellides stroemi* | 2 | 0.0056 |
|  | *Tharyx* sp. | 16 | 0.1008 |
|  | *Thracia* sp. | 2 | 1.016 |
|  | *Tiron spiniferus* | 4 | 0.0094 |
|  | *Unciola irrorata* | 136 | 1.3436 |
| vg16 | Actiniaria (burrowing) | 2 | 0.0016 |
|  | *Aglaophamus circinata* | 2 | 0.032 |
|  | *Alvania* sp. | 2 | 0.001 |
|  | Amphiuridae | 2 | 0.0058 |
|  | *Aricidea catherinae* | 8 | 0.0286 |
|  | *Aricidea* sp. B | 10 | 0.0174 |
|  | *Axionice maculata* | 2 | 0.0018 |
|  | *Chaetozone setosa* | 2 | 0.0052 |
|  | *Chaetozone* sp. A | 2 | 0.0058 |
|  | *Chone* sp. | 20 | 0.0106 |
|  | *Clymenura borealis* | 2 | 0.239 |
|  | *Corymorpha pendula* | 2 | 0.0006 |
|  | *Cyclocardia novangliae* | 2 | 0.004 |
|  | *Ericthonius fasciatus* | 90 | 0.1166 |
|  | *Eteone longa* | 2 | 0.0224 |
|  | *Euclymene zonalis* | 44 | 1.0364 |
|  | *Exogone* sp. | 24 | 0.0054 |
|  | *Galathowenia oculata* | 2 | 0.0064 |
|  | Gastropoda | 4 | 0.032 |
|  | *Glycera capitata* | 22 | 0.1952 |
|  | *Lumbrinerides acuta* | 2 | 0.0738 |
|  | Nemertea | 6 | 0.0144 |
|  | Nephtyidae sp. juvenile | 2 | 0.0022 |
|  | *Nothria conchylega* | 4 | 0.028 |
|  | Oligochaeta | 8 | 0.0016 |
|  | *Paraonis* sp. | 4 | 0.0046 |
|  | *Parvicardium pinnulatum* | 2 | 0.045 |
|  | *Photis* sp. | 2 | 0.0022 |
|  | *Polycirrus* sp. | 4 | 0.0192 |
|  | *Polydora caulleryi* | 6 | 0.0114 |
|  | Polygordiidae | 14 | 0.017 |
|  | *Proclea graffi* | 2 | 0.0018 |
|  | Questidae | 6 | 0.0046 |
|  | Sabellidae | 10 | 0.003 |
|  | *Scalibregma inflatum* | 2 | 0.005 |
|  | *Solariella obscura* | 2 | 0.018 |
|  | *Spio filicornis* | 4 | 0.0048 |
|  | Syllidae | 20 | 0.0052 |
|  | *Thracia* sp. | 2 | 0.1552 |
|  | *Tiron spiniferus* | 4 | 0.0062 |
|  | *Unciola irrorata* | 44 | 0.311 |
| vg17 | *Aglaophamus circinata* | 6 | 0.0724 |
|  | *Ampharete finmarchica* | 8 | 0.0428 |
|  | *Aricidea* sp. B | 14 | 0.0094 |
|  | *Axionice maculata* | 24 | 0.175 |
|  | *Chaetozone* sp. A | 18 | 0.0788 |
|  | *Chone* sp. | 10 | 0.018 |
|  | *Cirolana polita* | 2 | 0.0222 |
|  | *Clymenura borealis* | 122 | 24.9274 |
|  | *Echinarachnius parma* | 4 | 0.0952 |
|  | *Edwardsia elegans* | 4 | 0.062 |
|  | *Ericthonius fasciatus* | 32 | 0.0428 |
|  | *Euclymene zonalis* | 22 | 0.121 |
|  | *Exogone* sp. | 66 | 0.0168 |
|  | Gastropoda | 6 | 0.0256 |
|  | *Glycera capitata* | 24 | 0.1926 |
|  | *Hippomedon serratus* | 2 | 0.0088 |
|  | *Lumbrinerides acuta* | 54 | 0.558 |
|  | *Mediomastus ambiseta* | 2 | 0.0012 |
|  | Nemertea | 2 | 0.0038 |
|  | *Notomastus latericeus* | 12 | 0.4396 |
|  | Oligochaeta | 22 | 0.0572 |
|  | *Orbinia swani* | 2 | 0.0946 |
|  | *Paraonis* sp. | 2 | 0.012 |
|  | *Polycirrus* sp. | 2 | 0.0068 |
|  | *Polydora caulleryi* | 4 | 0.1276 |
|  | Polygordiidae | 10 | 0.0038 |
|  | *Protodorvillea kefersteini* | 2 | 0.0006 |
|  | Questidae | 2 | 0.0004 |
|  | Sabellidae | 2 | 0.0004 |
|  | *Scalibregma inflatum* | 10 | 0.7388 |
|  | *Scolelepis* sp. A | 4 | 0.0092 |
|  | *Solariella obscura* | 2 | 0.0098 |
|  | *Spio filicornis* | 12 | 0.014 |
|  | Syllidae | 24 | 0.004 |
|  | *Unciola irrorata* | 24 | 0.1892 |
| vg18 | *Ampharete acutifrons* | 4 | 0.004 |
|  | *Ampharete finmarchica* | 2 | 0.0028 |
|  | Amphiuridae | 34 | 0.034 |
|  | *Arctica islandica* | 4 | 0.1272 |
|  | *Aricidea catherinae* | 52 | 0.057 |
|  | *Aricidea* sp. B | 34 | 0.046 |
|  | Ascidiacea (solitary) | 2 | 0.0296 |
|  | *Astarte* sp. | 24 | 1.1656 |
|  | *Axionice maculata* | 34 | 0.1806 |
|  | *Bylgides* sp. | 2 | 0.002 |
|  | Capitellidae | 4 | 0.0098 |
|  | *Chone* sp. | 296 | 0.3796 |
|  | *Cirolana polita* | 2 | 0.0344 |
|  | *Clymenura borealis* | 6 | 1.3774 |
|  | *Cyclocardia novangliae* | 20 | 0.135 |
|  | *Cylichna alba* | 2 | 0.0154 |
|  | *Edwardsia elegans* | 70 | 0.0352 |
|  | *Ericthonius fasciatus* | 474 | 0.2264 |
|  | *Eteone longa* | 12 | 0.0926 |
|  | *Euchone incolor* | 22 | 0.0044 |
|  | *Euclymene zonalis* | 468 | 2.7958 |
|  | *Exogone* sp. | 320 | 0.0378 |
|  | *Galathowenia oculata* | 2 | 0.0282 |
|  | Gastropoda | 12 | 0.06 |
|  | *Glycera capitata* | 154 | 3.0122 |
|  | *Harmothoe imbricata* | 6 | 0.0354 |
|  | *Lumbrinerides acuta* | 46 | 0.7774 |
|  | *Mediomastus ambiseta* | 2 | 0.0004 |
|  | *Modiolus modiolus* | 8 | 0.0016 |
|  | *Moelleria costulata* | 2 | 0.001 |
|  | Mytiloidea | 30 | 0.0486 |
|  | Nemertea | 10 | 0.0426 |
|  | Nephtyidae sp. juvenile | 2 | 0.0006 |
|  | *Nereis* sp. | 8 | 0.0208 |
|  | *Nothria conchylega* | 24 | 0.0638 |
|  | Oligochaeta | 30 | 0.0768 |
|  | *Ophelina breviata* | 2 | 0.0012 |
|  | *Ophiura sarsi* | 2 | 0.0486 |
|  | *Ophiura* sp. juvenile | 12 | 0.0062 |
|  | *Orbinia swani* | 4 | 0.0008 |
|  | Pandalidae | 4 | 0.0132 |
|  | *Paraonis* sp. | 24 | 0.0246 |
|  | *Parvicardium pinnulatum* | 24 | 0.0478 |
|  | *Phascolion strombus* | 6 | 0.2404 |
|  | *Phoxocephalus holbolli* | 2 | 0.0008 |
|  | *Polycirrus* sp. | 20 | 0.1918 |
|  | *Polydora caulleryi* | 124 | 0.3372 |
|  | Polygordiidae | 118 | 0.1486 |
|  | *Prionospio cirrifera* | 18 | 0.0056 |
|  | *Proclea graffi* | 18 | 0.0236 |
|  | *Protodorvillea kefersteini* | 8 | 0.0118 |
|  | *Puncturella noachina* | 4 | 0.001 |
|  | Questidae | 284 | 0.019 |
|  | Sabellidae | 88 | 0.026 |
|  | *Scalibregma inflatum* | 2 | 0.0066 |
|  | *Scolelepis* sp. A | 2 | 0.8934 |
|  | Sipuncula | 8 | 0.0018 |
|  | *Spio filicornis* | 10 | 0.0034 |
|  | *Stenopleustes inermis* | 6 | 0.002 |
|  | *Strongylocentrotus* sp. | 6 | 0.0042 |
|  | Syllidae | 120 | 0.0146 |
|  | *Terebellides stroemi* | 4 | 0.0064 |
|  | *Tharyx* sp. | 50 | 0.2204 |
|  | *Tiron spiniferus* | 8 | 0.0172 |
|  | *Unciola irrorata* | 64 | 0.3108 |
| vg19 | *Aglaophamus circinata* | 4 | 0.0394 |
|  | *Ampelisca agassizi* | 2 | 0.0048 |
|  | *Ampharete finmarchica* | 6 | 0.0138 |
|  | *Aricidea catherinae* | 8 | 0.0052 |
|  | *Aricidea* sp. B | 28 | 0.0124 |
|  | Ascidiacea (solitary) | 2 | 0.0666 |
|  | *Axionice maculata* | 2 | 0.0056 |
|  | Bivalvia | 20 | 0.0252 |
|  | *Chaetozone* sp. A | 22 | 0.1158 |
|  | *Chone* sp. | 18 | 0.0134 |
|  | *Clymenura borealis* | 22 | 4.4938 |
|  | *Diastylis sculpta* | 2 | 0.0032 |
|  | *Edwardsia elegans* | 8 | 0.0082 |
|  | *Ericthonius fasciatus* | 26 | 0.0282 |
|  | *Euclymene zonalis* | 38 | 0.3158 |
|  | *Exogone* sp. | 132 | 0.0216 |
|  | Gastropoda | 6 | 0.0286 |
|  | *Glycera capitata* | 34 | 0.3942 |
|  | *Lumbrinerides acuta* | 22 | 0.4094 |
|  | Mytiloidea | 2 | 0.0098 |
|  | Nephtyidae sp. juvenile | 4 | 0.001 |
|  | *Nereis* sp. | 6 | 0.0894 |
|  | *Nothria conchylega* | 2 | 0.0034 |
|  | *Notomastus latericeus* | 2 | 0.0318 |
|  | Oligochaeta | 26 | 0.0372 |
|  | Pandalidae | 22 | 0.0282 |
|  | *Paraonis* sp. | 2 | 0.0014 |
|  | *Parvicardium pinnulatum* | 2 | 0.002 |
|  | *Periploma leanum* | 2 | 0.3084 |
|  | *Placopecten magellanicus* | 2 | 0.0038 |
|  | *Polycirrus* sp. | 12 | 0.0486 |
|  | Polygordiidae | 56 | 0.035 |
|  | *Prionospio cirrifera* | 2 | 0.0034 |
|  | *Proclea graffi* | 4 | 0.003 |
|  | *Ptilanthura tenuis* | 1 | 0.0063 |
|  | Questidae | 14 | 0.002 |
|  | Sabellidae | 2 | 0.0018 |
|  | *Solariella obscura* | 8 | 0.4488 |
|  | *Spio filicornis* | 26 | 0.0296 |
|  | *Spiochaetopterus typicus* | 2 | 0.124 |
|  | Syllidae | 90 | 0.0078 |
|  | *Tharyx* sp. | 4 | 0.013 |
|  | *Tiron spiniferus* | 2 | 0.0174 |
|  | *Unciola irrorata* | 64 | 0.0802 |
| vg21 | *Ampharete finmarchica* | 2 | 0.0122 |
|  | *Arctica islandica* | 2 | 0.0694 |
|  | *Aricidea wassi* | 2 | 0.003 |
|  | Ascidiacea (solitary) | 54 | 0.292 |
|  | *Astarte* sp. | 2 | 4.4608 |
|  | Campanulariidae | 2 | 0.0014 |
|  | *Clymenura borealis* | 16 | 0.0424 |
|  | *Crangon septemspinosa* | 2 | 0.0104 |
|  | Cumacea sp. A | 4 | 0.0128 |
|  | *Cylichna alba* | 2 | 0.0004 |
|  | *Echinarachnius parma* | 4 | 0.0384 |
|  | *Edwardsia elegans* | 6 | 0.038 |
|  | *Ensis directus* | 4 | 0.844 |
|  | *Euspira* sp. | 4 | 0.1268 |
|  | *Goniadella gracilis* | 10 | 0.008 |
|  | *Hippomedon serratus* | 10 | 0.0172 |
|  | *Levensinea gracilis* | 16 | 0.0138 |
|  | *Lumbrinerides acuta* | 82 | 1.0206 |
|  | *Lumbrineris fragilis* | 2 | 0.0064 |
|  | *Nassarius* sp. | 4 | 0.2022 |
|  | Nemertea | 16 | 0.1398 |
|  | Nephtyidae sp. juvenile | 6 | 0.0332 |
|  | *Nephtys bucera* | 8 | 0.512 |
|  | *Nephtys caeca* | 4 | 2.9228 |
|  | *Nereis* sp. | 2 | 0.0216 |
|  | Oligochaeta | 10 | 0.002 |
|  | *Ophelia limacina* | 12 | 6.1306 |
|  | *Ophiopholis aculeata* | 2 | 0.0136 |
|  | *Ophiura robusta* | 2 | 0.002 |
|  | *Orbinia swani* | 8 | 0.1372 |
|  | *Paraonis* sp. | 10 | 0.0076 |
|  | *Parvicardium pinnulatum* | 4 | 0.002 |
|  | *Periploma leanum* | 2 | 0.0282 |
|  | *Pherusa plumosa* | 2 | 0.006 |
|  | *Phyllodoce mucosa* | 6 | 0.0064 |
|  | Platyhelminthes | 2 | 0.0422 |
|  | *Pleusymtes glaber* | 2 | 0.0026 |
|  | Polygordiidae | 8 | 0.0018 |
|  | *Protomedeia fasciata* | 2 | 0.0128 |
|  | *Psammonyx* sp. | 2 | 0.0482 |
|  | *Scoloplos armiger* | 6 | 0.081 |
|  | *Spisula solidissima* | 4 | 5.3022 |
|  | Syllidae | 6 | 0.0006 |
|  | *Tharyx* sp. | 10 | 0.0124 |
| vg22 | *Acanthodoris pilosa* | 2 | 0.0154 |
|  | *Achelia* sp. | 6 | 0.0176 |
|  | *Aeginina longicornis* | 4 | 0.0022 |
|  | *Ampharete finmarchica* | 34 | 0.0932 |
|  | *Arctica islandica* | 4 | 0.0538 |
|  | *Aricidea catherinae* | 8 | 0.0048 |
|  | *Aricidea* sp. A | 4 | 0.0036 |
|  | Ascidiacea (solitary) | 4 | 0.0266 |
|  | *Astarte* sp. | 2 | 0.0342 |
|  | Asteroidea | 6 | 0.0082 |
|  | *Autolytus* sp. | 4 | 0.0024 |
|  | Campanulariidae | 2 | 0.0006 |
|  | *Cancer borealis* | 4 | 0.025 |
|  | *Caprella* sp. | 16 | 0.0192 |
|  | Cerianthidae | 4 | 9.2918 |
|  | *Chaetozone* sp. A | 18 | 0.0284 |
|  | *Cistenides granulata* | 4 | 0.0126 |
|  | *Clymenura borealis* | 2 | 0.0308 |
|  | *Crangon septemspinosa* | 14 | 0.258 |
|  | *Cucumaria frondosa* | 6 | 241.6746 |
|  | Cumacea sp. A | 2 | 0.0018 |
|  | *Dendrobeania* sp. | 2 | 0.0994 |
|  | *Dyopedos* sp. | 2 | 0.0016 |
|  | *Echinarachnius parma* | 12 | 0.0046 |
|  | *Edotea montosa* | 2 | 0.0048 |
|  | *Edwardsia elegans* | 368 | 0.9884 |
|  | *Euchone papillosa* | 2 | 0.0034 |
|  | *Euclymene zonalis* | 448 | 6.0508 |
|  | *Eucratea loricata* | 2 | 0.0318 |
|  | *Euspira* sp. | 4 | 0.0916 |
|  | *Exogone* sp. | 2 | 0.0002 |
|  | Gastropoda | 2 | 0.0042 |
|  | *Glycera capitata* | 6 | 0.03 |
|  | *Goniadella gracilis* | 34 | 0.0382 |
|  | *Harmothoe extenuata* | 20 | 0.0516 |
|  | *Ischyrocerus* sp. | 10 | 0.0186 |
|  | *Lamprops* sp. | 2 | 0.0008 |
|  | *Leptognathia* sp. | 4 | 0.001 |
|  | *Levensinea gracilis* | 4 | 0.0044 |
|  | *Lumbrinerides acuta* | 70 | 0.567 |
|  | *Lumbrineris fragilis* | 26 | 1.6092 |
|  | *Modiolus modiolus* | 8 | 0.005 |
|  | *Monoculodes* sp. | 2 | 0.0106 |
|  | Mytiloidea | 24 | 1.717 |
|  | *Nassarius* sp. | 2 | 0.5792 |
|  | Nemertea | 32 | 1.8634 |
|  | *Nephtys bucera* | 4 | 0.1416 |
|  | *Nephtys caeca* | 10 | 1.3124 |
|  | *Nereis* sp. | 56 | 0.5624 |
|  | *Nicolea venusula* | 38 | 0.0276 |
|  | Nudibranchia Group 3 | 2 | 0.0194 |
|  | *Ophelia limacina* | 4 | 0.0952 |
|  | *Ophiopholis aculeata* | 20 | 0.084 |
|  | *Pagurus* sp. | 6 | 0.0234 |
|  | *Paraonis* sp. | 52 | 0.0304 |
|  | *Parougia eliasoni* | 4 | 0.0048 |
|  | *Parvicardium pinnulatum* | 20 | 0.0182 |
|  | *Periploma leanum* | 2 | 0.0108 |
|  | *Pherusa plumosa* | 4 | 0.008 |
|  | *Pholoe tecta* | 2 | 0.0012 |
|  | *Photis* sp. | 8 | 0.0354 |
|  | *Phoxocephalus holbolli* | 2 | 0.0032 |
|  | *Phyllodoce maculata* | 4 | 0.0002 |
|  | *Phyllodoce mucosa* | 2 | 0.0006 |
|  | *Pleusymtes glaber* | 26 | 0.016 |
|  | Polygordiidae | 102 | 0.0392 |
|  | *Protodorvillea kefersteini* | 2 | 0.0002 |
|  | *Protomedeia fasciata* | 18 | 0.0118 |
|  | *Psolus* sp. | 12 | 0.0172 |
|  | *Scolelepis* sp. A | 2 | 0.0222 |
|  | *Scolelepis squamata* | 24 | 0.043 |
|  | *Sertularia mirabilis* | 2 | 0.0076 |
|  | *Sertularia* sp. | 2 | 0.002 |
|  | *Spio filicornis* | 40 | 0.2074 |
|  | *Spiophanes bombyx* | 4 | 0.0052 |
|  | *Strongylocentrotus* sp. | 4 | 0.001 |
|  | Syllidae | 2 | 0.0002 |
|  | *Tharyx* sp. | 58 | 0.3772 |
|  | *Unciola irrorata* | 132 | 0.9332 |
| vg23 | *Aglaophamus circinata* | 2 | 0.1388 |
|  | *Aricidea catherinae* | 6 | 0.0106 |
|  | *Aricidea wassi* | 18 | 0.0244 |
|  | Ascidiacea (solitary) | 6 | 0.0178 |
|  | *Chaetozone setosa* | 2 | 0.0024 |
|  | *Chaetozone* sp. A | 2 | 0.0032 |
|  | *Cylichna alba* | 2 | 0.0018 |
|  | *Cyrtodaria siliqua* | 2 | 0.003 |
|  | *Echinarachnius parma* | 6 | 0.002 |
|  | *Edwardsia elegans* | 4 | 0.0142 |
|  | *Ensis directus* | 6 | 0.0016 |
|  | *Euspira* sp. | 2 | 0.0194 |
|  | *Exogone* sp. | 4 | 0.0054 |
|  | *Levensinea gracilis* | 6 | 0.0036 |
|  | *Lumbrinerides acuta* | 54 | 0.6926 |
|  | *Lumbrineris fragilis* | 2 | 1.469 |
|  | Nemertea | 14 | 0.3974 |
|  | *Nephtys bucera* | 8 | 0.209 |
|  | *Nephtys caeca* | 2 | 14.164 |
|  | Oligochaeta | 2 | 0.0002 |
|  | *Ophelia limacina* | 18 | 2.92 |
|  | *Paraonis* sp. | 2 | 0.0004 |
|  | Polygordiidae | 4 | 0.0002 |
|  | *Scolelepis squamata* | 2 | 0.0018 |
|  | *Scoloplos armiger* | 8 | 0.0454 |
|  | *Spio filicornis* | 4 | 0.0242 |
|  | *Spiophanes bombyx* | 2 | 0.0008 |
|  | *Tharyx* sp. | 16 | 0.0946 |
| vg24 | *Aglaophamus circinata* | 6 | 0.4728 |
|  | *Ampharete finmarchica* | 32 | 0.022 |
|  | *Aricidea catherinae* | 6 | 0.0114 |
|  | Ascidiacea (solitary) | 18 | 0.0428 |
|  | *Bathyporeia quoddyensis* | 2 | 0.0002 |
|  | Cerianthidae | 16 | 10.8084 |
|  | *Chaetozone* sp. A | 12 | 0.0186 |
|  | *Cistenides granulata* | 6 | 0.0072 |
|  | *Clymenura borealis* | 2 | 0.0002 |
|  | Cumacea sp. A | 6 | 0.0122 |
|  | *Cyrtodaria siliqua* | 4 | 0.006 |
|  | *Dendrobeania* sp. | 2 | 0.0256 |
|  | *Echinarachnius parma* | 50 | 0.0162 |
|  | *Edwardsia elegans* | 32 | 0.0512 |
|  | *Ensis directus* | 10 | 0.0016 |
|  | *Euchone papillosa* | 2 | 0.0048 |
|  | *Euclymene zonalis* | 26 | 0.0668 |
|  | *Eucratea loricata* | 2 | 0.0024 |
|  | *Euspira* sp. | 4 | 0.0384 |
|  | Gastropoda | 2 | 0.0018 |
|  | *Goniadella gracilis* | 10 | 0.0182 |
|  | *Levensinea gracilis* | 2 | 0.001 |
|  | *Lumbrinerides acuta* | 128 | 1.513 |
|  | *Lumbrineris fragilis* | 12 | 0.1162 |
|  | Mytiloidea | 10 | 0.076 |
|  | *Nassarius* sp. | 4 | 0.026 |
|  | Nemertea | 14 | 0.584 |
|  | *Neosabellides* sp. | 8 | 0.006 |
|  | *Nephtys bucera* | 4 | 0.048 |
|  | *Nephtys caeca* | 2 | 0.014 |
|  | *Nereis* sp. | 46 | 0.218 |
|  | Oligochaeta | 4 | 0.0076 |
|  | *Ophelia limacina* | 6 | 0.0076 |
|  | *Orbinia swani* | 6 | 0.4574 |
|  | *Paraonis* sp. | 22 | 0.0096 |
|  | *Parougia eliasoni* | 2 | 0.0002 |
|  | *Parvicardium pinnulatum* | 4 | 0.0242 |
|  | *Photis* sp. | 2 | 0.0002 |
|  | *Phyllodoce groenlandica* | 2 | 0.009 |
|  | *Phyllodoce mucosa* | 4 | 0.0006 |
|  | *Polycirrus* sp. | 4 | 0.0164 |
|  | Polygordiidae | 76 | 0.021 |
|  | *Protomedeia fasciata* | 2 | 0.0026 |
|  | *Pseudounicola obliquua* | 4 | 0.0032 |
|  | *Scolelepis squamata* | 16 | 0.0174 |
|  | *Scoloplos armiger* | 2 | 0.007 |
|  | *Solariella obscura* | 2 | 0.0324 |
|  | *Spio filicornis* | 4 | 0.014 |
|  | *Spiophanes bombyx* | 14 | 0.0016 |
|  | *Tharyx* sp. | 26 | 0.0752 |
|  | *Thracia* sp. | 2 | 0.0118 |
|  | *Unciola irrorata* | 56 | 0.2206 |
| vg25 | Actiniaria (burrowing) | 10 | 0.8734 |
|  | Actiniaria (non burrowing) | 4 | 0.0032 |
|  | *Ampharete finmarchica* | 194 | 0.1388 |
|  | *Aricidea catherinae* | 2 | 0.0012 |
|  | *Aricidea* sp. B | 2 | 0.0056 |
|  | Ascidiacea (solitary) | 10 | 0.022 |
|  | Asteroidea | 6 | 0.0054 |
|  | *Autolytus* sp. | 14 | 0.0034 |
|  | Campanulariidae | 2 | 0.0782 |
|  | *Cancer borealis* | 8 | 0.0564 |
|  | *Caprella* sp. | 4 | 0.0056 |
|  | *Chaetozone* sp. A | 10 | 0.0218 |
|  | *Chone* sp. | 8 | 0.0022 |
|  | *Cirolana polita* | 2 | 0.028 |
|  | *Clymenura borealis* | 10 | 0.049 |
|  | *Cucumaria frondosa* | 200 | 1.477 |
|  | *Dyopedos* sp. | 2 | 0.0016 |
|  | *Echinarachnius parma* | 10 | 0.0006 |
|  | *Edwardsia elegans* | 98 | 0.54 |
|  | *Eteone longa* | 2 | 0.0054 |
|  | *Euclymene zonalis* | 4064 | 42.8222 |
|  | *Eucratea loricata* | 2 | 0.0092 |
|  | *Exogone* sp. | 2 | 0.0002 |
|  | *Gersemia rubrifornis* | 2 | 0.0412 |
|  | *Glycera capitata* | 18 | 0.9582 |
|  | *Glycera dibranchiata* | 2 | 0.0892 |
|  | *Goniadella gracilis* | 1382 | 4.5304 |
|  | *Harmothoe extenuata* | 82 | 0.202 |
|  | *Harmothoe imbricata* | 2 | 0.0256 |
|  | *Hydrozoa athecata* | 2 | 0.0002 |
|  | *Ischyrocerus* sp. | 62 | 0.0212 |
|  | *Leptognathia* sp. | 8 | 0.0022 |
|  | *Lumbrinerides acuta* | 160 | 1.7574 |
|  | *Lumbrineris fragilis* | 56 | 1.47 |
|  | *Modiolus modiolus* | 2 | 0.0002 |
|  | *Mysta barbata* | 2 | 0.0012 |
|  | Mytiloidea | 22 | 0.3472 |
|  | Nemertea | 350 | 1.175 |
|  | *Neosabellides* sp. | 2 | 0.0028 |
|  | *Nephtys caeca* | 2 | 5.7214 |
|  | *Nereis* sp. | 76 | 0.7804 |
|  | *Nicolea venusula* | 16 | 0.0048 |
|  | Oligochaeta | 158 | 0.1826 |
|  | *Ophelia limacina* | 12 | 0.1406 |
|  | *Ophiopholis aculeata* | 2 | 0.0072 |
|  | *Orbinia swani* | 4 | 0.0058 |
|  | *Pagurus* sp. | 2 | 0.0064 |
|  | *Paraonis* sp. | 94 | 0.073 |
|  | *Parougia eliasoni* | 8 | 0.0034 |
|  | *Parvicardium pinnulatum* | 6 | 0.0016 |
|  | *Pherusa plumosa* | 52 | 0.019 |
|  | *Pholoe minuta* | 6 | 0.001 |
|  | *Pholoe tecta* | 74 | 0.0084 |
|  | *Photis* sp. | 4 | 0.0008 |
|  | *Phoxocephalus holbolli* | 2 | 0.0002 |
|  | *Phyllodoce groenlandica* | 2 | 0.0042 |
|  | *Phyllodoce maculata* | 10 | 0.0352 |
|  | *Phyllodoce mucosa* | 54 | 0.0296 |
|  | *Pleusymtes glaber* | 106 | 0.0558 |
|  | Polygordiidae | 192 | 0.2282 |
|  | *Pontogeneia inermis* | 2 | 0.0014 |
|  | *Protodorvillea kefersteini* | 92 | 0.029 |
|  | *Protomedeia fasciata* | 32 | 0.02 |
|  | *Psolus* sp. | 24 | 0.0404 |
|  | Questidae | 20 | 0.0018 |
|  | *Scolelepis* sp. A | 2 | 1.5298 |
|  | *Scolelepis squamata* | 2 | 0.0068 |
|  | *Sertularia* sp. | 2 | 0.0064 |
|  | *Spio filicornis* | 2 | 0.009 |
|  | *Spiophanes bombyx* | 2 | 0.0014 |
|  | Stenothoidae | 2 | 0.0002 |
|  | *Strongylocentrotus* sp. | 18 | 0.0334 |
|  | Syllidae | 10 | 0.0036 |
|  | *Tharyx* sp. | 10 | 0.0154 |
|  | *Unciola irrorata* | 46 | 0.0788 |
| vg26 | *Achelia* sp. | 4 | 0.0024 |
|  | *Aeginina longicornis* | 2 | 0.0028 |
|  | *Ampharete finmarchica* | 180 | 0.155 |
|  | *Arcteobia anticostiensis* | 2 | 0.0024 |
|  | *Arctica islandica* | 2 | 0.0068 |
|  | *Argissa hamatipes* | 2 | 0.0004 |
|  | *Aricidea catherinae* | 14 | 0.0126 |
|  | *Aricidea* sp. A | 2 | 0.0058 |
|  | Ascidiacea (solitary) | 10 | 0.0192 |
|  | Asteroidea | 4 | 0.0062 |
|  | *Autolytus* sp. | 2 | 0.0004 |
|  | Campanulariidae | 2 | 2.1492 |
|  | Capitellidae | 2 | 0.002 |
|  | *Chaetozone* sp. A | 18 | 0.0868 |
|  | *Clymenura borealis* | 6 | 0.265 |
|  | *Crangon septemspinosa* | 10 | 0.0774 |
|  | *Cucumaria frondosa* | 74 | 0.4578 |
|  | *Dendrobeania* sp. | 2 | 0.003 |
|  | *Echinarachnius parma* | 8 | 0.0014 |
|  | *Edwardsia elegans* | 58 | 0.215 |
|  | *Ericthonius rubricornis* | 2 | 0.0002 |
|  | *Eteone longa* | 2 | 0.0118 |
|  | *Euchone papillosa* | 2 | 0.0006 |
|  | *Euclymene zonalis* | 846 | 25.8962 |
|  | *Eucratea loricata* | 2 | 0.0006 |
|  | *Euspira* sp. | 2 | 0.0318 |
|  | *Exogone* sp. | 2 | 0.0002 |
|  | *Glycera capitata* | 10 | 0.735 |
|  | *Glycera dibranchiata* | 2 | 0.1772 |
|  | *Goniadella gracilis* | 718 | 2.3312 |
|  | *Harmothoe extenuata* | 22 | 0.071 |
|  | *Harmothoe imbricata* | 2 | 0.0116 |
|  | *Ischyrocerus* sp. | 42 | 0.0086 |
|  | *Lumbrinerides acuta* | 52 | 0.6886 |
|  | *Lumbrineris fragilis* | 54 | 6.6648 |
|  | *Modiolus modiolus* | 2 | 0.0008 |
|  | Mytiloidea | 8 | 0.2416 |
|  | Nemertea | 210 | 0.3682 |
|  | *Nephtys bucera* | 8 | 0.188 |
|  | *Nereis* sp. | 78 | 1.1966 |
|  | *Nicolea venusula* | 8 | 0.0036 |
|  | Oligochaeta | 64 | 0.0734 |
|  | *Ophelia limacina* | 4 | 0.0002 |
|  | *Orbinia swani* | 2 | 0.0438 |
|  | *Paraonis* sp. | 32 | 0.0314 |
|  | *Parougia eliasoni* | 6 | 0.0018 |
|  | *Parvicardium pinnulatum* | 6 | 0.0052 |
|  | *Pherusa plumosa* | 20 | 0.0102 |
|  | *Pholoe tecta* | 42 | 0.0136 |
|  | *Photis* sp. | 2 | 0.0002 |
|  | *Phyllodoce mucosa* | 4 | 0.003 |
|  | Platyhelminthes | 2 | 0.037 |
|  | *Pleusymtes glaber* | 24 | 0.014 |
|  | *Polycirrus* sp. | 12 | 0.4086 |
|  | Polygordiidae | 230 | 0.1584 |
|  | *Pontogeneia inermis* | 2 | 0.0016 |
|  | *Protodorvillea kefersteini* | 10 | 0.0048 |
|  | *Protomedeia fasciata* | 8 | 0.0072 |
|  | *Psolus* sp. | 46 | 0.0648 |
|  | Questidae | 2 | 0.0004 |
|  | *Scolelepis squamata* | 10 | 0.0096 |
|  | *Sertularia* sp. | 2 | 0.001 |
|  | *Spio filicornis* | 24 | 0.1184 |
|  | *Spiophanes bombyx* | 2 | 0.0002 |
|  | *Strongylocentrotus* sp. | 18 | 0.025 |
|  | Syllidae | 12 | 0.002 |
|  | *Tharyx* sp. | 42 | 0.0878 |
|  | *Thuiaria* sp. | 2 | 0.0198 |
|  | *Unciola irrorata* | 170 | 0.6034 |
| vg27 | *Ampharete finmarchica* | 2 | 0.0292 |
|  | Ascidiacea (solitary) | 12 | 0.04 |
|  | *Chaetozone setosa* | 2 | 0.0002 |
|  | *Chaetozone* sp. A | 2 | 0.0032 |
|  | *Clymenura borealis* | 18 | 0.4524 |
|  | *Cucumaria frondosa* | 2 | 0.0524 |
|  | *Echinarachnius parma* | 2 | 0.0002 |
|  | *Edwardsia elegans* | 2 | 0.0098 |
|  | *Ensis directus* | 4 | 0.6496 |
|  | *Euspira* sp. | 4 | 0.1198 |
|  | *Glycera dibranchiata* | 2 | 0.1296 |
|  | *Lumbrinerides acuta* | 20 | 0.2404 |
|  | *Lumbrineris fragilis* | 2 | 3.802 |
|  | *Nassarius* sp. | 2 | 0.5104 |
|  | Nemertea | 6 | 0.1656 |
|  | *Nephtys bucera* | 8 | 0.592 |
|  | *Nephtys caeca* | 2 | 1.7158 |
|  | *Ophelia limacina* | 4 | 0.0504 |
|  | *Orbinia swani* | 4 | 0.0528 |
|  | *Periploma leanum* | 2 | 0.0078 |
|  | Polygordiidae | 2 | 0.0286 |
|  | *Psammonyx* sp. | 2 | 0.003 |
|  | *Scolelepis squamata* | 6 | 0.007 |
|  | *Scoloplos armiger* | 32 | 0.2648 |
|  | *Spiophanes bombyx* | 8 | 0.0224 |
|  | *Tharyx* sp. | 2 | 0.0274 |
| vg28 | *Aricidea catherinae* | 2 | 0.0022 |
|  | *Aricidea wassi* | 30 | 0.0878 |
|  | Ascidiacea (solitary) | 94 | 0.2922 |
|  | *Astarte* sp. | 2 | 0.1162 |
|  | *Cirolana polita* | 4 | 0.1568 |
|  | *Clymenura borealis* | 2 | 0.07 |
|  | *Echinarachnius parma* | 6 | 0.002 |
|  | *Edwardsia elegans* | 4 | 0.03 |
|  | *Ensis directus* | 8 | 97.4138 |
|  | *Euspira* sp. | 2 | 0.0242 |
|  | *Glycera dibranchiata* | 4 | 0.2296 |
|  | *Lumbrinerides acuta* | 54 | 0.6608 |
|  | *Nassarius* sp. | 2 | 0.0452 |
|  | Nemertea | 18 | 0.3398 |
|  | *Nephtys bucera* | 10 | 0.528 |
|  | Oligochaeta | 2 | 0.0002 |
|  | *Ophelia limacina* | 4 | 0.683 |
|  | *Periploma leanum* | 2 | 0.0296 |
|  | *Scoloplos armiger* | 32 | 0.1986 |
|  | *Spiophanes bombyx* | 4 | 0.0012 |
|  | *Tharyx* sp. | 2 | 0.0056 |
| vg29 | *Ampharete finmarchica* | 18 | 0.026 |
|  | *Aricidea* sp. B | 12 | 0.0088 |
|  | *Aricidea wassi* | 2 | 0.005 |
|  | Ascidiacea (solitary) | 2 | 0.0422 |
|  | *Cancer borealis* | 2 | 0.008 |
|  | *Echinarachnius parma* | 2 | 0.0002 |
|  | *Ensis directus* | 2 | 0.004 |
|  | *Euclymene zonalis* | 22 | 0.3042 |
|  | *Euspira* sp. | 4 | 0.06 |
|  | *Exogone* sp. | 2 | 0.0006 |
|  | *Glycera dibranchiata* | 2 | 0.1224 |
|  | *Goniadella gracilis* | 152 | 0.1736 |
|  | *Hippomedon serratus* | 2 | 0.0342 |
|  | *Leptognathia* sp. | 2 | 0.0002 |
|  | *Lumbrinerides acuta* | 16 | 0.7298 |
|  | *Lumbrineris fragilis* | 6 | 0.1094 |
|  | *Nassarius* sp. | 6 | 0.544 |
|  | Nemertea | 42 | 0.4806 |
|  | *Nephtys bucera* | 18 | 0.5246 |
|  | *Nephtys caeca* | 2 | 2.3524 |
|  | *Nereis* sp. | 4 | 0.008 |
|  | Oligochaeta | 24 | 0.0038 |
|  | *Ophelia limacina* | 30 | 0.0218 |
|  | *Paraonis* sp. | 12 | 0.0058 |
|  | *Periploma leanum* | 2 | 0.1382 |
|  | Polygordiidae | 180 | 0.0648 |
|  | *Protodorvillea kefersteini* | 58 | 0.0038 |
|  | Questidae | 70 | 0.0188 |
|  | *Scolelepis squamata* | 60 | 0.1878 |
|  | Syllidae | 12 | 0.0044 |
|  | *Tharyx* sp. | 12 | 0.0274 |
|  | *Unciola irrorata* | 4 | 0.0216 |
| vg30 | *Ampharete finmarchica* | 4 | 0.0038 |
|  | Ascidiacea (solitary) | 44 | 0.1722 |
|  | *Bathyporeia quoddyensis* | 2 | 0.004 |
|  | *Cirolana polita* | 2 | 0.0216 |
|  | *Clymenura borealis* | 8 | 0.0082 |
|  | *Cylichna alba* | 2 | 0.0552 |
|  | *Echinarachnius parma* | 16 | 0.004 |
|  | *Edwardsia elegans* | 2 | 0.0102 |
|  | *Ensis directus* | 2 | 0.0016 |
|  | *Euspira* sp. | 6 | 0.1184 |
|  | *Glycera dibranchiata* | 2 | 0.0116 |
|  | *Lumbrinerides acuta* | 10 | 0.2154 |
|  | *Lumbrineris fragilis* | 2 | 0.0038 |
|  | Mytiloidea | 2 | 0.003 |
|  | *Nassarius* sp. | 2 | 0.0888 |
|  | Nemertea | 16 | 0.15 |
|  | *Nephtys bucera* | 4 | 0.0384 |
|  | *Nereis* sp. | 2 | 0.0004 |
|  | *Ophelia limacina* | 4 | 0.0094 |
|  | *Paraonis* sp. | 2 | 0.004 |
|  | *Phyllodoce mucosa* | 4 | 0.0016 |
|  | Polygordiidae | 4 | 0.0002 |
|  | *Scoloplos armiger* | 8 | 0.0582 |
|  | *Spiophanes bombyx* | 10 | 0.0182 |
|  | *Unciola irrorata* | 2 | 0.006 |
| vg31 | Actiniaria (burrowing) | 24 | 2.7866 |
|  | *Aeginina longicornis* | 2 | 0.0024 |
|  | *Ampharete finmarchica* | 120 | 0.4316 |
|  | *Anonyx sarsi* | 8 | 0.027 |
|  | *Arctica islandica* | 10 | 1.2876 |
|  | *Aricidea catherinae* | 20 | 0.0112 |
|  | Ascidiacea (solitary) | 52 | 2.456 |
|  | *Autolytus* sp. | 4 | 0.0016 |
|  | *Balanus* sp. | 2 | 0.004 |
|  | *Boreocingula* sp. | 4 | 0.0536 |
|  | Campanulariidae | 2 | 0.0432 |
|  | Cerianthidae | 12 | 11.2718 |
|  | *Chaetozone* sp. A | 2 | 0.0042 |
|  | *Chone* sp. | 56 | 0.9466 |
|  | *Cistenides granulata* | 16 | 0.0214 |
|  | *Clymenura borealis* | 54 | 7.581 |
|  | *Colus* sp. | 2 | 0.0264 |
|  | *Crangon septemspinosa* | 6 | 0.0662 |
|  | *Cucumaria frondosa* | 192 | 0.3736 |
|  | *Cylichna alba* | 10 | 0.0182 |
|  | *Cyrtodaria siliqua* | 448 | 648.7996 |
|  | *Drilonereis magna* | 6 | 0.0602 |
|  | *Dyopedos* sp. | 2 | 0.0038 |
|  | *Echinarachnius parma* | 14 | 0.0152 |
|  | *Edwardsia elegans* | 76 | 3.0804 |
|  | *Eteone longa* | 2 | 0.001 |
|  | *Euchone papillosa* | 56 | 0.264 |
|  | *Euclymene zonalis* | 2596 | 42.6048 |
|  | *Eucratea loricata* | 2 | 0.0002 |
|  | *Eulalia bilineata* | 6 | 0.0046 |
|  | *Exogone* sp. | 286 | 0.0758 |
|  | Gastropoda | 4 | 0.0092 |
|  | *Glycera capitata* | 324 | 6.248 |
|  | *Haploops* sp. | 2 | 0.0016 |
|  | *Ischyrocerus* sp. | 280 | 0.1266 |
|  | *Laphania boecki* | 4 | 0.2668 |
|  | *Lumbrinerides acuta* | 164 | 3.58 |
|  | *Mediomastus ambiseta* | 4 | 0.0048 |
|  | *Metopella* sp. | 2 | 0.0002 |
|  | *Musculus* sp. | 2 | 0.0278 |
|  | Mytiloidea | 56 | 0.2096 |
|  | Nemertea | 252 | 1.2008 |
|  | *Nephtys caeca* | 2 | 2.2232 |
|  | *Nereis* sp. | 8 | 0.1192 |
|  | *Nothria conchylega* | 2 | 0.008 |
|  | *Notomastus latericeus* | 6 | 0.0782 |
|  | Oligochaeta | 132 | 0.2024 |
|  | *Ophiura* sp. juvenile | 4 | 0.0002 |
|  | *Orbinia swani* | 10 | 0.0342 |
|  | *Pagurus* sp. | 2 | 0.3322 |
|  | *Paraonis* sp. | 84 | 0.121 |
|  | *Parougia eliasoni* | 10 | 0.0094 |
|  | *Parvicardium pinnulatum* | 6 | 0.0258 |
|  | *Periploma leanum* | 2 | 0.9758 |
|  | *Pholoe tecta* | 126 | 0.1098 |
|  | *Phyllodoce maculata* | 6 | 0.0042 |
|  | *Pleusymtes glaber* | 76 | 0.0468 |
|  | *Polycirrus* sp. | 10 | 0.2114 |
|  | *Polydora concharum* | 2 | 0.0012 |
|  | Polygordiidae | 308 | 2.6886 |
|  | Polynoidae sp. juvenile | 18 | 0.041 |
|  | *Protodorvillea gaspeensis* | 2 | 0.0012 |
|  | *Protomedeia fasciata* | 8 | 0.003 |
|  | *Scolelepis* sp. A | 2 | 0.2962 |
|  | *Scoloplos armiger* | 2 | 0.0182 |
|  | *Sertularia polyzonias* | 2 | 0.0084 |
|  | *Sertularia* sp. | 2 | 0.0076 |
|  | *Spio filicornis* | 16 | 0.0598 |
|  | *Strongylocentrotus* sp. | 4 | 0.0446 |
|  | Syllidae | 96 | 0.0162 |
|  | *Tharyx* sp. | 106 | 0.4076 |
|  | *Thracia* sp. | 26 | 0.0194 |
|  | *Thuiaria* sp. | 2 | 0.0904 |
|  | *Unciola irrorata* | 154 | 0.4948 |
| vg32 | *Aglaophamus circinata* | 2 | 0.0294 |
|  | *Ampharete finmarchica* | 24 | 0.0612 |
|  | *Arctica islandica* | 24 | 6.4762 |
|  | *Aricidea wassi* | 6 | 0.01 |
|  | Ascidiacea (solitary) | 2 | 0.0002 |
|  | *Capitella capitata* | 4 | 0.003 |
|  | *Chiridotea tuftsii* | 4 | 0.0568 |
|  | *Cirolana polita* | 2 | 0.1466 |
|  | *Cistenides granulata* | 6 | 0.0126 |
|  | *Clymenura borealis* | 40 | 3.6688 |
|  | *Crangon septemspinosa* | 2 | 0.042 |
|  | *Cyrtodaria siliqua* | 8 | 394.7074 |
|  | *Echinarachnius parma* | 18 | 73.1714 |
|  | *Edwardsia elegans* | 62 | 0.1158 |
|  | *Euchone papillosa* | 2 | 0.0058 |
|  | *Euclymene zonalis* | 2 | 0.0144 |
|  | *Eucratea loricata* | 2 | 0.001 |
|  | *Euspira* sp. | 2 | 0.0004 |
|  | *Glycera capitata* | 2 | 0.0058 |
|  | *Hippomedon serratus* | 8 | 0.1528 |
|  | *Levensinea gracilis* | 8 | 0.006 |
|  | *Lumbrinerides acuta* | 22 | 0.1922 |
|  | *Mactromeris polynyma* | 2 | 14.728 |
|  | *Monoculodes* sp. | 2 | 0.0028 |
|  | Mytiloidea | 2 | 0.005 |
|  | Nemertea | 12 | 0.0384 |
|  | *Nephtys bucera* | 4 | 0.3516 |
|  | *Notomastus latericeus* | 2 | 0.0592 |
|  | *Ophelia limacina* | 6 | 2.0864 |
|  | *Orbinia swani* | 6 | 1.0082 |
|  | *Pagurus* sp. | 2 | 0.0048 |
|  | *Paraonis* sp. | 6 | 0.005 |
|  | *Photis* sp. | 2 | 0.0004 |
|  | *Pleusymtes glaber* | 2 | 0.0008 |
|  | *Polycirrus* sp. | 2 | 0.0012 |
|  | *Pontoporeia* sp. | 2 | 0.0052 |
|  | *Pseudounicola obliquua* | 2 | 0.0028 |
|  | *Scoloplos armiger* | 4 | 0.1942 |
|  | *Solariella obscura* | 4 | 0.0482 |
|  | *Spio filicornis* | 4 | 0.0136 |
|  | *Spiophanes bombyx* | 2 | 0.0002 |
|  | *Tharyx* sp. | 16 | 0.0764 |
|  | *Travisia* sp. | 6 | 0.1652 |
|  | *Unciola irrorata* | 32 | 0.1466 |
| vg33 | *Aglaophamus circinata* | 4 | 0.7422 |
|  | *Ampharete finmarchica* | 78 | 0.44 |
|  | *Arctica islandica* | 18 | 2.5182 |
|  | *Aricidea catherinae* | 14 | 0.0068 |
|  | Ascidiacea (solitary) | 2 | 0.0036 |
|  | *Autolytus* sp. | 2 | 0.0002 |
|  | *Boreocingula* sp. | 6 | 0.0004 |
|  | Campanulariidae | 2 | 0.0064 |
|  | Cerianthidae | 2 | 7.8804 |
|  | *Chaetozone* sp. A | 120 | 0.158 |
|  | *Chone* sp. | 304 | 16.3842 |
|  | *Cistenides granulata* | 18 | 0.0086 |
|  | *Clymenura borealis* | 16 | 1.7776 |
|  | *Corophium crassicorne* | 6 | 0.0022 |
|  | *Crangon septemspinosa* | 2 | 0.0194 |
|  | *Cucumaria frondosa* | 4 | 0.0052 |
|  | *Cylichna alba* | 6 | 0.0032 |
|  | *Cyrtodaria siliqua* | 88 | 0.9326 |
|  | *Diastylis sculpta* | 2 | 0.0002 |
|  | *Drilonereis magna* | 2 | 0.0052 |
|  | *Dyopedos* sp. | 2 | 0.0004 |
|  | *Echinarachnius parma* | 2 | 0.0042 |
|  | *Edwardsia elegans* | 122 | 4.0374 |
|  | *Ericthonius fasciatus* | 8 | 0.0146 |
|  | *Euchone papillosa* | 22 | 0.154 |
|  | *Euclymene zonalis* | 826 | 13.9806 |
|  | *Eucratea loricata* | 2 | 0.0034 |
|  | *Exogone* sp. | 66 | 0.0176 |
|  | *Glycera capitata* | 296 | 3.5142 |
|  | *Harmothoe extenuata* | 2 | 0.0012 |
|  | *Ischyrocerus* sp. | 248 | 0.0766 |
|  | *Lumbrinerides acuta* | 62 | 0.6876 |
|  | *Lumbrineris fragilis* | 2 | 0.0172 |
|  | *Mediomastus ambiseta* | 4 | 0.0002 |
|  | *Musculus* sp. | 4 | 0.0088 |
|  | Mytiloidea | 24 | 0.229 |
|  | Nemertea | 84 | 0.9154 |
|  | Nephtyidae sp. juvenile | 2 | 0.016 |
|  | *Nephtys caeca* | 2 | 3.3986 |
|  | *Nereis* sp. | 8 | 0.1268 |
|  | *Notomastus latericeus* | 12 | 0.2328 |
|  | Oligochaeta | 170 | 0.12 |
|  | *Ophelia limacina* | 12 | 0.0002 |
|  | *Ophiura* sp. juvenile | 4 | 0.0002 |
|  | *Orbinia swani* | 6 | 0.5072 |
|  | *Paraonis* sp. | 188 | 0.1576 |
|  | *Parougia eliasoni* | 14 | 0.004 |
|  | *Parvicardium pinnulatum* | 14 | 0.6162 |
|  | *Periploma leanum* | 2 | 0.1454 |
|  | *Pholoe tecta* | 38 | 0.0168 |
|  | *Photis* sp. | 8 | 0.0004 |
|  | *Polycirrus* sp. | 8 | 0.1022 |
|  | Polygordiidae | 124 | 0.0702 |
|  | *Protodorvillea gaspeensis* | 12 | 0.0148 |
|  | *Protomedeia fasciata* | 36 | 0.0164 |
|  | *Psolus* sp. | 14 | 0.0256 |
|  | *Scoloplos armiger* | 2 | 0.0048 |
|  | *Serripes groenlandicus* | 2 | 0.0002 |
|  | *Solariella obscura* | 4 | 0.0156 |
|  | *Spio filicornis* | 14 | 0.0272 |
|  | *Spiophanes bombyx* | 4 | 0.0408 |
|  | Syllidae | 158 | 0.0208 |
|  | *Tharyx* sp. | 138 | 0.4866 |
|  | *Thracia* sp. | 6 | 0.0202 |
|  | *Unciola irrorata* | 688 | 2.9614 |
|  | *Velutina* sp. | 2 | 0.0032 |
| vg34 | Actiniaria (burrowing) | 14 | 0.9554 |
|  | Actiniaria (non burrowing) | 2 | 0.0014 |
|  | *Aglaophamus circinata* | 6 | 0.1724 |
|  | *Ampharete finmarchica* | 40 | 0.1174 |
|  | *Anonyx sarsi* | 12 | 0.0568 |
|  | *Arctica islandica* | 26 | 13.3824 |
|  | *Aricidea catherinae* | 10 | 0.011 |
|  | Cerianthidae | 2 | 2.0636 |
|  | *Chaetozone* sp. A | 28 | 0.0564 |
|  | *Chone* sp. | 8 | 0.0134 |
|  | *Clymenura borealis* | 34 | 1.9672 |
|  | *Cylichna alba* | 4 | 0.448 |
|  | *Cyrtodaria siliqua* | 16 | 389.3208 |
|  | *Dyopedos* sp. | 6 | 0.0044 |
|  | *Echinarachnius parma* | 10 | 103.92 |
|  | *Edwardsia elegans* | 8 | 0.5874 |
|  | *Euchone papillosa* | 4 | 0.0128 |
|  | *Euclymene zonalis* | 448 | 4.1124 |
|  | *Euspira* sp. | 4 | 0.0582 |
|  | *Exogone* sp. | 78 | 0.0216 |
|  | Gastropoda | 2 | 0.0024 |
|  | *Glycera capitata* | 94 | 0.762 |
|  | *Haploops* sp. | 2 | 0.0008 |
|  | *Ischyrocerus* sp. | 58 | 0.0116 |
|  | *Levensinea gracilis* | 6 | 0.0054 |
|  | *Lumbrinerides acuta* | 94 | 1.1252 |
|  | *Mactromeris polynyma* | 2 | 10.3626 |
|  | *Monoculodes* sp. | 2 | 0.003 |
|  | Mytiloidea | 8 | 0.0568 |
|  | Nemertea | 130 | 0.2198 |
|  | Nephtyidae sp. juvenile | 4 | 0.0192 |
|  | *Nephtys caeca* | 4 | 3.0964 |
|  | *Notomastus latericeus* | 20 | 0.5812 |
|  | Oligochaeta | 190 | 0.051 |
|  | *Ophelia limacina* | 4 | 0.0004 |
|  | *Orbinia swani* | 2 | 0.7566 |
|  | *Pandora* sp. | 6 | 5.4174 |
|  | *Paraonis* sp. | 94 | 0.1156 |
|  | *Parougia eliasoni* | 22 | 0.0044 |
|  | *Parvicardium pinnulatum* | 2 | 0.0012 |
|  | *Periploma leanum* | 2 | 3.1948 |
|  | *Pholoe minuta* | 2 | 0.0046 |
|  | *Photis* sp. | 2 | 0.0008 |
|  | *Pleusymtes glaber* | 14 | 0.0454 |
|  | Polygordiidae | 378 | 0.2464 |
|  | *Protomedeia fasciata* | 28 | 0.0092 |
|  | *Scoloplos armiger* | 2 | 0.0208 |
|  | *Spio filicornis* | 6 | 0.0228 |
|  | Syllidae | 140 | 0.0242 |
|  | *Tharyx* sp. | 24 | 0.0828 |
|  | *Thracia* sp. | 4 | 0.244 |
| vg35 | *Ampharete finmarchica* | 80 | 0.3378 |
|  | *Arctica islandica* | 2 | 0.2762 |
|  | *Argissa hamatipes* | 4 | 0.0032 |
|  | *Aricidea catherinae* | 12 | 0.009 |
|  | *Boreocingula* sp. | 28 | 0.0244 |
|  | Campanulariidae | 2 | 0.001 |
|  | Cerianthidae | 10 | 18.1294 |
|  | *Chone* sp. | 502 | 63.3516 |
|  | *Cistenides granulata* | 6 | 0.001 |
|  | *Cyclocardia* sp. A | 2 | 0.0034 |
|  | *Cyrtodaria siliqua* | 110 | 0.065 |
|  | *Drilonereis magna* | 16 | 0.722 |
|  | *Dyopedos* sp. | 2 | 0.0018 |
|  | *Echinarachnius parma* | 8 | 0.009 |
|  | *Edwardsia elegans* | 176 | 9.1624 |
|  | *Ericthonius fasciatus* | 28 | 0.035 |
|  | *Euchone papillosa* | 38 | 0.15 |
|  | *Euclymene zonalis* | 854 | 15.3072 |
|  | *Exogone* sp. | 40 | 0.0078 |
|  | Gastropoda | 2 | 0.0048 |
|  | *Glycera capitata* | 86 | 3.8148 |
|  | *Ischyrocerus* sp. | 90 | 0.0338 |
|  | *Laphania boecki* | 2 | 0.0076 |
|  | *Lumbrinerides acuta* | 52 | 0.747 |
|  | *Metopella* sp. | 2 | 0.0002 |
|  | *Moelleria costulata* | 10 | 0.0056 |
|  | *Monoculodes* sp. | 2 | 0.0152 |
|  | *Musculus* sp. | 2 | 0.0518 |
|  | Mytiloidea | 46 | 0.2376 |
|  | Nemertea | 10 | 0.1216 |
|  | Nephtyidae sp. juvenile | 2 | 0.0058 |
|  | *Nereis* sp. | 8 | 0.2266 |
|  | *Notomastus latericeus* | 6 | 0.1188 |
|  | Oligochaeta | 82 | 0.03 |
|  | *Ophiura* sp. juvenile | 2 | 0.0002 |
|  | *Orbinia swani* | 6 | 0.0368 |
|  | *Orchomenella minuta* | 2 | 0.0016 |
|  | Ostracoda | 4 | 0.0018 |
|  | *Paraonis* sp. | 158 | 0.0718 |
|  | *Parougia eliasoni* | 12 | 0.0032 |
|  | *Parvicardium pinnulatum* | 28 | 0.255 |
|  | *Pholoe tecta* | 22 | 0.0506 |
|  | *Photis* sp. | 20 | 0.006 |
|  | *Phyllodoce groenlandica* | 2 | 0.1278 |
|  | *Phyllodoce mucosa* | 8 | 0.0358 |
|  | *Pleusymtes glaber* | 4 | 0.0026 |
|  | Polygordiidae | 18 | 0.063 |
|  | Polynoidae sp. juvenile | 2 | 0.0008 |
|  | *Protodorvillea gaspeensis* | 2 | 0.0076 |
|  | *Protomedeia fasciata* | 80 | 0.0548 |
|  | *Psolus* sp. | 6 | 0.0086 |
|  | *Scoloplos armiger* | 2 | 0.018 |
|  | *Solariella obscura* | 2 | 0.0006 |
|  | *Spio filicornis* | 18 | 0.0364 |
|  | *Spiophanes bombyx* | 6 | 0.0416 |
|  | Syllidae | 44 | 0.0044 |
|  | *Tachyrhynchus reticulatus* | 2 | 0.19 |
|  | *Tharyx* sp. | 366 | 1.135 |
|  | *Thracia* sp. | 6 | 0.37 |
|  | *Unciola irrorata* | 908 | 2.5786 |
| vg36 | *Aglaophamus circinata* | 4 | 0.737 |
|  | *Ampharete finmarchica* | 6 | 0.0394 |
|  | *Anonyx sarsi* | 2 | 0.0152 |
|  | *Arctica islandica* | 50 | 15.3682 |
|  | *Aricidea wassi* | 8 | 0.0558 |
|  | Ascidiacea (solitary) | 4 | 0.0506 |
|  | *Capitella capitata* | 4 | 0.0116 |
|  | Cerianthidae | 2 | 5.6678 |
|  | *Cirolana polita* | 2 | 0.3336 |
|  | *Clymenura borealis* | 46 | 4.4452 |
|  | *Cylichna alba* | 4 | 0.3488 |
|  | *Cyrtodaria siliqua* | 34 | 297.8532 |
|  | *Echinarachnius parma* | 30 | 98.8626 |
|  | *Edwardsia elegans* | 10 | 0.1796 |
|  | *Euchone papillosa* | 4 | 0.0182 |
|  | *Euclymene zonalis* | 6 | 0.0554 |
|  | *Leptognathia* sp. | 2 | 0.0052 |
|  | *Levensinea gracilis* | 6 | 0.0054 |
|  | *Lumbrinerides acuta* | 26 | 0.3862 |
|  | *Mactromeris polynyma* | 4 | 14.22 |
|  | Nemertea | 6 | 0.7642 |
|  | Nephtyidae sp. juvenile | 6 | 0.0416 |
|  | *Nephtys caeca* | 2 | 0.5026 |
|  | *Notomastus latericeus* | 6 | 0.1412 |
|  | Oligochaeta | 4 | 0.0002 |
|  | *Ophelia limacina* | 6 | 0.4086 |
|  | *Orbinia swani* | 8 | 0.539 |
|  | *Paraonis* sp. | 2 | 0.0012 |
|  | *Parougia eliasoni* | 2 | 0.0002 |
|  | *Parvicardium pinnulatum* | 2 | 0.1514 |
|  | *Periploma leanum* | 4 | 0.236 |
|  | *Pholoe tecta* | 2 | 0.0438 |
|  | *Phoxocephalus holbolli* | 2 | 0.0038 |
|  | Platyhelminthes | 2 | 0.3152 |
|  | *Scalibregma inflatum* | 2 | 0.3118 |
|  | *Scolelepis* sp. A | 2 | 0.0762 |
|  | *Scoloplos armiger* | 6 | 0.0698 |
|  | *Spio filicornis* | 16 | 0.0328 |
|  | *Spiophanes bombyx* | 12 | 0.217 |
|  | Syllidae | 2 | 0.0014 |
|  | *Tharyx* sp. | 22 | 0.0996 |
|  | *Travisia* sp. | 14 | 0.26 |
|  | *Unciola irrorata* | 212 | 1.197 |
| vg37 | *Aglaophamus circinata* | 4 | 1.454 |
|  | *Ampharete finmarchica* | 16 | 0.0936 |
|  | *Arctica islandica* | 16 | 4.0428 |
|  | *Aricidea wassi* | 10 | 0.0576 |
|  | *Capitella capitata* | 2 | 0.0082 |
|  | *Chaetozone* sp. A | 6 | 0.0412 |
|  | *Chiridota laevis* | 2 | 0.0234 |
|  | *Cirolana polita* | 2 | 0.077 |
|  | *Clymenura borealis* | 38 | 6.0596 |
|  | *Cyrtodaria siliqua* | 20 | 144.0762 |
|  | *Diaphana minuta* | 2 | 0.0154 |
|  | *Echinarachnius parma* | 34 | 62.2394 |
|  | *Edwardsia elegans* | 18 | 0.0324 |
|  | *Euchone papillosa* | 6 | 0.0248 |
|  | *Euclymene zonalis* | 10 | 0.0778 |
|  | *Exogone* sp. | 16 | 0.0038 |
|  | *Glycera capitata* | 2 | 0.007 |
|  | *Harmothoe extenuata* | 2 | 0.0024 |
|  | *Hippomedon serratus* | 6 | 0.206 |
|  | *Levensinea gracilis* | 22 | 0.0192 |
|  | *Lumbrinerides acuta* | 32 | 0.4598 |
|  | *Mactromeris polynyma* | 2 | 0.6344 |
|  | Nemertea | 6 | 0.019 |
|  | *Notomastus latericeus* | 6 | 0.1662 |
|  | Oligochaeta | 2 | 0.0002 |
|  | *Ophelia limacina* | 8 | 3.7848 |
|  | *Orbinia swani* | 8 | 1.3188 |
|  | *Paraonis* sp. | 18 | 0.028 |
|  | *Parougia eliasoni* | 4 | 0.0014 |
|  | *Periploma leanum* | 6 | 0.2286 |
|  | *Phoxocephalus holbolli* | 2 | 0.0064 |
|  | Polygordiidae | 2 | 0.0006 |
|  | *Scolelepis* sp. A | 2 | 0.08 |
|  | *Scoloplos armiger* | 6 | 0.4694 |
|  | *Solariella obscura* | 4 | 0.2538 |
|  | *Spio filicornis* | 20 | 0.1082 |
|  | *Spiophanes bombyx* | 12 | 0.2436 |
|  | Syllidae | 16 | 0.0032 |
|  | *Tharyx* sp. | 56 | 0.2384 |
|  | *Travisia* sp. | 4 | 0.1738 |
|  | *Unciola irrorata* | 156 | 0.6376 |
| vg38 | Actiniaria (burrowing) | 2 | 0.6614 |
|  | *Aglaophamus circinata* | 4 | 1.075 |
|  | *Ampharete finmarchica* | 26 | 0.0446 |
|  | *Anonyx sarsi* | 2 | 0.0158 |
|  | *Aricidea catherinae* | 8 | 0.0066 |
|  | *Boreocingula* sp. | 2 | 0.002 |
|  | Campanulariidae | 2 | 0.0132 |
|  | Cerianthidae | 4 | 24.545 |
|  | *Chaetozone* sp. A | 16 | 0.0582 |
|  | *Chone* sp. | 30 | 0.033 |
|  | *Cistenides granulata* | 2 | 0.0008 |
|  | *Clymenura borealis* | 22 | 2.6056 |
|  | *Cucumaria frondosa* | 50 | 0.0422 |
|  | *Cyclocardia novangliae* | 2 | 0.0088 |
|  | *Cylichna alba* | 2 | 0.0436 |
|  | *Cyrtodaria siliqua* | 50 | 0.0084 |
|  | *Echinarachnius parma* | 10 | 0.014 |
|  | *Edwardsia elegans* | 22 | 0.9048 |
|  | *Edwardsia sulcata* | 4 | 0.0572 |
|  | *Ericthonius fasciatus* | 4 | 0.011 |
|  | *Euchone papillosa* | 8 | 0.0154 |
|  | *Euclymene zonalis* | 892 | 13.4184 |
|  | *Eucratea loricata* | 2 | 0.0056 |
|  | *Euspira* sp. | 2 | 0.044 |
|  | *Exogone* sp. | 20 | 0.006 |
|  | *Glycera capitata* | 200 | 3.6474 |
|  | *Goniadella gracilis* | 2 | 0.0058 |
|  | *Ischyrocerus* sp. | 56 | 0.0246 |
|  | *Laphania boecki* | 4 | 0.1704 |
|  | *Lumbrinerides acuta* | 48 | 0.6798 |
|  | *Mactromeris polynyma* | 2 | 0.0002 |
|  | *Musculus* sp. | 4 | 0.0034 |
|  | Mytiloidea | 14 | 0.012 |
|  | Nemertea | 98 | 0.1154 |
|  | Nephtyidae sp. juvenile | 2 | 0.0116 |
|  | *Nephtys caeca* | 2 | 5.3502 |
|  | *Nereis* sp. | 2 | 0.015 |
|  | *Notomastus latericeus* | 6 | 0.1848 |
|  | Oligochaeta | 62 | 0.0124 |
|  | *Ophelia limacina* | 4 | 0.0002 |
|  | *Ophiura* sp. juvenile | 2 | 0.0016 |
|  | *Orbinia swani* | 2 | 0.0622 |
|  | *Paraonis* sp. | 144 | 0.297 |
|  | *Parougia eliasoni* | 6 | 0.0026 |
|  | *Parvicardium pinnulatum* | 6 | 0.1296 |
|  | *Periploma leanum* | 4 | 0.029 |
|  | *Pholoe tecta* | 4 | 0.0004 |
|  | *Pleusymtes glaber* | 18 | 0.0096 |
|  | *Polydora concharum* | 2 | 0.0038 |
|  | Polygordiidae | 260 | 0.2868 |
|  | Polynoidae sp. juvenile | 2 | 0.0002 |
|  | *Pontogeneia inermis* | 2 | 0.011 |
|  | *Protodorvillea gaspeensis* | 4 | 0.0002 |
|  | *Protodorvillea kefersteini* | 2 | 0.0002 |
|  | *Protomedeia fasciata* | 46 | 0.035 |
|  | *Rhizocaulus verticillatus* | 2 | 0.0034 |
|  | *Sertularia* sp. | 2 | 0.0002 |
|  | *Spio filicornis* | 4 | 0.0088 |
|  | Syllidae | 180 | 0.028 |
|  | *Tharyx* sp. | 42 | 0.1856 |
|  | *Thracia* sp. | 2 | 0.0018 |
|  | *Unciola irrorata* | 74 | 0.1864 |
| vg38e | *Aglaophamus circinata* | 10 | 2.003 |
|  | *Ampharete finmarchica* | 16 | 0.1656 |
|  | *Arctica islandica* | 56 | 13.3048 |
|  | *Aricidea wassi* | 6 | 0.03 |
|  | Campanulariidae | 2 | 0.0028 |
|  | *Chiridotea tuftsii* | 4 | 0.1068 |
|  | *Cirolana polita* | 6 | 0.5836 |
|  | *Clymenura borealis* | 86 | 13.2228 |
|  | *Cylichna alba* | 2 | 0.0616 |
|  | *Cyrtodaria siliqua* | 18 | 0.4622 |
|  | *Diaphana minuta* | 2 | 0.022 |
|  | *Diastylis sculpta* | 2 | 0.0066 |
|  | *Echinarachnius parma* | 28 | 247.0766 |
|  | *Edwardsia elegans* | 32 | 1.483 |
|  | *Euchone papillosa* | 8 | 0.0668 |
|  | *Eudorellopsis deformis* | 2 | 0.0016 |
|  | *Euspira* sp. | 8 | 0.1994 |
|  | *Hippomedon serratus* | 10 | 0.1876 |
|  | *Liocyma fluctuosa* | 2 | 0.8822 |
|  | *Lumbrinerides acuta* | 20 | 0.6524 |
|  | *Mactromeris polynyma* | 2 | 0.0162 |
|  | *Monoculodes* sp. | 4 | 0.0106 |
|  | Nemertea | 10 | 1.1368 |
|  | *Nephtys caeca* | 6 | 4.7204 |
|  | *Nereis* sp. | 6 | 0.5614 |
|  | *Notomastus latericeus* | 16 | 0.3798 |
|  | Oligochaeta | 2 | 0.0002 |
|  | *Ophelia limacina* | 4 | 1.7078 |
|  | *Orbinia swani* | 8 | 0.6168 |
|  | *Orchomenella minuta* | 2 | 0.0028 |
|  | *Paraonis* sp. | 6 | 0.0104 |
|  | *Periploma leanum* | 10 | 0.0956 |
|  | *Pholoe tecta* | 4 | 0.0876 |
|  | *Phoxocephalus holbolli* | 10 | 0.0342 |
|  | Phyllodocidae sp. juvenile | 2 | 0.0208 |
|  | *Pleustes panoplus* | 2 | 0.032 |
|  | Polygordiidae | 2 | 0.0028 |
|  | *Scolelepis* sp. A | 2 | 0.112 |
|  | *Scoloplos armiger* | 12 | 0.9496 |
|  | *Serripes groenlandicus* | 4 | 0.004 |
|  | *Sertularia* sp. | 2 | 0.0036 |
|  | *Solariella obscura* | 6 | 0.1304 |
|  | *Spio filicornis* | 2 | 0.0038 |
|  | *Spiophanes bombyx* | 14 | 0.1074 |
|  | *Tharyx* sp. | 4 | 0.0196 |
|  | *Travisia* sp. | 18 | 0.5054 |
|  | *Unciola irrorata* | 130 | 0.8156 |
| vg39 | Actiniaria (non burrowing) | 2 | 0.0152 |
|  | *Ampharete finmarchica* | 70 | 0.3752 |
|  | *Anomia* sp. | 2 | 0.0706 |
|  | *Aricidea catherinae* | 58 | 0.0412 |
|  | Ascidiacea (solitary) | 2 | 0.0008 |
|  | Asteroidea | 4 | 0.1812 |
|  | *Boreocingula* sp. | 18 | 0.041 |
|  | Campanulariidae | 2 | 0.0056 |
|  | *Caprella* sp. | 2 | 0.0022 |
|  | Cerianthidae | 14 | 34.023 |
|  | *Chone* sp. | 670 | 38.7706 |
|  | *Cistenides granulata* | 4 | 0.0046 |
|  | *Corophium crassicorne* | 132 | 0.1144 |
|  | *Cryptonatica affinis* | 2 | 1.0226 |
|  | *Cucumaria frondosa* | 22 | 0.0466 |
|  | *Cyclocardia novangliae* | 4 | 0.0318 |
|  | *Cylichna alba* | 2 | 0.0218 |
|  | *Cyrtodaria siliqua* | 12 | 1.3844 |
|  | *Dendrobeania* sp. | 2 | 0.2808 |
|  | *Diastylis sculpta* | 4 | 0.0118 |
|  | *Drilonereis magna* | 8 | 1.525 |
|  | *Dyopedos* sp. | 4 | 0.00252 |
|  | *Echinarachnius parma* | 14 | 0.0268 |
|  | *Edwardsia elegans* | 258 | 14.6686 |
|  | *Edwardsia sipunculoides* | 2 | 0.0382 |
|  | *Ericthonius fasciatus* | 250 | 0.6152 |
|  | *Eteone longa* | 2 | 0.0598 |
|  | *Euchone papillosa* | 16 | 0.059 |
|  | *Euclymene zonalis* | 506 | 4.6312 |
|  | *Eulalia viridis* | 2 | 0.0004 |
|  | *Exogone* sp. | 70 | 0.01 |
|  | *Galathowenia oculata* | 2 | 0.0576 |
|  | *Glycera capitata* | 92 | 4.4404 |
|  | *Goniada maculata* | 8 | 0.609 |
|  | *Grammaria abietina* | 2 | 0.0178 |
|  | *Harmothoe extenuata* | 8 | 0.0396 |
|  | *Harmothoe imbricata* | 6 | 0.2432 |
|  | *Hydrallmania falcata* | 2 | 0.0098 |
|  | *Ischyrocerus* sp. | 56 | 0.0324 |
|  | *Laonice cirrata* | 2 | 0.0642 |
|  | *Laphania boecki* | 4 | 0.1202 |
|  | *Leptocheirus pinguis* | 8 | 0.2148 |
|  | *Leucosolenia* sp. | 2 | 0.0134 |
|  | *Lumbrinerides acuta* | 2 | 0.0198 |
|  | *Mediomastus ambiseta* | 20 | 0.0154 |
|  | *Melina* sp. | 2 | 0.0386 |
|  | *Moelleria costulata* | 4 | 0.004 |
|  | *Monoculodes* sp. | 2 | 0.0094 |
|  | *Musculus* sp. | 2 | 0.01 |
|  | Mytiloidea | 90 | 1.2314 |
|  | Nemertea | 14 | 0.6034 |
|  | *Nereis* sp. | 16 | 0.11 |
|  | *Nicomache lumbricalis* | 4 | 2.7938 |
|  | *Nothria conchylega* | 2 | 0.0576 |
|  | *Notomastus latericeus* | 20 | 0.6402 |
|  | *Ophelia limacina* | 2 | 0.0084 |
|  | *Ophiura* sp. juvenile | 2 | 0.0058 |
|  | *Paraonis* sp. | 4 | 0.0042 |
|  | *Parvicardium pinnulatum* | 6 | 0.1786 |
|  | *Periploma leanum* | 2 | 0.046 |
|  | *Petaloproctus tenuis* | 2 | 0.0018 |
|  | *Pholoe tecta* | 6 | 0.0088 |
|  | *Photis* sp. | 14 | 0.018 |
|  | *Phoxocephalus holbolli* | 2 | 0.0006 |
|  | *Phyllodoce maculata* | 10 | 0.0074 |
|  | Phyllodocidae sp. juvenile | 4 | 0.0056 |
|  | *Polycirrus* sp. | 12 | 0.3608 |
|  | *Polydora concharum* | 6 | 0.0602 |
|  | *Praxillella praetermissa* | 18 | 0.0368 |
|  | *Prionospio steenstrupi* | 2 | 0.0008 |
|  | *Protodorvillea gaspeensis* | 2 | 0.003 |
|  | *Protomedeia fasciata* | 62 | 0.044 |
|  | *Psolus* sp. | 8 | 0.0006 |
|  | *Rhizocaulus verticillatus* | 2 | 0.0392 |
|  | Sabellidae | 22 | 0.0044 |
|  | *Scolelepis* sp. A | 2 | 0.07 |
|  | *Scoloplos armiger* | 2 | 0.0018 |
|  | *Serripes groenlandicus* | 2 | 0.0038 |
|  | *Sertularia polyzonias* | 2 | 0.039 |
|  | *Sertularia* sp. | 2 | 0.0046 |
|  | *Spio filicornis* | 4 | 0.037 |
|  | *Spiochaetopterus typicus* | 6 | 0.0928 |
|  | Syllidae | 8 | 0.0614 |
|  | *Tharyx* sp. | 66 | 0.0886 |
|  | *Thelepus cincinnatus* | 2 | 0.7152 |
|  | *Thracia* sp. | 8 | 0.0306 |
|  | *Thuiaria* sp. | 2 | 0.2806 |
|  | *Travisia* sp. | 38 | 1.2786 |
|  | *Tricellaria gracilis* | 2 | 0.0018 |
|  | *Unciola irrorata* | 1480 | 10.8562 |
| vg40 | *Achelia* sp. | 2 | 0.0014 |
|  | Actiniaria (non burrowing) | 2 | 0.0748 |
|  | *Ampharete finmarchica* | 8 | 0.0116 |
|  | *Anomia* sp. | 14 | 0.0264 |
|  | *Anonyx sarsi* | 4 | 0.0268 |
|  | *Arctica islandica* | 4 | 0.137 |
|  | Asteroidea | 4 | 0.5022 |
|  | *Autolytus* sp. | 2 | 0.0026 |
|  | *Caprella* sp. | 278 | 0.143 |
|  | *Chaetozone* sp. | 2 | 0.003 |
|  | *Chaetozone* sp. A | 6 | 0.0038 |
|  | *Chlamys islandica* | 2 | 0.0018 |
|  | *Chone* sp. | 4 | 0.001 |
|  | *Clymenura borealis* | 4 | 0.2134 |
|  | *Crisia* sp. | 2 | 0.0022 |
|  | *Cucumaria frondosa* | 12 | 0.0112 |
|  | *Cyrtodaria siliqua* | 8 | 0.0118 |
|  | Didemnidae | 2 | 8.5896 |
|  | *Drilonereis magna* | 2 | 0.4582 |
|  | *Echinarachnius parma* | 12 | 0.0918 |
|  | *Edwardsia elegans* | 6 | 0.2242 |
|  | *Ericthonius rubricornis* | 12 | 0.0244 |
|  | *Euchone papillosa* | 4 | 0.0126 |
|  | *Euclymene zonalis* | 8 | 0.0578 |
|  | *Eulalia viridis* | 8 | 0.0182 |
|  | *Exogone* sp. | 12 | 0.0028 |
|  | *Gersemia rubrifornis* | 2 | 1.2556 |
|  | *Glycera capitata* | 10 | 0.509 |
|  | *Hiatella arctica* | 4 | 0.0852 |
|  | Hydrozoa Athecata | 2 | 0.0118 |
|  | *Ischyrocerus* sp. | 76 | 0.012 |
|  | *Leucosolenia* sp. | 2 | 0.2458 |
|  | *Lumbrinerides acuta* | 2 | 0.0238 |
|  | *Mediomastus ambiseta* | 2 | 0.0012 |
|  | *Modiolus modiolus* | 10 | 505.321 |
|  | *Munna fabricii* | 2 | 0.0002 |
|  | *Musculus* sp. | 4 | 0.042 |
|  | Mytiloidea | 6 | 0.024 |
|  | Nemertea | 12 | 0.0556 |
|  | *Nereis* sp. | 4 | 0.051 |
|  | *Nicolea venusula* | 2 | 0.0178 |
|  | Nudibranchia Group 4b | 6 | 0.0464 |
|  | Oligochaeta | 18 | 0.0006 |
|  | *Ophiopholis aculeata* | 44 | 0.0818 |
|  | *Ophiura* sp. juvenile | 4 | 0.002 |
|  | *Orbinia swani* | 2 | 0.0082 |
|  | *Paraonis* sp. | 4 | 0.0032 |
|  | *Parougia eliasoni* | 2 | 0.0008 |
|  | *Pholoe tecta* | 4 | 0.009 |
|  | *Photis* sp. | 10 | 0.0068 |
|  | *Phoxocephalus holbolli* | 2 | 0.0036 |
|  | *Phyllodoce maculata* | 10 | 0.0018 |
|  | *Polycirrus* sp. | 10 | 0.1082 |
|  | *Polydora concharum* | 2 | 0.0002 |
|  | Polygordiidae | 8 | 0.0008 |
|  | Polynoidae sp. juvenile | 2 | 0.001 |
|  | Porifera | 2 | 0.075 |
|  | *Praxillella praetermissa* | 2 | 0.0046 |
|  | *Protodorvillea gaspeensis* | 2 | 0.0002 |
|  | *Protomedeia fasciata* | 8 | 0.001 |
|  | *Pseudopotamilla* sp. | 8 | 0.0158 |
|  | *Psolus* sp. | 2 | 0.0016 |
|  | Sabellidae | 46 | 0.0238 |
|  | *Serripes groenlandicus* | 6 | 0.0022 |
|  | *Spio filicornis* | 4 | 0.0348 |
|  | *Spiophanes bombyx* | 2 | 0.0072 |
|  | *Strongylocentrotu*s sp. | 6 | 2.1158 |
|  | Syllidae | 16 | 0.0112 |
|  | *Tharyx* sp. | 22 | 0.0488 |
|  | *Thelepus cincinnatus* | 18 | 8.2062 |
|  | *Thracia* sp. | 4 | 0.016 |
|  | *Unciola irrorata* | 72 | 0.311 |
|  | *Urticina felina* | 2 | 0.1986 |
| vg41 | *Acanthonotozoma serratum* | 4 | 0.0154 |
|  | *Achelia* sp. | 32 | 0.0452 |
|  | Onchidoridae | 2 | 0.088 |
|  | *Aeginina longicornis* | 2 | 0.006 |
|  | *Ampharete finmarchica* | 16 | 0.2458 |
|  | Amphiuridae | 2 | 0.01 |
|  | *Anomia* sp. | 378 | 0.4234 |
|  | *Aricidea catherinae* | 6 | 0.0072 |
|  | *Asabellides* sp. | 2 | 0.019 |
|  | Ascidiacea (solitary) | 32 | 10.1114 |
|  | Asteroidea | 18 | 0.8196 |
|  | *Autolytus* sp. | 4 | 0.0042 |
|  | *Balanus* sp. | 2 | 0.0008 |
|  | Bryozoa Cyclostomata | 2 | 0.1124 |
|  | Buccinidae | 2 | 14.5546 |
|  | Campanulariidae | 2 | 0.0282 |
|  | *Cancer borealis* | 2 | 0.2322 |
|  | *Capitella capitata* | 6 | 0.0126 |
|  | *Caprella* sp. | 272 | 0.2356 |
|  | Cerianthidae | 4 | 11.671 |
|  | *Chaetozone* sp. | 10 | 0.0038 |
|  | *Chlamys islandica* | 8 | 0.0296 |
|  | *Chone* sp. | 98 | 27.6074 |
|  | *Cirratulus cirratus* | 36 | 0.082 |
|  | *Cistenides granulata* | 42 | 0.3876 |
|  | *Clymenura borealis* | 2 | 0.0078 |
|  | *Corophium crassicorne* | 2 | 0.0064 |
|  | *Crisia* sp. | 2 | 0.0002 |
|  | *Dendrobeania* sp. | 2 | 10.256 |
|  | Didemnidae | 2 | 0.1178 |
|  | *Dodecaceria fimbriata* | 6 | 0.0132 |
|  | *Dyopedos* sp. | 14 | 0.009 |
|  | *Edwardsia elegans* | 8 | 1.0172 |
|  | *Enipo gracilis* | 4 | 0.3742 |
|  | *Ericthonius rubricornis* | 122 | 0.169 |
|  | *Eteone longa* | 10 | 0.4918 |
|  | *Eualus pusiolus* | 6 | 0.1396 |
|  | *Euclymene zonalis* | 162 | 3.0444 |
|  | *Eudendrium* sp. | 2 | 0.04 |
|  | *Eulalia bilineata* | 2 | 0.0452 |
|  | *Eulalia viridis* | 54 | 0.097 |
|  | *Eunoe nodosa* | 2 | 0.2246 |
|  | *Exogone* sp. | 58 | 0.011 |
|  | Gastropoda | 4 | 0.178 |
|  | *Gattyana cirrhosa* | 4 | 0.012 |
|  | *Gattyana nutti* | 2 | 0.0546 |
|  | *Glycera capitata* | 28 | 0.8476 |
|  | *Golfingia* sp. | 2 | 0.0028 |
|  | *Harmothoe extenuata* | 6 | 0.1436 |
|  | *Harmothoe imbricata* | 4 | 0.0414 |
|  | *Hartmania moorei* | 2 | 0.024 |
|  | *Hiatella arctica* | 132 | 1.7582 |
|  | *Hyas coarctatus* | 4 | 0.1868 |
|  | Hydrozoa Athecata | 2 | 0.0032 |
|  | *Ischyrocerus* sp. | 74 | 0.0198 |
|  | *Janira alta* | 8 | 0.0114 |
|  | *Lafoea* sp. | 2 | 0.275 |
|  | *Leucosolenia* sp. | 2 | 0.5138 |
|  | *Maera danae* | 8 | 0.0578 |
|  | *Margarites striatus* | 2 | 0.0056 |
|  | *Mediomastus ambiseta* | 6 | 0.0128 |
|  | *Melina* sp. | 2 | 0.1266 |
|  | *Modiolus modiolus* | 66 | 2839.8364 |
|  | *Moelleria costulata* | 6 | 0.012 |
|  | *Munna fabricii* | 10 | 0.0002 |
|  | *Musculus* sp. | 16 | 0.0252 |
|  | Mytiloidea | 20 | 0.1764 |
|  | *Nainereis quadricuspida* | 6 | 0.0124 |
|  | Nemertea | 82 | 0.3518 |
|  | Nephtyidae sp. juvenile | 2 | 0.0062 |
|  | *Nephtys discors* | 2 | 29.34 |
|  | *Nereis* sp. | 30 | 0.1484 |
|  | *Nicolea venusula* | 6 | 0.0846 |
|  | *Nicomache lumbricalis* | 8 | 4.3886 |
|  | *Notomastus latericeus* | 12 | 0.1164 |
|  | Nudibranchia Group 4b | 8 | 0.0074 |
|  | Nudibranchia Group 4b-2 | 16 | 0.012 |
|  | *Nymphon hirtipes* | 8 | 0.0046 |
|  | *Ophelia limacina* | 2 | 0.0534 |
|  | *Ophiopholis aculeata* | 112 | 9.9372 |
|  | *Ophiura robusta* | 144 | 1.01 |
|  | *Ophiura* sp. juvenile | 8 | 0.0176 |
|  | Ostracoda | 2 | 0.0004 |
|  | *Owenia fusiformis* | 2 | 0.0056 |
|  | *Pagurus* sp. | 4 | 0.054 |
|  | *Paraonis* sp. | 10 | 0.0184 |
|  | *Parougia eliasoni* | 8 | 0.0004 |
|  | *Parvicardium pinnulatum* | 2 | 0.2072 |
|  | *Petaloproctus tenuis* | 144 | 2.0972 |
|  | *Pherusa plumosa* | 2 | 0.0006 |
|  | *Pholoe tecta* | 4 | 0.0066 |
|  | *Photis* sp. | 16 | 0.0128 |
|  | *Phoxocephalus holbolli* | 10 | 0.0096 |
|  | *Phyllodoce maculata* | 124 | 0.6758 |
|  | Phyllodocidae sp. juvenile | 4 | 0.006 |
|  | Platyhelminthes | 2 | 0.0098 |
|  | *Pleusymtes glaber* | 158 | 0.1058 |
|  | *Polycirrus* sp. | 90 | 1.6094 |
|  | *Polydora caulleryi* | 8 | 0.1022 |
|  | *Polydora concharum* | 100 | 1.9356 |
|  | *Praxillella praetermissa* | 4 | 0.1348 |
|  | *Praxillura ornata* | 26 | 1.1888 |
|  | *Protodorvillea gaspeensis* | 2 | 0.0002 |
|  | *Protomedeia fasciata* | 8 | 0.0056 |
|  | *Protomystides* sp. | 2 | 0.008 |
|  | *Pseudopotamilla* sp. | 408 | 27.585 |
|  | *Psolus* sp. | 22 | 0.052 |
|  | *Puncturella noachina* | 20 | 0.1158 |
|  | *Rhizocaulus verticillatus* | 2 | 0.0586 |
|  | Sabellidae | 116 | 0.0672 |
|  | *Scalibregma inflatum* | 20 | 0.0342 |
|  | *Scoloplos armiger* | 4 | 0.2104 |
|  | *Scrupocellaria scabra* | 2 | 0.1152 |
|  | *Sertularia* sp. | 2 | 0.4758 |
|  | *Spio filicornis* | 2 | 0.017 |
|  | *Stenosemus albus* | 6 | 0.2182 |
|  | Stenothoidae | 2 | 0.0002 |
|  | *Strongylocentrotus* sp. | 10 | 0.0042 |
|  | Syllidae | 80 | 0.0546 |
|  | *Tharyx* sp. | 148 | 0.2418 |
|  | *Thelepus cincinnatus* | 246 | 56.4312 |
|  | *Thracia* sp. | 8 | 0.0368 |
|  | *Tiron spiniferus* | 2 | 0.0034 |
|  | *Travisia* sp. | 2 | 0.017 |
|  | *Unciola irrorata* | 154 | 0.8444 |
|  | *Velutina* sp. | 6 | 0.0458 |
| vg42 | *Acanthonotozoma serratum* | 4 | 0.0504 |
|  | Actiniaria (burrowing) | 2 | 0.0226 |
|  | Actiniaria (non burrowing) | 2 | 0.085 |
|  | Onchidoridae | 2 | 0.0388 |
|  | *Aeginina longicornis* | 30 | 0.1292 |
|  | *Ampharete finmarchica* | 26 | 0.3408 |
|  | Amphiuridae | 4 | 0.0096 |
|  | *Anomia* sp. | 30 | 0.0454 |
|  | *Arctica islandica* | 2 | 0.008 |
|  | *Aricidea catherinae* | 24 | 0.0246 |
|  | *Aricidea* sp. B | 2 | 0.0016 |
|  | *Asabellides* sp. | 6 | 0.1314 |
|  | Ascidiacea (solitary) | 108 | 0.6444 |
|  | Asteroidea | 18 | 0.1304 |
|  | *Autolytus* sp. | 2 | 0.0018 |
|  | *Boreocingula* sp. | 4 | 0.0074 |
|  | Campanulariidae | 2 | 0.0058 |
|  | *Caprella* sp. | 328 | 0.5052 |
|  | Cerianthidae | 26 | 42.3596 |
|  | *Chaetozone* sp. A | 40 | 0.0614 |
|  | *Chlamys islandica* | 2 | 0.0324 |
|  | *Chone* sp. | 58 | 6.6732 |
|  | *Cistenides granulata* | 18 | 0.101 |
|  | *Corophium crassicorne* | 64 | 0.0716 |
|  | *Crisia* sp. | 2 | 0.0002 |
|  | *Cucumaria frondosa* | 92 | 0.254 |
|  | *Cyclocardia novangliae* | 16 | 0.4388 |
|  | *Cyrtodaria siliqua* | 2 | 344.7 |
|  | *Dendrobeania* sp. | 2 | 0.0916 |
|  | *Doto* sp. | 2 | 0.0208 |
|  | *Drilonereis magna* | 6 | 0.0734 |
|  | *Echinarachnius parma* | 22 | 0.0476 |
|  | *Edwardsia elegans* | 26 | 3.8056 |
|  | *Ericthonius rubricornis* | 72 | 0.0894 |
|  | *Eualus pusiolus* | 2 | 0.1258 |
|  | *Eubranchus* sp. | 2 | 0.0118 |
|  | *Euchone papillosa* | 16 | 0.098 |
|  | *Euclymene zonalis* | 406 | 4.0004 |
|  | *Eulalia viridis* | 2 | 0.0426 |
|  | *Euspira* sp. | 2 | 0.0208 |
|  | *Exogone* sp. | 30 | 0.0058 |
|  | *Gattyana cirrhosa* | 12 | 0.0406 |
|  | *Gersemia rubrifornis* | 2 | 0.0996 |
|  | *Glycera capitata* | 44 | 1.5524 |
|  | *Goniada maculata* | 6 | 0.8648 |
|  | *Harmothoe extenuata* | 4 | 0.0834 |
|  | *Harmothoe imbricata* | 8 | 0.0622 |
|  | *Hiatella arctica* | 16 | 0.3378 |
|  | *Idmidronea* sp. | 2 | 0.0372 |
|  | *Ischyrocerus* sp. | 16 | 0.0112 |
|  | *Janira alta* | 2 | 0.008 |
|  | *Lafoea* sp. | 2 | 0.0118 |
|  | *Leptocheirus pinguis* | 2 | 0.0056 |
|  | *Maera danae* | 6 | 0.1118 |
|  | *Margarites striatus* | 20 | 0.4906 |
|  | *Mediomastus ambiseta* | 14 | 0.0692 |
|  | *Modiolus modiolus* | 14 | 250.5474 |
|  | *Moelleria costulata* | 10 | 0.0132 |
|  | *Musculus* sp. | 4 | 0.0098 |
|  | Mytiloidea | 82 | 0.1868 |
|  | *Myxicola infundibulum* | 2 | 0.18 |
|  | Nemertea | 22 | 0.2532 |
|  | *Nephtys caeca* | 4 | 6.1 |
|  | *Nereis* sp. | 10 | 0.0796 |
|  | *Nicomache lumbricalis* | 4 | 0.9002 |
|  | Nudibranchia Group 4b | 2 | 0.0024 |
|  | Oligochaeta | 18 | 0.0004 |
|  | *Ophiopholis aculeata* | 16 | 0.0078 |
|  | *Ophiura robusta* | 92 | 0.5634 |
|  | *Ophiura sarsi* | 2 | 0.0012 |
|  | *Orbinia swani* | 2 | 0.0208 |
|  | *Owenia fusiformis* | 2 | 0.0338 |
|  | *Paraonis* sp. | 2 | 0.0502 |
|  | *Parougia eliasoni* | 2 | 0.0022 |
|  | *Parvicardium pinnulatum* | 6 | 0.4342 |
|  | *Photis* sp. | 2 | 0.0054 |
|  | *Phoxocephalus holbolli* | 10 | 0.014 |
|  | *Phyllodoce groenlandica* | 2 | 0.223 |
|  | *Phyllodoce maculata* | 26 | 0.0782 |
|  | *Phyllodoce mucosa* | 4 | 0.0084 |
|  | Platyhelminthes | 2 | 0.0226 |
|  | *Pleusymtes glaber* | 6 | 0.0062 |
|  | *Polycirrus* sp. | 14 | 0.2832 |
|  | *Polydora concharum* | 8 | 0.0082 |
|  | Polygordiidae | 8 | 0.0114 |
|  | Porifera | 4 | 0.1238 |
|  | *Prionospio steenstrupi* | 2 | 0.0062 |
|  | *Protomedeia fasciata* | 40 | 0.0344 |
|  | *Psolus* sp. | 24 | 0.0922 |
|  | *Puncturella noachina* | 6 | 0.0424 |
|  | *Rhizocaulus verticillatus* | 2 | 0.0262 |
|  | Sabellidae | 12 | 0.0086 |
|  | *Scalibregma inflatum* | 2 | 0.0684 |
|  | *Scolelepis* sp. A | 2 | 0.0376 |
|  | *Scoloplos armiger* | 6 | 0.563 |
|  | *Scrupocellaria scabra* | 2 | 0.0808 |
|  | *Sertularia* sp. | 2 | 0.0148 |
|  | *Sertularia tricuspidatus* | 2 | 0.0004 |
|  | *Spio filicornis* | 30 | 0.0934 |
|  | *Stenosemus albus* | 10 | 0.3338 |
|  | *Strongylocentrotus* sp. | 20 | 0.2238 |
|  | Syllidae | 2 | 0.0002 |
|  | *Tharyx* sp. | 50 | 0.1384 |
|  | *Thelepus cincinnatus* | 52 | 4.4578 |
|  | *Tonicella rubra* | 16 | 0.6192 |
|  | *Travisia* sp. | 8 | 0.6154 |
|  | *Tricellaria gracilis* | 2 | 0.0088 |
|  | Turridae | 2 | 0.01 |
|  | *Unciola irrorata* | 882 | 5.211 |
| vg43 | Actiniaria (burrowing) | 8 | 1.8434 |
|  | *Anonyx sarsi* | 6 | 0.0264 |
|  | *Apherusa* sp. | 72 | 0.0304 |
|  | *Aricidea* sp. B | 2 | 0.0034 |
|  | *Chlamys islandica* | 2 | 0.0106 |
|  | *Cistenides granulata* | 2 | 0.0732 |
|  | *Crangon septemspinosa* | 2 | 0.0146 |
|  | *Cucumaria frondosa* | 10 | 0.0084 |
|  | *Cyclocardia novangliae* | 2 | 0.037 |
|  | *Cyrtodaria siliqua* | 6 | 0.0186 |
|  | *Edwardsia elegans* | 26 | 2.7868 |
|  | *Euchone papillosa* | 2 | 0.0276 |
|  | *Euclymene zonalis* | 22 | 0.9868 |
|  | *Exogone* sp. | 28 | 0.0048 |
|  | Gastropoda | 34 | 0.0176 |
|  | *Glycera capitata* | 120 | 1.1072 |
|  | *Harmothoe extenuata* | 18 | 0.0426 |
|  | *Hartmania moorei* | 2 | 0.0058 |
|  | Ischyrocerus sp. | 36 | 0.0212 |
|  | *Laphania boecki* | 14 | 0.561 |
|  | *Metopella* sp. | 2 | 0.0002 |
|  | *Modiolus modiolus* | 2 | 0.0034 |
|  | *Moelleria costulata* | 12 | 0.0098 |
|  | Mytiloidea | 34 | 0.1768 |
|  | Nemertea | 50 | 0.0284 |
|  | *Nereis* sp. | 2 | 0.0542 |
|  | Oligochaeta | 36 | 0.068 |
|  | *Ophiura robusta* | 44 | 0.1704 |
|  | *Paraonis* sp. | 4 | 0.002 |
|  | *Parougia eliasoni* | 2 | 0.0048 |
|  | *Pholoe tecta* | 44 | 0.0164 |
|  | *Pleustes panoplus* | 2 | 0.006 |
|  | *Pleusymtes glaber* | 182 | 0.121 |
|  | *Polycirrus* sp. | 4 | 0.0898 |
|  | Polygordiidae | 38 | 0.0892 |
|  | *Protodorvillea gaspeensis* | 28 | 0.0062 |
|  | *Protomedeia fasciata* | 20 | 0.0068 |
|  | *Rhizocaulus verticillatus* | 2 | 0.0054 |
|  | *Scoloplos armiger* | 2 | 0.0288 |
|  | *Serripes groenlandicus* | 6 | 0.0014 |
|  | *Spio filicornis* | 2 | 0.018 |
|  | *Strongylocentrotus* sp. | 8 | 0.0484 |
|  | Syllidae | 14 | 0.004 |
|  | *Syrrhoe crenulata* | 4 | 0.0392 |
|  | *Tharyx* sp. | 2 | 0.0034 |
|  | *Thracia* sp. | 2 | 0.0004 |
|  | *Unciola irrorata* | 112 | 0.355 |
| vg44 | *Acaulis primarius* | 4 | 0.0014 |
|  | Actiniaria (burrowing) | 96 | 4.8524 |
|  | Actiniaria (non burrowing) | 2 | 0.0028 |
|  | *Aglaophamus circinata* | 2 | 0.5842 |
|  | *Ampharete finmarchica* | 58 | 0.346 |
|  | *Anonyx sarsi* | 12 | 0.0582 |
|  | *Arcteobia anticostiensis* | 2 | 0.012 |
|  | *Aricidea catherinae* | 4 | 0.0008 |
|  | Ascidiacea (solitary) | 26 | 0.2622 |
|  | Buccinidae | 4 | 0.0626 |
|  | Campanulariidae | 2 | 0.011 |
|  | *Caprella* sp. | 2 | 0.0002 |
|  | *Chaetozone* sp. A | 2 | 0.0004 |
|  | *Chone* sp. | 6 | 0.0126 |
|  | *Clymenura borealis* | 2 | 0.0114 |
|  | *Crangon septemspinosa* | 4 | 0.026 |
|  | *Cucumaria frondosa* | 4 | 0.0288 |
|  | *Cyrtodaria siliqua* | 8 | 0.0544 |
|  | *Diaphana minuta* | 2 | 0.0016 |
|  | *Dyopedos* sp. | 6 | 0.001 |
|  | *Echinarachnius parma* | 20 | 0.027 |
|  | *Edwardsia elegans* | 14 | 1.3562 |
|  | *Euchone papillosa* | 28 | 0.1306 |
|  | *Euclymene zonalis* | 766 | 5.1896 |
|  | *Euspira* sp. | 2 | 0.002 |
|  | *Exogone* sp. | 8 | 0.007 |
|  | *Glycera capitata* | 72 | 1.6748 |
|  | *Goniadella gracilis* | 2 | 0.049 |
|  | *Harmothoe extenuata* | 4 | 0.0172 |
|  | *Harmothoe imbricata* | 2 | 0.0564 |
|  | *Ischyrocerus* sp. | 6 | 0.0254 |
|  | *Laphania boecki* | 2 | 0.0414 |
|  | *Lumbrinerides acuta* | 14 | 0.2864 |
|  | *Monoculodes* sp. | 6 | 0.0466 |
|  | Mytiloidea | 18 | 0.0604 |
|  | Nemertea | 212 | 0.1856 |
|  | Nephtyidae sp. juvenile | 4 | 0.0102 |
|  | *Nephtys caeca* | 2 | 4.1426 |
|  | *Nicolea venusula* | 2 | 0.011 |
|  | Oligochaeta | 330 | 0.1804 |
|  | *Ophelia limacina* | 332 | 0.1806 |
|  | *Ophiura* sp. juvenile | 4 | 0.0028 |
|  | Ostracoda | 2 | 0.0002 |
|  | *Paraonis* sp. | 20 | 0.0364 |
|  | *Parougia eliasoni* | 6 | 0.002 |
|  | *Parvicardium pinnulatum* | 6 | 0.1074 |
|  | *Pholoe tecta* | 6 | 0.002 |
|  | *Phyllodoce maculata* | 2 | 0.0002 |
|  | Platyhelminthes | 118 | 0.0178 |
|  | *Pleusymtes glaber* | 72 | 0.0472 |
|  | *Polydora caulleryi* | 4 | 0.0262 |
|  | Polygordiidae | 1038 | 5.2388 |
|  | *Protodorvillea kefersteini* | 4 | 0.0074 |
|  | *Protomedeia fasciata* | 50 | 0.0298 |
|  | *Psolus* sp. | 2 | 0.0002 |
|  | *Serripes groenlandicus* | 16 | 0.018 |
|  | *Spio filicornis* | 32 | 0.1364 |
|  | Syllidae | 70 | 0.0114 |
|  | *Syrrhoe crenulata* | 2 | 0.004 |
|  | *Tharyx* sp. | 2 | 0.0288 |
|  | *Thracia* sp. | 2 | 0.0008 |
|  | *Unciola irrorata* | 174 | 0.7596 |
| vg45 | Actiniaria (burrowing) | 2 | 0.0284 |
|  | *Aglaophamus circinata* | 2 | 0.196 |
|  | *Ampharete finmarchica* | 6 | 0.0288 |
|  | *Anonyx sarsi* | 2 | 0.0148 |
|  | *Arctica islandica* | 4 | 0.0426 |
|  | *Aricidea* sp. B | 2 | 0.002 |
|  | *Aricidea wassi* | 4 | 0.0312 |
|  | Ascidiacea (solitary) | 8 | 0.022 |
|  | *Capitella capitata* | 2 | 0.001 |
|  | *Chaetozone* sp. A | 8 | 0.0226 |
|  | *Chiridotea tuftsii* | 6 | 0.0176 |
|  | *Cirolana polita* | 12 | 0.5674 |
|  | *Clymenura borealis* | 14 | 0.672 |
|  | *Crangon septemspinosa* | 16 | 0.2046 |
|  | *Cylichna alba* | 6 | 0.3374 |
|  | *Cyrtodaria siliqua* | 2 | 137.12 |
|  | *Dyopedos* sp. | 2 | 0.0024 |
|  | *Echinarachnius parma* | 96 | 548.7788 |
|  | *Edotea montosa* | 2 | 0.0124 |
|  | *Edwardsia elegans* | 18 | 0.1034 |
|  | *Euchone papillosa* | 2 | 0.0032 |
|  | *Euclymene zonalis* | 8 | 0.0134 |
|  | *Euspira* sp. | 4 | 0.0158 |
|  | *Exogone* sp. | 6 | 0.0002 |
|  | *Hippomedon serratus* | 8 | 0.0168 |
|  | *Laonice cirrata* | 2 | 0.0906 |
|  | *Levensinea gracilis* | 12 | 0.0046 |
|  | *Lumbrinerides acuta* | 60 | 0.7456 |
|  | *Mactromeris polynyma* | 6 | 192.1062 |
|  | *Monoculodes* sp. | 2 | 0.006 |
|  | Nemertea | 6 | 0.1056 |
|  | *Nephtys bucera* | 2 | 0.0636 |
|  | *Nephtys caeca* | 2 | 0.6582 |
|  | *Nereis* sp. | 2 | 0.0876 |
|  | *Notomastus latericeus* | 4 | 0.08 |
|  | Oligochaeta | 8 | 0.0286 |
|  | *Ophelia limacina* | 4 | 0.0712 |
|  | *Ophiura sarsi* | 12 | 0.0068 |
|  | *Orbinia swani* | 6 | 0.8582 |
|  | *Paraonis* sp. | 4 | 0.003 |
|  | *Parougia eliasoni* | 2 | 0.0002 |
|  | *Parvicardium pinnulatum* | 2 | 0.0042 |
|  | *Periploma leanum* | 6 | 0.0776 |
|  | *Phoxocephalus holbolli* | 8 | 0.0442 |
|  | *Polycirrus* sp. | 2 | 0.013 |
|  | *Protodorvillea gaspeensis* | 2 | 0.0002 |
|  | *Protomedeia fasciata* | 2 | 0.0022 |
|  | *Psammonyx* sp. | 6 | 0.0096 |
|  | *Scoloplos armiger* | 2 | 0.1174 |
|  | *Serripes groenlandicus* | 66 | 0.0916 |
|  | *Spio filicornis* | 10 | 0.0934 |
|  | *Spiophanes bombyx* | 18 | 0.2248 |
|  | *Tharyx* sp. | 6 | 0.0258 |
|  | *Thracia* sp. | 2 | 0.4262 |
|  | *Travisia* sp. | 6 | 0.0302 |
|  | *Unciola irrorata* | 28 | 0.1296 |
| vg46 | *Acaulis primarius* | 4 | 0.0022 |
|  | Actiniaria (burrowing) | 8 | 0.0786 |
|  | *Ampharete finmarchica* | 22 | 0.0476 |
|  | *Anonyx sarsi* | 12 | 0.0772 |
|  | *Arctica islandica* | 2 | 0.0456 |
|  | Ascidiacea (solitary) | 16 | 0.1118 |
|  | *Capitella capitata* | 4 | 0.0132 |
|  | *Chaetozone* sp. A | 28 | 0.0314 |
|  | *Clymenura borealis* | 8 | 0.7876 |
|  | *Crangon septemspinosa* | 10 | 0.137 |
|  | *Cylichna alba* | 10 | 0.4582 |
|  | *Cyrtodaria siliqua* | 6 | 132.2504 |
|  | *Dyopedos* sp. | 2 | 0.0002 |
|  | *Echinarachnius parma* | 48 | 268.3192 |
|  | *Edwardsia elegans* | 44 | 0.2224 |
|  | *Ensis directus* | 4 | 0.6594 |
|  | *Euchone papillosa* | 6 | 0.017 |
|  | *Euclymene zonalis* | 36 | 0.0336 |
|  | *Euspira* sp. | 2 | 0.0354 |
|  | *Exogone* sp. | 10 | 0.0058 |
|  | *Glycera capitata* | 2 | 0.0094 |
|  | *Hippomedon serratus* | 6 | 0.0144 |
|  | *Leptognathia* sp. | 4 | 0.001 |
|  | *Levensinea gracilis* | 12 | 0.0082 |
|  | *Lumbrinerides acuta* | 48 | 0.388 |
|  | *Mactromeris polynyma* | 2 | 31.1 |
|  | Nemertea | 8 | 0.1342 |
|  | *Nephtys bucera* | 6 | 0.5258 |
|  | *Nephtys caeca* | 4 | 0.97 |
|  | *Nothria conchylega* | 2 | 0.0002 |
|  | *Notomastus latericeus* | 2 | 0.043 |
|  | Oligochaeta | 24 | 0.0192 |
|  | *Ophelia limacina* | 4 | 0.0322 |
|  | *Orbinia swani* | 4 | 0.191 |
|  | *Pagurus* sp. | 2 | 0.027 |
|  | *Paraonis* sp. | 8 | 0.0072 |
|  | *Periploma leanum* | 4 | 0.052 |
|  | *Photis* sp. | 2 | 0.0008 |
|  | *Pleusymtes glaber* | 2 | 0.0022 |
|  | Polygordiidae | 70 | 0.0264 |
|  | *Protodorvillea kefersteini* | 2 | 0.0002 |
|  | *Protomedeia fasciata* | 10 | 0.0048 |
|  | *Psammonyx* sp. | 8 | 0.0548 |
|  | *Scolelepis* sp. A | 2 | 0.0584 |
|  | *Serripes groenlandicus* | 10 | 0.0056 |
|  | *Solariella obscura* | 2 | 0.011 |
|  | *Spio filicornis* | 6 | 0.012 |
|  | *Spiophanes bombyx* | 6 | 0.1278 |
|  | Syllidae | 6 | 0.0002 |
|  | *Tharyx* sp. | 2 | 0.0026 |
|  | *Unciola irrorata* | 30 | 0.149 |
| vg47 | *Ampharete finmarchica* | 12 | 0.0494 |
|  | *Anonyx sarsi* | 2 | 0.0026 |
|  | *Arctica islandica* | 6 | 0.2674 |
|  | *Aricidea wassi* | 4 | 0.0158 |
|  | Ascidiacea (solitary) | 34 | 0.4434 |
|  | *Chaetozone* sp. A | 12 | 0.0304 |
|  | *Clymenura borealis* | 20 | 2.7422 |
|  | *Cyrtodaria siliqua* | 14 | 421.9588 |
|  | *Diaphana minuta* | 6 | 0.0318 |
|  | *Echinarachnius parma* | 54 | 133.327 |
|  | *Edwardsia elegans* | 30 | 0.634 |
|  | *Euchone papillosa* | 8 | 0.0208 |
|  | *Euclymene zonalis* | 18 | 0.037 |
|  | *Exogone* sp. | 8 | 0.0016 |
|  | *Levensinea gracilis* | 8 | 0.0082 |
|  | *Lumbrinerides acuta* | 30 | 0.3234 |
|  | Mytiloidea | 2 | 0.2264 |
|  | Nemertea | 14 | 0.088 |
|  | *Nephtys bucera* | 4 | 0.2812 |
|  | *Nephtys caeca* | 4 | 2.091 |
|  | Oligochaeta | 8 | 0.0132 |
|  | *Ophelia limacina* | 2 | 0.3294 |
|  | *Orbinia swani* | 2 | 0.927 |
|  | *Paraonis* sp. | 4 | 0.006 |
|  | *Parougia eliasoni* | 2 | 0.0008 |
|  | *Phoxocephalus holbolli* | 4 | 0.0296 |
|  | *Polycirrus* sp. | 4 | 0.0554 |
|  | Polygordiidae | 22 | 0.0072 |
|  | *Protomedeia fasciata* | 2 | 0.0034 |
|  | *Serripes groenlandicus* | 2 | 0.0046 |
|  | *Spio filicornis* | 6 | 0.0138 |
|  | *Spiophanes bombyx* | 10 | 0.156 |
|  | Syllidae | 6 | 0.0004 |
|  | *Tharyx* sp. | 4 | 0.0152 |
|  | *Unciola irrorata* | 4 | 0.019 |
| vg48 | Actiniaria (burrowing) | 20 | 2.0952 |
|  | Actiniaria (non burrowing) | 2 | 0.0008 |
|  | *Ampharete finmarchica* | 38 | 0.0936 |
|  | *Anonyx sarsi* | 32 | 0.1276 |
|  | *Arctica islandica* | 14 | 1.0108 |
|  | *Aricidea catherinae* | 2 | 0.0172 |
|  | *Aricidea* sp. B | 2 | 0.0142 |
|  | Ascidiacea (solitary) | 48 | 1.6184 |
|  | Campanulariidae | 2 | 0.0098 |
|  | *Chaetozone* sp. A | 110 | 0.3628 |
|  | *Clymenura borealis* | 16 | 2.8472 |
|  | *Colus stimpsoni* | 4 | 55.92 |
|  | *Cyrtodaria siliqua* | 10 | 0.0784 |
|  | *Diaphana minuta* | 4 | 0.0764 |
|  | *Diastylis sculpta* | 2 | 0.0022 |
|  | *Dyopedos* sp. | 2 | 0.0004 |
|  | *Echinarachnius parma* | 50 | 317.4866 |
|  | *Edotea montosa* | 8 | 0.0152 |
|  | *Edwardsia elegans* | 52 | 1.289 |
|  | *Euchone papillosa* | 44 | 0.1966 |
|  | *Euclymene zonalis* | 170 | 1.506 |
|  | *Euspira* sp. | 2 | 0.355 |
|  | *Exogone* sp. | 148 | 0.0482 |
|  | *Glycera capitata* | 14 | 0.0954 |
|  | *Goniadella gracilis* | 26 | 0.076 |
|  | *Hippomedon serratus* | 4 | 0.0102 |
|  | *Ischyrocerus* sp. | 22 | 0.012 |
|  | *Laphania boecki* | 2 | 0.0016 |
|  | *Leptognathia* sp. | 2 | 0.0004 |
|  | *Levensinea gracilis* | 14 | 0.007 |
|  | *Lumbrinerides acuta* | 182 | 2.5716 |
|  | *Mactromeris polynyma* | 4 | 2.0324 |
|  | Mytiloidea | 6 | 0.018 |
|  | Nemertea | 172 | 0.0654 |
|  | *Nephtys bucera* | 4 | 0.5574 |
|  | *Nephtys caeca* | 2 | 0.6924 |
|  | *Nereis* sp. | 2 | 0.0122 |
|  | Oligochaeta | 196 | 0.423 |
|  | *Ophelia limacina* | 8 | 0.2368 |
|  | *Orbinia swani* | 2 | 0.0966 |
|  | *Paraonis* sp. | 24 | 0.0154 |
|  | *Paredwardsia arenaria* | 2 | 0.3242 |
|  | *Parougia eliasoni* | 20 | 0.0116 |
|  | *Parvicardium pinnulatum* | 6 | 0.7188 |
|  | *Periploma leanum* | 4 | 2.3458 |
|  | *Phoxocephalus holbolli* | 6 | 0.014 |
|  | *Pleusymtes glaber* | 8 | 0.0068 |
|  | *Polycirrus* sp. | 10 | 0.0174 |
|  | Polygordiidae | 472 | 0.2034 |
|  | *Protodorvillea kefersteini* | 36 | 0.023 |
|  | *Protomedeia fasciata* | 60 | 0.0316 |
|  | *Scolelepis* sp. A | 2 | 0.0482 |
|  | *Serripes groenlandicus* | 24 | 0.0508 |
|  | *Spio filicornis* | 6 | 0.0242 |
|  | Syllidae | 128 | 0.0312 |
|  | *Tharyx* sp. | 2 | 0.0118 |
|  | *Unciola irrorata* | 110 | 0.1816 |
| vg49 | Actiniaria (burrowing) | 10 | 0.7676 |
|  | *Ampharete finmarchica* | 16 | 0.019 |
|  | *Anonyx sarsi* | 2 | 0.0026 |
|  | *Arctica islandica* | 8 | 462.0918 |
|  | Ascidiacea (solitary) | 12 | 0.5474 |
|  | Capitellidae | 2 | 0.0048 |
|  | *Chaetozone setosa* | 10 | 0.035 |
|  | *Chaetozone* sp. A | 24 | 0.068 |
|  | *Clymenura borealis* | 14 | 1.6482 |
|  | *Colus stimpsoni* | 4 | 17.6848 |
|  | *Cyrtodaria siliqua* | 20 | 189 |
|  | *Diaphana minuta* | 6 | 0.1878 |
|  | *Diastylis sculpta* | 2 | 0.0014 |
|  | *Dyopedos* sp. | 2 | 0.0016 |
|  | *Echinarachnius parma* | 52 | 173.2372 |
|  | *Edwardsia elegans* | 10 | 0.2964 |
|  | *Euchone papillosa* | 2 | 0.0162 |
|  | *Euclymene zonalis* | 12 | 0.0208 |
|  | *Euspira* sp. | 2 | 0.015 |
|  | *Exogone* sp. | 10 | 0.0088 |
|  | *Glycera capitata* | 2 | 0.0256 |
|  | *Hippomedon serratus* | 4 | 0.012 |
|  | *Ischyrocerus* sp. | 2 | 0.0018 |
|  | *Leptognathia* sp. | 2 | 0.0012 |
|  | *Lumbrinerides acuta* | 50 | 0.4928 |
|  | Nemertea | 2 | 0.0002 |
|  | Nephtyidae sp. juvenile | 4 | 0.0672 |
|  | *Nephtys bucera* | 2 | 0.1786 |
|  | *Notomastus latericeus* | 4 | 0.0662 |
|  | Oligochaeta | 12 | 0.053 |
|  | *Ophelia limacina* | 4 | 0.0302 |
|  | *Orbinia swani* | 4 | 0.9342 |
|  | *Paraonis* sp. | 4 | 0.0078 |
|  | *Parougia eliasoni* | 4 | 0.0018 |
|  | *Periploma leanum* | 2 | 0.4782 |
|  | *Phoxocephalus holbolli* | 8 | 0.0222 |
|  | Polygordiidae | 8 | 0.0026 |
|  | *Psammonyx* sp. | 4 | 0.0036 |
|  | *Serripes groenlandicus* | 6 | 0.0226 |
|  | *Spio filicornis* | 2 | 0.0044 |
|  | Syllidae | 2 | 0.0002 |
|  | *Travisia* sp. | 2 | 0.0036 |
|  | *Unciola irrorata* | 2 | 0.0032 |
| vg50 | Actiniaria (burrowing) | 4 | 1.8016 |
|  | *Aeginina longicornis* | 2 | 0.0026 |
|  | *Aglaophamus circinata* | 20 | 1.855 |
|  | *Ampharete finmarchica* | 106 | 0.5112 |
|  | *Anonyx sarsi* | 4 | 0.0122 |
|  | *Arctica islandica* | 26 | 382.9156 |
|  | *Aricidea catherinae* | 4 | 0.004 |
|  | *Aricidea wassi* | 8 | 0.0308 |
|  | Ascidiacea (solitary) | 122 | 19.4572 |
|  | *Capitella capitata* | 20 | 0.045 |
|  | *Chaetozone setosa* | 6 | 0.061 |
|  | *Chaetozone* sp. A | 8 | 0.0166 |
|  | *Cirolana polita* | 154 | 0.6312 |
|  | *Cistenides granulata* | 2 | 0.0882 |
|  | *Clymenura borealis* | 90 | 1.9252 |
|  | *Cylichna alba* | 2 | 0.0016 |
|  | *Cyrtodaria siliqua* | 8 | 171.63 |
|  | *Diaphana minuta* | 4 | 0.034 |
|  | *Echinarachnius parma* | 138 | 264.7182 |
|  | *Ensis directus* | 2 | 4.94 |
|  | *Euchone papillosa* | 38 | 0.262 |
|  | *Euclymene zonalis* | 44 | 0.4414 |
|  | *Eunucula tenuis* | 2 | 0.015 |
|  | *Euspira* sp. | 12 | 1.1366 |
|  | Hydrozoa Athecata | 2 | 0.0176 |
|  | *Leptognathia* sp. | 2 | 0.001 |
|  | *Levensinea gracilis* | 2 | 0.0014 |
|  | *Lumbrinerides acuta* | 14 | 0.2744 |
|  | Nassarius sp. | 4 | 0.6242 |
|  | Nemertea | 8 | 0.8582 |
|  | *Nephtys bucera* | 2 | 1.5854 |
|  | *Nereis* sp. | 2 | 0.0334 |
|  | *Notomastus latericeus* | 12 | 0.2672 |
|  | Oligochaeta | 4 | 0.01 |
|  | *Ophelia limacina* | 20 | 3.905 |
|  | *Orbinia swani* | 4 | 0.4218 |
|  | *Parougia eliasoni* | 2 | 0.0002 |
|  | *Parvicardium pinnulatum* | 2 | 0.532 |
|  | *Periploma leanum* | 38 | 0.7586 |
|  | *Phoronis* sp. | 2 | 0.0014 |
|  | *Phoxocephalus holbolli* | 8 | 0.0316 |
|  | *Platyhelminthes* | 2 | 0.0782 |
|  | *Pleusymtes glaber* | 2 | 0.0016 |
|  | *Polycirrus* sp. | 14 | 0.0672 |
|  | *Polydora socialis* | 2 | 0.001 |
|  | Sabellidae | 4 | 0.0008 |
|  | *Scolelepis* sp. A | 2 | 0.053 |
|  | *Scolelepis squamata* | 6 | 0.1016 |
|  | *Scoloplos armiger* | 4 | 0.0372 |
|  | *Spio filicornis* | 20 | 0.0916 |
|  | *Spiophanes bombyx* | 8 | 0.152 |
|  | *Tharyx* sp. | 20 | 0.109 |
|  | *Unciola irrorata* | 52 | 0.3132 |
| vg51 | Actiniaria (burrowing) | 6 | 0.9708 |
|  | *Aglaophamus circinata* | 34 | 4.9782 |
|  | *Ampharete finmarchica* | 66 | 0.2604 |
|  | *Anonyx sarsi* | 2 | 0.0106 |
|  | *Aphrodita hastata* | 4 | 32.2 |
|  | *Arctica islandica* | 2 | 0.1946 |
|  | *Aricidea catherinae* | 16 | 0.0674 |
|  | *Aricidea wassi* | 10 | 0.0626 |
|  | Ascidiacea (solitary) | 42 | 5.7014 |
|  | Capitellidae | 18 | 0.0598 |
|  | Cerianthidae | 4 | 3.7918 |
|  | *Chaetozone setosa* | 12 | 0.0534 |
|  | *Chaetozone* sp. A | 24 | 0.0464 |
|  | *Cirolana polita* | 6 | 0.4136 |
|  | *Cistenides granulata* | 10 | 0.1636 |
|  | *Clymenura borealis* | 70 | 7.6572 |
|  | *Crangon septemspinosa* | 8 | 0.105 |
|  | *Cucumaria frondosa* | 2 | 0.0022 |
|  | *Cyrtodaria siliqua* | 6 | 0.0196 |
|  | *Echinarachnius parma* | 36 | 214.39 |
|  | *Edotea montosa* | 6 | 0.0178 |
|  | *Edwardsia elegans* | 2 | 0.095 |
|  | *Euchone papillosa* | 22 | 0.1358 |
|  | *Euclymene zonalis* | 104 | 1.959 |
|  | *Eucratea loricata* | 2 | 0.0246 |
|  | *Euspira* sp. | 6 | 0.4376 |
|  | *Glycera dibranchiata* | 6 | 0.385 |
|  | *Goniadella gracilis* | 2 | 0.0002 |
|  | *Hippomedon serratus* | 12 | 0.0138 |
|  | *Levensinea gracilis* | 2 | 0.0018 |
|  | *Lumbrinerides acuta* | 4 | 0.0784 |
|  | *Lumbrineris fragilis* | 8 | 2.1922 |
|  | *Monoculodes* sp. | 2 | 0.0026 |
|  | Nemertea | 14 | 11.056 |
|  | *Nephtys bucera* | 4 | 0.1624 |
|  | *Nephtys caeca* | 2 | 0.231 |
|  | *Nereis* sp. | 2 | 0.0432 |
|  | *Notomastus latericeus* | 22 | 0.7508 |
|  | Oligochaeta | 2 | 0.0158 |
|  | *Ophelia limacina* | 8 | 0.5404 |
|  | *Orbinia swani* | 4 | 0.0512 |
|  | *Pagurus* sp. | 2 | 0.0068 |
|  | *Paraonis* sp. | 4 | 0.0056 |
|  | *Parougia eliasoni* | 4 | 0.0008 |
|  | *Periploma leanum* | 18 | 6.6924 |
|  | *Phoxocephalus holbolli* | 4 | 0.011 |
|  | *Phyllodoce mucosa* | 2 | 0.0002 |
|  | *Polycirrus* sp. | 8 | 0.1298 |
|  | Polygordiidae | 6 | 0.003 |
|  | Sabellidae | 2 | 0.0002 |
|  | *Scolelepis* sp. A | 4 | 0.565 |
|  | *Scolelepis squamata* | 4 | 0.0862 |
|  | *Scoloplos armiger* | 2 | 0.0078 |
|  | *Serripes groenlandicus* | 2 | 0.0026 |
|  | *Solariella obscura* | 2 | 0.058 |
|  | *Spio filicornis* | 44 | 0.1902 |
|  | *Spiophanes bombyx* | 6 | 0.0748 |
|  | Syllidae | 2 | 0.0002 |
|  | *Tharyx* sp. | 16 | 0.0378 |
|  | *Unciola irrorata* | 152 | 0.9208 |
| vg52 | *Aglaophamus circinata* | 50 | 3.058 |
|  | *Ampharete acutifrons* | 2 | 0.0068 |
|  | *Ampharete finmarchica* | 30 | 0.148 |
|  | *Aphrodita hastata* | 4 | 0.719 |
|  | *Arcteobia anticostiensis* | 10 | 0.0504 |
|  | *Arctica islandica* | 180 | 682.1152 |
|  | *Aricidea catherinae* | 320 | 0.8734 |
|  | Ascidiacea (solitary) | 2 | 0.0214 |
|  | *Autolytus* sp. | 4 | 0.0028 |
|  | *Brada villosa* | 4 | 0.0288 |
|  | *Bylgides* sp. | 2 | 0.0776 |
|  | Campanulariidae | 2 | 0.0136 |
|  | *Capitella capitata* | 108 | 0.4732 |
|  | Capitellidae | 16 | 0.0816 |
|  | *Chiridotea tuftsii* | 6 | 0.0508 |
|  | *Chone* sp. | 2 | 0.005 |
|  | *Cistenides granulata* | 202 | 0.6168 |
|  | *Clymenura borealis* | 24 | 7.2416 |
|  | *Corophium crassicorne* | 2 | 0.0032 |
|  | *Crangon septemspinosa* | 4 | 0.0416 |
|  | *Cucumaria frondosa* | 6 | 0.0084 |
|  | *Cylichna alba* | 4 | 0.1266 |
|  | *Cyrtodaria siliqua* | 16 | 433.295 |
|  | *Diastylis sculpta* | 4 | 0.0114 |
|  | *Dyopedos* sp. | 4 | 0.015 |
|  | *Echinarachnius parma* | 66 | 147.8136 |
|  | *Edotea montosa* | 18 | 0.0688 |
|  | *Edwardsia elegans* | 276 | 36.688 |
|  | *Eteone longa* | 34 | 0.2604 |
|  | *Euchone papillosa* | 6 | 0.065 |
|  | *Euclymene zonalis* | 8 | 0.6086 |
|  | *Eulalia bilineata* | 2 | 0.0076 |
|  | Gastropoda | 2 | 0.0058 |
|  | *Gattyana cirrhosa* | 2 | 0.0042 |
|  | *Glycera dibranchiata* | 4 | 1.1478 |
|  | *Goniada maculata* | 6 | 0.126 |
|  | *Leptocheirus pinguis* | 6 | 0.1718 |
|  | *Mactromeris polynyma* | 4 | 0.0254 |
|  | *Mediomastus ambiseta* | 20 | 0.0652 |
|  | *Microphthalmus aberrans* | 250 | 0.0218 |
|  | Nemertea | 10 | 0.8796 |
|  | *Neosabellides* sp. | 10 | 0.0052 |
|  | Nephtyidae sp. juvenile | 12 | 0.0688 |
|  | *Nephtys caeca* | 2 | 3.0226 |
|  | *Nereis* sp. | 10 | 0.1808 |
|  | Oligochaeta | 10 | 0.0088 |
|  | *Ophelia limacina* | 6 | 0.0614 |
|  | *Orbinia swani* | 2 | 0.1226 |
|  | Ostracoda | 2 | 0.0022 |
|  | *Owenia fusiformis* | 10 | 0.0296 |
|  | *Parougia eliasoni* | 4 | 0.004 |
|  | *Parvicardium pinnulatum* | 4 | 0.3204 |
|  | *Pelonaia corrugata* | 4 | 8.3224 |
|  | *Pherusa plumosa* | 8 | 8.4176 |
|  | *Pholoe minuta* | 2 | 0.0064 |
|  | *Phoronis* sp. | 40 | 1.0324 |
|  | *Photis* sp. | 2 | 0.0002 |
|  | *Phyllodoce maculata* | 34 | 0.0038 |
|  | *Phyllodoce mucosa* | 46 | 0.4172 |
|  | Platyhelminthes | 2 | 0.407 |
|  | *Polycirrus* sp. | 8 | 0.1798 |
|  | *Polydora concharum* | 516 | 10.531 |
|  | *Polydora socialis* | 32 | 0.0522 |
|  | Polygordiidae | 4 | 0.001 |
|  | *Protomedeia fasciata* | 2 | 0.0034 |
|  | Sabellidae | 2 | 0.0014 |
|  | *Scoloplos armiger* | 60 | 0.72 |
|  | *Serripes groenlandicus* | 32 | 0.0408 |
|  | *Spio filicornis* | 52 | 0.6028 |
|  | *Spiophanes bombyx* | 136 | 0.832 |
|  | *Sthenelais limicola* | 6 | 1.3048 |
|  | *Tharyx* sp. | 40 | 0.3518 |
|  | *Thracia* sp. | 2 | 0.0088 |
|  | *Thuiaria* sp. | 2 | 0.2062 |
|  | *Unciola irrorata* | 16 | 0.1504 |
| vg53 | *Aglaophamus circinata* | 46 | 7.4552 |
|  | *Ampelisca macrocephala* | 2 | 0.0158 |
|  | *Ampharete acutifrons* | 4 | 0.02 |
|  | *Ampharete finmarchica* | 18 | 0.0802 |
|  | *Aphrodita hastata* | 4 | 0.013 |
|  | *Arcteobia anticostiensis* | 4 | 0.0202 |
|  | *Arctica islandica* | 292 | 729.4862 |
|  | *Aricidea catherinae* | 136 | 0.4524 |
|  | Ascidiacea (solitary) | 268 | 3.4722 |
|  | *Brada villosa* | 4 | 0.105 |
|  | *Bylgides* sp. | 2 | 0.0382 |
|  | Campanulariidae | 2 | 0.0256 |
|  | Capitellidae | 10 | 0.0204 |
|  | Cerianthidae | 4 | 7.2998 |
|  | *Chiridotea tuftsii* | 2 | 0.0352 |
|  | *Cistenides granulata* | 172 | 0.2988 |
|  | *Clymenella torquata* | 10 | 0.3998 |
|  | *Clymenura borealis* | 2 | 0.19 |
|  | *Corophium crassicorne* | 4 | 0.0042 |
|  | *Cucumaria frondosa* | 22 | 0.0388 |
|  | *Cyrtodaria siliqua* | 6 | 253.948 |
|  | *Echinarachnius parma* | 28 | 272.414 |
|  | *Edotea montosa* | 14 | 0.0568 |
|  | *Edwardsia elegans* | 326 | 33.2852 |
|  | *Eteone longa* | 14 | 0.4302 |
|  | *Euchone papillosa* | 12 | 0.0616 |
|  | *Euclymene zonalis* | 2 | 0.0002 |
|  | *Eucratea loricata* | 2 | 0.0034 |
|  | *Eunucula tenuis* | 284 | 1.3778 |
|  | *Euspira* sp. | 2 | 0.0338 |
|  | *Galathowenia oculata* | 6 | 0.0372 |
|  | *Goniada maculata* | 14 | 2.0206 |
|  | Hydrozoa Athecata | 2 | 0.0288 |
|  | *Leptocheirus pinguis* | 2 | 0.0652 |
|  | *Mediomastus ambiseta* | 16 | 0.049 |
|  | *Microphthalmus aberrans* | 18 | 0.002 |
|  | Nemertea | 2 | 0.1766 |
|  | Nephtyidae sp. juvenile | 4 | 0.0002 |
|  | *Nephtys caeca* | 4 | 1.9296 |
|  | *Nereis* sp. | 10 | 0.3996 |
|  | *Ophelina acuminata* | 2 | 0.0096 |
|  | *Orbinia swani* | 2 | 0.0026 |
|  | *Owenia fusiformis* | 32 | 0.0486 |
|  | *Pagurus* sp. | 2 | 0.0308 |
|  | *Parougia eliasoni* | 2 | 0.0002 |
|  | *Parvicardium pinnulatum* | 2 | 0.0104 |
|  | *Pelonaia corrugata* | 18 | 24.7836 |
|  | *Pherusa plumosa* | 10 | 2.2716 |
|  | *Pholoe minuta* | 6 | 0.0152 |
|  | *Phoronis* sp. | 136 | 3.5058 |
|  | *Photis* sp. | 8 | 0.0084 |
|  | *Phyllodoce mucosa* | 46 | 0.5948 |
|  | *Polycirrus* sp. | 10 | 0.5932 |
|  | *Polydora concharum* | 28 | 0.8406 |
|  | *Polydora socialis* | 88 | 1.0462 |
|  | Sabellidae | 38 | 0.0372 |
|  | *Scoloplos armiger* | 52 | 0.864 |
|  | *Serripes groenlandicus* | 32 | 0.0442 |
|  | *Solariella obscura* | 4 | 0.0408 |
|  | *Spio filicornis* | 54 | 0.2942 |
|  | *Spiophanes bombyx* | 84 | 0.4116 |
|  | *Sthenelais limicola* | 2 | 0.4446 |
|  | *Tharyx* sp. | 2 | 0.0292 |
|  | *Thracia* sp. | 4 | 0.013 |
|  | *Thyasira* sp. | 2 | 0.0048 |
|  | *Unciola irrorata* | 6 | 0.01 |
| vg54 | Actiniaria (burrowing) | 2 | 0.0192 |
|  | *Aglaophamus circinata* | 50 | 4.6836 |
|  | *Ampelisca macrocephala* | 2 | 0.0108 |
|  | *Ampharete acutifrons* | 4 | 0.02 |
|  | *Ampharete finmarchica* | 10 | 0.0802 |
|  | *Aporrhais occidentalis* | 2 | 24.9352 |
|  | *Arcteobia anticostiensis* | 6 | 0.0624 |
|  | *Arctica islandica* | 402 | 1555.8246 |
|  | *Aricidea* sp. B | 4 | 0.0008 |
|  | Ascidiacea (solitary) | 142 | 0.2798 |
|  | *Brada villosa* | 4 | 0.22 |
|  | Campanulariidae | 2 | 0.0014 |
|  | *Capitella capitata* | 12 | 0.0182 |
|  | Capitellidae | 4 | 0.006 |
|  | Cerianthidae | 4 | 7.4964 |
|  | *Chiridotea tuftsii* | 4 | 0.0446 |
|  | *Chone* sp. | 2 | 0.005 |
|  | *Cistenides granulata* | 162 | 0.5644 |
|  | *Clymenella torquata* | 10 | 0.853 |
|  | *Clymenura borealis* | 2 | 0.0016 |
|  | *Corophium crassicorne* | 12 | 0.0158 |
|  | *Cucumaria frondosa* | 12 | 0.009 |
|  | *Cyrtodaria siliqua* | 6 | 262.6298 |
|  | *Diastylis sculpta* | 2 | 0.0238 |
|  | *Dyopedos* sp. | 2 | 0.0024 |
|  | *Echinarachnius parma* | 36 | 50.2366 |
|  | *Edotea montosa* | 2 | 0.0198 |
|  | *Edwardsia elegans* | 388 | 19.9744 |
|  | *Ensis directus* | 2 | 5.2132 |
|  | *Eteone longa* | 18 | 0.1244 |
|  | *Euchone papillosa* | 8 | 0.037 |
|  | *Eunucula tenuis* | 126 | 1.24 |
|  | *Gattyana cirrhosa* | 2 | 0.0046 |
|  | *Goniada maculata* | 12 | 0.5534 |
|  | Hydrozoa Athecata | 2 | 0.0036 |
|  | *Leptocheirus pinguis* | 12 | 0.2956 |
|  | *Macoma calcarea* | 2 | 0.0158 |
|  | *Mediomastus ambiseta* | 10 | 0.037 |
|  | *Microphthalmus aberrans* | 16 | 0.0018 |
|  | *Musculus* sp. | 2 | 0.0044 |
|  | Nephtyidae sp. juvenile | 4 | 0.003 |
|  | *Nephtys caeca* | 2 | 0.1214 |
|  | *Nereis* sp. | 4 | 0.0504 |
|  | *Owenia fusiformis* | 14 | 0.0036 |
|  | *Pagurus* sp. | 2 | 4.058 |
|  | *Pelonaia corrugata* | 10 | 6.0672 |
|  | *Pentamera calcigera* | 4 | 4.928 |
|  | *Pherusa plumosa* | 8 | 0.3162 |
|  | *Pholoe tecta* | 2 | 0.1782 |
|  | *Phoronis* sp. | 452 | 0.8196 |
|  | *Photis* sp. | 2 | 0.0008 |
|  | *Phyllodoce maculata* | 4 | 0.0014 |
|  | *Phyllodoce mucosa* | 20 | 0.1564 |
|  | *Podoceropsis* sp. | 6 | 0.0086 |
|  | *Polycirrus* sp. | 14 | 0.1464 |
|  | *Polydora concharum* | 12 | 0.2618 |
|  | *Polydora socialis* | 60 | 0.1754 |
|  | Polygordiidae | 4 | 0.001 |
|  | *Prionospio steenstrupi* | 2 | 0.0024 |
|  | Sabellidae | 6 | 0.0042 |
|  | *Scalibregma inflatum* | 2 | 0.3044 |
|  | *Scoloplos armiger* | 30 | 1.3044 |
|  | *Serripes groenlandicus* | 36 | 0.0498 |
|  | *Siliqua squama* | 4 | 28.2824 |
|  | *Spio filicornis* | 34 | 0.1986 |
|  | *Spiophanes bombyx* | 18 | 0.1458 |
|  | Stenothoidae | 4 | 0.002 |
|  | *Sthenelais limicola* | 2 | 0.2478 |
|  | *Tharyx* sp. | 8 | 0.0694 |
|  | *Thracia* sp. | 12 | 0.8088 |
|  | *Thyasira* sp. | 2 | 0.0036 |
|  | *Unciola irrorata* | 2 | 0.01 |
| vg55 | *Aeginina longicornis* | 2 | 0.017 |
|  | *Aglaophamus circinata* | 130 | 8.5304 |
|  | *Ampharete acutifrons* | 4 | 0.0176 |
|  | *Ampharete finmarchica* | 14 | 0.0482 |
|  | *Aphrodita hastata* | 6 | 1.3004 |
|  | *Arcteobia anticostiensis* | 6 | 0.092 |
|  | *Arctica islandica* | 514 | 165.3342 |
|  | *Aricidea catherinae* | 198 | 0.5184 |
|  | *Aricidea* sp. B | 6 | 0.0084 |
|  | *Asabellides* sp. | 14 | 0.0078 |
|  | Ascidiacea (solitary) | 30 | 0.0498 |
|  | *Autolytus* sp. | 8 | 0.0028 |
|  | Campanulariidae | 2 | 0.0176 |
|  | *Capitella capitata* | 18 | 0.0406 |
|  | Capitellidae | 4 | 0.0016 |
|  | Cerianthidae | 8 | 5.2252 |
|  | *Chaetozone setosa* | 2 | 0.0004 |
|  | *Chiridotea tuftsii* | 12 | 0.0624 |
|  | *Cirratulus cirratus* | 2 | 0.0134 |
|  | *Cistenides granulata* | 152 | 0.1906 |
|  | *Clymenella torquata* | 8 | 0.2198 |
|  | *Clymenura borealis* | 8 | 2.3268 |
|  | *Colus* sp. | 6 | 2.3906 |
|  | *Corophium crassicorne* | 12 | 0.0178 |
|  | *Crangon septemspinosa* | 2 | 0.006 |
|  | *Cucumaria frondosa* | 54 | 0.0322 |
|  | *Cyrtodaria siliqua* | 8 | 0.4712 |
|  | *Diaphana minuta* | 6 | 0.006 |
|  | *Diastylis sculpta* | 8 | 0.0526 |
|  | *Echinarachnius parma* | 90 | 116.1892 |
|  | *Edotea montosa* | 10 | 0.0306 |
|  | *Edwardsia elegans* | 82 | 9.9478 |
|  | *Eteone longa* | 42 | 0.4026 |
|  | *Euchone papillosa* | 8 | 0.0678 |
|  | *Eudorellopsis deformis* | 6 | 0.0046 |
|  | *Euspira* sp. | 2 | 0.1388 |
|  | *Gattyana cirrhosa* | 2 | 0.0004 |
|  | *Goniada maculata* | 22 | 1.2154 |
|  | *Harmothoe imbricata* | 2 | 0.0882 |
|  | *Hydrozoa athecata* | 2 | 0.0012 |
|  | *Leptocheirus pinguis* | 10 | 0.0874 |
|  | *Leptognathia* sp. | 8 | 0.0026 |
|  | *Macoma calcarea* | 2 | 0.0764 |
|  | *Mactromeris polynyma* | 4 | 0.0546 |
|  | *Mediomastus ambiseta* | 14 | 0.0478 |
|  | *Microphthalmus aberrans* | 74 | 0.0116 |
|  | Nemertea | 2 | 0.104 |
|  | Nephtyidae sp. juvenile | 14 | 0.0104 |
|  | *Nephtys caeca* | 12 | 3.7536 |
|  | *Nereis* sp. | 12 | 0.436 |
|  | *Ophelia limacina* | 18 | 0.071 |
|  | *Ophiura sarsi* | 6 | 0.015 |
|  | *Orbinia swani* | 6 | 0.0252 |
|  | Ostracoda | 2 | 0.0004 |
|  | *Owenia fusiformis* | 2 | 0.0002 |
|  | *Parougia eliasoni* | 8 | 0.0018 |
|  | *Parvicardium pinnulatum* | 2 | 0.0758 |
|  | *Pelonaia corrugata* | 2 | 1.608 |
|  | *Pentamera calcigera* | 2 | 0.0644 |
|  | *Pherusa plumosa* | 12 | 1.6534 |
|  | *Pholoe minuta* | 6 | 0.013 |
|  | *Phoronis* sp. | 32 | 0.8062 |
|  | *Photis* sp. | 24 | 0.0096 |
|  | *Phyllodoce maculata* | 12 | 0.003 |
|  | *Phyllodoce mucosa* | 52 | 0.7822 |
|  | Platyhelminthes | 2 | 0.3442 |
|  | *Polycirrus* sp. | 8 | 0.2962 |
|  | *Polydora caulleryi* | 6 | 0.0146 |
|  | *Polydora concharum* | 46 | 1.2106 |
|  | *Polydora socialis* | 224 | 0.524 |
|  | Polygordiidae | 6 | 0.0034 |
|  | *Prionospio steenstrupi* | 6 | 0.0118 |
|  | Sabellidae | 30 | 0.0174 |
|  | *Scoloplos armiger* | 14 | 1.8478 |
|  | *Serripes groenlandicus* | 162 | 0.1422 |
|  | *Siliqua squama* | 2 | 26.84 |
|  | *Spio filicornis* | 60 | 0.258 |
|  | *Spiophanes bombyx* | 294 | 1.798 |
|  | *Sthenelais limicola* | 2 | 0.113 |
|  | *Tharyx* sp. | 18 | 0.0726 |
|  | *Thracia* sp. | 8 | 0.0162 |
|  | *Thuiaria* sp. | 2 | 0.539 |
|  | *Unciola irrorata* | 2 | 0.0088 |
| vg56 | *Acanthohaustorius spinosus* | 2 | 0.011 |
|  | *Aglaophamus circinata* | 42 | 4.0066 |
|  | *Ampharete acutifrons* | 2 | 0.008 |
|  | *Ampharete finmarchica* | 38 | 0.152 |
|  | *Arcteobia anticostiensis* | 8 | 0.02 |
|  | *Arctica islandica* | 314 | 2602.637 |
|  | *Aricidea catherinae* | 104 | 0.3644 |
|  | Ascidiacea (solitary) | 6 | 0.0078 |
|  | *Bylgides* sp. | 2 | 0.095 |
|  | *Capitella capitata* | 8 | 0.0162 |
|  | Capitellidae | 20 | 0.0582 |
|  | *Chaetozone setosa* | 12 | 0.009 |
|  | *Chiridotea tuftsii* | 2 | 0.0248 |
|  | *Cirolana polita* | 4 | 0.0354 |
|  | *Cistenides granulata* | 18 | 0.2782 |
|  | *Clymenura borealis* | 56 | 10.9864 |
|  | *Colus* sp. | 2 | 0.974 |
|  | *Corophium crassicorne* | 2 | 0.0044 |
|  | *Cucumaria frondosa* | 2 | 0.0004 |
|  | *Cyrtodaria siliqua* | 18 | 309.1548 |
|  | *Echinarachnius parma* | 46 | 301.5492 |
|  | *Edwardsia elegans* | 18 | 1.2124 |
|  | *Eteone longa* | 84 | 1.2898 |
|  | *Euchone papillosa* | 8 | 0.0286 |
|  | *Euclymene zonalis* | 4 | 0.1916 |
|  | *Eudendrium* sp. | 2 | 0.0056 |
|  | *Eudorellopsis deformis* | 8 | 0.0068 |
|  | *Exogone* sp. | 2 | 0.0002 |
|  | *Goniada maculata* | 4 | 0.0572 |
|  | *Mediomastus ambiseta* | 10 | 0.15 |
|  | *Microphthalmus aberrans* | 4 | 0.0002 |
|  | Nemertea | 6 | 2.0516 |
|  | Nephtyidae sp. juvenile | 4 | 0.0022 |
|  | *Nephtys caeca* | 2 | 2.2346 |
|  | *Nereis* sp. | 4 | 0.02 |
|  | *Notomastus latericeus* | 4 | 0.147 |
|  | Oligochaeta | 30 | 0.0468 |
|  | *Ophelia limacina* | 22 | 0.166 |
|  | *Ophelina acuminata* | 2 | 0.1036 |
|  | *Orbinia swani* | 16 | 1.0188 |
|  | *Pherusa plumosa* | 4 | 0.0048 |
|  | *Phyllodoce mucosa* | 12 | 0.563 |
|  | *Polycirrus* sp. | 6 | 0.5016 |
|  | *Polydora socialis* | 210 | 0.4986 |
|  | *Priscillina armata* | 10 | 0.1634 |
|  | *Pseudounicola obliquua* | 4 | 0.0038 |
|  | *Scoloplos armiger* | 100 | 1.4676 |
|  | *Serripes groenlandicus* | 26 | 0.029 |
|  | *Sertularia* sp. | 2 | 0.0002 |
|  | *Siliqua squama* | 2 | 0.0132 |
|  | *Spio filicornis* | 32 | 0.08 |
|  | *Spiophanes bombyx* | 166 | 1.3736 |
|  | *Sthenelais limicola* | 2 | 0.3176 |
|  | *Tharyx* sp. | 10 | 0.0526 |
|  | *Travisia* sp. | 2 | 1.2672 |
|  | *Unciola irrorata* | 2 | 0.0034 |
| vg57 | *Acanthohaustorius spinosus* | 40 | 0.2162 |
|  | *Aeginina longicornis* | 14 | 0.0622 |
|  | *Aglaophamus circinata* | 48 | 4.1928 |
|  | *Ampelisca macrocephala* | 2 | 0.0158 |
|  | *Ampharete finmarchica* | 114 | 0.4116 |
|  | *Anonyx sarsi* | 2 | 0.0024 |
|  | *Arcteobia anticostiensis* | 12 | 0.0364 |
|  | *Arctica islandica* | 48 | 489.382 |
|  | *Aricidea catherinae* | 10 | 0.0134 |
|  | *Aricidea wassi* | 4 | 0.003 |
|  | Ascidiacea (solitary) | 42 | 1.4894 |
|  | *Autolytus* sp. | 10 | 0.006 |
|  | Campanulariidae | 2 | 0.0002 |
|  | Capitellidae | 10 | 0.0086 |
|  | *Caprella* sp. | 2 | 0.0042 |
|  | *Chaetozone setosa* | 14 | 0.042 |
|  | *Chaetozone* sp. A | 2 | 0.0028 |
|  | *Chiridotea tuftsii* | 8 | 0.0596 |
|  | *Chone* sp. | 10 | 0.0042 |
|  | *Cirolana polita* | 10 | 0.2794 |
|  | *Clymenura borealis* | 144 | 19.881 |
|  | *Colus* sp. | 2 | 0.5776 |
|  | *Corophium crassicorne* | 2 | 0.0032 |
|  | *Corymorpha pendula* | 2 | 0.01 |
|  | *Crangon septemspinosa* | 2 | 0.0112 |
|  | *Cylichna alba* | 2 | 0.044 |
|  | *Cyrtodaria siliqua* | 8 | 161.7062 |
|  | *Dyopedos* sp. | 2 | 0.0042 |
|  | *Echinarachnius parma* | 42 | 633.3216 |
|  | *Edotea montosa* | 2 | 0.0154 |
|  | *Edwardsia sipunculoides* | 6 | 0.6146 |
|  | *Ensis directus* | 2 | 0.5066 |
|  | *Ericthonius rubricornis* | 2 | 0.0002 |
|  | *Eteone longa* | 148 | 1.5644 |
|  | *Euchone papillosa* | 14 | 0.037 |
|  | *Euclymene zonalis* | 4 | 0.0104 |
|  | *Eudorellopsis deformis* | 6 | 0.0042 |
|  | *Euspira* sp. | 2 | 0.0216 |
|  | *Goniadella gracilis* | 2 | 0.0008 |
|  | *Hippomedon serratus* | 2 | 0.1424 |
|  | *Hydrozoa athecata* | 2 | 0.0026 |
|  | *Leptocheirus pinguis* | 2 | 0.0446 |
|  | *Mactromeris polynyma* | 2 | 4.646 |
|  | *Microphthalmus aberrans* | 24 | 0.0002 |
|  | *Modiolus modiolus* | 2 | 0.0052 |
|  | *Monoculodes* sp. | 2 | 0.021 |
|  | Nemertea | 10 | 0.7168 |
|  | *Nephtys caeca* | 4 | 6.226 |
|  | *Nereis* sp. | 10 | 0.5096 |
|  | *Notomastus latericeus* | 18 | 0.222 |
|  | Oligochaeta | 34 | 0.0876 |
|  | *Ophelia limacina* | 26 | 0.1112 |
|  | *Ophelina acuminata* | 2 | 0.0512 |
|  | *Ophiura* sp. juvenile | 2 | 0.002 |
|  | *Orbinia swani* | 10 | 0.3848 |
|  | *Parougia eliasoni* | 6 | 0.0014 |
|  | *Periploma leanum* | 2 | 0.0038 |
|  | *Photis* sp. | 8 | 0.0034 |
|  | *Phyllodoce mucosa* | 16 | 0.1666 |
|  | Platyhelminthes | 2 | 0.0548 |
|  | *Polycirrus* sp. | 14 | 0.3044 |
|  | *Polydora concharum* | 6 | 0.1034 |
|  | *Polydora socialis* | 144 | 0.2432 |
|  | Polygordiidae | 2 | 0.0004 |
|  | *Protomedeia fasciata* | 2 | 0.0008 |
|  | *Pseudounicola obliquua* | 32 | 0.0264 |
|  | *Scoloplos armiger* | 26 | 0.6776 |
|  | *Serripes groenlandicus* | 12 | 0.0158 |
|  | *Solariella obscura* | 2 | 0.0868 |
|  | *Spio filicornis* | 74 | 0.264 |
|  | *Spiophanes bombyx* | 314 | 4.1942 |
|  | *Thuiaria* sp. | 2 | 0.4366 |
|  | *Unciola irrorata* | 44 | 0.1762 |
| vg58 | *Aeginina longicornis* | 2 | 0.0082 |
|  | *Aglaophamus circinata* | 66 | 8.9302 |
|  | *Ampharete acutifrons* | 4 | 0.0176 |
|  | *Ampharete finmarchica* | 40 | 0.2064 |
|  | *Aphrodita hastata* | 2 | 0.1224 |
|  | *Arcteobia anticostiensis* | 6 | 0.012 |
|  | *Arctica islandica* | 244 | 250.4234 |
|  | *Aricidea catherinae* | 132 | 0.3012 |
|  | Ascidiacea (solitary) | 40 | 0.1334 |
|  | *Bylgides* sp. | 2 | 0.0126 |
|  | *Capitella capitata* | 30 | 0.0538 |
|  | Capitellidae | 2 | 0.0022 |
|  | Cerianthidae | 10 | 20.8826 |
|  | *Chaetozone* sp. A | 2 | 0.0002 |
|  | *Chiridotea tuftsii* | 14 | 0.1284 |
|  | *Cistenides granulata* | 172 | 0.2316 |
|  | *Clymenella torquata* | 4 | 0.0224 |
|  | *Clymenura borealis* | 12 | 6.4694 |
|  | *Corophium crassicorne* | 20 | 0.0282 |
|  | *Crangon septemspinosa* | 8 | 0.1362 |
|  | *Cucumaria frondosa* | 30 | 0.0334 |
|  | *Cylichna alba* | 6 | 0.1196 |
|  | *Cyrtodaria siliqua* | 10 | 274.8038 |
|  | *Diastylis sculpta* | 8 | 0.074 |
|  | *Dyopedos* sp. | 10 | 0.0066 |
|  | *Echinarachnius parma* | 114 | 44.5424 |
|  | *Edotea montosa* | 12 | 0.0684 |
|  | *Edwardsia elegans* | 2 | 25.792 |
|  | *Eteone longa* | 68 | 0.503 |
|  | *Euchone incolor* | 2 | 0.0018 |
|  | *Euchone papillosa* | 26 | 0.104 |
|  | *Euclymene zonalis* | 68 | 3.4622 |
|  | *Eucratea loricata* | 2 | 0.0052 |
|  | *Eudorellopsis deformis* | 10 | 0.0152 |
|  | *Gattyana cirrhosa* | 4 | 0.0114 |
|  | *Goniada maculata* | 20 | 1.6548 |
|  | *Hydrozoa Athecata* | 2 | 0.0096 |
|  | *Leptocheirus pinguis* | 8 | 0.0972 |
|  | *Leptognathia* sp. | 4 | 0.0024 |
|  | *Lumbrineris fragilis* | 2 | 0.0012 |
|  | *Macoma calcarea* | 2 | 0.0084 |
|  | *Mactromeris polynyma* | 6 | 0.168 |
|  | *Mediomastus ambiseta* | 18 | 0.0392 |
|  | *Microphthalmus aberrans* | 32 | 0.0034 |
|  | *Monoculodes* sp. | 2 | 0.009 |
|  | Nemertea | 8 | 10.4298 |
|  | *Neosabellides* sp. | 26 | 0.0086 |
|  | Nephtyidae sp. juvenile | 16 | 0.091 |
|  | *Nephtys caeca* | 10 | 15.6314 |
|  | *Nereis* sp. | 8 | 0.2328 |
|  | *Ophelia limacina* | 12 | 0.2056 |
|  | *Ophiura* sp. juvenile | 6 | 0.0183 |
|  | *Orbinia swani* | 6 | 0.0938 |
|  | *Owenia fusiformis* | 4 | 0.0052 |
|  | *Parougia eliasoni* | 2 | 0.0002 |
|  | *Parvicardium pinnulatum* | 4 | 0.0022 |
|  | *Pentamera calcigera* | 12 | 38.7122 |
|  | *Periploma leanum* | 8 | 5.0962 |
|  | *Pherusa plumosa* | 58 | 24.7786 |
|  | *Pholoe minuta* | 14 | 0.078 |
|  | *Pholoe tecta* | 2 | 0.0002 |
|  | *Phoronis* sp. | 28 | 0.0212 |
|  | *Photis* sp. | 66 | 0.0478 |
|  | *Phyllodoce maculata* | 8 | 0.0004 |
|  | *Phyllodoce mucosa* | 50 | 0.5512 |
|  | *Polycirrus* sp. | 8 | 0.5894 |
|  | *Polydora concharum* | 160 | 3.7098 |
|  | *Polydora socialis* | 238 | 0.6314 |
|  | Polygordiidae | 6 | 0.0016 |
|  | *Prionospio steenstrupi* | 16 | 0.0344 |
|  | *Protomedeia fasciata* | 10 | 0.0078 |
|  | *Psammonyx* sp. | 2 | 0.0022 |
|  | Sabellidae | 40 | 0.0322 |
|  | *Scoloplos armiger* | 72 | 1.253 |
|  | *Serripes groenlandicus* | 140 | 0.1416 |
|  | *Sphaerodoropsis minuta* | 2 | 0.0002 |
|  | *Spio filicornis* | 52 | 0.22 |
|  | *Spiophanes bombyx* | 234 | 1.5804 |
|  | *Sthenelais limicola* | 4 | 1.1246 |
|  | *Tharyx* sp. | 18 | 0.1316 |
|  | *Thracia* sp. | 6 | 0.2812 |
|  | *Thuiaria* sp. | 2 | 0.0012 |
|  | Unciola irrorata | 4 | 0.0036 |
| vg59 | *Acanthohaustorius spinosus* | 10 | 0.1674 |
|  | Actiniaria (burrowing) | 20 | 1.77 |
|  | *Aeginina longicornis* | 4 | 0.0166 |
|  | *Aglaophamus circinata* | 56 | 2.8886 |
|  | *Ampharete finmarchica* | 178 | 0.7758 |
|  | *Anonyx sarsi* | 2 | 0.013 |
|  | *Aphrodita hastata* | 4 | 0.0084 |
|  | *Arcteobia anticostiensis* | 4 | 0.0024 |
|  | *Arctica islandica* | 30 | 237.245 |
|  | *Aricidea catherinae* | 2 | 0.0144 |
|  | *Aricidea wassi* | 20 | 0.1424 |
|  | Ascidiacea (solitary) | 80 | 7.0896 |
|  | *Cancer borealis* | 2 | 0.03 |
|  | *Capitella capitata* | 8 | 0.0208 |
|  | Capitellidae | 16 | 0.019 |
|  | *Chaetozone setosa* | 4 | 0.0136 |
|  | *Chaetozone* sp. A | 4 | 0.0074 |
|  | *Chone* sp. | 16 | 0.009 |
|  | *Cirolana polita* | 8 | 0.6562 |
|  | *Cistenides granulata* | 8 | 0.646 |
|  | *Clymenura borealis* | 140 | 14.6706 |
|  | *Corymorpha pendula* | 14 | 0.3736 |
|  | *Crangon septemspinosa* | 8 | 0.429 |
|  | *Cylichna alba* | 2 | 0.0062 |
|  | *Cyrtodaria siliqua* | 4 | 19.8296 |
|  | *Dyopedos* sp. | 36 | 0.0456 |
|  | *Echinarachnius parma* | 42 | 276.7172 |
|  | *Edotea montosa* | 2 | 0.0024 |
|  | *Eteone longa* | 56 | 0.539 |
|  | *Euchone papillosa* | 56 | 0.2664 |
|  | *Euclymene zonalis* | 6 | 0.168 |
|  | *Eudorellopsis deformis* | 6 | 0.0092 |
|  | *Euspira* sp. | 2 | 0.0558 |
|  | *Harmothoe imbricata* | 2 | 0.03 |
|  | *Hippomedon serratus* | 8 | 0.0812 |
|  | *Leptocheirus pinguis* | 10 | 0.5808 |
|  | *Levensinea gracilis* | 4 | 0.0018 |
|  | *Mactromeris polynyma* | 2 | 0.0144 |
|  | *Microphthalmus aberrans* | 20 | 0.0016 |
|  | *Monoculodes* sp. | 2 | 0.0156 |
|  | Nemertea | 2 | 0.019 |
|  | *Nephtys caeca* | 8 | 3.1122 |
|  | *Notomastus latericeus* | 6 | 0.125 |
|  | *Ophelia limacina* | 40 | 0.5152 |
|  | *Ophelina acuminata* | 2 | 0.0024 |
|  | *Ophiura sarsi* | 2 | 0.0134 |
|  | *Orbinia swani* | 14 | 0.2138 |
|  | *Pagurus* sp. | 2 | 0.0308 |
|  | *Pandora* sp. | 4 | 0.6024 |
|  | *Parougia eliasoni* | 6 | 0.0002 |
|  | *Parvicardium pinnulatum* | 2 | 0.4842 |
|  | *Periploma leanum* | 4 | 1.9032 |
|  | *Pherusa plumosa* | 4 | 0.0246 |
|  | *Photis* sp. | 4 | 0.0002 |
|  | *Phyllodoce mucosa* | 8 | 0.132 |
|  | *Pleustes panoplus* | 2 | 0.0094 |
|  | *Polycirrus* sp. | 12 | 0.2336 |
|  | *Polydora concharum* | 2 | 0.0258 |
|  | *Polydora socialis* | 106 | 0.2224 |
|  | *Protomedeia fasciata* | 2 | 0.0014 |
|  | *Scolelepis* sp. A | 2 | 0.17 |
|  | *Scoloplos armiger* | 24 | 0.115 |
|  | *Serripes groenlandicus* | 2 | 0.002 |
|  | *Siliqua squama* | 6 | 1.9652 |
|  | *Spio filicornis* | 112 | 0.2724 |
|  | *Spiophanes bombyx* | 318 | 4.072 |
|  | *Sthenelais limicola* | 2 | 0.5234 |
|  | *Unciola irrorata* | 68 | 0.4154 |
| wc_evg01 | *Ampharete finmarchica* | 42 | 0.145 |
|  | *Aricidea* sp. B | 26 | 0.0332 |
|  | Ascidiacea (solitary) | 6 | 0.677 |
|  | Campanulariidae | 2 | 0.0012 |
|  | *Cancer borealis* | 2 | 0.0088 |
|  | *Capitella capitata* | 20 | 0.0404 |
|  | Capitellidae | 208 | 0.1942 |
|  | *Chaetozone setosa* | 20 | 0.0594 |
|  | *Chaetozone* sp. A | 136 | 0.351 |
|  | *Cistenides granulata* | 22 | 0.0438 |
|  | *Clymenura borealis* | 92 | 4.968 |
|  | *Crangon septemspinosa* | 4 | 0.12 |
|  | *Cylichna alba* | 96 | 0.0566 |
|  | *Cyrtodaria siliqua* | 4 | 285.2 |
|  | *Diaphana minuta* | 8 | 0.0654 |
|  | *Echinarachnius parma* | 60 | 202.0032 |
|  | *Edotea montosa* | 4 | 0.0084 |
|  | *Edwardsia elegans* | 106 | 2.736 |
|  | *Ericthonius rubricornis* | 2 | 0.0004 |
|  | *Eteone longa* | 2 | 0.0054 |
|  | *Euchone papillosa* | 4 | 0.017 |
|  | *Euclymene zonalis* | 6 | 0.0256 |
|  | *Exogone* sp. | 108 | 0.0836 |
|  | *Glycera capitata* | 2 | 0.023 |
|  | *Harmothoe extenuata* | 2 | 0.0078 |
|  | *Hippomedon serratus* | 16 | 0.2832 |
|  | *Leptognathia* sp. | 12 | 0.0052 |
|  | *Levensinea gracilis* | 44 | 0.0354 |
|  | *Lumbrinerides acuta* | 92 | 1.5252 |
|  | *Metopella* sp. | 2 | 0.0004 |
|  | *Musculus* sp. | 4 | 0.0038 |
|  | Mytiloidea | 2 | 0.0002 |
|  | Nemertea | 16 | 2.985 |
|  | Nephtyidae sp. juvenile | 4 | 0.0118 |
|  | *Nephtys bucera* | 2 | 1.8432 |
|  | *Nephtys caeca* | 2 | 0.518 |
|  | *Notomastus latericeus* | 88 | 0.2132 |
|  | Oligochaeta | 36 | 0.1112 |
|  | *Ophelia limacina* | 2 | 0.0038 |
|  | *Ophiura* sp. juvenile | 2 | 0.001 |
|  | *Orbinia swani* | 2 | 0.4622 |
|  | *Pagurus* sp. | 4 | 0.0106 |
|  | *Paraonis* sp. | 44 | 0.0636 |
|  | *Parougia eliasoni* | 8 | 0.0036 |
|  | *Parvicardium pinnulatum* | 6 | 0.1116 |
|  | *Periploma leanum* | 6 | 4.3654 |
|  | *Pherusa plumosa* | 2 | 0.0006 |
|  | *Pholoe tecta* | 4 | 0.0004 |
|  | *Photis* sp. | 18 | 0.0062 |
|  | *Phoxocephalus holbolli* | 4 | 0.0146 |
|  | *Phyllodoce mucosa* | 22 | 0.0476 |
|  | Platyhelminthes | 10 | 0.0868 |
|  | *Pleusymtes glaber* | 4 | 0.0028 |
|  | Polygordiidae | 88 | 0.0376 |
|  | *Protodorvillea kefersteini* | 2 | 0.0002 |
|  | *Protomedeia fasciata* | 22 | 0.0138 |
|  | Sabellidae | 4 | 0.0008 |
|  | *Scolelepis squamata* | 2 | 0.0124 |
|  | *Solariella obscura* | 2 | 0.216 |
|  | *Spio filicornis* | 10 | 0.0204 |
|  | *Spiophanes bombyx* | 276 | 0.3492 |
|  | Syllidae | 14 | 0.0026 |
|  | *Unciola irrorata* | 72 | 0.2466 |
| wc_evg02 | Actiniaria (burrowing) | 80 | 20.137 |
|  | *Anonyx sarsi* | 2 | 0.0214 |
|  | *Arctica islandica* | 4 | 0.0032 |
|  | *Argissa hamatipes* | 2 | 0.0044 |
|  | *Aricidea catherinae* | 4 | 0.006 |
|  | Ascidiacea (solitary) | 16 | 0.032 |
|  | Asteroidea | 2 | 0.001 |
|  | *Autolytus* sp. | 2 | 0.0012 |
|  | *Caprella* sp. | 4 | 0.0182 |
|  | *Chaetozone* sp. A | 8 | 0.0324 |
|  | *Chone* sp. | 8 | 0.0258 |
|  | *Cistenides granulata* | 2 | 0.0006 |
|  | *Clymenura borealis* | 2 | 0.0212 |
|  | *Corophium crassicorne* | 16 | 0.0746 |
|  | Crustacea | 2 | 0.0002 |
|  | *Cucumaria frondosa* | 8 | 0.0148 |
|  | *Cyrtodaria siliqua* | 2 | 0.0068 |
|  | *Drilonereis magna* | 4 | 0.0206 |
|  | *Echinarachnius parma* | 10 | 0.02 |
|  | *Edotea montosa* | 2 | 0.0032 |
|  | *Eteone longa* | 2 | 0.0024 |
|  | *Eteone trilineata* | 2 | 0.0024 |
|  | *Euchone papillosa* | 2 | 0.0126 |
|  | *Euclymene zonalis* | 646 | 8.674 |
|  | *Exogone* sp. | 4 | 0.0014 |
|  | Gastropoda | 2 | 0.001 |
|  | *Glycera capitata* | 88 | 6.318 |
|  | *Harmothoe extenuata* | 18 | 0.0334 |
|  | *Hiatella arctica* | 2 | 0.0002 |
|  | Invert. undetermined | 22 | 0.026 |
|  | *Laphania boecki* | 4 | 0.044 |
|  | *Lumbrinerides acuta* | 16 | 0.2472 |
|  | *Monoculodes* sp. | 2 | 0.0098 |
|  | *Musculus* sp. | 2 | 0.002 |
|  | Mytiloidea | 36 | 0.0436 |
|  | Nemertea | 88 | 0.205 |
|  | *Nephtys caeca* | 2 | 5.704 |
|  | *Nereis* sp. | 2 | 0.0248 |
|  | Oligochaeta | 242 | 1.4052 |
|  | *Ophiopholis aculeata* | 2 | 0.0096 |
|  | *Ophiura robusta* | 2 | 0.0244 |
|  | *Ophiura* sp. juvenile | 4 | 0.001 |
|  | *Paraonis* sp. | 28 | 0.0718 |
|  | *Parougia eliasoni* | 6 | 0.0024 |
|  | *Parvicardium pinnulatum* | 18 | 0.1152 |
|  | *Petaloproctus tenuis* | 2 | 0.0068 |
|  | *Pholoe tecta* | 6 | 0.0026 |
|  | *Photis* sp. | 2 | 0.001 |
|  | Platyhelminthes | 2 | 0.0052 |
|  | *Pleusymtes glaber* | 10 | 0.0064 |
|  | *Polydora caulleryi* | 10 | 0.0204 |
|  | *Polydora socialis* | 2 | 0.0016 |
|  | Polygordiidae | 984 | 3.9214 |
|  | *Protodorvillea gaspeensis* | 2 | 0.0004 |
|  | *Protomedeia fasciata* | 52 | 0.0276 |
|  | *Psolus* sp. | 8 | 0.0114 |
|  | Questidae | 2 | 0.001 |
|  | *Sertularia polyzonias* | 2 | 0.0204 |
|  | *Spio filicornis* | 14 | 0.0302 |
|  | *Spiophanes bombyx* | 2 | 0.0002 |
|  | *Strongylocentrotus* sp. | 4 | 0.0016 |
|  | Syllidae | 14 | 0.004 |
|  | *Thracia* sp. | 4 | 0.0082 |
|  | *Unciola irrorata* | 64 | 0.1698 |
| wc_vg01 | *Aeginina longicornis* | 6 | 0.0264 |
|  | Ascidiacea (solitary) | 6 | 0.1376 |
|  | Asteroidea | 10 | 0.8376 |
|  | *Boreotrophon* sp. | 2 | 0.0376 |
|  | Bryozoa Ascophora | 2 | 0.0742 |
|  | Campanulariidae | 2 | 0.1976 |
|  | *Caprella* sp. | 122 | 0.0666 |
|  | *Cauloramphus* sp. | 2 | 0.0148 |
|  | *Chone* sp. | 6 | 0.3158 |
|  | *Corophium crassicorne* | 8 | 0.004 |
|  | *Cucumaria frondosa* | 2 | 0.001 |
|  | *Dendrobeania* sp. | 2 | 0.0686 |
|  | Didemnidae | 2 | 0.0886 |
|  | *Dyopedos* sp. | 6 | 0.0012 |
|  | *Edwardsia elegans* | 2 | 0.1428 |
|  | *Ericthonius rubricornis* | 48 | 0.0512 |
|  | *Euclymene zonalis* | 34 | 0.0186 |
|  | *Eucratea loricata* | 2 | 0.01 |
|  | *Eudendrium* sp. | 2 | 0.1044 |
|  | *Eulalia bilineata* | 4 | 0.008 |
|  | *Eulalia viridis* | 12 | 0.0578 |
|  | *Exogone* sp. | 22 | 0.0028 |
|  | *Gattyana cirrhosa* | 2 | 0.0092 |
|  | *Glycera capitata* | 4 | 0.0002 |
|  | *Hiatella arctica* | 14 | 0.0096 |
|  | *Ischyrocerus* sp. | 48 | 0.009 |
|  | *Lafoea* sp. | 2 | 0.007 |
|  | *Leucosolenia* sp. | 2 | 0.0984 |
|  | *Modiolus modiolus* | 2 | 0.0002 |
|  | Mytiloidea | 16 | 0.2002 |
|  | *Myxicola infundibulum* | 2 | 0.0106 |
|  | Nemertea | 42 | 0.1382 |
|  | *Nereis* sp. | 12 | 0.0034 |
|  | *Nicomache lumbricalis* | 84 | 0.8542 |
|  | *Ophiura robusta* | 4 | 0.0056 |
|  | *Ophiura* sp. juvenile | 6 | 0.0004 |
|  | *Parvicardium pinnulatum* | 6 | 0.0036 |
|  | *Petaloproctus tenuis* | 116 | 0.4052 |
|  | *Pholoe tecta* | 4 | 0.0002 |
|  | *Photis* sp. | 42 | 0.0202 |
|  | *Phyllodoce maculata* | 174 | 0.0668 |
|  | *Pleusymtes glaber* | 2 | 0.0032 |
|  | *Polycirrus* sp. | 26 | 0.1402 |
|  | *Polydora socialis* | 16 | 0.0352 |
|  | Polygordiidae | 2 | 0.0012 |
|  | *Protodorvillea kefersteini* | 2 | 0.0004 |
|  | *Protomedeia fasciata* | 12 | 0.0056 |
|  | *Pseudopotamilla* sp. | 40 | 1.6372 |
|  | *Psolus* sp. | 6 | 0.0392 |
|  | *Puncturella noachina* | 2 | 0.02 |
|  | *Scrupocellaria scabra* | 2 | 0.01 |
|  | *Sertularia mirabilis* | 2 | 0.577 |
|  | *Sertularia* sp. | 2 | 0.0564 |
|  | *Spiophanes bombyx* | 2 | 0.0022 |
|  | *Stenosemus albus* | 2 | 0.0788 |
|  | Stenothoidae | 4 | 0.0002 |
|  | *Strongylocentrotus* sp. | 22 | 0.0462 |
|  | *Sycettida* sp. | 2 | 0.018 |
|  | Syllidae | 26 | 0.015 |
|  | *Tharyx* sp. | 2 | 0.0096 |
|  | *Thelepus cincinnatus* | 30 | 1.5776 |
|  | *Unciola irrorata* | 120 | 0.5454 |
| wc_vg02 | *Acarina* sp. | 2 | 0.0002 |
|  | Actiniaria (burrowing) | 2 | 0.156 |
|  | *Ampharete finmarchica* | 6 | 0.0166 |
|  | Amphipoda | 2 | 0.0006 |
|  | *Anomia* sp. | 8 | 0.0266 |
|  | *Aricidea* sp. B | 2 | 0.002 |
|  | Ascidiacea (colonial) | 2 | 1.5546 |
|  | Ascidiacea (solitary) | 2 | 0.0064 |
|  | Asteroidea | 12 | 2.9726 |
|  | *Boreotrophon* sp. | 4 | 0.073 |
|  | Bryozoa Ascophora | 2 | 0.0836 |
|  | Bryozoa Cyclostomata | 2 | 0.0152 |
|  | *Capitella capitata* | 12 | 0.0118 |
|  | *Caprella* sp. | 50 | 0.0718 |
|  | *Chone* sp. | 18 | 2.334 |
|  | *Cirratulus cirratus* | 18 | 0.035 |
|  | *Cistenides granulata* | 18 | 0.1934 |
|  | *Clymenura borealis* | 6 | 0.0886 |
|  | *Corophium crassicorne* | 2 | 0.001 |
|  | *Cyclocardia novangliae* | 16 | 0.1856 |
|  | *Cyclocardia* sp. A | 44 | 0.0312 |
|  | *Dendrobeania* sp. | 2 | 0.2094 |
|  | Didemnidae | 2 | 46.465 |
|  | *Dodecaceria fimbriata* | 6 | 0.0052 |
|  | *Dyopedos* sp. | 2 | 0.0006 |
|  | *Echinarachnius parma* | 18 | 0.01 |
|  | *Ericthonius rubricornis* | 62 | 0.0924 |
|  | *Euclymene zonalis* | 28 | 0.31 |
|  | *Eucratea loricata* | 2 | 0.0016 |
|  | *Eudendrium* sp. | 2 | 0.0146 |
|  | *Eulalia viridis* | 42 | 0.0828 |
|  | *Eunoe oerstedi* | 2 | 0.2172 |
|  | *Exogone* sp. | 38 | 0.0112 |
|  | Gastropoda | 2 | 0.0042 |
|  | *Glycera capitata* | 14 | 0.1462 |
|  | *Goniada maculata* | 2 | 0.0606 |
|  | *Henricia* sp. | 2 | 0.059 |
|  | *Hiatella arctica* | 78 | 0.5586 |
|  | *Hyas coarctatus* | 2 | 0.0136 |
|  | *Hydrozoa Athecata* | 2 | 0.0146 |
|  | *Janira alta* | 8 | 0.0174 |
|  | *Lafoea* sp. | 2 | 0.0398 |
|  | *Margarites striatus* | 8 | 0.3084 |
|  | *Melita dentata* | 4 | 0.0556 |
|  | *Modiolus modiolus* | 80 | 1707.7006 |
|  | *Moelleria costulata* | 2 | 0.0006 |
|  | *Monoculodes* sp. | 2 | 0.0008 |
|  | *Musculus* sp. | 18 | 0.0214 |
|  | Mytiloidea | 28 | 0.028 |
|  | Nemertea | 54 | 0.4172 |
|  | *Neosabellides* sp. | 4 | 0.0206 |
|  | *Nephtys discors* | 2 | 1.7176 |
|  | *Nereis* sp. | 6 | 0.0198 |
|  | *Nicomache lumbricalis* | 38 | 0.9372 |
|  | *Ophiopholis aculeata* | 180 | 4.714 |
|  | *Ophiura robusta* | 64 | 0.5386 |
|  | *Ophiura* sp. juvenile | 102 | 0.0208 |
|  | *Owenia fusiformis* | 2 | 0.0026 |
|  | *Parvicardium pinnulatum* | 8 | 0.002 |
|  | *Petaloproctus tenuis* | 82 | 0.3436 |
|  | *Pherusa plumosa* | 2 | 0.0028 |
|  | *Pholoe tecta* | 4 | 0.001 |
|  | *Photis* sp. | 42 | 0.0252 |
|  | *Phoxocephalus holbolli* | 8 | 0.0036 |
|  | *Phyllodoce maculata* | 94 | 0.1936 |
|  | *Pleusymtes glaber* | 18 | 0.002 |
|  | *Polycirrus* sp. | 140 | 1.4318 |
|  | *Polydora socialis* | 10 | 0.0188 |
|  | Polynoidae sp. juvenile | 8 | 0.0016 |
|  | Porifera | 2 | 0.0552 |
|  | *Praxillella praetermissa* | 10 | 0.1166 |
|  | *Protomedeia fasciata* | 16 | 0.0068 |
|  | *Pseudopotamilla* sp. | 14 | 0.0942 |
|  | *Psolus* sp. | 6 | 0.0052 |
|  | *Puncturella noachina* | 30 | 0.187 |
|  | *Rhizocaulus verticillatus* | 2 | 0.0144 |
|  | Sabellidae | 10 | 0.0092 |
|  | *Sertularia polyzonias* | 2 | 0.012 |
|  | *Sertularia* sp. | 2 | 0.0038 |
|  | *Stenosemus albus* | 10 | 0.2544 |
|  | Stenothoidae | 6 | 0.0002 |
|  | *Strongylocentrotus* sp. | 34 | 0.0424 |
|  | Syllidae | 70 | 0.065 |
|  | *Terebratulina septentrionalis* | 2 | 0.0016 |
|  | *Thelepus cincinnatus* | 176 | 23.8914 |
|  | *Thracia* sp. | 14 | 0.0024 |
|  | *Tiron spiniferus* | 2 | 0.0014 |
|  | *Unciola irrorata* | 122 | 0.573 |
|  | *Velutina* sp. | 14 | 0.0166 |
| wc_vg03 | Actiniaria (burrowing) | 6 | 0.2032 |
|  | Actiniaria (non burrowing) | 2 | 0.0646 |
|  | *Aglaophamus circinata* | 2 | 0.0934 |
|  | *Ampharete finmarchica* | 58 | 0.1672 |
|  | *Anomia* sp. | 8 | 0.0502 |
|  | *Anonyx sarsi* | 28 | 0.354 |
|  | *Arctica islandica* | 2 | 0.0282 |
|  | *Argissa hamatipes* | 2 | 0.002 |
|  | *Aricidea catherinae* | 4 | 0.0018 |
|  | Ascidiacea (solitary) | 14 | 0.6864 |
|  | Asteroidea | 2 | 1.2306 |
|  | *Boreocingula* sp. | 4 | 0.002 |
|  | Campanulariidae | 2 | 0.0078 |
|  | *Chaetozone* sp. A | 8 | 0.0188 |
|  | *Chone* sp. | 50 | 0.1156 |
|  | *Cistenides granulata* | 234 | 0.1402 |
|  | *Clymenura borealis* | 14 | 1.2736 |
|  | *Crangon septemspinosa* | 24 | 0.3286 |
|  | *Crystallophrisson nitidulum* | 4 | 0.0008 |
|  | *Cucumaria frondosa* | 24 | 0.0386 |
|  | *Cylichna alba* | 2 | 0.0014 |
|  | *Cyrtodaria siliqua* | 64 | 0.1026 |
|  | *Drilonereis magna* | 6 | 0.0304 |
|  | *Echinarachnius parma* | 16 | 0.0024 |
|  | *Edwardsia elegans* | 42 | 0.7426 |
|  | *Ericthonius rubricornis* | 2 | 0.0066 |
|  | *Euchone papillosa* | 102 | 0.7472 |
|  | *Euclymene zonalis* | 2094 | 24.9932 |
|  | *Eulalia bilineata* | 2 | 0.0088 |
|  | *Euspira* sp. | 10 | 0.0588 |
|  | *Exogone* sp. | 52 | 0.0194 |
|  | Gastropoda | 6 | 0.011 |
|  | *Glycera capitata* | 516 | 16.844 |
|  | *Goniadella gracilis* | 2 | 0.0198 |
|  | *Harmothoe extenuata* | 62 | 0.1514 |
|  | *Harmothoe imbricata* | 2 | 0.0588 |
|  | *Laphania boecki* | 28 | 1.3488 |
|  | *Lumbrinerides acuta* | 54 | 1.0346 |
|  | *Margarites striatus* | 8 | 0.0324 |
|  | *Mediomastus ambiseta* | 2 | 0.0002 |
|  | *Modiolus modiolus* | 8 | 0.0018 |
|  | *Monoculodes* sp. | 14 | 0.1188 |
|  | *Monoculodes tuberculatus* | 2 | 0.0006 |
|  | *Munna fabricii* | 2 | 0.0002 |
|  | *Musculus* sp. | 6 | 0.0174 |
|  | Mytiloidea | 118 | 5.041 |
|  | Nemertea | 66 | 0.3752 |
|  | *Neosabellides* sp. | 2 | 0.0008 |
|  | Oligochaeta | 22 | 0.0086 |
|  | *Ophiura robusta* | 74 | 0.1016 |
|  | *Ophiura* sp. juvenile | 92 | 0.0484 |
|  | *Pagurus* sp. | 12 | 0.0672 |
|  | *Paraonis* sp. | 10 | 0.0228 |
|  | *Parougia eliasoni* | 4 | 0.0028 |
|  | *Parvicardium pinnulatum* | 62 | 0.3306 |
|  | *Pherusa plumosa* | 4 | 0.0008 |
|  | *Pholoe tecta* | 36 | 0.023 |
|  | *Phyllodoce maculata* | 8 | 0.001 |
|  | *Placopecten magellanicus* | 2 | 0.0002 |
|  | Platyhelminthes | 8 | 0.0504 |
|  | *Pleustes panoplus* | 2 | 0.0256 |
|  | *Pleusymtes glaber* | 8 | 0.0056 |
|  | *Polycirrus* sp. | 4 | 0.1308 |
|  | *Polydora caulleryi* | 276 | 0.7916 |
|  | Polygordiidae | 1070 | 3.3864 |
|  | *Proclea graffi* | 2 | 0.0006 |
|  | *Protomedeia fasciata* | 602 | 0.5194 |
|  | *Psolus* sp. | 30 | 0.0598 |
|  | Questidae | 6 | 0.001 |
|  | *Sclerocrangon boreas* | 2 | 13.807 |
|  | *Sertularia polyzonias* | 2 | 0.0148 |
|  | *Spio filicornis* | 10 | 0.0306 |
|  | *Spiophanes bombyx* | 30 | 0.071 |
|  | *Stenosemus albus* | 2 | 0.0002 |
|  | *Strongylocentrotus* sp. | 274 | 0.1822 |
|  | Syllidae | 10 | 0.0014 |
|  | *Tachyrhynchus erosus* | 2 | 0.1074 |
|  | *Tharyx* sp. | 2 | 0.001 |
|  | *Thracia* sp. | 58 | 0.0394 |
|  | *Unciola irrorata* | 644 | 4.3942 |
| wc_vg04 | Actiniaria (non burrowing) | 2 | 0.0016 |
|  | Alcyonacea | 2 | 0.4804 |
|  | Asteroidea | 4 | 1.561 |
|  | *Caprella* sp. | 72 | 0.1374 |
|  | *Chone* sp. | 2 | 1.6468 |
|  | *Crossaster papposus* | 2 | 0.0788 |
|  | *Edwardsia elegans* | 2 | 0.0616 |
|  | *Ericthonius rubricornis* | 22 | 0.0746 |
|  | *Euchone papillosa* | 2 | 0.008 |
|  | *Euclymene zonalis* | 16 | 0.103 |
|  | *Eulalia viridis* | 2 | 0.0096 |
|  | *Hiatella arctica* | 2 | 0.0002 |
|  | *Modiolus modiolus* | 8 | 184.7012 |
|  | *Moelleria costulata* | 2 | 0.0024 |
|  | Mytiloidea | 6 | 0.2512 |
|  | *Nicomache lumbricalis* | 2 | 0.9752 |
|  | Oligochaeta | 2 | 0.0006 |
|  | *Ophiopholis aculeata* | 4 | 0.0038 |
|  | *Ophiura robusta* | 2 | 0.0468 |
|  | *Ophiura* sp. juvenile | 4 | 0.0018 |
|  | *Owenia fusiformis* | 2 | 0.0172 |
|  | *Paraonis* sp. | 2 | 0.0004 |
|  | *Photis* sp. | 8 | 0.0084 |
|  | *Phyllodoce maculata* | 10 | 0.0152 |
|  | *Placopecten magellanicus* | 2 | 439 |
|  | *Pleusymtes glaber* | 2 | 0.0002 |
|  | *Polydora socialis* | 2 | 0.0004 |
|  | *Pseudopotamilla* sp. | 2 | 0.3612 |
|  | *Psolus* sp. | 2 | 0.0024 |
|  | *Sertularia polyzonias* | 2 | 0.0372 |
|  | *Sertularia* sp. | 2 | 0.004 |
|  | *Sertularia tricuspidatus* | 2 | 0.04 |
|  | *Stenosemus albus* | 6 | 0.0822 |
|  | *Strongylocentrotus* sp. | 12 | 0.259 |
|  | *Tharyx* sp. | 2 | 0.0002 |
|  | *Thelepus cincinnatus* | 10 | 0.709 |
|  | *Tonicella rubra* | 2 | 0.4038 |
|  | *Unciola irrorata* | 8 | 0.0264 |
| wc_vg05 | Actiniaria (burrowing) | 10 | 0.0294 |
|  | *Ampharete finmarchica* | 6 | 0.026 |
|  | *Apherusa* sp. | 74 | 0.0272 |
|  | *Aricidea catherinae* | 26 | 0.0426 |
|  | *Aricidea* sp. B | 4 | 0.0006 |
|  | *Asabellides* sp. | 2 | 0.001 |
|  | Ascidiacea (solitary) | 14 | 0.0418 |
|  | *Astarte* sp. | 4 | 0.0006 |
|  | Asteroidea | 2 | 0.0002 |
|  | *Brada granosa* | 2 | 0.7452 |
|  | *Cancer borealis* | 2 | 0.0046 |
|  | Capitellidae | 8 | 0.0058 |
|  | Cerianthidae | 4 | 9.423 |
|  | *Chaetozone* sp. A | 22 | 0.027 |
|  | *Chone* sp. | 34 | 0.0442 |
|  | Cirratulidae | 2 | 0.0018 |
|  | *Cistenides granulata* | 276 | 0.3194 |
|  | *Corophium crassicorne* | 2 | 0.0006 |
|  | *Crangon septemspinosa* | 4 | 0.0256 |
|  | *Cucumaria frondosa* | 56 | 0.0618 |
|  | *Cyclocardia* sp. A | 6 | 0.0008 |
|  | *Cylichna alba* | 30 | 0.0156 |
|  | *Cyrtodaria siliqua* | 14 | 0.2568 |
|  | *Diastylis sculpta* | 2 | 0.0018 |
|  | *Drilonereis magna* | 6 | 0.1438 |
|  | *Echinarachnius parma* | 14 | 0.14 |
|  | *Edwardsia elegans* | 14 | 1.9988 |
|  | *Eteone longa* | 18 | 0.044 |
|  | *Eualus pusiolus* | 12 | 0.1116 |
|  | *Euchone papillosa* | 2 | 0.005 |
|  | *Euclymene zonalis* | 318 | 3.2358 |
|  | *Eumida* sp. | 4 | 0.0146 |
|  | *Exogone* sp. | 238 | 0.065 |
|  | Gastropoda | 724 | 0.2086 |
|  | *Gattyana cirrhosa* | 2 | 0.0128 |
|  | *Glycera capitata* | 154 | 3.7496 |
|  | *Guernea nordenskioldi* | 6 | 0.0038 |
|  | *Harmothoe extenuata* | 156 | 0.2466 |
|  | *Harmothoe imbricata* | 8 | 0.2992 |
|  | *Hiatella arctica* | 28 | 0.0084 |
|  | *Laonice cirrata* | 2 | 0.1056 |
|  | *Laphania boecki* | 8 | 0.0426 |
|  | *Lumbrinerides acuta* | 2 | 0.0232 |
|  | *Maera danae* | 20 | 0.289 |
|  | *Moelleria costulata* | 70 | 0.0434 |
|  | *Monoculodes* sp. | 2 | 0.0006 |
|  | *Musculus* sp. | 48 | 0.0198 |
|  | Mytiloidea | 250 | 0.1294 |
|  | Nemertea | 204 | 0.2216 |
|  | *Neosabellides* sp. | 2 | 0.0016 |
|  | Nephtyidae sp. juvenile | 6 | 0.004 |
|  | *Nereis* sp. | 4 | 0.0004 |
|  | Oligochaeta | 136 | 0.0468 |
|  | *Ophelia limacina* | 8 | 0.0012 |
|  | *Ophiura* sp. juvenile | 252 | 0.1118 |
|  | *Paraonis* sp. | 24 | 0.0188 |
|  | *Parougia eliasoni* | 4 | 0.0022 |
|  | *Parvicardium pinnulatum* | 54 | 0.0062 |
|  | *Phascolion strombus* | 2 | 0.005 |
|  | *Pherusa plumosa* | 8 | 0.001 |
|  | *Philine* sp. | 2 | 0.0002 |
|  | *Pholoe tecta* | 156 | 0.0882 |
|  | *Phyllodoce groenlandica* | 8 | 0.0466 |
|  | *Phyllodoce maculata* | 80 | 0.097 |
|  | *Phyllodoce mucosa* | 56 | 0.0424 |
|  | Phyllodocidae sp. juvenile | 16 | 0.0132 |
|  | *Placopecten magellanicus* | 4 | 0.003 |
|  | Platyhelminthes | 2 | 0.0114 |
|  | *Pleurogonium spinosissimus* | 4 | 0.0008 |
|  | *Polycirrus* sp. | 16 | 0.2316 |
|  | *Polydora socialis* | 3720 | 1.497 |
|  | Polygordiidae | 132 | 0.1398 |
|  | *Prionospio steenstrupi* | 26 | 0.0076 |
|  | *Protodorvillea gaspeensis* | 26 | 0.0052 |
|  | *Protomedeia fasciata* | 214 | 0.1178 |
|  | *Psolus* sp. | 54 | 0.0528 |
|  | *Scolelepis squamata* | 2 | 0.003 |
|  | *Scoloplos armiger* | 8 | 0.1652 |
|  | *Spio filicornis* | 26 | 0.06 |
|  | *Spiophanes bombyx* | 12 | 0.0044 |
|  | *Stenosemus albus* | 4 | 0.1192 |
|  | *Strongylocentrotus* sp. | 54 | 0.0166 |
|  | Syllidae | 86 | 0.0104 |
|  | *Syrrhoe crenulata* | 2 | 0.006 |
|  | *Tharyx* sp. | 80 | 0.2238 |
|  | *Thracia* sp. | 44 | 0.029 |
|  | *Tiron spiniferus* | 6 | 0.0116 |
|  | *Travisia* sp. | 12 | 1.1192 |
|  | *Unciola irrorata* | 322 | 1.6108 |
| wc_vg06 | Actiniaria (burrowing) | 102 | 14.4316 |
|  | *Aglaophamus circinata* | 4 | 0.416 |
|  | *Ampharete finmarchica* | 12 | 0.0534 |
|  | *Anonyx sarsi* | 4 | 0.0458 |
|  | *Arcteobia anticostiensis* | 2 | 0.0084 |
|  | *Aricidea wassi* | 2 | 0.0014 |
|  | Ascidiacea (solitary) | 20 | 0.6948 |
|  | Asteroidea | 2 | 0.0064 |
|  | *Chaetozone* sp. A | 16 | 0.035 |
|  | *Chone* sp. | 14 | 0.0186 |
|  | *Cistenides granulata* | 26 | 0.021 |
|  | *Crangon septemspinosa* | 6 | 0.0558 |
|  | *Cucumaria frondosa* | 34 | 0.032 |
|  | *Cyclocardia novangliae* | 2 | 0.007 |
|  | *Cylichna alba* | 4 | 0.0048 |
|  | *Cyrtodaria siliqua* | 18 | 345.678 |
|  | *Dulichia* sp. | 2 | 0.0002 |
|  | *Echinarachnius parma* | 60 | 49.4028 |
|  | *Edotea montosa* | 2 | 0.0022 |
|  | *Ericthonius rubricornis* | 2 | 0.0068 |
|  | *Euchone papillosa* | 20 | 0.0982 |
|  | *Euclymene zonalis* | 162 | 7.2596 |
|  | *Euspira* sp. | 2 | 0.0128 |
|  | *Exogone* sp. | 16 | 0.0056 |
|  | *Glycera capitata* | 84 | 3.64 |
|  | *Harmothoe extenuata* | 12 | 0.0496 |
|  | Invert. undetermined | 2 | 0.0004 |
|  | *Ischyrocerus* sp. | 4 | 0.0144 |
|  | *Laphania boecki* | 2 | 0.0152 |
|  | *Lumbrinerides acuta* | 46 | 0.7818 |
|  | *Musculus* sp. | 6 | 0.0048 |
|  | Mytiloidea | 22 | 1.6362 |
|  | *Nainereis quadricuspida* | 2 | 0.0004 |
|  | Nemertea | 66 | 0.1116 |
|  | *Nephtys caeca* | 2 | 3.6122 |
|  | Oligochaeta | 122 | 0.3326 |
|  | *Ophelia limacina* | 2 | 0.0774 |
|  | *Ophiura* sp. juvenile | 40 | 0.04 |
|  | *Pagurus* sp. | 2 | 0.0052 |
|  | *Paraonis* sp. | 40 | 0.0528 |
|  | *Parougia eliasoni* | 2 | 0.0034 |
|  | *Parvicardium pinnulatum* | 28 | 0.0346 |
|  | *Pholoe tecta* | 6 | 0.0016 |
|  | *Phyllodoce maculata* | 2 | 0.0006 |
|  | Platyhelminthes | 4 | 0.0234 |
|  | *Polycirrus* sp. | 2 | 0.0036 |
|  | Polygordiidae | 1038 | 3.6186 |
|  | *Protodorvillea gaspeensis* | 2 | 0.001 |
|  | *Protomedeia fasciata* | 154 | 0.082 |
|  | *Psolus* sp. | 32 | 0.0502 |
|  | Questidae | 2 | 0.0002 |
|  | *Spio filicornis* | 16 | 0.0262 |
|  | *Spiophanes bombyx* | 2 | 0.0008 |
|  | *Strongylocentrotus* sp. | 12 | 0.0078 |
|  | Syllidae | 62 | 0.0114 |
|  | *Syrrhoe crenulata* | 2 | 0.0006 |
|  | *Thracia* sp. | 10 | 0.008 |
|  | *Unciola irrorata* | 84 | 0.2954 |
|  | *Velutina* sp. | 2 | 0.0134 |
| wc_vg07 | *Aglaophamus circinata* | 4 | 0.932 |
|  | *Ampharete finmarchica* | 64 | 0.2126 |
|  | *Argissa hamatipes* | 2 | 0.0002 |
|  | *Aricidea* sp. B | 10 | 0.012 |
|  | *Aricidea wassi* | 2 | 0.012 |
|  | Ascidiacea (solitary) | 2 | 0.2248 |
|  | Bivalvia | 2 | 0.9604 |
|  | Capitellidae | 6 | 0.0012 |
|  | *Chaetozone setosa* | 4 | 0.0134 |
|  | *Chaetozone* sp. A | 24 | 0.0572 |
|  | *Clymenura borealis* | 44 | 3.0384 |
|  | *Crangon septemspinosa* | 4 | 0.115 |
|  | *Cyrtodaria siliqua* | 4 | 203.3818 |
|  | *Diaphana minuta* | 4 | 0.0038 |
|  | *Echinarachnius parma* | 38 | 160.931 |
|  | *Edotea montosa* | 4 | 0.0096 |
|  | *Edwardsia elegans* | 66 | 2.2544 |
|  | *Euchone papillosa* | 8 | 0.0186 |
|  | *Euclymene zonalis* | 6 | 0.059 |
|  | *Exogone* sp. | 24 | 0.0084 |
|  | *Hippomedon serratus* | 18 | 0.2576 |
|  | *Leptognathia* sp. | 8 | 0.0046 |
|  | *Levensinea gracilis* | 16 | 0.0144 |
|  | *Lumbrinerides acuta* | 78 | 0.768 |
|  | *Mactromeris polynyma* | 4 | 178.3 |
|  | *Musculus* sp. | 2 | 0.0034 |
|  | Nemertea | 4 | 0.0022 |
|  | Nephtyidae sp. juvenile | 6 | 0.0102 |
|  | *Nephtys caeca* | 6 | 2.4772 |
|  | *Notomastus latericeus* | 10 | 0.0534 |
|  | Oligochaeta | 22 | 0.0728 |
|  | *Ophelia limacina* | 6 | 2.695 |
|  | *Orchomenella minuta* | 2 | 0.0024 |
|  | *Paraonis* sp. | 8 | 0.0044 |
|  | *Parougia eliasoni* | 2 | 0.0002 |
|  | *Parvicardium pinnulatum* | 6 | 0.0518 |
|  | *Periploma leanum* | 10 | 2.8924 |
|  | *Photis* sp. | 4 | 0.0014 |
|  | *Phoxocephalus holbolli* | 8 | 0.011 |
|  | *Phyllodoce mucosa* | 2 | 0.0168 |
|  | *Platyhelminthes* | 12 | 0.0888 |
|  | *Polydora caulleryi* | 2 | 0.001 |
|  | Polygordiidae | 6 | 0.0054 |
|  | *Protodorvillea kefersteini* | 2 | 0.0002 |
|  | *Protomedeia fasciata* | 2 | 0.0002 |
|  | *Psammonyx* sp. | 2 | 0.0062 |
|  | Questidae | 2 | 0.0032 |
|  | *Scolelepis squamata* | 2 | 0.0092 |
|  | *Serripes groenlandicus* | 2 | 0.0092 |
|  | *Spio filicornis* | 8 | 0.0352 |
|  | *Spiophanes bombyx* | 100 | 0.1938 |
|  | Syllidae | 8 | 0.0012 |
|  | *Thracia* sp. | 4 | 0.2604 |
|  | *Unciola irrorata* | 30 | 0.0926 |
| wc_vg08 | Actiniaria (burrowing) | 2 | 0.052 |
|  | *Aglaophamus circinata* | 2 | 0.0466 |
|  | *Ampharete finmarchica* | 30 | 0.0414 |
|  | *Arctica islandica* | 8 | 3.8798 |
|  | *Aricidea wassi* | 6 | 0.0166 |
|  | Ascidiacea (solitary) | 8 | 0.0476 |
|  | *Chaetozone* sp. A | 8 | 0.005 |
|  | *Chiridotea tuftsii* | 2 | 0.0096 |
|  | *Chone* sp. | 2 | 0.0024 |
|  | *Cirolana polita* | 2 | 0.01 |
|  | *Cistenides granulata* | 6 | 0.0218 |
|  | *Clymenura borealis* | 22 | 2.4544 |
|  | *Colus stimpsoni* | 2 | 27.32 |
|  | *Corophium crassicorne* | 10 | 0.0268 |
|  | *Crangon septemspinosa* | 6 | 0.074 |
|  | *Cylichna alba* | 10 | 0.384 |
|  | *Cyrtodaria siliqua* | 4 | 249.0632 |
|  | *Diaphana minuta* | 2 | 0.0124 |
|  | *Echinarachnius parma* | 32 | 581.8032 |
|  | *Edotea montosa* | 10 | 0.0268 |
|  | *Edwardsia elegans* | 20 | 0.8546 |
|  | *Euchone papillosa* | 4 | 0.0058 |
|  | *Euclymene zonalis* | 4 | 0.0484 |
|  | *Exogone* sp. | 2 | 0.0002 |
|  | *Hippomedon serratus* | 10 | 0.06 |
|  | *Leptognathia* sp. | 4 | 0.0006 |
|  | *Levensinea gracilis* | 18 | 0.0094 |
|  | *Lumbrinerides acuta* | 64 | 0.3986 |
|  | Lysianassidae | 2 | 0.0014 |
|  | *Monoculodes* sp. | 4 | 0.0072 |
|  | Mytiloidea | 2 | 0.0028 |
|  | Nemertea | 4 | 0.1016 |
|  | *Nephtys bucera* | 6 | 0.6472 |
|  | *Nephtys caeca* | 2 | 0.5404 |
|  | *Notomastus latericeus* | 2 | 0.0514 |
|  | Oligochaeta | 10 | 0.0276 |
|  | *Ophelia limacina* | 6 | 0.1824 |
|  | *Orbinia swani* | 2 | 0.1986 |
|  | *Pandora* sp. | 2 | 0.7598 |
|  | *Periploma leanum* | 2 | 1.202 |
|  | *Photis* sp. | 4 | 0.001 |
|  | *Phoxocephalus holbolli* | 20 | 0.036 |
|  | Platyhelminthes | 10 | 0.067 |
|  | *Spiophanes bombyx* | 2 | 0.0018 |
|  | *Tharyx* sp. | 12 | 0.0344 |
| wc_vg09 | Actiniaria (burrowing) | 6 | 0.3378 |
|  | *Aglaophamus circinata* | 8 | 1.8756 |
|  | *Alvania* sp. | 2 | 0.001 |
|  | *Ampharete finmarchica* | 44 | 0.096 |
|  | *Anonyx sarsi* | 4 | 0.0356 |
|  | *Aricidea catherinae* | 2 | 0.004 |
|  | *Aricidea* sp. B | 8 | 0.0082 |
|  | *Aricidea wassi* | 2 | 0.0146 |
|  | Ascidiacea (solitary) | 12 | 0.149 |
|  | Campanulariidae | 2 | 0.017 |
|  | *Cancer borealis* | 2 | 0.0408 |
|  | *Capitella capitata* | 10 | 0.0216 |
|  | Capitellidae | 14 | 0.0102 |
|  | *Chaetozone setosa* | 2 | 0.003 |
|  | *Chaetozone* sp. A | 38 | 0.0652 |
|  | *Cistenides granulata* | 6 | 0.0016 |
|  | *Clymenura borealis* | 36 | 2.1226 |
|  | *Crangon septemspinosa* | 22 | 0.2932 |
|  | *Cylichna alba* | 8 | 0.3378 |
|  | *Cyrtodaria siliqua* | 12 | 322.5462 |
|  | *Diaphana minuta* | 8 | 0.0146 |
|  | *Dyopedos* sp. | 2 | 0.0018 |
|  | *Echinarachnius parma* | 22 | 288.1796 |
|  | *Edotea montosa* | 4 | 0.0088 |
|  | *Edwardsia elegans* | 60 | 2.6634 |
|  | *Euchone incolor* | 4 | 0.0044 |
|  | *Euchone papillosa* | 10 | 0.0274 |
|  | *Euclymene zonalis* | 2 | 0.0022 |
|  | *Euspira* sp. | 2 | 0.0006 |
|  | *Exogone* sp. | 76 | 0.0286 |
|  | *Glycera capitata* | 4 | 0.0262 |
|  | *Goniadella gracilis* | 4 | 0.0054 |
|  | *Harmothoe extenuata* | 2 | 0.03 |
|  | *Hippomedon serratus* | 10 | 0.0516 |
|  | *Ischyrocerus* sp. | 2 | 0.0038 |
|  | *Leptognathia* sp. | 2 | 0.0012 |
|  | *Levensinea gracilis* | 12 | 0.0092 |
|  | *Lumbrinerides acuta* | 76 | 1.1084 |
|  | *Mactromeris polynyma* | 2 | 0.0004 |
|  | *Modiolus modiolus* | 2 | 0.0016 |
|  | Nemertea | 12 | 0.0138 |
|  | *Nephtys caeca* | 8 | 1.6122 |
|  | *Notomastus latericeus* | 4 | 0.2594 |
|  | Oligochaeta | 36 | 0.0196 |
|  | *Ophelia limacina* | 18 | 10.2334 |
|  | *Paraonis* sp. | 2 | 0.0014 |
|  | *Parvicardium pinnulatum* | 10 | 0.0008 |
|  | *Pholoe tecta* | 2 | 0.0006 |
|  | *Phoxocephalus holbolli* | 22 | 0.0346 |
|  | *Platyhelminthes* | 8 | 0.4014 |
|  | Polychaeta | 10 | 0.007 |
|  | Polygordiidae | 246 | 0.1068 |
|  | *Protodorvillea kefersteini* | 6 | 0.001 |
|  | *Psammonyx* sp. | 14 | 0.3124 |
|  | *Psolus* sp. | 2 | 0.0008 |
|  | *Spio filicornis* | 6 | 0.0076 |
|  | *Spiophanes bombyx* | 138 | 0.1622 |
|  | Syllidae | 18 | 0.008 |
|  | *Tharyx* sp. | 16 | 0.0334 |
|  | *Unciola irrorata* | 26 | 0.065 |
| wc_vg10 | *Aglaophamus circinata* | 2 | 0.1696 |
|  | *Ampharete finmarchica* | 36 | 0.0768 |
|  | *Aphrodita hastata* | 2 | 101.8 |
|  | *Arctica islandica* | 2 | 252.4 |
|  | *Aricidea* sp. B | 4 | 0.0064 |
|  | Campanulariidae | 2 | 0.0004 |
|  | *Cancer borealis* | 2 | 0.3156 |
|  | *Capitella capitata* | 2 | 0.0058 |
|  | *C*apitellidae | 8 | 0.006 |
|  | *Chaetozone* sp. A | 22 | 0.0788 |
|  | *Cistenides granulata* | 4 | 0.0058 |
|  | *Clymenura borealis* | 16 | 0.7728 |
|  | *Cylichna alba* | 6 | 0.3232 |
|  | *Cyrtodaria siliqua* | 4 | 117.0002 |
|  | *Echinarachnius parma* | 28 | 110.2978 |
|  | *Edwardsia elegans* | 40 | 1.3186 |
|  | *Euchone papillosa* | 6 | 0.0272 |
|  | *Euclymene zonalis* | 2 | 0.019 |
|  | *Hippomedon serratus* | 6 | 0.0942 |
|  | *Levensinea gracilis* | 6 | 0.0028 |
|  | *Lumbrinerides acuta* | 42 | 0.4336 |
|  | Lysianassidae | 6 | 0.0164 |
|  | Mytiloidea | 2 | 0.0008 |
|  | *Nephtys bucera* | 2 | 0.047 |
|  | *Notomastus latericeus* | 2 | 0.0026 |
|  | Oligochaeta | 2 | 0.0188 |
|  | *Ophelia limacina* | 10 | 11.87 |
|  | *Orbinia swani* | 4 | 0.9452 |
|  | *Paraonis* sp. | 2 | 0.002 |
|  | *Parougia eliasoni* | 2 | 0.001 |
|  | *Periploma leanum* | 2 | 1.3698 |
|  | *Phoxocephalus holbolli* | 2 | 0.0034 |
|  | *Phyllodoce groenlandica* | 2 | 0.012 |
|  | Platyhelminthes | 4 | 0.0222 |
|  | Polygordiidae | 4 | 0.0014 |
|  | *Protomedeia fasciata* | 2 | 0.0014 |
|  | *Scolelepis* sp. A | 2 | 0.0624 |
|  | *Spio filicornis* | 2 | 0.003 |
|  | *Spiophanes bombyx* | 16 | 0.0664 |
|  | Syllidae | 2 | 0.0002 |
|  | *Unciola irrorata* | 8 | 0.0202 |
| wh_evg01 | *Aeginina longicornis* | 4 | 0.0158 |
|  | *Ampharete finmarchica* | 2 | 0.0016 |
|  | Amphipoda | 2 | 0.008 |
|  | *Arctica islandica* | 2 | 5.4102 |
|  | *Asabellides* sp. | 4 | 0.0026 |
|  | Ascidiacea (solitary) | 8 | 0.0188 |
|  | Asteroidea | 6 | 0.0024 |
|  | *Balanus* sp. | 2 | 0.0052 |
|  | Campanulariidae | 2 | 0.0256 |
|  | *Cancer borealis* | 4 | 0.0182 |
|  | *Caprella* sp. | 4 | 0.001 |
|  | Cerianthidae | 4 | 3.5144 |
|  | *Chaetozone* sp. A | 30 | 0.0296 |
|  | *Chone* sp. | 48 | 8.5804 |
|  | *Cistenides granulata* | 142 | 0.0378 |
|  | *Clymenura borealis* | 6 | 0.0236 |
|  | *Cucumaria frondosa* | 184 | 0.3086 |
|  | *Cylichna alba* | 12 | 0.0102 |
|  | *Cyrtodaria siliqua* | 34 | 15.2592 |
|  | *Drilonereis magna* | 8 | 0.3772 |
|  | *Edwardsia elegans* | 26 | 4.8382 |
|  | *Eteone longa* | 16 | 0.0654 |
|  | *Eualus pusiolus* | 50 | 0.3144 |
|  | *Euchone papillosa* | 4 | 0.0062 |
|  | *Euclymene zonalis* | 510 | 10.1716 |
|  | *Eucratea loricata* | 2 | 0.158 |
|  | *Eulalia bilineata* | 4 | 0.0828 |
|  | *Exogone* sp. | 2 | 0.0002 |
|  | *Glycera capitata* | 190 | 4.8906 |
|  | *Goniadella gracilis* | 2 | 0.0048 |
|  | *Harmothoe extenuata* | 60 | 0.3548 |
|  | *Harmothoe imbricata* | 16 | 0.7794 |
|  | *Hiatella arctica* | 2 | 0.0016 |
|  | *Ischyrocerus* sp. | 4 | 0.0012 |
|  | *Laonice cirrata* | 4 | 0.002 |
|  | *Laphania boecki* | 4 | 0.1418 |
|  | *Lichenopora* sp. | 2 | 0.0002 |
|  | *Lumbrinerides acuta* | 8 | 0.0898 |
|  | Lysianassidae | 2 | 0.003 |
|  | *Melita dentata* | 24 | 0.1284 |
|  | *Moelleria costulata* | 2 | 0.0018 |
|  | *Monoculodes* sp. | 2 | 0.0006 |
|  | *Monoculodes tuberculatus* | 10 | 0.0022 |
|  | Mytiloidea | 12 | 0.0232 |
|  | Nemertea | 6 | 0.1482 |
|  | *Neosabellides* sp. | 4 | 0.0098 |
|  | *Nephtys caeca* | 2 | 1.2856 |
|  | *Nereis* sp. | 2 | 0.0292 |
|  | Oligochaeta | 34 | 0.0938 |
|  | *Ophelia limacina* | 2 | 0.0204 |
|  | *Ophiopholis aculeata* | 6 | 0.0522 |
|  | *Ophiura robusta* | 336 | 0.8282 |
|  | *Ophiura* sp. juvenile | 28 | 0.0292 |
|  | *Paraonis* sp. | 6 | 0.0048 |
|  | *Parvicardium pinnulatum* | 6 | 0.6532 |
|  | *Pholoe minuta* | 70 | 0.0288 |
|  | *Pholoe tecta* | 2 | 0.0054 |
|  | *Phyllodoce maculata* | 10 | 0.0344 |
|  | *Placopecten magellanicus* | 4 | 0.0884 |
|  | Platyhelminthes | 18 | 0.197 |
|  | *Pleustes panoplus* | 2 | 0.0038 |
|  | *Pleusymtes glaber* | 6 | 0.0098 |
|  | *Polycirrus* sp. | 10 | 0.2232 |
|  | *Polydora concharum* | 6 | 0.0518 |
|  | Polygordiidae | 4 | 0.0004 |
|  | *Protomedeia fasciata* | 8 | 0.0088 |
|  | *Psolus* sp. | 226 | 0.35 |
|  | *Scoloplos armiger* | 22 | 0.4302 |
|  | *Spio filicornis* | 4 | 0.0058 |
|  | *Spiophanes bombyx* | 4 | 0.0032 |
|  | *Strongylocentrotus pallidus* | 138 | 0.7192 |
|  | *Tharyx* sp. | 18 | 0.0408 |
|  | *Thracia* sp. | 48 | 0.0766 |
|  | *Travisia* sp. | 18 | 0.5994 |
|  | *Unciola irrorata* | 226 | 1.1186 |
| wh_evg02 | *Acanthohaustorius spinosus* | 2 | 0.0956 |
|  | *Aglaophamus circinata* | 6 | 2.0912 |
|  | *Ampharete finmarchica* | 28 | 0.0454 |
|  | *Anonyx sarsi* | 2 | 0.0166 |
|  | *Arctica islandica* | 70 | 158.04 |
|  | *Aricidea* sp. B | 4 | 0.0038 |
|  | *Aricidea wassi* | 18 | 0.0552 |
|  | Ascidiacea (solitary) | 4 | 0.275 |
|  | *Capitella capitata* | 2 | 0.0018 |
|  | Capitellidae | 12 | 0.0142 |
|  | *Chaetozone* sp. A | 26 | 0.0224 |
|  | *Chiridotea tuftsii* | 2 | 0.0076 |
|  | *Cirolana polita* | 8 | 0.061 |
|  | *Cistenides granulata* | 8 | 0.0068 |
|  | *Clymenura borealis* | 50 | 6.2632 |
|  | *Corophium crassicorne* | 20 | 0.0172 |
|  | *Cucumaria frondosa* | 4 | 0.0076 |
|  | *Cylichna alba* | 2 | 0.1048 |
|  | *Cyrtodaria siliqua* | 8 | 541.9644 |
|  | *Echinarachnius parma* | 30 | 227.9448 |
|  | *Edwardsia elegans* | 36 | 0.6852 |
|  | *Euchone papillosa* | 4 | 0.0134 |
|  | *Euclymene zonalis* | 12 | 0.0856 |
|  | *Eudorellopsis deformis* | 2 | 0.001 |
|  | *Exogone* sp. | 6 | 0.0008 |
|  | *Glycera capitata* | 2 | 0.0208 |
|  | *Hippomedon serratus* | 2 | 0.0106 |
|  | *Laphania boecki* | 2 | 0.0028 |
|  | *Leptognathia* sp. | 2 | 0.0002 |
|  | *Levensinea gracilis* | 32 | 0.021 |
|  | *Lumbrinerides acuta* | 12 | 0.1352 |
|  | *Mactromeris polynyma* | 6 | 127.42 |
|  | Mytiloidea | 2 | 0.0188 |
|  | Nemertea | 6 | 2.5952 |
|  | Nephtyidae sp. juvenile | 8 | 0.0558 |
|  | *Nephtys bucera* | 2 | 0.5278 |
|  | *Nephtys caeca* | 4 | 4.4442 |
|  | *Nereis* sp. | 2 | 0.013 |
|  | *Notomastus latericeus* | 6 | 0.0614 |
|  | *Ophelia limacina* | 38 | 1.3674 |
|  | *Ophiura robusta* | 4 | 0.0286 |
|  | *Ophiura* sp. juvenile | 2 | 0.0024 |
|  | *Orbinia swani* | 4 | 0.4632 |
|  | *Pandora* sp. | 2 | 0.775 |
|  | *Paraonis* sp. | 6 | 0.013 |
|  | *Parvicardium pinnulatum* | 2 | 0.0002 |
|  | *Photis* sp. | 2 | 0.0018 |
|  | *Phoxocephalus holbolli* | 4 | 0.0028 |
|  | *Phyllodoce maculata* | 26 | 0.0966 |
|  | *Phyllodoce mucosa* | 2 | 0.0074 |
|  | Platyhelminthes | 8 | 0.0876 |
|  | *Pleusymtes glaber* | 2 | 0.001 |
|  | *Polydora socialis* | 20 | 0.022 |
|  | Polygordiidae | 10 | 0.0022 |
|  | *Pseudounicola obliquua* | 54 | 0.045 |
|  | *Scoloplos armiger* | 30 | 0.9256 |
|  | *Solariella obscura* | 12 | 0.1994 |
|  | *Spiophanes bombyx* | 50 | 0.2106 |
|  | *Tharyx* sp. | 24 | 0.0556 |
|  | *Thracia* sp. | 2 | 0.0034 |
|  | *Unciola irrorata* | 236 | 1.0526 |
| wh_vg01 | *Aeginina longicornis* | 18 | 0.1096 |
|  | *Ampharete finmarchica* | 42 | 0.0632 |
|  | *Aphrodita hastata* | 2 | 0.0002 |
|  | *Aricidea catherinae* | 168 | 0.1006 |
|  | *Asabellides* sp. | 6 | 0.0278 |
|  | Ascidiacea (solitary) | 4 | 0.007 |
|  | *Astarte* sp. | 2 | 0.0006 |
|  | *Boreocingula* sp. | 22 | 0.0078 |
|  | Campanulariidae | 2 | 0.0066 |
|  | *Capitella capitata* | 2 | 0.0002 |
|  | Capitellidae | 6 | 0.001 |
|  | *Caprella* sp. | 4 | 0.0082 |
|  | Cerianthidae | 8 | 1.0938 |
|  | *Chaetozone setosa* | 10 | 0.0302 |
|  | *Chaetozone* sp. A | 172 | 0.112 |
|  | *Chlamys islandica* | 2 | 0.0002 |
|  | *Chone* sp. | 274 | 51.8596 |
|  | *Cistenides granulata* | 56 | 0.0404 |
|  | *Clymenura borealis* | 606 | 4.1758 |
|  | *Cucumaria frondosa* | 12 | 0.0042 |
|  | *Cylichna alba* | 6 | 0.1358 |
|  | *Cyrtodaria siliqua* | 6 | 0.0018 |
|  | *Dodecaceria fimbriata* | 2 | 0.0036 |
|  | *Drilonereis magna* | 4 | 0.1468 |
|  | *Dyopedos* sp. | 2 | 0.0002 |
|  | *Edwardsia elegans* | 180 | 16.152 |
|  | *Ericthonius fasciatus* | 4 | 0.0122 |
|  | *Eteone longa* | 8 | 0.0212 |
|  | *Eualus pusiolus* | 2 | 0.0016 |
|  | *Euchone papillosa* | 12 | 0.0574 |
|  | *Eulalia bilineata* | 2 | 0.0072 |
|  | *Eulalia viridis* | 4 | 0.0006 |
|  | *Euspira* sp. | 4 | 0.038 |
|  | *Exogone* sp. | 194 | 0.0218 |
|  | Gastropoda | 2 | 0.0966 |
|  | *Glycera capitata* | 88 | 1.8202 |
|  | *Grammaria abietina* | 2 | 0.0682 |
|  | *Guernea nordenskioldi* | 2 | 0.001 |
|  | *Harmothoe extenuata* | 8 | 0.1964 |
|  | *Ischyrocerus* sp. | 8 | 0.0004 |
|  | *Laphania boecki* | 4 | 0.0448 |
|  | *Maera danae* | 48 | 0.6142 |
|  | *Mediomastus ambiseta* | 18 | 0.014 |
|  | *Microphthalmus aberrans* | 2 | 0.0002 |
|  | *Moelleria costulata* | 18 | 0.006 |
|  | *Monoculodes tuberculatus* | 2 | 0.0002 |
|  | *Musculus* sp. | 2 | 3.2254 |
|  | *Mysta barbata* | 2 | 0.0002 |
|  | Mytiloidea | 358 | 1.051 |
|  | Nemertea | 12 | 0.3692 |
|  | Nephtyidae sp. juvenile | 6 | 0.0042 |
|  | *Nephtys caeca* | 4 | 38.4432 |
|  | *Nereis* sp. | 10 | 0.1084 |
|  | Oligochaeta | 4 | 0.0002 |
|  | *Ophelia limacina* | 18 | 0.0408 |
|  | *Ophelina acuminata* | 2 | 0.0002 |
|  | *Orbinia swani* | 6 | 0.0388 |
|  | Ostracoda | 4 | 0.0006 |
|  | *Owenia fusiformis* | 2 | 0.0036 |
|  | *Pagurus* sp. | 2 | 0.0388 |
|  | *Paraonis* sp. | 22 | 0.0164 |
|  | *Parougia eliasoni* | 8 | 0.0002 |
|  | *Parvicardium pinnulatum* | 4 | 0.2606 |
|  | *Periploma leanum* | 10 | 0.0056 |
|  | *Pherusa plumosa* | 4 | 0.0002 |
|  | *Pholoe tecta* | 46 | 0.017 |
|  | *Photis* sp. | 10 | 0.004 |
|  | *Phoxocephalus holbolli* | 2 | 0.0016 |
|  | *Phyllodoce groenlandica* | 4 | 0.012 |
|  | *Phyllodoce maculata* | 152 | 0.0794 |
|  | *Phyllodoce mucosa* | 22 | 0.0258 |
|  | Phyllodocidae sp. juvenile | 6 | 0.0006 |
|  | Platyhelminthes | 2 | 0.0168 |
|  | *Polycirrus* sp. | 10 | 0.1186 |
|  | *Polydora socialis* | 72 | 0.0596 |
|  | Polygordiidae | 48 | 0.0096 |
|  | *Protodorvillea gaspeensis* | 30 | 0.0016 |
|  | *Protodorvillea kefersteini* | 20 | 0.0006 |
|  | *Protomedeia fasciata* | 10 | 0.003 |
|  | *Psolus* sp. | 4 | 0.0046 |
|  | *Rhizocaulus verticillatus* | 2 | 0.177 |
|  | *Scoloplos armiger* | 2 | 0.0088 |
|  | *Scrupocellaria scabra* | 2 | 0.0002 |
|  | *Sertularia* sp. | 2 | 0.3394 |
|  | Sipuncula | 2 | 0.0002 |
|  | *Spiophanes bombyx* | 2 | 0.0008 |
|  | *Stenosemus albus* | 2 | 0.1534 |
|  | *Sthenelais limicola* | 2 | 0.0892 |
|  | Syllidae | 42 | 0.0012 |
|  | *Tachyrhynchus reticulatus* | 2 | 0.256 |
|  | *Tharyx* sp. | 152 | 0.3394 |
|  | *Thelepus cincinnatus* | 2 | 0.2154 |
|  | *Thracia* sp. | 8 | 0.0032 |
|  | *Travisia* sp. | 12 | 0.2002 |
|  | *Unciola irrorata* | 548 | 2.7422 |
| wh_vg02 | *Alvania* sp. | 6 | 0.0076 |
|  | *Ampharete finmarchica* | 70 | 0.1748 |
|  | *Aphrodita hastata* | 2 | 0.0008 |
|  | *Arctica islandica* | 6 | 23.162 |
|  | Ascidiacea (solitary) | 38 | 0.172 |
|  | Bivalvia | 2 | 0.0018 |
|  | Capitellidae | 2 | 0.0002 |
|  | Cerianthidae | 4 | 1.2976 |
|  | *Chaetozone* sp. A | 32 | 0.0458 |
|  | *Chone* sp. | 16 | 0.1144 |
|  | *Cirolana polita* | 2 | 0.0092 |
|  | *Cistenides granulata* | 106 | 0.0422 |
|  | *Clymenura borealis* | 24 | 3.1568 |
|  | *Corophium crassicorne* | 4 | 0.0022 |
|  | *Cucumaria frondosa* | 92 | 0.0658 |
|  | *Cylichna alba* | 26 | 0.1306 |
|  | *Cyrtodaria siliqua* | 42 | 231.9466 |
|  | *Drilonereis magna* | 10 | 1.4936 |
|  | *Echinarachnius parma* | 4 | 0.0014 |
|  | *Edwardsia elegans* | 172 | 10.2972 |
|  | *Epitonium greenlandicum* | 2 | 0.6734 |
|  | *Ericthonius fasciatus* | 10 | 0.0096 |
|  | *Eteone longa* | 6 | 0.0396 |
|  | *Euchone papillosa* | 34 | 0.204 |
|  | *Euclymene zonalis* | 980 | 12.984 |
|  | *Eudorellopsis deformis* | 2 | 0.0014 |
|  | *Euspira* sp. | 6 | 0.033 |
|  | *Exogone* sp. | 2 | 0.0026 |
|  | Gastropoda | 2 | 0.001 |
|  | *Glycera capitata* | 78 | 4.7372 |
|  | *Harmothoe extenuata* | 2 | 0.0024 |
|  | *Laphania boecki* | 8 | 0.0838 |
|  | *Leptocheirus pinguis* | 2 | 0.0568 |
|  | *Lumbrinerides acuta* | 28 | 0.5802 |
|  | *Mediomastus ambiseta* | 2 | 0.0018 |
|  | *Moelleria costulata* | 10 | 0.0182 |
|  | *Monoculodes* sp. | 4 | 0.005 |
|  | *Musculus* sp. | 6 | 0.1972 |
|  | Mytiloidea | 24 | 0.7522 |
|  | Nemertea | 6 | 0.2462 |
|  | Nephtyidae sp. juvenile | 2 | 0.0042 |
|  | *Nereis* sp. | 12 | 0.1654 |
|  | *Notomastus latericeus* | 26 | 0.9198 |
|  | Oligochaeta | 66 | 0.2742 |
|  | *Ophelia limacina* | 52 | 0.2898 |
|  | *Ophiura sarsi* | 2 | 0.0346 |
|  | *Orbinia swani* | 16 | 0.882 |
|  | *Pagurus* sp. | 2 | 0.0108 |
|  | *Paraonis* sp. | 60 | 0.0458 |
|  | *Parougia eliasoni* | 2 | 0.0002 |
|  | *Parvicardium pinnulatum* | 14 | 1.3404 |
|  | *Pholoe tecta* | 16 | 0.0068 |
|  | *Photis* sp. | 4 | 0.0078 |
|  | *Phyllodoce groenlandica* | 2 | 0.0002 |
|  | *Phyllodoce mucosa* | 30 | 0.1396 |
|  | Platyhelminthes | 20 | 0.2274 |
|  | *Polycirrus* sp. | 4 | 0.1046 |
|  | Polygordiidae | 8 | 0.0016 |
|  | *Protomedeia fasciata* | 14 | 0.009 |
|  | *Scoloplos armiger* | 14 | 0.3814 |
|  | *Spio filicornis* | 4 | 0.011 |
|  | *Spiophanes bombyx* | 44 | 0.03 |
|  | Syllidae | 6 | 0.0006 |
|  | *Tharyx* sp. | 70 | 0.244 |
|  | *Thracia* sp. | 48 | 1.218 |
|  | *Unciola irrorata* | 1250 | 4.3216 |
| wh_vg03 | Actiniaria (burrowing) | 16 | 0.2942 |
|  | Actiniaria (non burrowing) | 14 | 0.1248 |
|  | *Aeginina longicornis* | 28 | 0.1014 |
|  | *Alvania* sp. | 2 | 0.0004 |
|  | *Ampharete finmarchica* | 108 | 0.1788 |
|  | *Anonyx sarsi* | 2 | 0.0186 |
|  | *Aricidea catherinae* | 8 | 0.0046 |
|  | Ascidiacea (solitary) | 4 | 0.006 |
|  | Asteroidea | 4 | 0.0034 |
|  | *Cancer borealis* | 2 | 0.0044 |
|  | *Caprella* sp. | 26 | 0.0362 |
|  | *Cauloramphus* sp. | 2 | 0.4834 |
|  | *Celleporella* sp. | 2 | 0.6936 |
|  | Cerianthidae | 8 | 4.912 |
|  | *Chaetozone* sp. A | 18 | 0.0556 |
|  | *Chlamys islandica* | 2 | 164.96 |
|  | *Chone* sp. | 60 | 0.122 |
|  | *Cistenides granulata* | 40 | 0.0188 |
|  | *Clymenura borealis* | 28 | 1.8368 |
|  | *Crangon septemspinosa* | 6 | 0.1092 |
|  | *Cucumaria frondosa* | 44 | 2949.6836 |
|  | *Cylichna alba* | 14 | 0.0412 |
|  | *Cyrtodaria siliqua* | 18 | 0.0338 |
|  | *Diaphana minuta* | 2 | 0.0002 |
|  | *Doto* sp. | 4 | 0.1354 |
|  | *Edwardsia elegans* | 32 | 4.2846 |
|  | *Ericthonius fasciatus* | 2 | 0.0002 |
|  | *Eteone longa* | 8 | 0.0246 |
|  | *Euchone papillosa* | 58 | 0.195 |
|  | *Euclymene zonalis* | 356 | 2.4152 |
|  | *Euspira* sp. | 2 | 0.0034 |
|  | *Exogone* sp. | 16 | 0.0072 |
|  | *Gastropoda* | 2 | 0.0084 |
|  | *Gattyana nutti* | 2 | 0.0048 |
|  | *Glycera capitata* | 80 | 2.298 |
|  | *Harmothoe extenuata* | 8 | 0.0478 |
|  | *Harmothoe imbricata* | 2 | 1.0762 |
|  | Invert. undetermined | 2 | 0.0014 |
|  | *Ischyrocerus* sp. | 4 | 0.0012 |
|  | *Laphania boecki* | 6 | 0.019 |
|  | *Lumbrinerides acuta* | 16 | 0.2656 |
|  | *Mediomastus ambiseta* | 2 | 0.004 |
|  | *Melita dentata* | 2 | 0.0162 |
|  | *Modiolus modiolus* | 2 | 0.639 |
|  | *Moelleria costulata* | 8 | 0.0048 |
|  | *Monoculodes* sp. | 2 | 0.0072 |
|  | Mytiloidea | 26 | 0.3662 |
|  | Nemertea | 10 | 0.144 |
|  | Nephtyidae sp. juvenile | 2 | 0.0048 |
|  | *Nereis* sp. | 2 | 0.0054 |
|  | *Notomastus latericeus* | 2 | 0.0414 |
|  | Oligochaeta | 18 | 0.0096 |
|  | *Ophelia limacina* | 6 | 0.0162 |
|  | *Ophiopholis aculeata* | 2 | 0.0764 |
|  | *Ophiura sarsi* | 20 | 0.2256 |
|  | *Orbinia swani* | 16 | 0.15 |
|  | *Paraonis* sp. | 28 | 0.0374 |
|  | *Parvicardium pinnulatum* | 36 | 0.1766 |
|  | *Periploma leanum* | 14 | 0.0124 |
|  | *Philine* sp. | 6 | 0.0098 |
|  | *Pholoe tecta* | 16 | 0.0078 |
|  | *Photis* sp. | 6 | 0.0068 |
|  | *Phyllodoce maculata* | 38 | 0.0438 |
|  | *Phyllodoce mucosa* | 4 | 0.041 |
|  | Platyhelminthes | 14 | 0.1306 |
|  | *Pleusymtes glaber* | 2 | 0.0012 |
|  | Polychaeta | 2 | 0.0002 |
|  | Polygordiidae | 78 | 0.045 |
|  | Polynoidae sp. juvenile | 2 | 0.0002 |
|  | *Pontogeneia inermis* | 2 | 0.0058 |
|  | *Protodorvillea kefersteini* | 2 | 0.002 |
|  | *Protomedeia fasciata* | 88 | 0.0614 |
|  | *Psolus* sp. | 28 | 0.032 |
|  | Questidae | 4 | 0.0002 |
|  | *Rhizocaulus verticillatus* | 2 | 0.6466 |
|  | Sabellidae | 2 | 0.0002 |
|  | *Scolelepis squamata* | 2 | 0.0774 |
|  | *Sertularia polyzonias* | 2 | 0.1046 |
|  | *Sertularia* sp. | 2 | 5.0203 |
|  | *Sertularia tricuspidatus* | 2 | 0.0178 |
|  | Sipuncula | 2 | 0.0002 |
|  | *Spiophanes bombyx* | 14 | 0.0492 |
|  | Spirorbidae | 20 | 0.0122 |
|  | *Strongylocentrotus pallidus* | 6 | 0.0148 |
|  | Syllidae | 12 | 0.002 |
|  | *Tharyx* sp. | 28 | 0.0564 |
|  | *Thracia* sp. | 32 | 0.0128 |
|  | *Thuiaria* sp. | 2 | 0.4604 |
|  | *Travisia* sp. | 2 | 0.0398 |
|  | *Unciola irrorata* | 360 | 1.1582 |
|  | *Velutina* sp. | 2 | 0.0088 |
| wh_vg04 | Actiniaria (burrowing) | 2 | 0.7354 |
|  | Actiniaria (non burrowing) | 10 | 0.0552 |
|  | *Aeginina longicornis* | 8 | 0.0402 |
|  | *Ampharete finmarchica* | 100 | 0.1772 |
|  | Amphipoda | 2 | 0.0022 |
|  | *Anonyx sarsi* | 10 | 0.4916 |
|  | *Aphrodita hastata* | 2 | 0.0018 |
|  | *Arctica islandica* | 2 | 0.2596 |
|  | *Aricidea catherinae* | 4 | 0.004 |
|  | *Asabellides* sp. | 2 | 0.0058 |
|  | Ascidiacea (solitary) | 18 | 0.2932 |
|  | *Cancer irroratus* | 2 | 11.0214 |
|  | *Caprella* sp. | 24 | 0.0394 |
|  | *Cauloramphus* sp. | 2 | 0.0096 |
|  | Cerianthidae | 16 | 32.8814 |
|  | *Chaetozone* sp. A | 14 | 0.0398 |
|  | *Chone* sp. | 82 | 1.0706 |
|  | *Cistenides granulata* | 114 | 0.1718 |
|  | *Clymenura borealis* | 10 | 3.4242 |
|  | *Cucumaria frondosa* | 74 | 32956.735 |
|  | *Cylichna alba* | 8 | 0.0884 |
|  | *Cyrtodaria siliqua* | 44 | 0.1736 |
|  | *Diaphana minuta* | 2 | 0.0018 |
|  | *Drilonereis magna* | 12 | 0.4708 |
|  | *Edwardsia elegans* | 80 | 4.5868 |
|  | *Ericthonius fasciatus* | 8 | 0.0068 |
|  | *Eteone longa* | 18 | 0.1058 |
|  | *Eualus pusiolus* | 58 | 0.513 |
|  | *Euchone papillosa* | 54 | 0.2786 |
|  | *Euclymene zonalis* | 1392 | 19.164 |
|  | *Eucratea loricata* | 2 | 0.054 |
|  | *Exogone* sp. | 22 | 0.0034 |
|  | Gastropoda | 2 | 0.0002 |
|  | *Glycera capitata* | 256 | 13.1574 |
|  | *Goniadella gracilis* | 4 | 0.013 |
|  | *Haplopoma* sp. | 2 | 0.01 |
|  | *Harmothoe extenuata* | 42 | 0.3812 |
|  | *Harmothoe imbricata* | 20 | 0.8506 |
|  | *Hippomedon serratus* | 2 | 0.004 |
|  | *Hyas coarctatus* | 2 | 0.0076 |
|  | Invert. undetermined | 2 | 0.005 |
|  | *Ischyrocerus* sp. | 2 | 0.0018 |
|  | *Laphania boecki* | 2 | 0.2654 |
|  | *Lumbrinerides acuta* | 20 | 0.4374 |
|  | *Melita dentata* | 22 | 0.312 |
|  | *Moelleria costulata* | 6 | 0.006 |
|  | *Monoculodes tuberculatus* | 10 | 0.0022 |
|  | *Musculus* sp. | 2 | 3.3278 |
|  | Mytiloidea | 36 | 0.5462 |
|  | Nemertea | 18 | 0.185 |
|  | *Nereis* sp. | 2 | 0.0028 |
|  | *Notomastus latericeus* | 6 | 0.1256 |
|  | Oligochaeta | 52 | 0.0864 |
|  | *Ophelia limacina* | 4 | 1.103 |
|  | *Ophiura robusta* | 158 | 0.159 |
|  | *Orbinia swani* | 8 | 0.1512 |
|  | *Pagurus* sp. | 6 | 0.0176 |
|  | *Paraonis* sp. | 70 | 0.0796 |
|  | *Parvicardium pinnulatum* | 16 | 0.1522 |
|  | *Periploma leanum* | 2 | 0.0038 |
|  | *Pholoe minuta* | 52 | 0.0308 |
|  | *Photis* sp. | 4 | 0.0026 |
|  | *Phyllodoce maculata* | 12 | 0.0124 |
|  | *Phyllodoce mucosa* | 8 | 0.0352 |
|  | Platyhelminthes | 24 | 0.176 |
|  | *Pleusymtes glaber* | 20 | 0.0122 |
|  | *Polycirrus* sp. | 10 | 0.4486 |
|  | *Polydora caulleryi* | 2 | 0.0252 |
|  | *Polydora socialis* | 26 | 0.0116 |
|  | Polygordiidae | 74 | 0.057 |
|  | *Pontogeneia inermis* | 20 | 0.058 |
|  | *Protomedeia fasciata* | 146 | 0.0766 |
|  | *Psolus* sp. | 106 | 0.141 |
|  | *Rhizocaulus verticillatus* | 2 | 1.4972 |
|  | *Scolelepis squamata* | 2 | 0.2746 |
|  | *Scoloplos armiger* | 4 | 0.0902 |
|  | *Sertularia polyzonias* | 2 | 0.4434 |
|  | *Sertularia* sp. | 2 | 0.001 |
|  | *Solariella obscura* | 4 | 0.164 |
|  | *Spio filicornis* | 4 | 0.0028 |
|  | *Spiophanes bombyx* | 38 | 0.0942 |
|  | *Strongylocentrotus* sp. | 52 | 0.2488 |
|  | Syllidae | 14 | 0.004 |
|  | *Syrrhoe crenulata* | 2 | 0.0106 |
|  | *Tharyx* sp. | 52 | 0.1524 |
|  | *Thracia* sp. | 64 | 0.082 |
|  | *Tiron spiniferus* | 2 | 0.0016 |
|  | *Travisia* sp. | 2 | 0.0166 |
|  | *Tricellaria gracilis* | 2 | 0.3354 |
|  | *Unciola irrorata* | 484 | 1.5794 |
| wh_vg05 | Actiniaria (burrowing) | 6 | 1.4098 |
|  | Onchidoridae | 10 | 0.0046 |
|  | *Aeginina longicornis* | 6 | 0.0216 |
|  | *Ampharete finmarchica* | 138 | 0.4118 |
|  | *Anonyx sarsi* | 10 | 0.2604 |
|  | *Arcteobia anticostiensis* | 2 | 0.001 |
|  | *Arctica islandica* | 4 | 0.5066 |
|  | *Argissa hamatipes* | 2 | 0.0002 |
|  | *Aricidea catherinae* | 14 | 0.0102 |
|  | *Aricidea* sp. B | 4 | 0.0002 |
|  | *Asabellides* sp. | 8 | 0.0018 |
|  | Ascidiacea (solitary) | 52 | 0.5964 |
|  | *Brada villosa* | 2 | 0.0002 |
|  | *Brania* sp. | 4 | 0.0002 |
|  | Campanulariidae | 2 | 0.0028 |
|  | *Cancer borealis* | 4 | 0.5436 |
|  | *Cauloramphus* sp. | 2 | 0.0004 |
|  | *Celleporella* sp. | 2 | 0.0024 |
|  | Cephalaspidea | 24 | 0.0272 |
|  | Cerianthidae | 2 | 5.4324 |
|  | *Chaetozone* sp. A | 46 | 0.0602 |
|  | *Chone* sp. | 40 | 0.1034 |
|  | *Cistenides granulata* | 70 | 0.0494 |
|  | *Clymenura borealis* | 14 | 1.8836 |
|  | *Crangon septemspinosa* | 12 | 0.1012 |
|  | *Cucumaria frondosa* | 116 | 554.065 |
|  | *Cylichna alba* | 20 | 0.02 |
|  | *Cyrtodaria siliqua* | 60 | 0.353 |
|  | *Diaphana minuta* | 4 | 0.0072 |
|  | *Dichelopandalus leptocerus* | 4 | 0.448 |
|  | *Drilonereis magna* | 8 | 0.1436 |
|  | *Edotea montosa* | 2 | 0.0022 |
|  | *Edwardsia elegans* | 26 | 3.0762 |
|  | *Ericthonius fasciatus* | 6 | 0.0064 |
|  | *Eteone longa* | 8 | 0.0332 |
|  | *Eualus pusiolus* | 4 | 0.0172 |
|  | *Euchone papillosa* | 64 | 0.3608 |
|  | *Euclymene zonalis* | 2498 | 31.0994 |
|  | *Eucratea loricata* | 2 | 0.0048 |
|  | *Euspira* sp. | 2 | 0.0022 |
|  | *Exogone* sp. | 44 | 0.009 |
|  | Gastropoda | 14 | 0.0192 |
|  | *Glycera capitata* | 288 | 8.2844 |
|  | *Goniada maculata* | 8 | 0.0316 |
|  | *Harmothoe extenuata* | 32 | 0.057 |
|  | *Harmothoe imbricata* | 4 | 0.8966 |
|  | *Laphania boecki* | 18 | 0.24 |
|  | *Lumbrinerides acuta* | 46 | 0.718 |
|  | *Moelleria costulata* | 2 | 0.0046 |
|  | *Monoculodes* sp. | 20 | 0.0424 |
|  | *Munna fabricii* | 2 | 0.0002 |
|  | Mytiloidea | 86 | 3.5926 |
|  | Nemertea | 48 | 0.135 |
|  | *Nephtys caeca* | 2 | 11.1974 |
|  | *Nereis* sp. | 4 | 0.0528 |
|  | *Nothria conchylega* | 2 | 0.0786 |
|  | *Notomastus latericeus* | 10 | 0.2676 |
|  | Oligochaeta | 306 | 1.333 |
|  | *Ophiura* sp. juvenile | 20 | 0.0062 |
|  | *Orbinia swani* | 2 | 0.0046 |
|  | *Pagurus* sp*.* | 6 | 0.1262 |
|  | *Paraonis* sp. | 362 | 0.6006 |
|  | *Parougia eliasoni* | 2 | 0.0002 |
|  | *Parvicardium pinnulatum* | 66 | 0.2396 |
|  | *Periploma leanum* | 10 | 0.5002 |
|  | *Pholoe tecta* | 92 | 0.0498 |
|  | *Photis* sp. | 10 | 0.0046 |
|  | *Phoxocephalus holbolli* | 2 | 0.0006 |
|  | *Phyllodoce maculata* | 6 | 0.0084 |
|  | *Phyllodoce mucosa* | 2 | 0.004 |
|  | *Placopecten magellanicus* | 2 | 198.4 |
|  | *Platyhelminthes* | 16 | 0.1516 |
|  | *Pleusymtes glaber* | 18 | 0.0514 |
|  | *Polycirrus* sp. | 4 | 0.013 |
|  | *Polydora socialis* | 20 | 0.0212 |
|  | Polygordiidae | 356 | 0.7068 |
|  | *Pontogeneia inermis* | 2 | 0.0036 |
|  | *Protodorvillea gaspeensis* | 8 | 0.0008 |
|  | *Protodorvillea kefersteini* | 2 | 0.0002 |
|  | *Protomedeia fasciata* | 130 | 0.1226 |
|  | *Psolus* sp. | 98 | 0.1018 |
|  | *Rhizocaulus verticillatus* | 2 | 0.0898 |
|  | *Scoloplos armiger* | 10 | 0.1978 |
|  | *Sertularia* sp. | 2 | 0.0184 |
|  | *Spio filicornis* | 2 | 0.0058 |
|  | *Spiophanes bombyx* | 12 | 0.004 |
|  | *Strongylocentrotus pallidus* | 16 | 0.4002 |
|  | Syllidae | 40 | 0.0012 |
|  | *Syrrhoe crenulata* | 2 | 0.005 |
|  | *Tharyx* sp. | 56 | 0.1152 |
|  | *Thracia* sp. | 58 | 0.318 |
|  | *Travisia* sp. | 2 | 0.5584 |
|  | *Unciola irrorata* | 124 | 0.5306 |
| wh_vg07 | Actiniaria (burrowing) | 2 | 0.007 |
|  | *Ampharete finmarchica* | 30 | 0.3162 |
|  | *Anonyx sarsi* | 4 | 0.0234 |
|  | *Arcteobia anticostiensis* | 2 | 0.0082 |
|  | *Arctica islandica* | 72 | 151.91 |
|  | *Argissa hamatipes* | 2 | 0.0008 |
|  | *Aricidea catherinae* | 2 | 0.0004 |
|  | *Aricidea* sp. B | 2 | 0.0038 |
|  | Ascidiacea (solitary) | 4 | 0.1426 |
|  | *Capitella capitata* | 10 | 0.0266 |
|  | Capitellidae | 10 | 0.0036 |
|  | *Chaetozone* sp. A | 50 | 0.2086 |
|  | *Chone* sp. | 4 | 0.0038 |
|  | *Cistenides granulata* | 58 | 0.0638 |
|  | *Clymenura borealis* | 64 | 3.083 |
|  | *Corymorpha pendula* | 2 | 0.0042 |
|  | *Crangon septemspinosa* | 4 | 0.118 |
|  | *Cucumaria frondosa* | 10 | 0.0254 |
|  | *Cyrtodaria siliqua* | 16 | 646.2946 |
|  | *Diaphana minuta* | 2 | 0.0104 |
|  | *Echinarachnius parma* | 2 | 73.76 |
|  | *Edwardsia elegans* | 28 | 1.1688 |
|  | *Eteone longa* | 6 | 0.0048 |
|  | *Euchone papillosa* | 6 | 0.0382 |
|  | *Euclymene zonalis* | 340 | 8.4536 |
|  | *Euspira* sp. | 2 | 0.079 |
|  | *Exogone* sp. | 10 | 0.006 |
|  | *Glycera capitata* | 36 | 0.7416 |
|  | *Goniadella gracilis* | 4 | 0.0114 |
|  | *Harmothoe extenuata* | 6 | 0.0124 |
|  | *Levensinea gracilis* | 4 | 0.0022 |
|  | *Lumbrinerides acuta* | 32 | 0.405 |
|  | *Mactromeris polynyma* | 4 | 79.7 |
|  | *Mediomastus ambiseta* | 4 | 0.022 |
|  | Nemertea | 38 | 0.2068 |
|  | *Notomastus latericeus* | 6 | 0.4674 |
|  | Oligochaeta | 142 | 0.0982 |
|  | *Pagurus* sp. | 2 | 0.006 |
|  | *Paraonis* sp*.* | 108 | 0.1936 |
|  | *Parougia eliasoni* | 2 | 0.0002 |
|  | *Parvicardium pinnulatum* | 16 | 0.0052 |
|  | *Pherusa plumosa* | 2 | 0.01 |
|  | *Pholoe minuta* | 10 | 0.009 |
|  | *Photis* sp. | 2 | 0.0048 |
|  | *Phoxocephalus holbolli* | 4 | 0.0058 |
|  | *Phyllodoce mucosa* | 4 | 0.0106 |
|  | Platyhelminthes | 18 | 0.1748 |
|  | *Pleusymtes glaber* | 2 | 0.0008 |
|  | *Podoceropsis* sp. | 6 | 0.0036 |
|  | *Polydora socialis* | 6 | 0.0086 |
|  | Polygordiidae | 308 | 0.2888 |
|  | *Pontogeneia inermis* | 4 | 0.012 |
|  | *Protomedeia fasciata* | 54 | 0.0208 |
|  | *Serripes groenlandicus* | 2 | 0.0034 |
|  | *Spio filicornis* | 2 | 0.0072 |
|  | *Spiophanes bombyx* | 28 | 0.0076 |
|  | *Strongylocentrotus pallidus* | 4 | 0.0002 |
|  | *Strongylocentrotus* sp. | 4 | 0.0002 |
|  | Syllidae | 46 | 0.0302 |
|  | *Thracia* sp. | 24 | 0.0738 |
|  | *Tiron spiniferus* | 2 | 0.0044 |
|  | *Unciola irrorata* | 220 | 0.8522 |
| wh_vg08 | Actiniaria (burrowing) | 2 | 2.0118 |
|  | *Aglaophamus circinata* | 10 | 1.2336 |
|  | *Ampharete finmarchica* | 28 | 0.1022 |
|  | *Anonyx sarsi* | 2 | 0.0048 |
|  | *Arctica islandica* | 156 | 457.12 |
|  | *Argissa hamatipes* | 2 | 0.0008 |
|  | *Aricidea catherinae* | 4 | 0.0062 |
|  | *Aricidea* sp. A | 4 | 0.0076 |
|  | *Aricidea* sp. B | 2 | 0.0094 |
|  | *Aricidea wassi* | 16 | 0.0464 |
|  | Ascidiacea (solitary) | 14 | 0.2008 |
|  | *Capitella capitata* | 2 | 0.0076 |
|  | Capitellidae | 18 | 0.0052 |
|  | Cerianthidae | 2 | 15.5522 |
|  | *Chaetozone* sp. A | 6 | 0.0192 |
|  | *Chiridotea tuftsii* | 6 | 0.0424 |
|  | *Cirolana polita* | 12 | 0.3148 |
|  | *Cistenides granulata* | 14 | 0.011 |
|  | *Clymenura borealis* | 144 | 8.1174 |
|  | *Corophium crassicorne* | 20 | 0.0116 |
|  | *Crangon septemspinosa* | 8 | 0.1038 |
|  | *Cucumaria frondosa* | 6 | 0.0086 |
|  | Cumacea sp. A | 2 | 0.003 |
|  | *Cylichna alba* | 2 | 0.0028 |
|  | *Cyrtodaria siliqua* | 10 | 688.383 |
|  | *Diastylis sculpta* | 2 | 0.0044 |
|  | *Echinarachnius parma* | 52 | 1998.0862 |
|  | *Edwardsia elegans* | 30 | 1.3092 |
|  | *Euchone papillosa* | 8 | 0.0366 |
|  | *Euclymene zonalis* | 4 | 0.0524 |
|  | *Eudorellopsis deformis* | 2 | 0.0014 |
|  | *Euspira* sp. | 2 | 0.0036 |
|  | *Hippomedon serratus* | 18 | 0.256 |
|  | *Ischyrocerus* sp. | 2 | 0.0002 |
|  | *Leptognathia* sp*.* | 4 | 0.0004 |
|  | *Levensinea gracilis* | 2 | 0.0002 |
|  | *Lumbrinerides acuta* | 50 | 0.6538 |
|  | *Lumbrineris fragilis* | 2 | 0.2318 |
|  | *Mactromeris polynyma* | 2 | 40.5 |
|  | *Monoculodes* sp. | 2 | 0.0024 |
|  | Nemertea | 4 | 0.3244 |
|  | Nephtyidae sp. juvenile | 6 | 0.0524 |
|  | *Nephtys bucera* | 2 | 0.3848 |
|  | *Nereis* sp. | 4 | 0.1396 |
|  | *Nothria conchylega* | 2 | 0.001 |
|  | *Notomastus latericeus* | 10 | 0.0948 |
|  | *Ophelia limacina* | 16 | 2.7712 |
|  | *Orbinia swani* | 12 | 1.1868 |
|  | *Paraonis* sp. | 6 | 0.0036 |
|  | *Parvicardium pinnulatum* | 8 | 0.0062 |
|  | *Periploma leanum* | 2 | 0.0068 |
|  | *Phoxocephalus holbolli* | 2 | 0.0078 |
|  | *Phyllodoce mucosa* | 8 | 0.0156 |
|  | Platyhelminthes | 16 | 0.1286 |
|  | *Polycirrus* sp. | 4 | 0.005 |
|  | *Protomedeia fasciata* | 18 | 0.0094 |
|  | *Pseudounicola obliquua* | 46 | 0.0278 |
|  | *Scoloplos armiger* | 38 | 1.8064 |
|  | *Solariella obscura* | 4 | 0.1812 |
|  | *Spiophanes bombyx* | 26 | 0.1714 |
|  | *Tharyx* sp. | 14 | 0.0552 |
|  | *Travisia* sp. | 6 | 0.0558 |
|  | *Unciola irrorata* | 344 | 1.4644 |
| wh_vg09 | *Aglaophamus circinata* | 2 | 0.9342 |
|  | *Ampharete finmarchica* | 22 | 0.045 |
|  | *Arctica islandica* | 8 | 211.48 |
|  | *Aricidea wassi* | 4 | 0.0092 |
|  | Ascidiacea (solitary) | 6 | 0.0534 |
|  | *Cancer borealis* | 2 | 0.0126 |
|  | Capitellidae | 8 | 0.0118 |
|  | *Chaetozone* sp. A | 14 | 0.0236 |
|  | *Cistenides granulata* | 22 | 0.0116 |
|  | *Clymenura borealis* | 30 | 1.2724 |
|  | *Crangon septemspinosa* | 4 | 0.0418 |
|  | *Cucumaria frondosa* | 8 | 0.008 |
|  | *Cyrtodaria siliqua* | 8 | 458.314 |
|  | *Echinarachnius parma* | 18 | 770.003 |
|  | *Edwardsia elegans* | 16 | 0.5784 |
|  | *Eteone longa* | 2 | 0.0006 |
|  | *Euchone papillosa* | 4 | 0.0076 |
|  | *Euclymene zonalis* | 44 | 0.4772 |
|  | *Euspira* sp. | 2 | 0.103 |
|  | *Exogone* sp. | 2 | 0.0002 |
|  | *Glycera capitata* | 4 | 0.017 |
|  | *Harmothoe extenuata* | 2 | 0.0052 |
|  | *Hippomedon serratus* | 2 | 0.0088 |
|  | *Levensinea gracilis* | 24 | 0.016 |
|  | *Lumbrinerides acuta* | 18 | 0.237 |
|  | *Mactromeris polynyma* | 2 | 55.14 |
|  | Nemertea | 6 | 1.0196 |
|  | *Neosabellides* sp. | 6 | 0.005 |
|  | Nephtyidae sp. juvenile | 2 | 0.0068 |
|  | *Notomastus latericeus* | 4 | 0.053 |
|  | Oligochaeta | 10 | 0.0238 |
|  | *Ophelia limacina* | 2 | 1.8062 |
|  | *Ophiura* sp. juvenile | 2 | 0.014 |
|  | *Orbinia swani* | 4 | 0.4538 |
|  | *Paraonis* sp*.* | 12 | 0.0154 |
|  | *Periploma leanum* | 2 | 1.4292 |
|  | *Pholoe tecta* | 4 | 0.0036 |
|  | *Photis* sp. | 2 | 0.0014 |
|  | Platyhelminthes | 4 | 0.0162 |
|  | *Pleusymtes glaber* | 4 | 0.004 |
|  | *Polydora socialis* | 4 | 0.0018 |
|  | Polygordiidae | 24 | 0.0074 |
|  | *Protomedeia fasciata* | 6 | 0.0024 |
|  | *Scoloplos armiger* | 4 | 0.6276 |
|  | *Serripes groenlandicus* | 8 | 0.0044 |
|  | *Spio filicornis* | 2 | 0.0034 |
|  | *Spiophanes bombyx* | 46 | 0.0944 |
|  | Syllidae | 2 | 0.0002 |
|  | *Tharyx* sp*.* | 10 | 0.0502 |
|  | *Unciola irrorata* | 122 | 0.6198 |
| wh_vg10 | *Alvania* sp*.* | 2 | 0.0034 |
|  | *Ampharete finmarchica* | 50 | 0.1228 |
|  | *Anonyx sarsi* | 4 | 0.0084 |
|  | *Arctica islandica* | 4 | 12.56 |
|  | *Aricidea wassi* | 14 | 0.0326 |
|  | Ascidiacea (solitary) | 54 | 0.8388 |
|  | Asteroidea | 2 | 0.0004 |
|  | Capitellidae | 2 | 0.0026 |
|  | *Chaetozone* sp. A | 12 | 0.0286 |
|  | *Chiridotea tuftsii* | 2 | 0.0092 |
|  | *Cirolana polita* | 8 | 0.2752 |
|  | *Cistenides granulata* | 18 | 0.012 |
|  | *Clymenura borealis* | 74 | 8.601 |
|  | *Colus* sp. | 2 | 8.8354 |
|  | *Crangon septemspinosa* | 4 | 0.0812 |
|  | *Cylichna alba* | 4 | 0.0104 |
|  | *Echinarachnius parma* | 26 | 66.9648 |
|  | *Edotea montosa* | 2 | 0.025 |
|  | *Edwardsia elegans* | 10 | 0.0452 |
|  | *Euchone papillosa* | 6 | 0.0246 |
|  | *Euclymene zonalis* | 16 | 0.1266 |
|  | *Euspira* sp. | 2 | 0.0056 |
|  | *Exogone* sp. | 2 | 0.0002 |
|  | *Hiatella arctica* | 2 | 0.0014 |
|  | *Hippomedon serratus* | 32 | 0.2528 |
|  | *Levensinea gracilis* | 8 | 0.0026 |
|  | *Lumbrinerides acuta* | 40 | 0.5012 |
|  | *Macoma calcarea* | 2 | 0.0012 |
|  | *Mactromeris polynyma* | 8 | 125.64 |
|  | Nemertea | 8 | 1.5682 |
|  | Nephtyidae sp. juvenile | 10 | 0.1192 |
|  | *Notomastus latericeus* | 20 | 0.2962 |
|  | Nudibranchia Group 4b | 2 | 0.0006 |
|  | Oligochaeta | 2 | 0.0018 |
|  | *Ophelia limacina* | 10 | 2.1186 |
|  | *Ophiacantha bidentata* | 2 | 0.0316 |
|  | *Orbinia swani* | 2 | 0.1838 |
|  | *Pagurus* sp. | 4 | 0.0808 |
|  | *Pandora* sp. | 2 | 0.0078 |
|  | *Paraonis* sp*.* | 6 | 0.004 |
|  | *Parvicardium pinnulatum* | 2 | 0.0022 |
|  | *Periploma leanum* | 8 | 1.0204 |
|  | *Phyllodoce mucosa* | 8 | 0.1106 |
|  | Platyhelminthes | 2 | 0.014 |
|  | *Polydora caulleryi* | 2 | 0.0004 |
|  | Polygordiidae | 2 | 0.0002 |
|  | Porifera | 2 | 0.0936 |
|  | *Protomedeia fasciata* | 4 | 0.0004 |
|  | Sabellidae | 2 | 0.0002 |
|  | *Scoloplos armiger* | 6 | 0.3366 |
|  | *Solariella obscura* | 2 | 0.004 |
|  | *Spio filicornis* | 6 | 0.0078 |
|  | *Spiophanes bombyx* | 38 | 0.4888 |
|  | Syllidae | 2 | 0.0002 |
|  | *Tharyx* sp. | 30 | 0.0678 |
|  | *Travisia* sp. | 2 | 0.1018 |
|  | *Unciola irrorata* | 612 | 2.2556 |

See S1 Table for location and other information related to each video-grab code.
