# Supplementary material for: Influence of Benthic Macrofauna as a Spatial Structuring Agent for Juvenile Haddock (Melanogrammus aeglefinus) on the Eastern Scotian Shelf, Atlantic Canada
Source: PLoS One. 2016 Sep 20;11(9):e0163374. doi: 10.1371/journal.pone.0163374 (PMC5029893; doi:10.1371/journal.pone.0163374)
Supplement: S3 Table — (DOCX) [file pone.0163374.s005.docx]

**S3 Table. Summary of ANOVA of transformed Total Biomass (g m^-2^) testing for effect of Year, Bank and Haddock Abundance.**


|  | **Source** | **DF** | **Sum of Squares** | **F Ratio** | **Prob > F** |  |
| --- | --- | --- | --- | --- | --- | --- |
|  | Model | 11 | 298.5206 | 17.9918 | <0.0001 |  |
|  | Error | 108 | 162.9038 |  |  |  |
|  | Total | 119 | 461.4244 |  |  |  |
| Effect Tests |  |  |  |  |  |  |
|  | Year | 1 | 2.1048 | 1.3954 | 0.2401 |  |
|  | Bank | 2 | 74.6369 | 24.7409 | <.0001 |  |
|  | HA | 1 | 36.5813 | 24.2522 | <.0001 |  |
|  | Bank*HA | 2 | 31.9964 | 10.6063 | <.0001 |  |
|  | Bank*Year | 2 | 18.3411 | 6.0798 | 0.0031 |  |
|  | HA*Year | 1 | 10.8549 | 7.1964 | 0.0085 |  |
|  | HA*Year*Bank | 2 | 4.3722 | 1.4493 | 0.2393 |  |

| **Effect** | **Level** | **Least Sq Mean** | |  | **Std Error** | | | **Arithmetic Mean** | | **Std Dev** | | **Raw Mean** | | **Std Dev** | |
| --- | --- | --- | --- | --- | --- | --- | --- | --- | --- | --- | --- | --- | --- | --- | --- |
| Bank |  |  | |  |  | | |  | |  | |  | |  | |
|  | Emerald | 2.9799 | |  | 0.28215 | | | 2.9647 | | 0.8913 | | 30.31 | | 44.95 | |
|  | Sable Island | 5.0405 | |  | 0.27462 | | | 4.2495 | | 2.0071 | | 376.71 | | 686.13 | |
|  | Western | 5.6260 | |  | 0.27462 | | | 5.8908 | | 1.5846 | | 1425.50 | | 5001.98 | |
| HA |  |  | |  |  | | |  | |  | |  | |  | |
|  | Low | 5.3368 | |  | 0.2283 | | | 5.0299 | | 1.8373 | | 507.96 | | 705.45 | |
|  | High | 3.7608 | |  | 0.2242 | | | 3.9041 | | 1.9421 | | 751.63 | | 4114.28 | |
| Bank x HA |  | |  |  | |  |  | |  | |  | |  | |  |
|  | Emerald,Low | | 3.3099 |  | | 0.4094 | 3.3001 | | 1.0457 | | 46.66 | | 63.46 | |  |
|  | Emerald,High | | 2.6498 |  | | 0.3884 | 2.7055 | | 0.6655 | | 17.67 | | 14.28 | |  |
|  | Sable,Low | | 6.8627 |  | | 0.3884 | 5.9997 | | 1.5224 | | 843.48 | | 869.00 | |  |
|  | Sable,High | | 3.2184 |  | | 0.3884 | 2.9767 | | 1.1880 | | 37.24 | | 57.70 | |  |
|  | Western,Low | | 5.8379 |  | | 0.3884 | 5.6611 | | 1.6233 | | 620.41 | | 682.60 | |  |
|  | Western,High | | 5.4141 |  | | 0.3884 | 6.1314 | | 1.5451 | | 2268.93 | | 7112.84 | |  |


Abbreviations: DF= degrees of freedom; Least Sq Mean = Least Squares Mean of predicted value compared to nominal value calculated from model; Arithmetic Mean calculated from ln (X+1) transformed biomass; Raw Mean calculated from untransformed data (g m^-2^); Std Error = standard error; Std Dev = standard deviation; HA=juvenile Haddock Abbundance.
